# Guidelines on diagnosis and management of gastroesophageal reflux disease in infants, children and adolescents: a joint consensus from Italian pediatric societies (SIP and SIGENP) -Part II: management

**DOI:** 10.1186/s13052-026-02255-0

**Published:** 2026-04-10

**Authors:** Silvia Salvatore, Caterina Strisciuglio, Elena Bozzola, Stefania Cappa, Antonio Corsello, Giovanni Di Nardo, Maurizio Fuoti, Liliana Guadagni, Antonino Gulino, Chiara Mameli, Massimiliano Orso, Licia Pensabene, Renato Tambucci, Francesca Vassallo, Claudio Romano, Annamaria Staiano, of The Italian Society of Pediatric Gastroenterology Hepatology and Nutrition (SIGENP)

**Affiliations:** 1 Pediatric Unit, Department of Medicine and Technical Innovation, Hospital “F. Del Ponte, University of Insubria, Varese, Italy; 2 Department of Woman, Child and General and Specialized Surgery, University of Campania “Luigi Vanvitelli”, Via Luigi De Crecchio, 2- 80138 Naples, Italy; 3 Pediatric Unit, Bambino Gesù Children’s Hospital, IRCCS, 00165 Rome, Italy; 4 Associazione Italiana Neonati Reflussanti (AINER), Milan, Italy; 5 Department of Pediatrics, Istituto Mediterraneo per i Trapianti e Terapie ad Alta Specializzazione - IRCCS ISMETT, UPMC, Palermo, Italy; 6 Department of Clinical-Surgical, Diagnostic and Pediatric Sciences, University of Pavia, Pavia, Italy; 7 Department of Neurosciences, Mental Health and Sensory Organs (NESMOS), Sapienza University of Rome, Rome, Italy; 8 Pediatric Gastroenterology and Endoscopy Unit, Department of Pediatric Specialties, Santobono Pausilipon Children’s Hospital, Naples, Italy; 9 Pediatric Gastroenterology and Endoscopy Unit, Children’s Hospital, ASST Spedali Civili, Brescia, Italy; 10 Department of Surgical and Biomedical Sciences, University of Perugia, Perugia, Italy; 11 Family Pediatrician, ASP Catania, Italy; 12 Department of Pediatrics, V Buzzi Children’s Hospital, Università di Milano, Milan, Italy; 13 Department of Biomedical and Clinical Science, Università di Milano, Milan, Italy; 14 C.R.E.A. Sanità (Centre for Applied Economic Research in Healthcare), Rome, Italy; 15 Pediatric Unit, Department of Medical and Surgical Sciences, University “Magna Graecia” of Catanzaro, 88100 Catanzaro, Italy; 16 Gastroenterology and Nutrition Unit, Bambino Gesù Children’s Hospital IRCCS, Rome, Italy; 17 General and Specialty Pediatrics Unit, Department of Integrated Activities for Mothers and Children, University of Naples “Federico II”, Naples, Italy; 18 Pediatric Gastroenterology and Cystic Fibrosis Unit, Department of Human Pathology in Adulthood and Childhood “G. Barresi”, University of Messina, 98125 Messina, Italy; 19 Unit of Pediatrics, Department of Translational Medical Science, Section of Paediatrics, University of Naples “Federico II”, A.O.U. “Federico II”, Naples, Italy

**Keywords:** Gastroesophageal reflux, Children, GERD, PPIs, Esophagitis, Esophageal impedance pH monitoring, Alginate, Diet, Surgery

## Abstract

**Background:**

Suspected gastroesophageal reflux disease (GERD) is one of the most common reasons for referral to pediatric and gastroenterology clinics, and proton pump inhibitors (PPIs) are frequently prescribed. Currently, there is still wide heterogeneity in treatment, especially in neonates and extraesophageal presentations. This article aimed to provide guidelines and a consensus of Italian experts on the management of GERD in infants, children, and adolescents, in order to improve the care and treatment of these patients.

**Methods:**

A multidisciplinary pediatric panel identified four key clinical questions (PICOs) regarding pharmacological and non-pharmacological treatments, surgical intervention, and prognosis of pediatric GERD. Four databases (PubMed/Medline, Embase, Web of Science, and Google Scholar) were searched from inception to May 2024, using a specific search string for each PICO and limiting the search to children (0–18 years) and to English language. Previous pediatric guidelines, systematic reviews, and clinical trials focused on the treatment of GERD were considered to formulate evidence-based recommendations. The Grading of Recommendations Assessment, Development and Evaluation (GRADE) and AGREE II systems were used to assess study quality; a 9-point Likert scale was used to rate the recommendations. A two-round Delphi method was conducted to reach consensus, defined as ≥ 80% agreement or disagreement.

**Results:**

The systematic review identified three previous pediatric guidelines, eight systematic reviews, 83 RCTs, and three observational studies. The panel provided 26 recommendations regarding the management and treatment of GERD in pediatric patients. All reached consensus, with the majority expressing strong support for the intervention. The panel also provided practice insights for each PICO to improve the clinical application and clarity of these guidelines.

**Conclusion:**

Regurgitation is common in infants, but in most cases, no treatment is necessary. When regurgitation causes discomfort, a brief trial of alginate or thickened formulas or a cow’s milk elimination diet may be considered, particularly if other symptoms, including crying and dermatitis, are associated. For children who complain of heartburn or have severe neurologic deficits, a short course of empirical PPIs treatment may be considered, monitoring the clinical response and testing if no improvement is seen. PPIs are recommended in infants and children with reflux esophagitis, or acid reflux detected by esophageal pH monitoring. Children at high- risk for chronic GERD requiring prolonged treatment and multidisciplinary care have been identified. Surgery should be considered for severe or complicated GERD after a comprehensive diagnostic workup and optimal pharmacological management.

**Supplementary Information:**

The online version contains supplementary material available at 10.1186/s13052-026-02255-0.

## Introduction

Gastroesophageal reflux disease (GERD) causes bothersome symptoms and/or complications that require specific management, including a nonpharmacological and dietary approach, particularly in infants, PPIs for acid GERD or reflux esophagitis, and, in selected severe cases, surgery [1–4].

In infants and in the presence of extraesophageal symptoms, acid suppressant agents are often prescribed in the absence of a clear diagnosis of GERD and without evidence of clinical benefit [5–8].

PPIs are considered appropriate for treating heartburn and epigastric pain in older children and adolescents or in cases of severe neurological impairment [2, 7]. However, uncertainty and ineffective therapeutic approaches have been reported worldwide [3, 9–11]. Furthermore, different GERD phenotypes and children at risk for persistent and severe GERD, requiring individualized treatment, have recently been described [2, 12].

The purpose of these guidelines is to provide pediatricians, primary care physicians, and gastroenterologists with an updated summary of evidence, recommendations, and practical guidance to aid and improve the management of GERD in infants, children, and adolescents.

## Methods

The complete description of the methods, the literature search strategy, the Prisma flowcharts, the results of the quality assessment of clinical studies and systematic reviews are reported in Part I of these Guidelines and in Additional Files 1–5.

In brief, these Guidelines were developed by a multidisciplinary panel composed of pediatricians with experience in gastroenterology and/or clinical guidelines, the President of the Italian Association of Neonates with Reflux (AINER), a representative of family pediatricians, and an external agency (CREA Sanità) with experience in data analysis and applied research in the healthcare field. A systematic literature search was conducted using PubMed, Embase, Web of Science, and Google Scholar, including previous guidelines, systematic reviews on pediatric GERD, and randomized clinical trials limited to children (0–18 years), published in English, from 1983 to May 2024. Additional studies were retrieved by manual review of references of articles identified by the systematic review.

The panel defined eight clinical questions on pediatric GERD (Table 1); the last four questions concern the treatment of GERD and are discussed in this guideline.

**Table 1:** List of the last four clinical questions identified as relevant for this guideline

Questions	PICO
5. What is the evidence of effectiveness of pharmacologic treatment for GER and GERD?	YES
6. What is the effectiveness of different non-pharmacologic treatment options for GER and GERD?	YES
7. What is the indication and the effectiveness of different surgical/endoscopic treatment options for GERD?	YES
8. What is the prognosis of GER and GERD and what are prognostic factors?	NO

Due to the descriptive nature, Question 8 was considered as a narrative question.

Improved diagnostic accuracy, appropriate use of pharmacological and non-pharmacological treatments, and improved patient outcomes are among the expected benefits of these guidelines and expert consensus. Pediatricians, pediatric gastroenterologists, primary care physicians, and other healthcare professionals treating children with suspected or confirmed GERD are the intended audience for this guideline.

The panel evaluated the efficacy and safety of pharmacological and non-pharmacological treatments in reducing GERD symptoms and treating or preventing GERD complications. Where applicable, comparators included placebo, no treatment, standard care, or alternative diagnostic or therapeutic interventions. Studies conducted in any healthcare setting without geographic restrictions were examined.

## Certainty assessment

The GRADE approach [13] was applied to assess the certainty of evidence for PICO questions 5, 6 and 7. The certainty of evidence was rated as high, moderate, low, or very low, based on the domains of risk of bias, inconsistency, indirectness, imprecision, and publication bias (Additional File 4). Inconsistency was evaluated based on the presence of unexplained heterogeneity across study results. For indirectness, we evaluated whether the populations, interventions, comparators and outcomes considered in the included studies correspond to those planned in the PICO questions of this Guideline. We downgraded for imprecision if the effect estimates were from studies with a small sample size and wide confidence intervals. Factors that may increase the certainty level, such as large magnitude of an effect, dose-response gradient, and effect of plausible residual confounding, were also considered. GRADE Evidence profile tables were created by GRADEpro GDT software [14] (Additional File 4).

## Evidence to decision framework

A formal Evidence to Decision (EtD) framework was applied for PICO questions 5 to 7 and the following domains were systematically evaluated:

- Priority of health problems.
- Desirable and Undesirable Effects Certainty of evidence of effects.
- Value, uncertainty or variability about the main outcomes.
- Balance of desirable and undesirable effects comparing the intervention and the control.
- Resources required, costs and costs effectiveness.
- Health Equity, Acceptability and feasibility of the intervention.

Based on the judgements given on these domains, the expert panel proposed the strength of recommendations (SoR) as strong/weak against/in favor of the intervention (Additional File 5).

## Consensus process

Recommendations, including their direction and strength, were developed through a combination of structured discussion and iterative voting. A two-round Delphi process was conducted between April and May 2025. Consensus was defined as ≥ 80% agreement (score 7–9) or disagreement (score 1–3) on a 9-point Likert scale.

## External review

To improve the validity, feasibility and clarity of this Guideline the manuscript was sent for comments, before submission, to external experts on GERD (Osvaldo Borrelli, Yvan Vandenplas, Mario Vieira, and the Presidents of Italian Federation of Societies of Digestive Diseases (FISMAD)), to the President of Italian Pediatric Society of Neonatology (SIN) and of an Association of Italian Family Pediatricians, not involved in the development process. Their suggestions and changes were incorporated in the final text of this Guideline.

### Facilitators and barriers to application

The panel qualitatively considered facilitators and barriers to implement the guideline recommendations during the development process, specifically within the Evidence to Decision (EtD) framework for PICO questions 5 to 7. Although no formal pilot testing or stakeholder surveys were conducted, the panel drew on its clinical experience in various healthcare facilities in Italy, including tertiary hospitals, community hospitals, and pediatric outpatient services. Considerations regarding potential facilitators and barriers were discussed during the formulation of the recommendations and helped guide decisions regarding their effectiveness, as well as the inclusion of non-pharmacological and context-sensitive options where evidence-based, or the formulation of recommendations to allow for flexibility based on available resources and clinical judgment.

### Implementation tools and advice

**Fig. 1:**
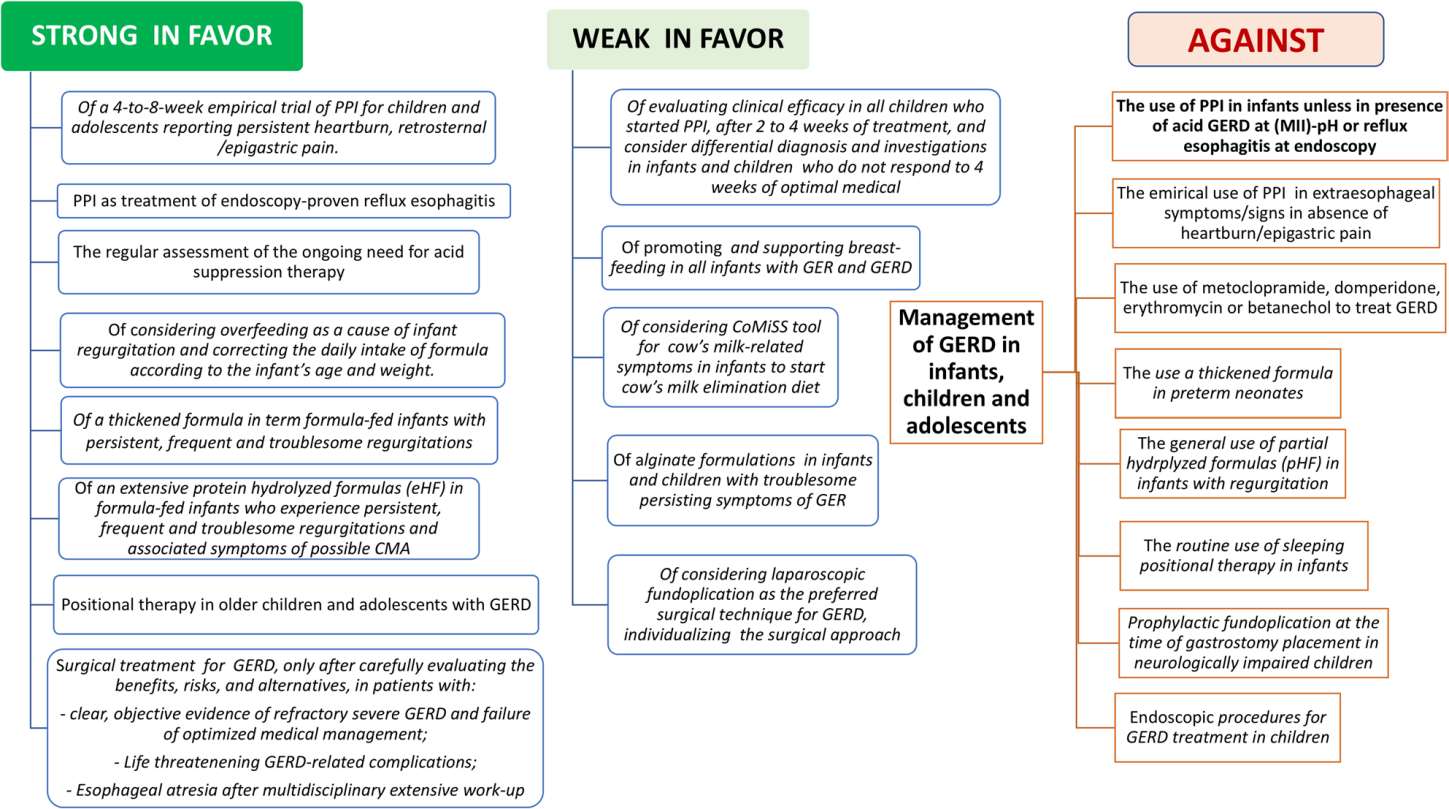
A simplified summary of the management of GER and GERD in infants, children and adolescents, according to the strength of recommendations

**Fig. 2:**
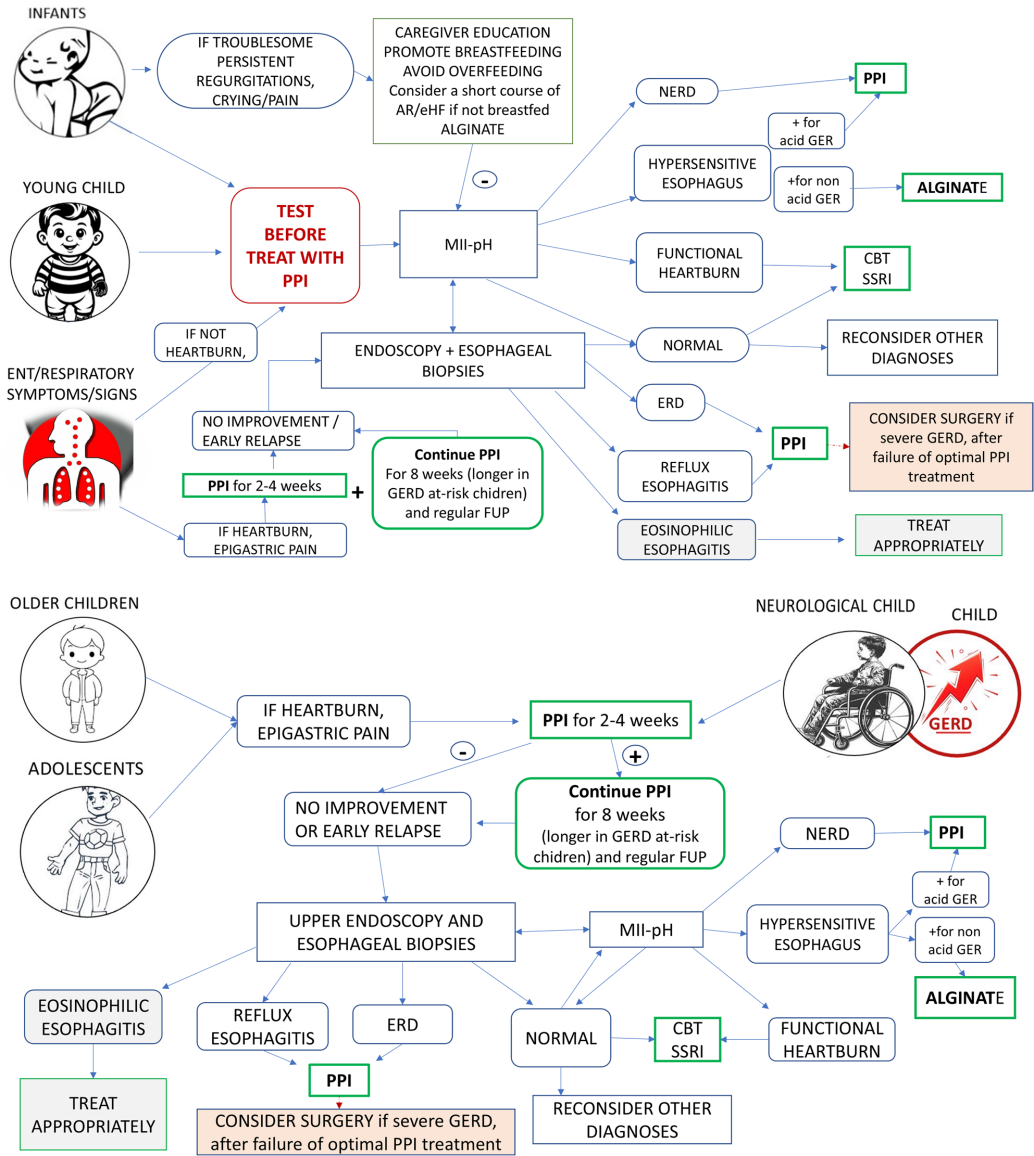
A simplified algorithm that summarizes the management of GERD in different pediatric settings. See the text for further details and explanations

To support the practical application of these guidelines, the recommendations were presented after each PICO question, providing their strength, and orientation. Two figures (Figs. 1 and 2) were created to represent the strength of recommendations for or against different treatments in infants, children, and adolescents and the treatment algorithm for pediatric GERD in different clinical scenarios. These tools are included to facilitate the readiness of guideline content and consistent application in clinical practice across different settings, including hospitals and outpatient clinics.

### Resource implications

No formal systematic search for economic evaluations or cost-effective studies was conducted or located within the scope of these guidelines. However, considerations regarding potential resource implications were discussed by the panel when formulating recommendations for PICO questions 5–7 (pharmacological, non-pharmacological, and surgical/endoscopic treatments). The panel considered, where applicable, the availability and costs of interventions in the Italian healthcare context, including drug acquisition, hospital procedures, and access to specialized services. Although quantitative cost data were not formally incorporated, qualitative judgments regarding the feasibility and sustainability of implementing each intervention were explicitly documented and contributed to the final formulation of the recommendations.

### Monitoring and auditing criteria

No formal set of monitoring or audit tools has yet been developed for this guideline. However, as the committee recognizes the importance of monitoring the implementation and concrete impact of the recommendations, a set of key process and outcome indicators will be proposed during the dissemination phase, in collaboration with national pediatric and gastroenterology societies and patient organizations. Potential indicators will include:

- Adherence to recommended diagnostic pathways (e.g., appropriate use of pH-impedance monitoring or upper GI endoscopy).
- Appropriate prescription of pharmacological treatments (e.g., PPIs limited to evidence-based indications and durations).
- Reduction in the use of ineffective or non-recommended interventions.
- Surgical referrals based on agreed clinical criteria.

Data will be collected through surveys or standardized data collection forms in the coming years.

Where possible, biennial-quadrennial audits will be encouraged to assess adherence and identify barriers to implementation. The panel will support the integration of these indicators into national and international networks to facilitate ongoing monitoring.

### Update procedure

The panel plans to regularly update these Guidelines every 5–7 years, in line with emerging evidence and evolving clinical practice, to ensure optimal management and clinical care of pediatric patients with GERD.

## Overall results

The results of each PICO systematic review and the included studies are reported in additional files (Files 1–5). The panel formulated 26 recommendations regarding the management of gastroesophageal reflux (GER) and GERD across paediatrics age groups and provided clinical practice suggestions, reported after each related PICO section, to improve clinical guidelines and practice approaches.

All recommendations reached consensus: 18 were rated strongly in favor and 8 were rated weakly in favor (Fig. 1). The management algorithm, including the different possible clinical scenarios, is illustrated in Fig. 2. A section dedicated to specific conditions has been added at the end of this document to assist clinicians treating complex pediatric patients.

## Management

### PICO Question 5: What are the effective and safe pharmacological treatment options to reduce the signs and symptoms of GERD?

#### Recommendation 5.1. Use of PPIs in infants

*We recommend against the use of PPIs to treat crying/distress or regurgitation*,* or other signs/symptoms included in the Infant Gastroesophageal Reflux Questionnaire Revised (I-GERQ-R) in otherwise healthy infants.*

AGREEMENT (scores 7–9:100%); SOR: 91.67% strong in favor; 8.33% weak in favor.

#### Recommendation 5.2. Use of PPIs in older children and adolescents

*We recommend a 4- to 8-week empirical study of PPIs in children and adolescents reporting persistent heartburn*,* retrosternal*,* or epigastric pain*,* and monitoring for clinical improvement.* AGREEMENT (scores 7–9:100%); SOR: 100% strong in favor.

#### Recommendation 5.3 Use of PPIs in reflux esophagitis

*We recommend using PPIs as first-line treatment of reflux-related upper GI endoscopy-proven reflux esophagitis in infants and children.*

AGREEMENT (scores 7–9:100%); SOR: 81.8% strong in favor; 18.2% weak in favor.

#### Recommendation 5.4. Use of PPIs in extraesophageal symptoms

*We recommend against the use of empirical PPIs in patients with extraesophageal symptoms (i.e.*,* cough*,* wheezing*,* asthma) in the absence of heartburn or retrosternal/epigastric pain and/or diagnostic testing suggestive of acid GERD or reflux esophagitis.*

AGREEMENT (scores 7–9:100%); SOR: 100% strong in favor.

#### Recommendation 5.5. Evaluation of efficacy of PPIs

*We suggest evaluating clinical efficacy in all children who started PPIs after 2 to 4 weeks of treatment. We also suggest considering differential diagnosis and investigations into infants and children not responding to 4 weeks of optimal medical therapy*,

AGREEMENT (scores 7–9:90 − 9%; scores 4–6:9.1%); SOR: 72.7% strong in favor; 27.3% weak in favor.

#### Recommendation 5.6. Monitoring of children on PPIs

*We recommend regular assessment of the need for ongoing acid suppression therapy in infants and children with GERD and long-term treatment.* AGREEMENT (scores 7–9:100%); SOR: 91.67% strong in favor; 8.33% weak in favor.

#### Recommendation 5.7. Metoclopramide, domperidone, erythromycin or bethanechol

*We do not recommend the use of metoclopramide*,* domperidone*,* erythromycin or bethanechol to treat GERD in infants and children.*

AGREEMENT (scores 7–9:100%); SOR: 83.33% strong in favor; 16.67% weak in favor.

### Study selection and characteristics

Our search strategy included 45 articles [15–59]. Prisma flow diagram, reasons for studies exclusion and characteristics of included studies are shown in Additional File 1 and File 2. Among the 45 included studies, there were 32 RCTs, seven randomized crossover trial [20, 34, 38, 44, 45, 48, 53] and six systematic reviews [15, 16, 31, 39, 43, 52]. Only 4 studies enrolled more than 100 patients [40, 46, 55, 58]. Placebo was the comparator in the control arm in all studies except 7 [17, 18, 20, 23, 29, 54, 59]. Many records (30 out of 45) reported safety outcomes and adverse events. Additional studies retrieved by the references of the included studies were included by the panel to provide the summary of evidence of efficacy and safety of pharmacological agents for treating GERD in pediatric population. In total 53 studies were considered: 47 were RCTs and 6 were systematic reviews.

### Summary of evidence

#### Acid-suppressant drugs: proton pump inhibitors (PPIs) and histamine H2 receptor antagonists (H2RAs)

##### PPIs vs. placebo

Eleven pediatric studies compared the efficacy of PPIs with placebo in pediatric GERD [15, 32, 35, 37, 40, 44–46, 51, 57, 58]. Seven studies focused on infants aged 1 to 12 months, and four enrolled children older than 12 months [15, 35, 37, 51]. The diagnosis of GERD varied across the studies and was based on pH monitoring, endoscopic findings, clinical symptoms, or I-GERQ-R score > 16. Various PPIs were assessed: lansoprazole, esomeprazole, rabeprazole, dexlansoprazole, pantoprazole, and omeprazole. All studies and three systematic reviews [52, 60, 61] showed that, in infants, PPIs were not significantly more effective than placebo to improve symptoms including regurgitation, crying, irritability, coughing, or back-arching attributed to GERD, despite reducing esophageal acid exposure.

In older children and adolescents reporting heartburn, a short course of PPIs therapy (two to four weeks) is effective in the ones in which acid GERD is associated, with no clear evidence that one PPIs is better than another [2, 60]. In severe GERD and at-risk individuals, prolonged or recurrent treatment is often needed. Halving the dose of PPIs for at least two weeks before suspension is suggested to avoid acid rebound and reappearance of symptoms.

There is emerging evidence of children and adolescents reporting heartburn that does not improve when treated with PPIs [2, 12]. In these subjects, investigations including upper GI endoscopy with esophageal biopsies and MII-pH are recommended to identify eosinophilic esophagitis and different phenotype of GERD, including NERD, hypersensitive esophagus and functional heartburn that required different treatment.

In infants and in children, PPIs are the first-choice drug for treating reflux esophagitis and acid GERD detected by MII-pH monitoring. The effective dose ranged between 1 and 1.7 mg/kg/day up to 20 mg/day in older children and 40 mg/day in adolescents, taken once daily, 30 min before breakfast, for 8–12 weeks [2, 23, 62]. In 2019, Gremse et al. showed that dexlansoprazole healed erosive esophagitis within 8 weeks in 88% of patients [37]. Borrelli et al. [63] evaluated lansoprazole, alginate, and their combination in children aged 12 months to 12 years with GERD. The study suggested that combining alginates and lansoprazole may offer additional benefits compared to either drug alone, although the small sample size warrants cautious interpretation.

Two RCTs [51, 64] demonstrated that omeprazole and lansoprazole did not significantly improve asthma control or pulmonary functions in children. However, in 2022, Yagoubi et al. [59] reported that the control of asthma was more frequent in children treated with omeprazole for 6 months than with placebo (84.8% vs. 11.5%; *p*<0.0001). Noteworthy, among the 102 children recruited, fifty-nine patients (58%) showed acid reflux at pH-monitoring.

##### PPIs vs. ranitidine

There is no clear evidence that PPIs are significantly better than high dose of ranitidine in improving GERD symptoms [18, 52, 65]. Cucchiara et al. [29] reported similar effect of both treatment (omeprazole 40 mg/day/1.73 m^2^ surface area vs. ranitidine 20 mg/kg/day) in healing esophagitis in a small group of children.

##### Other H2RAs vs. placebo, antacids, alginates and sucralfate

Four blind RCTs evaluated different H2RAs—famotidine, cimetidine, and nizatidine—in small-size pediatric populations with GERD [28, 47, 50, 66]. Orenstein et al. (2003) [47] examined the effects of famotidine at 0.5 mg/kg/day in infants diagnosed with GER, observing a reduction in regurgitation frequency compared to baseline but noting adverse effects including irritability and drowsiness. Long before, Simeone et al. [50] reported healing of esophagitis with nizatine (Simeone) and Cucchiara et al. (1989) [28] reported symptomatic and endoscopic improvements in children treated with cimetidine, without a significant impact on symptoms. Furthermore, Cucchiara et al. [66] did not show a significant benefit for treating esophagitis with cimetidine compared to a magnesium-aluminium hydroxide mixture in infants and children.

##### Safety concerns about acid suppressant drugs

Adverse events in children treated with PPIs ranged from 47% to 72% and most commonly include diarrhea, upper respiratory tract infections, fever, and rash. Orenstein et al. [46] reported a significant increase in lower respiratory tract infections in children on lansoprazole vs. placebo (10 vs. 2 events; *p* = 0.032). Hussain et al. [40] noted elevated serum gastrin levels (potentially indicating rebound acid hypersecretion) in 5% of children on rabeprazole. Winter et al. [58] reported no significant differences in safety between pantoprazole and placebo and Gremse et al. [37] noted that dexlansoprazole was well-tolerated in adolescents, with only mild adverse effects (headache, abdominal pain). A meta-analysis evaluating six studies reporting the outcomes of more than 900,000 children and young adults treated with acid suppressant drugs, reported a pooled relative risk of 1.17 (95% CI = 1.1–1.25; *P* < 0.001) for fracture with the use of PPIs versus non-use of these medications in children. However, causality and the role of unmeasured/residual confounding factors in this association were not defined [67]. A French nationwide cohort study, including 606,645 children who received PPIs, showed that PPIs exposure was associated with an increased risk of serious infections overall (aHR, 1.34; 95% CI, 1.32–1.36), for the digestive tract, ear and respiratory tract, kidneys and urinary tract, and nervous system (aHR, 1.31; 95% CI, 1.11–1.54) and for both bacterial (aHR, 1.56; 95% CI, 1.50–1.63) and viral infections (aHR, 1.30; 95% CI, 1.28–1.33) [6]. A systematic review by Alla et al. (2024) [15] including 30 studies (18 RCTs and 12 cohort studies, with a total of 762,505 children with a mean age of 7.39 ± 4.69 years) investigated the incidence of adverse events across six different PPIs. Gastrointestinal adverse events, especially diarrhea and respiratory infections, were most commonly reported, consistent with previous findings. Still, case-control studies suggested an increased risk of infections (necrotizing enterocolitis, pneumonia, sepsis, urinary tract infections, and Clostridium difficile infection) in infants and children on acid-suppressive therapy.

Importantly, since 2019 Italian, European and American Drug Agencies (AIFA, EMA and FDA) and other regulatory bodies in various countries requested the removal of all ranitidine products from the market. Ranitidine was withdrawn due to safety concerns related to the contaminant N-Nitrosodimethylamine (NDMA), found in some batches and belonging to nitrosamine class, that has been linked to an increased risk of cancer in animal studies.

As for the limited efficacy on symptoms and the risk of adverse effects inappropriate or prolonged use of acid suppressant drugs should be avoided, limiting treatment to confirmed GERD cases and regularly monitoring therapy to ensure minimal effective dose and the shortest possible duration. Given the high prescription rates, further research on PPIs safety in pediatric populations is critical for optimal care of these patients.

#### Prokinetics

##### Domperidone and metoclopramide

Both domperidone and metoclopramide are antidopaminergic agents that act on gastric emptying. De Loore et al. [33] conducted a two-week double-blind trial in 47 infants and children, observing a significant reduction in vomiting episodes among those receiving domperidone or metoclopramide compared to placebo (*p* < 0.001), with higher effectiveness with domperidone than metoclopramide (*p* < 0.05). Similarly, Carroccio et al. [24] found that domperidone—mainly when combined with antacids—reduced clinical symptoms and improved pH-monitoring results compared to placebo. However, they did not provide individual data (resulting in very low-quality evidence). Notably, none of these studies reported adverse events, although the overall evidence quality was very low, leaving uncertainty about any potential risks associated with domperidone use in this population.

##### Safety concerns about domperidone and metoclopramide

A meta-analysis on metoclopramide safety, reviewing 108 studies (57 prospective), indicated extrapyramidal symptoms (9%; 95% CI 5%–17%), diarrhea (6%; 95% CI 4%–9%), and sedation (6%; 95% CI 3%–12% in multiple-dose studies) as the most frequent adverse events [39].Arrhythmias, respiratory failure, neuroleptic malignant syndrome, and tardive dyskinesia have been rarely associated with metoclopramide. Its therapeutic window is narrow, and some regulatory agencies have restricted its use in neonates and children due to neurological risk [39]. Similar concerns exist for domperidone, associated with QTc prolongation and cardiac adverse events (serious ventricular arrhythmias and sudden cardiac death in rare cases). Extrapyramidal side effects are also possible. Given insufficient clinical efficacy and risk of adverse effects, they are not recommended in infants and children with GERD [2].

##### Cisapride

Cisapride is a mixed serotonergic agent that promotes the release of acetylcholine in the myenteric plexus, enhancing gastric emptying and esophageal motility. However, cisapride has been progressively withdrawn from the market since 2000 or restricted in several countries due to the risk of QTc prolongation and potentially fatal cardiac arrhythmias, particularly when associated with certain other medications.

##### Erythromycin and bethanechol

The literature search identified only one RCT on premature infants with suspected GERD, testing erythromycin ethylsuccinate 50 mg/kg/day vs. placebo [19]. There was no statistical difference between the intervention and control groups in the number of reflux episodes, detected by MII-pH, and in cardiorespiratory events (apnea, bradycardia, desaturation). No adverse events were reported [19].

Erythromycin and azithromycin, that are motilin agonists, are not approved by the FDA for GERD treatment. Bethanechol, a direct cholinergic agonist, is not FDA-approved for pediatric use because of uncertain efficacy and high risk of adverse events.

##### Baclofen

A systematic review published in 2023 included 26 studies performed in adult and children with GERD and showed improvement in symptoms and in esophageal manometry or pH monitoring parameters using baclofen, a gamma-amino-butyric-acid B receptor agonist that may inhibit transient lower esophageal sphincter relaxation (TLESR). Mild neurological and mental status deterioration were the most commonly reported side effects, occurring in 5% of patients on short-term treatment and in 20% of patients using it long-term [16].To date, two RCTs have been published assessing the effect of baclofen in pediatric patients one investigated children with GERD and assessed a single dose of baclofen [68] and the other monitored only symptoms [69].

In the Australian double-blind RCT study, manometry and pH-monitoring were performed in 30 children for four hours: two hours after infusion of 250 ml of cow’s milk and two hours after another infusion of cow’s milk combined with a single dose of 0.5 mg/kg baclofen or placebo. Baclofen significantly reduced the TLESR events and acid GER episodes while accelerating gastric emptying time measured through (13) C octanoate breath test [68].

**Table:** 

PRACTICE POINTS
• PPIs are the primary medications for children and adolescents with acid GERD symptoms, and reflux esophagitis.
• A 4- to 8-week treatment with once-daily PPIs at dose of 1 to 1.7 mg/kg/day, up to 20 mg/day in older children and 40 mg/day in adolescents, is effective in most patients with acid GERD without risk factors or other conditions.
• Empirical PPIs use should be avoided in otherwise healthy infants experiencing crying, irritability or regurgitation/vomiting, and in children with extraesophageal symptoms.
• Other antacids and prokinetics should not be routinely used to treat GERD.
• Safety concerns, including potential adverse effects of PPIs and other agents, require careful monitoring and, if possible, a short duration of treatment.

### PICO question 6: What is the effectiveness of different non-pharmacologic treatment options for GER and GERD?

#### Recommendation 6.1.1 Breastfeeding

*Based on expert opinion*,* the panel recommends promoting and supporting breast-feeding in all infants with GER and GERD.*

AGREEMENT (scores 7–9:100%); SOR: 72.7% strong in favor; 27.3% weak in favor.

#### Recommendation 6.1.2. Consider overfeeding

*Based on expert opinion*,* the panel recommends considering overfeeding as a cause of infant regurgitation and correcting the daily intake of formula according to the infant’s age and weight.*

AGREEMENT (scores 7–9:100%); SOR: 91.67% strong in favor; 8.33% weak in favor.

#### Recommendation 6.1.3. Thickened formulas

*The panel suggests considering the use of a thickened formula in term formula-fed infants who experience persistent*,* frequent and troublesome regurgitations that do not improve after parental reassurance and education. The panel recommends regular monitoring of symptom improvement and of the need for protracted intervention.*

AGREEMENT (scores 7–9:90 − 9%; scores 4–6:9.1%); SOR: 83.33% strong in favor; 16.67% weak in favor.

#### Recommendation 6.1.4. Thickened formulas in preterm neonates

*The panel recommends not to use a thickened formula in preterm neonates due to minimal data and potential risks in this vulnerable population.*

AGREEMENT (scores 7–9:100%); SOR: 91.67% strong in favor; 8.33% weak in favor.

#### Recommendation 6.2.1. Extensive protein hydrolyzed formulas

*The panel recommends considering the use of an extensive protein hydrolyzed (eHF) in formula-fed infants who experience persistent*,* frequent and troublesome regurgitations*,* which do not improve after parental reassurance and education and are associated with other symptoms of possible Cow’s milk allergy (CMA).*

AGREEMENT (scores 7–9:90.9%; scores 4–6:9.1%); SOR: 91.67% strong in favor; 8.33% weak in favor.

#### Recommendation 6.2.2. CoMiSS tool

*The panel suggests that the CoMiSS tool can be considered a practical tool for raising awareness of possible cow’s milk-related symptoms in infants but is not diagnostic of CMA. The panel recommends a thorough medical history*,* physical assessment*,* and a clear benefit from a 2–4 week trial of eHF followed by an oral challenge with cow’s milk to confirm or rule out CMA.* AGREEMENT (scores 7–9:81.8%; scores 4–6:8.1%; scores 1–3:8.1%); SOR: 66.67% strong in favor; 33.33% weak in favor.

#### Recommendation 6.2.3. Partial hydrolyzed formulas (pHF)

*The panel does not recommend the general use of partially protein hydrolyzed formulas in infants with regurgitation or infants with suspect symptoms of CMA.*

AGREEMENT (scores 7–9:100%); SOR: 83.33% strong in favor; 16.7% weak in favor.

#### Recommendation 6.3. Probiotics

*Based on limited evidence and heterogeneity in the intervention trials*,* the panel cannot formulate any specific recommendation for or against using probiotics to prevent or treat infant regurgitation.*

AGREEMENT (scores 7–9:100%); SOR: 91.67% strong in favor; 8.33% weak in favor.

#### Recommendation 6.4. Alginate formulations

*The panel suggests that alginate formulations may be considered in infants and children with troublesome persistent symptoms of GER that cannot be adequately managed through conservative measures. Efficacy should be reassessed after 1 to 2 weeks and regularly monitored with attempts of discontinuation to avoid unnecessary protracted use. Caution should be exercised*,* particularly in subjects at risk of intestinal obstruction*,* renal impairment*,* or on concomitant thickened formulas.*

AGREEMENT (scores 7–9:100%); SOR: 72.7% strong in favor; 27.3% weak in favor.

#### Recommendation 6.5.1. Positional therapy in infants

*The panel recommends against the routine use of positional therapy to alleviate symptoms of GERD in sleeping infants due to insufficient evidence of efficacy (head elevation) and safety concerns (lateral and prone position)*,* particularly for the associated increased risks of sudden infant death syndrome.*

AGREEMENT (scores 7–9:100%); SOR: 91.67% strong in favor; 8.33% weak in favor.

#### Recommendation 6.5.2. Positional measures in older children

*Positional measures may be considered in older children with GERD.*

AGREEMENT (scores 7–9:90.1%; scores 4–6:9.1%); SOR: 90.1% strong in favor; 9.1% weak in favor.

### Study selection

A total of 2,052 records were identified in the electronic databases: 803 PubMed, 868 from Embase, and 381 from Web of Science. Six additional records were identified through a manual search for the reference lists of relevant articles. After removing duplicates, 1,761 records remained for title and abstract screening. Of these, 63 full-text articles were assessed for eligibility, including 40 studies [65, 70–108] in the final review. The Prisma 2020 flow diagram can be found in Additional File 1. A list of excluded studies and reasons for exclusion is provided in Additional File 1.

### Study characteristics

The main characteristics of the included studies are shown in Additional File 2. The included studies were published between 1983 and 2024. Most clinical trials (32 out of 39) were conducted in a single country, while 7 trials were multinational.

Of the 40 included studies, 39 were randomized controlled trials (RCTs), of which 15 employed a crossover design. One study [82] was a systematic review of RCTs. The included studies primarily involved full-term infants and, to a lesser extent, preterm or very low birth weight infants [77–79, 81]. Most studies focused on otherwise healthy infants with frequent regurgitation, few studies included infants with GERD [71, 85, 87, 94], with diagnosis based on clinical criteria or pH monitoring.

The sample sizes of the 39 RCTs ranged from 5 to 960 participants, with a median of 53 infants and an interquartile range of 70. Seventeen studies (43.6%) enrolled fewer than 50 infants, 14 (35.9%) enrolled between 50 and 100, and 8 (20.5%) enrolled more than 100 infants.

The included studies assessed a wide range of non-pharmacological interventions for managing GER or GERD in infants. Most trials (*n* = 25) evaluated dietary modifications, such as thickened formulas (e.g., with rice starch, carob flour, or locust bean gum), hydrolyzed or soy-based formulas, and formulas enriched with prebiotics or postbiotics. Probiotics were investigated in three studies [71, 82, 86], most commonly testing *Lactobacillus reuteri* or *Bifidobacterium animalis*. Six studies [81, 88, 95–97, 101] assessed positioning therapy, including prone, left lateral, and head-elevated positions. Four trials [70, 74, 80, 89] evaluated alginate or alginate-based reflux suppressants. Two studies [87, 94] examined manual therapies, including massage therapy.

The comparators used in the studies were: standard formulas, different thickening agents or protein compositions, placebo or no treatment, alternative body positions (e.g., prone vs. supine or lateral), sham massage or massage without active ingredients.

### Summary of evidence

The initial approach to manage infant regurgitation effectively relies on parental reassurance and education regarding positioning and feeding, as well as avoiding overfeeding, as recommended by NASPGHAN-ESPGHAN and the NICE guidelines on GER [1, 2].

Most studies evaluated different non-pharmacological interventions in infants, including dietary strategies, particularly thickened infant formulas and protein hydrolysed formulas; probiotics; medical devices (mainly alginates); infant positioning; and physical treatments, such as massage therapy and osteopathy.

### Diet strategies and thickened feeding

Although there are no studies evaluating different breastfeeding modalities and data comparing human milk with formulas in infants with GERD and GERD are very limited, breastfeeding should always be supported and promoted for its many beneficial properties. In formula-fed infants, parents should receive advice on how to adapt feeding practices for GERD in infants, such as feeding volume and frequency, milk thickening, or the use of special formulas [2].

The search identified eighteen studies on thickened feedings [65, 72, 73, 75, 76, 83, 84, 90–93, 99, 100, 104–108], one on soy-based formula with added soy fiber [98], two on extensive protein hydrolyzed protein formula [78, 102], three on partial protein hydrolyzed formulas [85, 103, 105] and two on maternal milk fortifiers [77, 79]. No studies on reduced feeding volumes, more frequent feedings, or amino-acid-based formulas met our inclusion criteria. Moreover, no RCT was found to assess different feeding practices or diets beyond infancy.

#### Thickened formulas

The principle of using anti-regurgitation (AR) formula in formula-fed infants with GER is based on incorporating a thickening agent into a standard infant formula to increase its viscosity, a quantitative rheological measurement of frictional resistance to shear in a fluid. This process should visibly decrease both the frequency and volume of regurgitation episodes and could improve growth in formula-fed infants [109].

Eleven RCTs have compared the thickened formula with the standard formula [75, 83, 84, 90–93, 104, 106–108], two with a formula enriched with rice flour [73, 99], two with a different formula enriched with prebiotics and probiotics [72, 100], one with a partially hydrolyzed thickened formula [105], one with parental counseling and lifestyle modifications [65], and another with infant positioning [76]. In two studies [72, 100], the thickened formula was enriched with locust bean gum and a specific combination of prebiotics and postbiotics. The study populations of all RCTs consisted of infants with uncomplicated GER and functional regurgitation.

Most studies have evaluated clinical benefits by measuring reductions in the frequency of regurgitation, vomiting episodes, and GER clinical scores. In few trials, the volume of GER has also been assessed. Six studies considered symptoms alongside esophageal pH and/or pH-impedance monitoring [73, 93, 99, 104, 107, 108]. In three studies, gastric emptying was assessed through scintigraphy or ultrasonographic emptying studies [75, 90, 92].

A significant decrease in the number of regurgitation and vomiting episodes using various thickening agents in AR formulas has been reported, showing a mean reduction in the episodes of regurgitation from 5.4 per day to 2.5 per day over one to four weeks. In subgroups of patients, improvement in esophageal pH and/or impedance parameters has also been observed but esophageal investigations have been rarely performed [109].

Only a few studies have assessed the impact of thickened formula on other symptoms of GER. One study [106] noted improvements in choking, gagging, coughing episodes, and sleep quality. In three studies [72, 73, 100] other gastrointestinal clinical symptoms and scores were reduced. One RCT [65] compared the efficacy of a rice starch thickened formula (reported as 14.3 g/100 ml) with a standard formula and with treatment with alginate plus simethicone. All three groups showed a significant reduction in regurgitation after two months of intervention, with a slightly better score in the alginate group.

The main thickening agents utilized were locust bean gum, cornstarch, and rice starch. Two studies noted an apparent superiority of adding locust bean gum and cornstarch compared to rice flour when added to standard infant formula [73, 99]. Only one study assessed the effect of a soy-fiber-thickened formula in 66 infants showing a slight reduction in the number of episodes of regurgitation/vomiting per day (-0.4 per day) after four weeks of intervention compared to 89 infants who were fed a standard formula [98].

No definitive superiority of one thickening agent over the others can be established, due to variations in the formula composition, comparisons, study designs, and outcome measures. No significant adverse events associated with the thickened formulas have been noted in term infants [109]. By contrast, thickened formulas are not recommended in preterm infants due to reported cases of necrotizing enterocolitis [110, 111]; metabolic acidosis, hypokalaemia, and increased stool frequency have been reported in six vomiting infants born preterm with a low birth weight fed with a locust bean gum thickened formula (0.2–0.5 g/100 mL) [112].

#### Home-thickened formula

Our research found no RCT comparing home-thickening agents in standard formulas to other interventions. It is worth noting that the effects of home-thickened feeding on viscosity, gastric emptying, calorie content, and overall, GER symptoms may vary compared to those of AR formulas. The risk of over-thickening the formula and using unsuitable thickening agents can result in undesirable symptoms and unbalanced nutritional intake. Therefore, we do not recommend home-thickening the standard formula using cereal flour due to limited data and potential risks.

#### Fortification of maternal milk in preterm and term infants

Our research has identified two studies on maternal milk fortification [77, 79] and its effects on GER. The first study assessed the impact of thickening human milk with precooked starch on GER in preterm infants and found no significant difference between the two groups. The second study compared the effects of a human milk fortifier derived from donkey milk on GER with those of a bovine milk fortifier in very low birth weight infants, indicating a potential benefit for the donkey fortifier group. No studies have been found regarding the thickening of breast milk in term infants.

### Hydrolyzed cow’s milk protein formulas

Hydrolyzed formulas may reduce regurgitation, vomiting, and crying, which are assumed to be GER-related because of possible associated CMA or delayed gastric emptying. However, RCTs are very limited, often recruiting small populations and heterogeneity in terms of formula composition (source of protein, degree of hydrolysis, thickening agent, additional components), diagnostic workup, outcome measures, rate of cow’s milk challenge, and follow-up evaluation [113].

In a group of 72 formula-fed infants presenting multiple symptoms, including more than five episodes per day of regurgitation or vomiting, both tthickened and non-thickened casein eHF significantly reduced regurgitation with a higher reduction of episodes of regurgitation in the group fed the thickened formula and with a negative cow’s milk challenge [102].

GERD and CMA can exhibit similar symptoms in infants [113]. According to the ESPGHAN-NASPGHAN guidelines on pediatric GER [2], along with the ESPGHAN position paper on the diagnosis, management, and prevention of CMA [114], a cow’s milk elimination diet should be evaluated for infants who do not show improvement with conservative GER treatments before investigations or pharmacological interventions. A 2 to 4-week trial of a cow’s milk elimination diet for breastfeeding mothers or an extensively protein hydrolyzed formula for formula-fed infants should be considered. An oral food challenge should be arranged to confirm or rule out the diagnosis of CMA.

In recent years, to increase awareness of cow’s milk-related symptoms in infants, a specific questionnaire (CoMiSS™) has been proposed and tested in many studies to help clinicians identifying infants presenting with various symptoms (including regurgitation) who may benefit from a cow’s milk elimination diet and an extensively eHF [115].

pHF are not indicated to treat CMA, and their efficacy in infants with regurgitation and crying is limited, possibly due to the combined presence of thickening agents, pre/probiotics, beta-palmitate, and reduced lactose content. In 12 infants with persistent regurgitation not improved with thickened or hydrolyzed formula, a significant decrease of regurgitation and crying was found with a double thickened (bean gum and processed tapioca starch) whey-based pHF compared to a (single) thickened casein-predominant formula [103]. In another double-blind, randomized, cross-over trial including 115 infants, the mean number and volume of regurgitations significantly decreased with two thickened formulas, with statistically better results for the whey-based pHF [105]. Another double-blind RCT by Indrio et al. [85] evaluated a whey-based pHF with starch and Lactobacillus (L.) reuteri (DSM 17938) on gastric emptying and regurgitation in 80 infants, 72 of whom completed the study. The test group showed a significant improvement in ultrasound assessment of gastric emptying rate and a reduction in daily regurgitations from 7.4 to 2.6, compared to controls.

**Table:** 

**PRACTICE POINTS CONCERNING INFANT FEEDING:**
1. In all infants with GER and GERD, physicians should promote and support breast-feeding.
2. Overfeeding is often a cause of infant regurgitation, and the daily intake of formula or infant weight gain should be considered.
3. Thickened formulas reduce episodes of regurgitation and vomiting and may offer a safe and beneficial effect for formula-fed term infants experiencing persistent and disabling/troublesome regurgitation who do not respond to parental reassurance, education, and conservative treatment. There is no clear clinical evidence that one thickening agent is better than another. Regular monitoring of symptom improvement and the need for protracted intervention is important to avoid unnecessary thickened feeding.
4. Thickened formulas should not be used in preterm newborns because of limited data and potential serious adverse events.
5. Home-made thickened formulas carry potential risks of worsening symptoms, increasing calories and unbalanced nutritional intake.
6. There is limited data about the potential benefits and safety of starch or fortifiers in maternal milk in treating GER in preterm and term infants.
7. In infants with persistent, frequent and troublesome regurgitations and additional symptoms suggestive of CMA, there is limited evidence of symptom improvement on cow’s milk protein elimination diet and eHF, particularly in infants scoring ≥ 10 using CoMiSS tool.
8. To diagnose CMA, symptoms (and CoMiSS score) must improve significantly on diet and relapse on cow’s milk reintroduction and challenge.

### Probiotics

Our search identified six studies evaluating the effect on regurgitation of different probiotic strains compared to placebo or no intervention, and one study on a thickened partial hydrolysed formula enriched with probiotic (L. reuteri DSM 17938) compared to a control formula without probiotics (as reported in the previous paragraphs) [85].In 2008, Indrio et al. investigated the effect of a probiotic (L. reuteri ATCC 55730, at a daily dose of 1 × 10(8) colony forming units (CFU)) or placebo for 30 days on feeding tolerance and gastrointestinal motility, in 20 healthy formula-fed preterm infants. Infants receiving probiotics showed a significant decrease in regurgitation and mean daily crying time, and increased gastric emptying rate, compared to infants in the placebo group [116].

In a double-blind RCT, Indrio et al. explored the effects of L. reuteri DSM 17,938 on regurgitation and gastric emptying. Forty-two infants participated, and 34 completed the study; the probiotic group demonstrated significant improvements, including a reduction in median fasting antral area and gastric emptying rate, as well as a reduction in median regurgitation episodes per day compared to the placebo group [86].In a double-blind RCT, 40 breastfed, full-term infants were assigned to receive (L. reuteri DSM 17938 (5 drops daily, 10(8)CFU) starting in the first 3 days of life and supplemented for 4 weeks) or placebo. Treated infants showed significantly fewer daily episodes of regurgitation at the end of treatment, compared to the placebo group [117].The preventive effect of the same strain of L. reuteri (DSM 17938) was confirmed in a large prospective RCT that enrolled 589 infants in whom supplementation began within the first week of life and continued for 90 days. At 3 months of age, the mean daily number of regurgitation episodes, as well as the mean duration of crying time, were significantly lower in the probiotic group compared to the placebo group (2.9 vs. 4.6 and 38 vs. 71 min, respectively) [118].In an open-label RCT, Baldassarre et al. explored the effect of Bifidobacterium animalis subsp. lactis BB-12 (six drops daily, 1 × 10(9) CFU) in 960 formula-fed infants with persistent regurgitation. The BB-12 group experienced a significant reduction in the I-GERQ-R compared to an increase in the control group (which received no probiotic), after two months of treatment (from 25.8% to 14.7% vs. from 31.7% to 50.7%) [71].

Recently, a single-blind RCT in 90 full-term infants, aged 1 to 4 months, showed a greater reduction in daily regurgitation episodes in infants treated with L. reuteri NCIMB 30,351 compared to the placebo group (-4.8 vs. -3) [119].

**Table:** 

**PRACTICE POINT**
There is currently no evidence of the efficacy of any specific probiotic strain in treating infant regurgitation; the possible prophylactic effect of L. reuteri on the development of troublesome regurgitation requires further well-designed, double-blind RCTs, with large populations and analysis of potential confounding variables (e.g., formula intake volume, neonatal complications, intercurrent infections, and concomitant medications). Currently, data are limited, and intervention studies are highly heterogeneous.

### Medical devices

#### Alginates

Alginates, derived from alginic acid in seaweed, form a gel that acts as a physical barrier in the proximal stomach. When combined with sodium or potassium bicarbonate, they also produce a carbon dioxide foam raft. Infant alginate formulations containing magnesium alginate without bicarbonate increase gastric viscosity but do not create a floating raft. Our search identified six RCTs of alginate use versus placebo, thickened formula, or PPIs in the treatment of GER/GERD.

The population included infants with functional regurgitation and GER from 4 studies [65, 70, 80, 89].

Miller et al. [89] found that, after two weeks of alginate treatment, the number of vomiting and regurgitation episodes in 24 h was significantly lower than at baseline (*p* = 0.009). However, the frequency and severity of vomiting and regurgitation did not differ statistically. In a double-blind trial, Del Buono et al. [80] analyzed the effects of sodium and magnesium alginate plus mannitol compared to placebo in infants with GER, who underwent esophageal MII-pH monitoring. They found no significant difference in the number of reflux events, acid reflux events, or total acid clearance per hour between the two groups. However, they reported a reduced average reflux height in the esophagus in the alginate group (*p* < 0.001).

Two more recent RCTs [65, 70] have shown a significant improvement in GER symptoms, as measured by I-GERQ-R during alginate treatment, compared to athickened formula and reassurance with lifestyle changes in one study [65], and with similar clinical efficacy to thickened formulas, after two weeks of treatment, in formula-fed infants in a multicenter cross-over RCT [70]. The beneficial effect of a formulation containing magnesium alginate in reducing GER symptoms was also found in a subgroup of exclusively breast-fed infants with frequent regurgitation [70].

In a prospective study enrolling 43 infants with ppersisting GER symptoms not responsive to behaviour and dietetic modifications, the following MII parameters significantly decreased during the 24-hour alginate administration compared to the previous 24-hour baseline recording: the median number of all MII reflux episodes (*p* < 0.001), acid, non-acid, proximal GER episodes, and bolus exposure index. Noteworthy, crying-fussiness, cough and regurgitation episodes significantly improved during the MII-pH period with alginate administration (*p* = 0.00012; *p* = 0.005 and *p* = 0.04, respectively) [120].

In conclusion, RCTs assessing the effect of alginate treatment in infants are limited, including small sample size and varying formulations. The reduction of symptoms of GER appears comparable to the one obtained with the thickened formulas.

In our review, only two studies [63, 74] included older children (aged 2–84 months and 12 months-12 years, respectively). The first study [74] examined symptoms and performed esophageal pH monitoring on a small cohort of patients (10 in the alginate group and 10 in the control group) with no esophagitis, showing that alginic acid and sodium bicarbonate can effectively manage GER in children, leading to symptom improvement and better esophageal pH monitoring results compared to placebo. The second RCT [63] found that alginate administered for 8 weeks was as effective as PPI (lansoprazole) in reducing symptom scores and esophageal acid exposure in a small group of patients with moderate esophagitis (10 patients in each group). Notably, the combination of alginate and PPI in a third group of 12 patients proved to be more effective than either treatment alone in alleviating symptoms and improving the reflux index, as measured by 24-hour pH monitoring.

However, the small population size and the limited data on this age group should be considered when drawing extensive conclusions.

#### Safety concern

Alginate formulations containing sodium may pose a risk of hypernatremia, particularly in subjects with renal impairment, congestive heart failure, preterm infants, or children experiencing diarrhea and vomiting [52]. Caution is also advised when using alginates that contain aluminum (aluminum-free formulations are now available) and in children with vomiting, diarrhea, or those at risk of intestinal obstruction. In infants receiving thickened feeds, the concurrent use of alginates may potentially increase the risk of intestinal obstruction [121]. According to NICE guidelines, thickened formulas should be discontinued if alginates are introduced [52, 122].

**Table:** 

**PRACTICE POINTS**
• There is limited evidence that alginate formulations may improve GERD symptoms and selected MII-pH parameters in infants and children.
• Alginate formulations may offer clinical benefit in selected individuals with troublesome, persistent symptoms of GER that **c**annot be adequately managed through conservative measures. A short course in alginate treatment, clinical reassessment after 1 to 2 weeks and regular monitoring with attempts at discontinuation is essential to avoid unnecessary and protracted use.
• Physicians should be aware that infants at risk of intestinal obstruction, renal impairment or on thickened formulas may have an increased risk of adverse effects and should be excluded from treatment or monitored more carefully with an adapted alginate dose and formulation.

### Esophageal mucosal protection

With advancements in understanding GERD pathophysiology and the relevance of defensive mechanisms and mucosal integrity, a novel therapeutic approach – esophageal mucosal protection – has recently been introduced. However, the available data on the safety and efficacy of mucosal protective drugs, such as sucralfate (a complex of sucrose sulfate and aluminum hydroxide), and formulations of mucopolysaccharides (such as sodium hyaluronate and sodium chondroitin sulfate) in the treatment of GERD are currently scant. Promising results have been observed in adults [123, 124], whilst, to date, we found only one pediatric retrospective study. The efficacy and safety of a formulation containing sodium hyaluronate and sodium chondroitin sulfate was evaluated in 25 adolescents with GERD-related symptoms showing significant symptom improvement following 3 weeks of treatment [125]. However, these findings require confirmation through a large, randomized clinical trial before this medical device can be considered a therapeutic option for managing GERD in children.

### Infant positioning

Postural treatment has traditionally been used to manage symptomatic GERD. In adults, elevating the head of the bed modestly reduces the time spent in a supine position with acid exposure compared to lying flat [126, 127]. Consequently, various measures have been attempted in infancy, including infant seats and elevating the head of the cot. However, sitting upright may lead to slumping in young infants who lack adequate truncal control, potentially increasing intra-abdominal pressure and reflux. Six [76, 81, 95–97, 126] randomized controlled trials were included in the review, which reported data on several positions (supine, prone, left lateral, right lateral, all horizontal, or with a 30° head elevation, infant seat at a 60° inclination, postprandial upright position for 90 min) in infants. Different positions and gastric emptying in infants were evaluated and compared by esophageal pH-monitoring and technetium 99 m milk scintigraphy. The studies revealed that the prone position significantly reduced reflux in infants compared to both the supine and right lateral positions. Moreover, the left lateral position was more effective than the right lateral and supine position to reduce GER episodes [126]. The 60 min postprandial period was evaluated by manometry, MII-pH, 13 C-octanoate breath tests and gastric volume scintigraphy in 10 term infants with GERD and in 10 controls, randomly assigned to right or left lateral position. Infants with GERD had more TLESR and GER episodes, and more pronounced proximal gastric distension and gastric emptying in the right compared to the left lateral position in the first postprandial hour. No statistical differences were found between the left lateral and prone positions [126]. Additionally, the 60° infant seat showed no effectiveness in reducing reflux compared to the horizontal prone position [97], and the prone-elevated positioning in a harness, in infants under six months old [96]. Moreover, no significant differences were observed with a 30° head elevation: for infants younger than six months with abnormal reflux, flat-prone positioning was as effective as head-elevated prone positioning [95]. Furthermore, maintaining a postprandial upright position for 90 min significantly reduced the frequency of regurgitation in infants; however, cereal-thickened formula proved to be even more effective in minimizing regurgitation episodes than positional treatment [76].

Based on the above, both prone and left lateral positions can effectively decrease episodes of reflux, whereas the benefit of positional treatment for GER-related signs or symptoms is uncertain. Nevertheless, prone positioning in infants is contraindicated due to the increased risk of Sudden Infant Death Syndrome (SIDS), and side sleeping cannot be recommended due to insufficient safety data. After evaluating the risk/benefit ratio of positional interventions, the supine position is the only recommended position for safe sleep-in infants. Moreover, elevating an infant’s head during sleep in a supine position was not effective or validated as usual practice; additionally, it may lead to the infant rolling to the foot of the crib, potentially compromising respiration.

We did not find RCTs assessing the effectiveness of positioning interventions in older children.

**Table:** 

**PRACTICE POINTS**
1. Prone and left lateral positions reduce episodes of reflux by placing the Lower Esophageal Sphincter (LES) less prone to reflux of gastric contents. However, evidence of symptom improvement is limited, and prone and lateral positions are contraindicated in infants, particularly in the first few months of life, due to the increased risk of SIDS.
2. Positional treatment, avoiding lying down soon after meals and elevating the head of the bed when sleeping, may be considered in older infants and children with GERD.

### Physical treatments

#### Massage therapy

The search identified one RCT which assessed the effects of massage therapy on infants with GERD. Neu et al. [94] randomly assigned 36 infants to either massage or sham therapy (rocking and holding). Both groups showed improvements in symptoms according to I-GERQ-R scores, with no significant difference after six weeks. Limitations included a small sample size and a short intervention duration.

Another RCT [87] evaluated the efficacy of abdominal massage with vs. without mastic gum oil (every 12 h for 2 weeks), in addition to omeprazole treatment in 90 infants with GERD. The authors reported a significant decrease in both mean composite and individual symptoms score in both groups, without statistical difference between the two interventions. In the first and fourth weeks of follow-up, symptom scores increased but did not reach baseline levels, possibly due to the short duration of treatment. Therefore, larger sample size studies and longer treatment duration are required before a clear conclusion can be drawn regarding the effect of massage therapy on infants with GERD.

#### Osteopathy

Osteopathy is a complementary medical practice that involves manual manipulation to treat joint and myofascial system functions. Applications of osteopathy have been assessed for visceral disorders, such as functional gastrointestinal disorders and GERD in adults, yielding some positive results [128]. Two RCTs in adult patients showed a significant reduction of GERD symptoms with the osteopathic treatment [129, 130].

Currently, there is no RCT available on the effectiveness of osteopathic treatment for GER or GERD in infants and children. Consequently, we do not advocate for the use of osteopathic manipulations or techniques for these conditions in the pediatric population due to the lack of supporting evidence.

**Table:** 

PRACTICE POINT
Studies assessing the effect and safety of massage and osteopathy in infants and children with GER are currently very limited and heterogeneous in terms of population, intervention and outcome measures.

### PICO question 7: What is the indication and the effectiveness of different surgical/endoscopic treatment options for GERD?

#### Recommendation 7.1. Surgical treatment

*We recommend considering surgical treatment for GERD in pediatric patients with clear*,* objectively defined evidence of severe and refractory GERD and failure of optimized medical management.*

AGREEMENT (scores 7–9:100%); SOR: 91.67% strong in favor; 8.33% weak in favor.

#### Recommendation 7.2. Preoperative workup

*We recommend a complete preoperative workup*,* including upper gastrointestinal endoscopy (with biopsies)*,* and esophageal pH monitoring (preferably with impedance) to objectively document GERD and assist in appropriate patient selection before considering antireflux surgery.*

AGREEMENT (scores 7–9:100%); SOR: 91.67% strong in favor; 8.33% weak in favor.

#### Recommendation 7.3. Indications for antireflux surgery

*We recommend considering antireflux surgery specifically in patients with*:

- *Persistent severe symptoms with objective evidence of GERD*,* or documented GERD-related complications such as persistent severe peptic esophagitis or life‐threatening complications (e.g.*,* recurrent aspiration or cardiorespiratory failure)*,* despite optimized PPIs therapy*.
- *Predisposing anatomic anomalies (e.g.*,* large hiatal hernia) or underlying conditions (e.g.*,* neurologic impairment) that increase the risk of GERD-related morbidity.*

AGREEMENT (scores 7–9:100%); SOR: 88.33% strong in favor; 16.67% weak in favor.

#### Recommendation 7.4. The surgical approach

*We suggest considering laparoscopic fundoplication as the preferred technique*,* given its association with shorter hospital stays and favorable perioperative outcomes compared with open surgery. However*,* the surgical approach (laparoscopic vs. open) should be individualized*,* taking into account the patient’s clinical status*,* caregiver preferences*,* and available resources.*

AGREEMENT (scores 7–9:100%); SOR: 58.33% strong in favor; 41.67% weak in favor.

#### Recommendation 7.5. Fundoplication and gastrostomy

*Routine prophylactic fundoplication at the time of gastrostomy placement is not recommended in children with neurological disabilities*,* as evidence is inconsistent with a reduction in reflux-related hospital admissions*,* while showing a high risk of postoperative complications in these patients.*

AGREEMENT (scores 7–9:100%); SOR: 66.67% strong in favor; 33.33% weak in favor.

#### Recommendation 7.6. Antireflux surgery in esophageal atresia

*We recommend that antireflux surgery in patients with esophageal atresia be considered only after a multidisciplinary evaluation and extensive preoperative workup*,* as these patients are at high risk for postoperative complications. We recommend against routine fundoplication for refractory/recurrent anastomotic stricture.*

AGREEMENT (scores 7–9:100%); SOR: 83.33% strong in favor; 16.67% weak in favor.

#### Recommendation 7.7. Decision-making process for antireflux surgery

*We recommend considering antireflux surgery only after carefully evaluating the benefits*,* risks*,* and alternatives*,* through a shared decision-making process between clinicians and the patient/caregivers.*

AGREEMENT (scores 7–9:100%); SOR: 75% strong in favor; 25% weak in favor.

#### Recommendation 7.8. Antireflux endoscopic procedures

*We do not recommend endoscopic procedures for GERD treatment in children due to the current lack of data.*

AGREEMENT (scores 7–9:100%); SOR: 75% strong in favor; 25% weak in favor.

### Study selection

The literature search retrieved 2,022 records (684 from PubMed, 1,061 from Embase and 277 from Web of Science). After removing duplicates, 1,713 records were screened by title and abstract, and 1,683 were excluded. A total of 30 full-text reports were assessed for eligibility, of which 10 reports [131–140] were included in the final analysis. The PRISMA flow diagram, the list of excluded studies, and the reasons for exclusion, are provided in Additional File 1.

### Study characteristics

The included studies were published between 2010 and 2023 and evaluated various surgical procedures for antireflux surgery in children. The comparisons were between laparoscopic Nissen fundoplication vs. open Nissen Fundoplication [131, 132, 134, 137–139] or laparoscopic Thal fundoplication [135, 136, 140] or vs. Hill-Snow technique [133].

Sample sizes ranged from 39 to 175 patients (mean: 77 patients). The average age of participants ranged from 6 months to 5.2 years. Most children had significant comorbidities.

Knatten et al. [134] reported that 52% of children were neurologically impaired, having cerebral palsy, genetic syndromes, central nervous system disorders, or brain injury due to perinatal asphyxia. Higher rates of neurological impairment were reported by McHoney et al. [137] (77%) and Kubiak et al. [135] (69%). In all included studies, the main indication for surgery was the persistence of GERD symptoms or complications unresponsive to optimal medical therapy. The characteristics of the included studies are detailed in Additional File 2.

### Summary of evidence

They included RCTs compared different surgical antireflux techniques; no RCT compared medical versus surgical approach. The studies often lack comprehensive documentation of GERD diagnoses and previous medical treatments that complicate the evaluation of both indications and outcomes of antireflux surgery. Indications for pediatric antireflux surgery remain poorly defined, leading to considerable variability among centers in both patient selection and surgical approach [141].

Current pediatric guidelines emphasize that only patients with clearly proven GERD should be considered for surgery, but they also emphasize that obtaining objective and definitive proof of GERD in children remains difficult to achieve, as there is no standard diagnostic tool [2].

In clinical practice, children considered for antireflux surgery typically experience persistent troublesome symptoms despite optimized medical therapy, suffer from complications such as reflux-related pulmonary aspiration or peptic esophagitis, or present with anatomical abnormalities like a large hiatal hernia. Selection for surgery is based on a combination of reflux-related symptoms, underlying comorbidities (e.g., neurological impairment or esophageal atresia), and a preoperative diagnostic workup, which typically includes upper gastrointestinal endoscopy, esophageal pH monitoring or pH impedance testing, and contrast imaging studies. However, many indications for surgery are based on individual experience [1, 2].

Fundoplication is the most common surgical procedure for pediatric GERD. In this procedure, the gastric fundus is wrapped around the distal esophagus to strengthen the LES by increasing its basal tone, reducing TLESR, and correcting anatomical defects, such as hiatal hernia. Different techniques include total fundoplication (a 360° wrap, the Nissen procedure) and partial fundoplication (a 270° posterior wrap Toupet or a 180° anterior wrap Dor procedure) [142].

Data from exiting literature suggests that fundoplication is effective in preventing reflux-related complications and in enhancing quality of life in well-selected patients. However, it permanently alters the gastroesophageal anatomy and may lead to a range of complications [143]. Inherent complications of antireflux surgery may be directly caused by the wrap, leading to an antegrade obstruction that causes dysphagia and/or a retrograde obstruction that prevents the venting of gas from the stomach and vomiting, potentially resulting in gas-bloat syndrome [136, 144–147]. The likelihood of experiencing dysphagia post-fundoplication rises considerably for patients with esophageal dysmotility, primarily due to insufficient peristaltic vigor to overcome the obstructive effect of the fundoplication [148]. Evidence from initial experiences suggests that high-resolution impedance manometry, when analyzed using the pressure-flow technique, provides a strong predictive basis for postoperative new-onset dysphagia [144, 149].

Other complications, such as wrap failure due to a loose or disrupted wrap, hiatal herniation, changes in gastric sensorimotor function leading to visceral hypersensitivity and retching, and rapid gastric emptying causing dumping syndrome, can also affect an otherwise successful GERD treatment [150–157].

Fundoplication procedures can be performed using either open or laparoscopic approaches [142]. Currently, laparoscopic fundoplication is widely considered the technique of choice by pediatric surgeons, as follow-up studies have shown that the laparoscopic approach is associated with superior outcomes (hospital stay, costs, infection rates, surgical complications, and unplanned readmissions) compared with the open procedure [155, 158, 159]. However, findings from three pediatric RCTs published in six reports showed a higher recurrence rate of GERD in laparoscopic surgery than in open surgery [131, 132, 134, 137–139]. Data comparing the outcomes of partial versus complete fundoplication in children is limited. Evidence from two RCTs (four reports, three of which are from the same group) suggests that there is no significant difference in symptom control between total and partial fundoplication, although the latter was associated with fewer postoperative side effects, particularly postoperative dysphagia [133, 135, 136, 140].

Underlying conditions, notably neurological impairment or prior esophageal atresia repair, are associated with higher post-surgical complication rates. Unfortunately, these conditions are those identified as high-risk for severe GERD and represent common indications for pediatric fundoplication [160–162]. However, there is a significant gap in research that assesses GERD in this population prior to surgical intervention and its correlation with outcomes after the procedure [163]. Neurologically impaired children undergoing gastrostomy are considered at high risk for developing or worsening GERD, which has historically led to the use of “prophylactic” antireflux surgery. However, fundoplication in this population carries a high postoperative morbidity rate (up to 50%), with a mortality rate of 1% to 3% [164–166]. Moreover, studies have shown that reflux-related hospitalizations in infants receiving both fundoplication and gastrostomy are similar to those in patients with gastrostomy alone, and MII-pH did not find a significant aggravation of GERD following gastrostomy [167–169]. Furthermore, evidence failed to show a consistent benefit of fundoplication for the treatment of aspiration pneumonia in neurologically impaired children [170–172]. Although data are limited and no clear superiority has been demonstrated compared to fundoplication, post-pyloric (jejunal) feeding may be considered as an alternative approach to manage GERD-related complications, particularly in children with neurological impairment who already had a gastrostomy in place and are experiencing refractory vomiting, retching, or bloating [162, 172, 173].

Up to 45% of patients with esophageal atresia (EA) undergo fundoplication, and nearly all long-gap patients are treated with this procedure, despite the lack of well-defined indications, as no controlled trials have assessed the role of surgical management of GERD in this population [161, 174, 175]. Fundoplication is performed in up to 45% of EA patients and almost all long-gap EA patients even if the indications for fundoplication are not clearly defined [161, 174, 175]. However, the poor outcomes observed with fundoplication suggest a need for re-evaluation of the frequent use of antireflux surgery in this specific population [176, 177]. Fundoplication may create an outflow obstruction that could exacerbate the dysphagia related to esophageal dysmotility or even result in new-onset dysphagia, which occurs in up to 50% of these children. Moreover, recent studies have raised questions about whether antireflux surgery can effectively prevent esophageal complications in these patients. Likewise, although recurrent or refractory anastomotic stricture is a key factor driving the use of systematic PPIs therapy and antireflux surgery in patients with EA, recent studies have shown that GERD treatment is unable to prevent anastomotic stricture formation, thereby questioning the true pathophysiological role of GERD in these strictures.

In children, it is currently not possible to establish clear recommendations for endoscopic treatment of GERD, because of the limited data available.

**Table:** 

**PRACTICE POINTS**
• Antireflux surgery should be considered for pediatric patients with confirmed severe refractory GERD or serious complications that do not respond to optimal medical treatment. However, potential post-surgical issues, such as dysphagia and wrap failure, should be considered before intervention.
• Surgery requires an extensive preoperative assessment to evaluate GERD severity and guide surgical planning, involving clinicians, patients, and caregivers through a shared decision-making process considering the benefits, risks, and alternatives.
• The choice of surgical techniques should depend on the individual patient’s characteristics, underlying comorbidities, and the surgeon’s experience, ensuring that the potential benefits outweigh the inherent risks.

## Key Question 8: What is the prognosis of GER and GERD in infants, children, and adolescents, and what are the prognostic factors?

### Study selection

For Key Question 8, search strategies (detailed in Additional File 1) were applied to PubMed, Embase, and Web of Science. In the identification phase, 2,104 articles were retrieved. After removing 189 duplicates, 1,915 records were screened. During the initial screening phase, 1,894 articles were excluded. Of the 20 articles initially selected, only four [178–181] satisfied our inclusion criteria and their main characteristics are summarized in Additional File 2. Manual retrieval from references of included studies provided additional information about the natural history and prognostic factors of GERD.

### Summary of evidence

There is a paucity of longitudinal studies that report the natural history of pediatric GER and GERD and explore the associated risk factors. Besides, there is heterogeneity in population in terms of age and comorbidity, diagnosis of GERD, outcome measures and follow-up data, limiting general conclusions and recommendations.

A number of studies showed that GER expressed as regurgitation occurs daily in at least 25% of healthy infants during the first 4 months of life, declining progressively and resolving in 95% of them at one year of age [2, 182, 183]. In children older than 18 months, the prevalence of GERD symptoms varies across studies, ranging from 0% to 38% of the population recruited, with > 10% of patients complaining weekly symptoms [183].

Martin et al. [184] reported that frequent regurgitation (> 90 days) during infancy, maternal GERD and smoking (before pregnancy or in the first year of baby life) significantly increase the risk of childhood GER symptoms, as reported by 693 children at 9 years of age (4.6 (95% CI: 1.5–13.8) for heartburn; 2.7 (95% CI: 1.4–5.5) for vomiting, and 4.7 (95% CI: 1.6–14.0) for acid regurgitation.

In a group of 225 adults with symptoms of GERD, 141 (63%) individuals recalled at least one childhood symptom (mostly beginning in infancy), compared with 54/154 (35%) adults not complaining about GERD (< 0.001) [185].

In 2004 El-Serag [186] found that 18/80 (23%) children (with no comorbidity) who had reflux erosive esophagitis at diagnosis (mean age of 5 years), at mean 15-yr follow-up had weekly heartburn and/or acid regurgitation, while 64/80 (80%) children had at least monthly symptoms, and other three patients did not report symptoms but were taken acid suppressant drugs. Overall, in this population 24/80 (30%) patients were taking either H2RA or PPIs, and 19 patients had undergone fundoplication. In the small group who underwent follow-up upper GI endoscopy, there were no significant differences between those without or with GERD symptoms as for demographic data, age of diagnosis, receipt of fundoplication, or current GERD treatment. At upper GI endoscopy, three patients had erosive esophagitis.

In 2007, the same author conducted a nested case-control telephone interview to examine the presence of GERD at a mean age of 18 years [187]. Heartburn or regurgitation was reported weekly in 52/113 (46%) participants with GERD esophagitis (diagnosed at a mean age of 10 years, with no associated comorbidity), 94% of whom were taking antireflux medications (PPIs, H2RAs, or antacids). Among the 33 controls, without childhood GERD, 30% of participants had weekly GERD symptoms. GERD was significantly associated with gender (female more than male) and, in female, with oral contraceptive pills. Other factors (i.e. weight, height, nonsteroidal anti-inflammatory drug use, race, family history of GERD, education level, employment status, tobacco smoking, alcohol, or coffee drinking) were not significant associated with persistent GERD [187].

In 2010 Ruigomez et al. [180] identified 1242 children or adolescents who were diagnosed GERD in the period 2000–2005, as registered in the Health Improvement Network UK primary care database. No case of esophagitis was recorded. During a mean 4-year follow-up period, 40 patients were diagnosed reflux esophagitis (incidence 10.9 per 1000 person-years). No cases of Barrett’s esophagus, esophageal stricture or esophageal ulcer were detected. GERD patients presented a double risk of asthma, pneumonia, cough or chest pain compared with children and adolescents with no diagnosis of GERD.

A review published in 2011 [188] included four longitudinal pediatric studies assessing GER or GERD evolution and pointed out that a high proportion (59–100%) of children with GERD (as for troublesome symptoms or esophagitis) continued to need anti-reflux treatment after 1–8 years.

In the 2018 systematic review on prognosis and prognostic factors of pediatric GERD, Singendonk included four studies that showed persistent GERD at follow-up (12 months to > 5 years) in 23% (weekly symptoms) to 68% (antireflux medication) of children who had esophagitis at diagnosis. In children without esophagitis at diagnosis, 1.4% developed esophagitis at follow-up (> 5 years) with no identification of prognostic factors and no cases of Barrett’s esophagus [181]. The only longitudinal follow up of a cohort of infants (*N* = 19) with reflux (histological) esophagitis who compared the effect of GERD therapy (acid suppression and prokinetic drugs) with no treatment (placebo), showed that after 12 months from diagnosis, despite symptom improvement or resolution, with parents’ global score rated “completely well” in 9, none of untreated ten infants had normal esophageal biopsy (defined as basal cell layer < 25% and papillary height < 53% of epithelial thickness) [189].

In a pediatric cohort of 166 long-term continuous (9 months − 11.2 years, mean duration 3 years) PPIs users, at least one GERD predisposing disorder was present in 79% of the patients, with the most common comorbidities represented by neuromotor impairment (in 66%) and esophageal atresia (in 14.5%). Endoscopic findings included hiatal hernia in 39% and Barrett’s esophagus in 4.8% patients [190].

A systematic review assessing Barrett’s esophagus in the pediatric population [191] included 18 studies and 130 pediatric cases (mean age 10.6 years, range 0.8–17.2 years). In 80 patients GERD was the only underlying diagnosis, whilst comorbidities were reported in 45 patients (20 children had neurological impairment, 13 esophageal atresia with/without tracheoesophageal fistula, six had chemotherapy, one previous caustic burns, one esophageal replacement with stomach, one peptic stricture, one chronic secretory diarrhea, one Fanconi anemia, one tetralogy of Fallot), and five children were considered otherwise healthy. Regarding treatment, 80 patients had surgery, 26 were on anti-reflux medical treatment and 16 had surgery combined with medical treatment [191]. According to a 2019 systematic review that included 25 studies (11 in infants and 14 in children) comprising a total population of 487,969 children, high body mass index and the use of alcohol or tobacco were associated with higher GERD symptom prevalence [183]. Family history of GERD and exposure to passive smoking were also identified as risk factors for the progression of GER symptoms in infancy in a cohort of 157 neonates [178].

In the recently reported Norway HUNT study [192], among 7620 participating adolescents, 33.2% reported reflux symptoms (3.6% as frequent symptoms). Symptoms were significantly more frequent in girls, smokers (OR 1.80; 95% CI 1.10–2.93) and obese adolescents (OR 2.50; 95% CI 1.70–3.66). A nationwide survey in Japan [193], collected data from 3,463 children with GERD, including 81 intractable GERD (defined as no symptomatic improvement of symptoms after 8 weeks of optimal medical reflux treatment and/or fundoplication, when indicated). In this population neurological impairment, esophageal atresia and congenital heart disease, overall accounted for 85% of patients. GERD is also common in selected syndromes (e.g. Down, Cornelia de Lange, Rett, CHARGE, VA(C)TERL) [2] and in patients with cystic fibrosis because of chronic cough, delayed gastric emptying, dysfunction of LES and impaired esophageal motility [194].

In 2023 Getsuwan et al. [195] aimed to develop a predictive model for refractory pediatric GERD, defined as unresponsive disease after optimal treatment with medication for > 8 weeks, by analysing clinical symptoms and MII-pH of 205 children (median (IQR) age 0.6 (0.3, 2.0) years), including 59% with motor disabilities. Multivariable analysis found a significant association between refractory GERD and motor disabilities (OR: 5.35; 95% CI: 2.06–13.91), recurrent aspiration pneumonia (OR: 2.78; 95% CI: 1.24–6.26), prematurity with neonatal onset of GERD < 40 weeks post-menstrual age (OR: 6.76; 95% CI: 1.96–23.33), and abnormal total reflux episodes according to age during MII-pH (OR: 2.78; 95% CI: 1.24–6.19), but not with the acid exposure time or symptom association analysis.

**Table:** 

**PRACTICE POINTS**
• Physicians should be aware that there are certain conditions that increase the risk, persistence, and severity of GERD in neonates, infants, children and adolescents. These predisposing conditions include preterm neonates; gastroesophageal malformations, particularly esophageal atresia and tracheoesophageal fistulas; severe neurological impairment; cystic fibrosis; selected genetic syndromes (Down, Cornelia de Lange, Rett, CHARGE, VA(C)TERL); obesity; exposure to tobacco and alcohol.
• In these at-risk individuals, clinical detection of GERD symptoms may be challenging and investigations including MII-pH and upper GI endoscopy may be indicated to identify acid reflux and associated symptoms and esophagitis, avoiding underdiagnosis and complications but also unnecessary prolonged treatment with PPIs.
• Regular assessment and monitoring of these patients based on a multidisciplinary approach is essential for an optimal care of this complex population.

## Special considerations

### Preterm newborns

GER episodes in preterm infants are common and are due to multiple factors, including a short esophagus, frequent feeding, the supine position, delayed gastric emptying, TLESR, and immaturity of esophageal motility [196–199]. In addition, GER may be increased by frequent complications of prematurity such as bronchopulmonary dysplasia, tracheomalacia, apnoea, infections, feeding intolerance, use of nasogastric tube, ventilation and xanthines [200]. Although numerous GER events occur in preterm newborns (frequency up to 2–4 events per hour, as detected by MII-pH) [196], these episodes are mostly weakly acidic because of the frequent milk feedings that buffer the gastric content. Nevertheless, empirical acid suppressive treatment is often prescribed in neonatal (intensive care) units when newborns present feeding intolerance or aversion, poor weight gain, regurgitation, apnoea, desaturation, stridor, or postprandial bradycardia, irritability, inconsolable crying. These symptoms and gastric emptying usually improve spontaneously with the growth and maturation of the neonate [196, 199] although prematurity, low birth weight and neonatal antibiotics were associated to an increased prevalence of regurgitation in the first months of life [201–203].

Long term evolution of GERD in preterm newborns is uncertain. In one report, esophageal adenocarcinoma was found to be highly associated (11-fold risk) to preterm and small-for-gestational age birth [204]. In a nested case-control study no significant relation was found to birth weight [205].

At present, there is no validated neonatal questionnaire or clinical score that accurately identify preterm newborns with pathological reflux or esophagitis or who may benefit from pharmacological reflux treatment. There is currently insufficient evidence of correlation and limited studies performed with simultaneous cardio-monitoring or polysomnography and MII-pH, making the diagnosis of GERD in this population still challenging, and symptom association with GER generally inconsistent [206]. A systematic review on the relationship between apnoea and GER in preterm neonates included 7 studies and found inconclusive results: increased frequency of apnoea after reflux compared to reflux-free period was reported in 3 studies whereas other 4 studies did not show a significant temporal association [207]. MII-pH is the best investigation to detect a temporal symptom–reflux association, but it is not often performed in Neonatal Units because of the lack of specific probes and expertise, the cost of the device and catheters, low feasibility in ventilated and very low birth weight preterm infants, and limited reference data in this age group. Abdominal ultrasound, X-ray, and gastrointestinal contrast studies are indicated to identify anatomical abnormalities but have no diagnostic value for GERD [2].

Reduction in the volume and/or fractioning of meals is often attempted to improve feeding tolerance. There is no evidence on the beneficial effect of non-nutritive sucking and pacifier on GERD in preterm neonates and, to date, no RCT compared the effect of continuous versus intermittent bolus tube feeding on GERD in preterm and low birth weight infants. Breast feeding or human milk should always be preferred in this vulnerable population for multiple beneficial effects, including better feeding tolerance and faster gastric emptying time compared to formulas. Extensive protein hydrolysed formulas showed efficacy in reducing GER in preterm formula fed infants [78] by accelerating gastric emptying and/or by treating eventually associated cow’s milk allergy. However, another trial did not show a significant benefit by using hydrolysed formulas in preterm infants with symptoms attributed to GER [208]. In this age group, protracted use of hydrolysed formulas should be carefully monitored due to possible insufficient protein and caloric content and intake. Thickening agents and thickened formulas are not recommended [111], because adverse effects including necrotizing enterocolitis, diarrhea, metabolic acidosis, and hypokalaemia have been reported in infants who were born premature [110, 112].

Safe sleep approaches and supine positioning on a flat and firm surface is recommended by American Academy of Pediatrics and endorsed by all National Pediatric Societies to reduce the risk of Sudden Infant death Syndrome [209]. In selected severe cases, placing infants in the right lateral position for the first postprandial hour and thereafter in the left lateral or prone position can be considered, ensuring close cardiorespiratory monitoring, as this positional treatment may enhance gastric emptying and reduce TLESR and reflux events [210–212].

In two studies enrolling preterm infants, an alginate formulation significantly decreased the number of acid GER and proximal esophageal reflux episodes during treatment [213, 214], although no reduction of apnoea was reported [214]. In a more recent study, including both preterm and term infants with suspect GERD submitted to 48-hours MII-pH tracings, two different alginate formulations showed similar efficacy in reducing total reflux episodes and symptom associated to GER events in approximately 70% of infants [120]. Unfortunately, a subgroups analysis of preterm infants was not performed. There is no evidence that prokinetics are effective on GERD in premature infants, while QTc prolongation is a possible unpredictable serious adverse event of both erythromycin and domperidone [215, 216]. In a double-blind RCT with a crossover design, Omari et al. showed a significantly reduction in the frequency of acid GER events and esophageal acid exposure, as detected by twenty-four-hour esophageal and gastric pH monitoring performed on days 7 and 14, but not in the symptomatic events in 10 preterm infants treated with omeprazole for seven days [45]. In another double-blid RCT, 52 premature neonates received esomeprazole 0.5 mg/kg or placebo once daily for 14 days and were assessed with simultaneous MII-pH, cardiorespiratory, and 8-hour video monitoring. The percentage of esophageal acid exposure and the number of acid reflux lasting more than 5 min significantly decreased more with esomeprazole compared to placebo, but no significant change was found in the two treatment groups in signs and symptoms (cardiorespiratory events, choking, irritability and vomiting) [32]. These two trials were the only studies considered eligible and included in a recent Cochrane Systematic review [217] assessing safety and efficacy of PPIs in preterm infants.

Despite a lack of evidence and growing safety concerns (e.g., necrotizing enterocolitis, increased risk of infections, alteration of the microbiota, and mineral absorption), empiric treatment with acid-suppressing agents has continued to be widely used worldwide in neonates and neonatal units [7, 8]. Except in cases with gastroesophageal congenital abnormalities (diaphragmatic hernia, hiatal hernia, esophageal atresia and tracheoesophageal fistula), empirical treatment with acid inhibitors is not recommended in this age group. Investigations including MII-pH should be performed to prove or rule out the presence of GERD and to identify the appropriate treatment for the individual patient in persistent otherwise unexplained symptoms.

**Table:** 

**PRACTICE POINTS:**
• Episodes of GER and regurgitation are common in preterm newborns, due to frequent feeding, lying position, delayed gastric emptying, immaturity of LES and esophageal motility and possible associated conditions (i.e. bronchopulmonary dysplasia, infections) or treatment (i.e. ventilation, xanthine).
• GERD is frequently suspected in preterm newborns with respiratory symptoms, feeding difficulties, or irritability; however, the causal relationship remains uncertain. Empirical treatment with acid-suppressive agents is not recommended because of the lack of proven clinical efficacy and the potential risk of adverse effects in this vulnerable population.
• Conservative management including feeding modification (i.e. smaller and more frequent feeding, hydrolysed protein formulas) and alginate formulations can be considered in selected preterm infants with persistent symptoms
• Thickened feeding and use of prokinetics in preterm newborns is not recommended for GERD because of possible serious adverse effects
• PPIs should be reserved to preterm newborns with demonstrated acid GERD and acid related symptoms by using MII-pH (with eventual combined polysomnography or cardiorespiratory monitoring)

### Non-erosive reflux disease (NERD)

GERD patients are currently classified into two major subgroups: *erosive reflux disease (ERD*) and *NERD*,* d*epending on the presence or absence of erosive disease evaluated via upper GI endoscopy and identified by visible endoscopic breaks or erosions in the esophageal mucosa [2]. In children with persistent reflux symptoms despite 4–8 weeks of acid suppression therapy and a negative upper GI endoscopy for erosions, ideally performed off-PPIs therapy to enhance diagnostic accuracy [218], the 2018 ESPGHAN-NASPGHAN guideline [2] recommend to perform MII-pH to distinguish between different reflux phenotypes.

Based on the results of MII-pH three *distinct non-erosive esophageal phenotypes (NEEPs*) can be identified: (a) non-erosive reflux disease (“true” NERD), characterized by abnormal esophageal acid exposure regardless of symptom correlation; (b) reflux hypersensitivity (RH), defined by normal esophageal acid exposure but a positive symptom association with acid or non-acid reflux; and (c) functional heartburn (FH), characterized by normal esophageal acid exposure with no symptom association. A fourth subgroup—normal Reflux Index-not otherwise specified (“normal RI-NOS”) includes patients with normal acid exposure, but unreliable symptom association due to a very limited number of reported symptoms during testing. Although not yet clearly defined, this last group is unlikely to benefit from acid-suppressive therapy. Therefore, accurate classification of NEEPs is essential, as each phenotype has distinct pathogenic mechanisms and may respond differently to medical and surgical interventions [219–222].

### Epidemiology

NERD is the most common form of GERD across all ages [219]. Erosive esophagitis is less frequent, particularly in children, occurring in only 12.4% compared to 13–30% in adults [223, 224]. Only a few studies have assessed NEEPs in children using Rome IV criteria [12, 225, 226]. In one study of 45 children (≥ 5 years) who underwent both upper GI endoscopy and MII-pH testing off PPIs therapy, 44% were diagnosed with FH, 29% with RH, and 27% with NERD, the last being more common in older children [225]. A European multicentre retrospective study [12] of 68 children who underwent both upper GI endoscopy and MII-pH off-therapy, confirmed FH as the most common non-erosive reflux phenotype (38.2%), followed by NERD (26.5%) and acid RH (20.6%). About 14.7% had an undetermined phenotype (normal-RI-NOS), but even if these were all considered reflux hypersensitivity, FH would still be the predominant phenotype [12]. In a prospective observational study [226], RH was the most prevalent phenotype (40.8%), followed by FH at 26.5% and NERD at 24.5%; however, if the 8.2% of patients with normal RI-NOS were reclassified as having FH, the prevalence of FH aligns more closely with that reported in previous studies. In contrast, adult studies using the same diagnostic criteria show a higher prevalence of true NERD (40–58.5%) and a lower prevalence of RH (14.6–35%) compared to pediatric studies; FH in adults ranges from 21.9% to 35.35% [221, 227].

### Pathophysiology of NEEPs

Reflux symptoms arise from varying contributions of acid exposure and esophageal hypersensitivity [224]. In erosive esophagitis, acid exposure is the primary driver; in FH hypersensitivity dominates whilst “true” NERD and RH involve both factors. In RH and FH, where acid has a limited role, other mechanisms—such as mucosal barrier dysfunction, central and peripheral sensitization, psychological factors, and genetic predisposition—play a central part, interacting via the brain-gut axis [228–236]. The differing prevalence of NEEPs between children and adults is thought to result from variations in mucosal nerve fibres distribution, levels of inflammation, and degrees of sensory sensitization [232]. Children may have more peripheral and central sensitization [237] and show deeper esophageal nerve fibres and less acid-sensing transient receptor transient receptor potential vanilloid-1 (TRPV1 expression [238], unlike adults with NERD who exhibit more superficial nerves and TRPV1 overexpression [239]. TRPV1 receptor is a nociceptive ion channel expressed on esophageal sensory nerve endings that is activated by acid, heat, and inflammatory mediators. Activation of TRPV1 enhances neuronal excitability and contributes to visceral hypersensitivity, thereby amplifying the perception of reflux events even when acid exposure is within the normal range. Adults with FH have nerve fibres resembling healthy controls [240]. Children also show lower rates of microscopic esophagitis and erosive disease [223–225], suggesting better mucosal repair and less inflammation-driven nerve fiber displacement.

### Clinical presentation

Symptom patterns in children with NERD may differ by GERD phenotype: regurgitation is common in NERD, abdominal pain in functional disorders [225], and chest pain is seen in NERD and RI-NOS, but not in RH [12]. However, studies in both children and adults consistently show that symptoms alone cannot reliably predict GERD phenotypes [12, 225, 241]. In contrast, Savarino et al. [242] reported a higher prevalence of heartburn in FH adult patients and more frequent epigastric pain in those with NERD.

### Diagnosis

For patients with troublesome reflux symptoms, upper GI endoscopy helps classify them into ERD or NERD and MII-pH is pivotal to distinguish NEEPs. NEEP classification relies primarily on acid exposure rate and symptom association probability (SAP), the latter being the most robust for linking symptoms to reflux during MII-pH monitoring [243]. Newer MII-pH metrics, particularly the post-reflux swallow-induced peristaltic wave (PSPW) index and the mean nocturnal baseline impedance (MNBI) have also proven effective for distinguishing GERD phenotypes in adults [12, 226, 228, 244–246] as confirmed by a recent systematic review [247].

### Treatment

Management of NEEPs should be directed toward addressing the underlying pathophysiology and preventing complications of GERD. From a physiological perspective, acid suppression—primarily with PPIs—is central to managing acid-driven symptoms, particularly in cases of ERD, true NERD, and acid reflux hypersensitivity [248]. Data evaluating treatment in NEEPs are currently scanty and mostly derive from studies performed in adult patients. In NERD, combining alginates with PPIs therapy improves the chances of complete heartburn relief compared to using PPI alone, suggesting enhanced symptom control with adjunctive therapy [249, 250]. Additionally, antireflux surgery, such as fundoplication, has been shown to significantly improve outcomes in NERD adult patients especially those with PPI-refractory symptoms [251–253], as well as in cases with non-acid RH [254, 255].

Selective serotonin reuptake inhibitors (SSRIs), have shown effectiveness in treating RH [256, 257] and FH [258]: citalopram improved symptoms in 62% of RH patients vs. 33% on placebo and raised sensory thresholds in manometry studies [256, 257]. Fluoxetine led to more heartburn-free days in FH patients compared to omeprazole or placebo [258].

Studies on Tricyclic antidepressants (TCAs) show current inconsistent results: nortriptyline reduced brain activity related to acid exposure without improving symptoms [259], and imipramine improved quality of life but not symptom relief in RH or FH patients [260].

Complementary therapies such as hypnotherapy, acupuncture, and deep breathing may help manage refractory heartburn, especially in FH, although evidence is limited [261–263]. Cognitive behavioural therapy (CBT) shows stronger support, particularly in NERD patients with mood disorders, where it improves psychological outcomes and quality of life more effectively than medication alone [264].

Recent progress in understanding NEEPs has prompted a revaluation of previous diagnostic and treatment algorithms [248], including the development of new pediatric-specific strategies to guide clinical decision-making more effectively [265].

In conclusion, GERD treatment has moved toward personalized approaches based on individual pathophysiology. Prolonged PPIs use does not appear appropriate for subgroups other than ERD, true NERD, and acid-related RH. Other phenotypes, such as FH or non-acid-related RH and normal RI-NOS may respond better to alternative therapies like neuromodulators (mostly SSRIs), CBT, or complementary treatments, but further research is needed, particularly in pediatric populations.

**Table:** 

**PRACTICE POINTS**
• Based on the results of upper GI endoscopy (presence or absence of visible breaks or erosions in the esophageal mucosa), ppatients are commonly classified into two major subgroups: erosive reflux disease (ERD) and non-erosive reflux disease (NERD), the latter being the most common form of GERD across all ages.
• In NERD patients with persistent symptoms despite 4–8 weeks of acid suppression, 24-hour MII-pH is essential to further classify them into non-erosive esophageal phenotypes (NEEPs)— “true” NERD, reflux hypersensitivity (RH), functional heartburn (FH) and undetermined phenotype (normal RI-NOS) —each with distinct underlying mechanisms.
• Prolonged PPIs use should be reserved to ERD, true NERD, and acid-related RH, while FH, non-acid-related RH and normal RI-NOS may benefit from alternative treatments, such as neuromodulators.
• While adults show a higher prevalence of “true” NERD, in children the predominant phenotype seems to be FH. Therefore, extending PPIs therapy without proper classification of NEEPs is not recommended.

### Cystic fibrosis

Children with cystic fibrosis (CF) have an increased prevalence of GERD, which is linked to worsened lung function, more frequent pulmonary exacerbations, and failure to thrive [266–268]. The exact causes remain unclear, and whether GERD in CF is a primary condition or a consequence of lung disease is uncertain. Potential contributing factors include delayed gastric emptying, LES dysfunction, poor esophageal motility, pancreatic insufficiency, micro aspiration, and bronchospasm [269, 270]. Recent studies estimate GERD prevalence in CF children at 34%–46%, significantly higher than the 3.2%–18.8% reported in non-CF individuals [183, 194, 271].

Children with CF should follow the same diagnostic approach for GERD as non-CF patients. In absence of alarm signs, initial management includes lifestyle and dietary modifications. If heartburn is present, a first 4–8 weeks trial of PPIs is recommended. Persistent or recurring symptoms after PPIs suspension warrant further evaluation with upper GI endoscopy. If no erosions are found and symptoms continue, MII-pH is indicated [2, 272] for detecting both acid and non-acid reflux and for assessing symptom association and phenotypes of NEEPs. Studies indicate that MII-pH identifies 30% more GERD cases in CF children than pH-metry alone. While upper GI contrast studies are not recommended for routine GERD diagnosis, they may be useful before eventual antireflux surgery to identify associated anatomical anomalies [273]. Chronic PPIs use should be balanced against possible adverse pulmonary outcomes, increased hospitalizations for pulmonary exacerbations, and a higher risk of Pseudomonas aeruginosa colonization and Clostridioides difficile infections [274–278]. Additionally, PPIs do not prevent proximal reflux or bile acid aspiration, both of which can worsen lung health and may increase the osteoporosis risk of CF patients. Clinicians should regularly assess the need for prolonged PPIs therapy and deprescribing it, whenever possible, considering nutritional and respiratory status and the risk of rebound acid hypersecretion [279–282]. For respiratory GERD symptoms, treatment should be approached cautiously. A study on CF patients (median age ~ 11 years) found omeprazole reduced abdominal pain and GERD symptoms but had no significant impact on cough, with no major difference between the omeprazole and placebo groups [283–285]. While prokinetic agents such as domperidone, erythromycin, azithromycin, and metoclopramide are used in CF adult patients with GERD, no studies have evaluated their efficacy in children.

Among alternative treatments, CF transmembrane conductance regulator (CFTR) shows promise in addressing GERD in CF. A longitudinal cohort study of 32 adults with advanced CF lung disease found that treatment with elexacaftor-tezacaftor-ivacaftor significantly reduced reflux symptoms, with the reflux symptom index (RSI) score decreasing from 15 to 5 (*p* < 0.001) after six months [286]. These findings suggest CFTR modulators may improve GERD symptoms in CF patients, but further research is needed, particularly in pediatric populations.

Finally, antireflux surgery is considered in CF patients with acute life-threatening events, chronic aspiration, frequent pulmonary exacerbations, declining lung function, failure to thrive, or persistent GERD despite medical therapy. Approximately 10% of CF patients who fail optimal therapy require antireflux surgery. The primary goal of this intervention is to preserve lung function, extend life expectancy, and improve quality of life [287]. The available literature on anti-reflux surgery in CF is limited, with inconsistent outcome measures and mixed results. Some studies suggest that it improves gastrointestinal symptoms, lung function (FEV1), and pulmonary exacerbations requiring intravenous antibiotics, particularly in patients with severe lung disease. Fundoplication has been associated with improved weight gain in CF children and those receiving gastrostomy feeding. However, other studies report a high likelihood of persistent GERD symptoms and continued medication use post-surgery, with limited nutritional or pulmonary benefits [288]. Long-term outcomes remain unclear; pediatric CF report a complication rate of 18% within two years post-surgery, although further research is needed to establish standardized outcome measures and assess long-term efficacy.

### Syndromic children

In recent years there is an increasing evidence that selected syndromes carry an increased risk of GERD [2, 289] requiring a life-long surveillance starting in early infancy, emphasizing the need for structured and tailored follow-up. In such patients GERD is far more complex disease because it arises from a mosaic of defects or malformations. Multiple pathogenic mechanisms are involved:

- Systemic hypotonia and neuromotor dysmotility, which decrease the LES tone and slow esophageal clearances observed in Down syndrome, Prader-Willi syndrome, Rett syndrome and cerebral palsy.
- Esophageal and/or gastroesophageal malformations: Cornelia de Lange, CHARGE, VACTERL esophageal atresia.
- Connective-tissue laxity causing cardial incompetence and hiatal hernia: Ehlers-Danlos, Marfan syndrome, osteogenesis imperfecta.
- Raised intragastric pressure or delayed gastric emptying due to microgastria, obesity or severe scoliosis: Smith–Lemli–Opitz, Prader-Willi, Rett.

In Down syndrome [290], general hypotonia and a high prevalence of ineffective or absent peristalsis (81% on manometry) reduce LES pressure, prolong clearance time and account for a GERD prevalence of about 14%. In Rett syndrome [291], loss of MeCP2 gene within the enteric nervous system disrupts nitrergic signaling and produces gastroesophageal hypomotility compounded by scoliosis, resulting in GERD in 40–70% of cases. In Cornelia de Lange [292], the combination of micrognathia, congenital esophageal stenoses and weak peristalsis results in frequent severe GERD (about 65% of patients) with early progression to Barrett’s esophagus. CHARGE syndrome [293], with IX–X cranial-nerve palsies, silent aspiration and sphincter hypotonia, generates persistent GERD despite airway surgery. In VACTERL [294] and repaired esophageal atresia, the shortened and denervated esophagus exhibits aperistalsis and ineffective pressure transitions, while anastomotic tension displaces the LES into the thorax, making GERD almost inevitable. In hypermobile Ehlers-Danlos [295], connective-tissue laxity undermines cardial integrity, favors hiatal hernia and is associated with motility disorders (abnormal manometry in 70% of cases). Prader-Willi syndrome combines hypotonia, obesity, raising intra-abdominal pressure, delaying gastric emptying, gastroesophageal dysmotility, impaired gastric accommodation and, together with hyperphagia, predisposed to chronic GERD, particularly in obese patients or in recurrent respiratory problems [296]. Gastrointestinal complications are frequently reported in individuals affected from autism-spectrum disorder possible related to altered pain perception and communication, and atypical feeding habits [297, 298]. Autonomic imbalance may alter transient LES tone and relaxations, quadrupling GERD risk and atypical symptoms. In Smith–Lemli–Opitz [299], microgastria and malrotations that raise intragastric pressure and favor mixed acid–biliary reflux, have been reported. In Angelman syndrome [300], loss of UBE3A gene in the myenteric plexus causes profound hypotonia and markedly delayed gastric emptying, leading to GERD in 80% of children.

In summary, loss of the LES–diaphragm pressure gradient (hypotonia or laxity), impaired peristaltic clearance (neurogenic dysmotility), delayed emptying or elevated intragastric pressure (obesity, microgastria) and anatomical defects such as hiatal hernia or esophageal shortening often coexist in the aforementioned syndromes inducing chronic reflux and complicated GERD that demands individually tailored diagnostic and therapeutic strategies from the pediatric age onward, as illustrated in Table 2.

**Table 2:** The following tables summary the main pathogenic mechanisms inducing GERD and report GERD prevalence in specific syndromes

a. Hypotonia and neuromotor dysfunction		
	Mechanistic rationale	GERD prevalence
Down syndrome (trisomy 21)	Generalized hypotonia, delayed gastric emptying, esophageal dysmotility	14–30% in pediatric follow-up cohorts
Prader–Willi syndrome	Hypotonia, dysmotility, impaired gastric accommodation, central obesity/apnea	unclear; 52% acid GERD in one study
Smith–Lemli–Opitz syndrome	Microgastria and intestinal dysmotility	GER common in infancy, often refractory to fundoplication
Ehlers–Danlos (hEDS)	Connective-tissue laxity predisposes to LES incompetence	> 50% of patients report GERD symptoms

b Neurodevelopmental syndromes		
	Mechanistic rationale	GERD prevalence
Rett syndrome	Severe autonomic dysfunction, scoliosis, truncal hypotonia	≈ 40% with clinically significant GERD (guideline data)
ASD (autism spectrum disorder)	Altered pain perception, atypical feeding habits	4-fold higher risk of GERD and complications vs. general population
Angelman syndrome	Hypotonia and feeding problems from birth	Up to 80% show GI symptoms, including GERD
Syndromes associated with severe neurologic impairments	Gastroesophageal dysmotility, frequent abnormal posture and eventual prolonged supine time	Overall considered as “high-risk group” in ESPGHAN/NASPGHAN and NICE guidelines

c Cranio-facial / oesophageal malformations		
	Mechanistic rationale	GERD prevalence
Cornelia de Lange syndrome	Micrognathia, esophageal atresia/stenoses	Severe GERD in ≈ ⅔ of patients; high rate of fundoplication
CHARGE syndrome	Choanal atresia, cranial-nerve deficits predisposing to aspiration	> 60% of adolescents/adults with persistent reflux
VACTERL / VATER association/repaired esophageal atresia	Repaired tracheo-esophageal fistula / esophageal atresia with foreshortened esophagus and related dysmotility	45% of adults as reported in one study

d. Connective-tissue disorders / ligamentous laxity		
Example conditions:		
Marfan syndrome, osteogenesis imperfecta and other heritable connective-tissue disorders (CTDs) are frequently associated with hiatal hernia and esophageal dysmotility.		

### Clinical management

Physicians should maintain a high level of suspicion and awareness of the increased risk of GERD beginning in infancy and childhood in patients with the above-mentioned syndromes, particularly in the presence of esophageal malformations, generalized hypotonia, connective tissue disease, gastroesophageal dysmotility, severe respiratory symptoms, and impaired communication [2, 289, 301].

If indicated, the diagnostic workup is based on 24-hour MII-pH [302] to detect underlying GER and symptom association. Early upper GI endoscopy with biopsy is advocated in cases with failure to thrive, iron-deficiency anemia, recurrent chest pain, increased risk of eosinophilic esophagitis or Barrett’s esophagus (esophageal atresia, Cornelia de Lange), with screening commencing at about 10 years of life and repeated every 3–5 years [303].

The therapeutic recommendations for non-syndromic patients may also apply for these children, although management is intrinsically multidisciplinary [304]. In particular, the dietitian guarantees appropriate nutrition, caloric density and formula choice; the physiatrist and orthopedic addresses skeletal malformation, abnormal posturing and scoliosis to mitigate traction on the cardia; the physiotherapist acts on muscle tone and posturing; the neurologists and neuropsychiatric evaluate anti-epileptic treatment and neurodevelopmental disorders; the speech-language pathologist optimizes swallowing; pulmonologists, otolaryngologists and dentists manage aspiration, laryngomalacia and acid-related dental erosion. General wellness, growth, nutritional status, pharmacological therapy, respiratory infections and oral health should be reviewed, ideally, every six months even in asymptomatic patients but the follow-up approach can be targeted to a specific syndrome and individual patient. Family empowerment is also crucial: caregivers should be aware that irritability, self-injurious behavior, feeding or sleeping problems, respiratory infections, post-prandial cyanosis may signal GERD and esophageal pain. Video tutorials on gastrostomy placement and care, specific symptom charts, and a dedicated telemedicine service could reduce complications and hospital admissions for these children, while also enabling early detection of exacerbations. These approaches could be explored in the coming years. Implementation of this proactive protocol would likely improve quality of life and outcomes in these complex pediatric patients.

## Conclusions

This guideline provides 26 recommendations and practice points based on updated systematic reviews and expert consensus to assist clinicians in the management of infants, children, and adolescents with GERD. Compared to previous guidelines, empiric treatment with PPIs is not recommended in infants and patients with extraesophageal symptoms, due to the lack of evidence of efficacy and potential adverse effects, particularly with prolonged use. Several non-pharmacological approaches, the characterization of GERD and NEEP phenotypes, and conditions at risk for GERD have also been reviewed and updated, and practice points for managing complex patients are illustrated. Newborns and infants frequently experience reflux and regurgitation, which, in most cases, resolves spontaneously or after dietary modification without the need for pharmacological intervention. In children who report heartburn or severe neurologic impairment, an empiric course of PPIs is recommended, but failure to respond has been increasingly reported. For persistent symptoms, MII-esophageal pH and upper GI endoscopy are indicated to detect and characterize reflux, GERD phenotypes, and esophagitis. PPIs treatment is the first-line treatment for reflux esophagitis or acid GERD, but long-term treatment must be carefully monitored. Surgery should be reserved for severe, refractory GERD, following a thorough diagnostic workup and optimal drug therapy. Subjects predisposed to severe GERD have been identified, requiring personalized and often multidisciplinary treatment.

## Electronic Supplementary Material

Below is the link to the electronic supplementary material.

Additional File 1

Additional File 2

Additional File 3

Additional File 4

Additional File 5

## Data Availability

Not applicable.

## Appendix

**Table 3:** Guideline development group

Name	Role	Area of Expertise	Institution	City, Country
Barbara Polistena	Evidence Review Team	Methodology / Evidence Synthesis	C.R.E.A. Sanità (Centre for Applied Economic Research in Healthcare)	Rome, Italy
Massimiliano Orso	Evidence Review Team	Methodology / Evidence Synthesis	C.R.E.A. Sanità (Centre for Applied Economic Research in Healthcare)	Rome, Italy
Liliana Guadagni	Evidence Review Team	Methodology / Evidence Synthesis	Department of Surgical and Biomedical Sciences, University of Perugia	Perugia, Italy
Claudio Lo Giudice	Evidence Review Team	Methodology / Evidence Synthesis	C.R.E.A. Sanità (Centre for Applied Economic Research in Healthcare)	Rome, Italy
Ferdinando Verneau	Evidence Review Team	Methodology / Evidence Synthesis	C.R.E.A. Sanità (Centre for Applied Economic Research in Healthcare)	Rome, Italy
Gaetano Caforio	Evidence Review Team	Methodology / Evidence Synthesis	C.R.E.A. Sanità (Centre for Applied Economic Research in Healthcare)	Rome, Italy

All panel members participated in at least one plenary meeting and contributed to the formulation, discussion, and/or voting of recommendations. Members of the Evidence Review Team conducted literature searches, data extraction, and GRADE assessments.
