# Supplementary material for: Guidelines on diagnosis and management of gastroesophageal reflux disease in infants, children and adolescents: a joint consensus from Italian pediatric societies (SIP and SIGENP) -Part II: management
Source: Ital J Pediatr. 2026 Apr 10;52:90. doi: 10.1186/s13052-026-02255-0 (PMC13182049; doi:10.1186/s13052-026-02255-0)
Supplement: Supplementary file 1 — Additional File 1 [file 13052_2026_2255_MOESM1_ESM.docx]

# Additional File 1

## Search strategy

### Search strategy to identify guidelines and consensus statements on gastroesophageal reflux (GER) and gastroesophageal reflux disease (GERD) in infants, children, and adolescents

|  | PubMed, 22/02/2024 |  |
| --- | --- | --- |
| Search | Query | Results |
| #1 | guideline[Title] OR guidelines[Title] OR consensus[Title] OR recommendation[Title] OR recommendations[Title] OR "guideline"[Publication Type] OR "practice guideline"[Publication Type] OR "consensus development conference"[Publication Type] | 194,681 |
| #2 | gastroesophageal reflux[Title/Abstract] OR gastro esophageal reflux[Title/Abstract] OR gastro-oesophageal reflux[Title/Abstract] OR GERD[Title/Abstract] OR GORD[Title/Abstract] | 30,097 |
| #3 | child OR children OR pediatric OR pediatrics OR paediatric OR paediatrics OR infant OR infants OR newborn OR newborns OR adolescent OR adolescents OR juvenile OR youth OR toddler OR toddlers OR kid OR kids OR baby OR babies OR teen OR teens OR preteen OR preteens OR neonate OR neonates OR (allchild[Filter] OR newborn[Filter] OR allinfant[Filter] OR infant[Filter] OR child[Filter] OR adolescent[Filter] OR preschoolchild[Filter]) | 5,464,813 |
| #4 | #1 AND #2 AND #3 | 126 |
| #5 | #1 AND #2 AND #3 Filters: English, from 2000 - 2024 | 97 |

|  | Embase, 22/02/2024 |  |
| --- | --- | --- |
| Search | Query | Results |
| #1 | guideline:ti OR guidelines:ti OR consensus:ti OR recommendation:ti OR recommendations:ti | 224,578 |
| #2 | (gastroesophageal reflux):ti,ab OR (gastro esophageal reflux):ti,ab OR (gastro-oesophageal reflux):ti,ab OR GERD:ti,ab OR GORD:ti,ab | 50,728 |
| #3 | child OR children OR pediatric OR pediatrics OR paediatric OR paediatrics OR infant OR infants OR newborn OR newborns OR adolescent OR adolescents OR juvenile OR youth OR toddler OR toddlers OR kid OR kids OR baby OR babies OR teen OR teens OR preteen OR preteens OR neonate OR neonates | 6,294,944 |
| #4 | #1 AND #2 AND #3 | 145 |
| #5 | #1 AND #2 AND #3 AND [english]/lim AND [2000-2024]/py | 110 |

|  | Web of Science, 22/02/2024 |  |
| --- | --- | --- |
| Search | Query | Results |
| #1 | TI=(guideline OR guidelines OR consensus) | 165,848 |
| #2 | TS=(gastroesophageal reflux OR gastro esophageal reflux OR gastro-oesophageal reflux OR GERD OR GORD) | 41,240 |
| #3 | TS=(child OR children OR pediatric OR pediatrics OR paediatric OR paediatrics OR infant OR infants OR newborn OR newborns OR adolescent OR adolescents OR juvenile OR youth OR toddler OR toddlers OR kid OR kids OR baby OR babies OR teen OR teens OR preteen OR preteens OR neonate OR neonates) | 3,118,367 |
| #4 | #1 AND #2 AND #3 | 103 |
| #5 | (#1 AND #2 AND #3) AND PY=(2000-2024) AND LA=(English) | 93 |

| Google Scholar, 22/02/2024 | | |
| --- | --- | --- |
| Search | Query | Results |
| #1 | (guideline OR guidelines OR consensus) AND (gastroesophageal reflux OR gastro esophageal reflux OR gastro-oesophageal reflux OR GERD OR GORD) AND (child OR children OR pediatric OR pediatrics OR paediatric OR paediatrics OR infant OR infants OR newborn OR newborns OR adolescent OR adolescents OR juvenile OR youth OR toddler OR toddlers OR kid OR kids OR baby OR babies OR teen OR teens OR preteen OR preteens OR neonate OR neonates) | first 50 results |

### PICO 5 - What is the evidence for the effectiveness of pharmacological treatments for GER and GERD in infants, children, and adolescents?

| PubMed, 12/05/2024 | | |
| --- | --- | --- |
| Search | Query | Results |
| #1 | gastroesophageal reflux OR gastro esophageal reflux OR gastrooesophageal reflux OR gastro oesophageal reflux OR GER OR GERD OR GOR OR GORD OR (gastric[Title/Abstract] AND (acid[Title/Abstract] OR reflux[Title/Abstract])) OR (reflux[Title/Abstract] AND (oesophagitis[Title/Abstract] OR esophagitis[Title/Abstract])) OR (erosive[Title/Abstract] AND (oesophag*[Title/Abstract] OR esophag*[Title/Abstract])) OR (regurgitation[Title/Abstract] NOT (aortic[Title/Abstract] OR mitral[Title/Abstract] OR tricuspid[Title/Abstract] OR valve[Title/Abstract] OR valvular[Title/Abstract] OR paravalvular[Title/Abstract] OR pulmonary [Title/Abstract])) OR "acid reflux"[Title/Abstract] | 95,574 |
| #2 | child OR children OR pediatric OR pediatrics OR paediatric OR paediatrics OR infant OR infants OR infancy OR newborn OR newborns OR adolescent OR adolescents OR juvenile OR youth OR toddler OR toddlers OR kid OR kids OR boy OR boys OR girl OR girls OR baby OR babies OR teen OR teens OR preteen OR preteens OR teenager OR teenagers OR pubescen* OR prepubescen* OR neonate OR neonates OR (allchild[Filter] OR newborn[Filter] OR allinfant[Filter] OR infant[Filter] OR child[Filter] OR adolescent[Filter] OR preschoolchild[Filter]) | 6,805,307 |
| #3 | "Antacids"[Mesh] OR "Alginates"[Mesh] OR "Proton Pump Inhibitors"[Mesh] OR "Ranitidine"[Mesh] OR "Nizatidine"[Mesh] OR "Cimetidine"[Mesh] OR "Famotidine"[Mesh] OR "lansoprazole"[Mesh] OR "omeprazole"[Mesh] OR "esomeprazole"[Mesh] OR "rabeprazole"[Mesh] OR "cisapride"[Mesh] OR "domperidone"[Mesh] OR "Metoclopramide"[Mesh] OR "erythromycin"[Mesh] OR "azithromycin"[Mesh] OR "clarithromycin"[Mesh] OR "Histamine H2 Antagonists" [Mesh] OR "Aluminum Hydroxide"[Mesh] OR "sodium bicarbonate"[Mesh] OR "calcium carbonate"[Mesh] OR (alginate*[Title/Abstract] OR gaviscon[Title/Abstract] OR almagate[Title/Abstract] OR almax[Title/Abstract] OR tisacid[Title/Abstract] OR magnesium hydroxide[Title/Abstract] OR brucite[Title/Abstract] OR magnesium hydrate[Title/Abstract] OR "milk of magnesia"[Title/Abstract] OR aluminum hydroxide[Title/Abstract] OR aldrox[Title/Abstract] OR algeldrate[Title/Abstract] OR alhydrogel[Title/Abstract] OR aloh-gel[Title/Abstract] OR alternagel[Title/Abstract] OR alu-cap[Title/Abstract] OR alu-tab[Title/Abstract] OR alugel[Title/Abstract] OR amphojel[Title/Abstract] OR andursil[Title/Abstract] OR brasivil[Title/Abstract] OR dialume[Title/Abstract] OR hydrated alumina[Title/Abstract] OR rocgel[Title/Abstract] OR maalox*[Title/Abstract] OR co-magaldrox[Title/Abstract] OR alucol[Title/Abstract] OR (alumina[Title/Abstract] AND magnesia[Title/Abstract]) OR aluminum hydroxide-magnesium hydroxide[Title/Abstract] OR aluminum magnesium hydroxide[Title/Abstract] OR co-magaldrox[Title/Abstract] OR magnesium aluminum hydroxide[Title/Abstract] OR maldroxal[Title/Abstract] OR mintox[Title/Abstract] OR novalucol[Title/Abstract] OR supralox[Title/Abstract] OR H2 antagonist*[Title/Abstract] OR ranitidin*[Title/Abstract] OR biotidin[Title/Abstract] OR pylorid[Title/Abstract] OR raniberl[Title/Abstract] OR ranisen[Title/Abstract] OR rantec[Title/Abstract] OR sostril[Title/Abstract] OR tritec[Title/Abstract] OR zantac[Title/Abstract] OR cimetidine[Title/Abstract] OR altramet[Title/Abstract] OR biomet[Title/Abstract] OR histodil[Title/Abstract] OR tagamet[Title/Abstract] OR zita[Title/Abstract] OR famotidine[Title/Abstract] OR pepcid[Title/Abstract] OR ym 11170[Title/Abstract] OR PPI[Title/Abstract] OR PPIs[Title/Abstract] OR proton pump inhibitor*[Title/Abstract] OR lansoprazol*[Title/Abstract] OR dexlansoprazole[Title/Abstract] OR agopton[Title/Abstract] OR lanzoprazol*[Title/Abstract] OR lanzor[Title/Abstract] OR prevacid[Title/Abstract] OR takepron[Title/Abstract] OR zoton[Title/Abstract] OR pantoprazole[Title/Abstract] OR protonix[Title/Abstract] OR pantozol[Title/Abstract] OR pantoloc[Title/Abstract] OR controloc[Title/Abstract] OR omeprazole[Title/Abstract] OR losec[Title/Abstract] OR nexium[Title/Abstract] OR prilosec[Title/Abstract] OR rapinex[Title/Abstract] OR zegerid[Title/Abstract] OR OMEZ[Title/Abstract] OR antra[Title/Abstract] OR mopral[Title/Abstract] OR rabeprazole[Title/Abstract] OR aciphex[Title/Abstract] OR dexrabeprazole[Title/Abstract] OR pariet[Title/Abstract] OR esomeprazol*[Title/Abstract] OR nexium[Title/Abstract] OR nexiam[Title/Abstract] OR metoclopramide[Title/Abstract] OR cerucal[Title/Abstract] OR degan[Title/Abstract] OR gastrobid continus[Title/Abstract] OR gastromax[Title/Abstract] OR maxolon[Title/Abstract] OR maxeran[Title/Abstract] OR metaclopramide[Title/Abstract] OR octamide[Title/Abstract] OR primperan[Title/Abstract] OR reglan[Title/Abstract] OR rimetin[Title/Abstract] OR domperidon*[Title/Abstract] OR motilium[Title/Abstract] OR kw 5338[Title/Abstract] OR erythromycin[Title/Abstract] OR aknemycin[Title/Abstract] OR T stat[Title/Abstract] OR e-base[Title/Abstract] OR emycin[Title/Abstract] OR emgel[Title/Abstract] OR eryc[Title/Abstract] OR eryderm[Title/Abstract] OR erythro[Title/Abstract] OR erythroped[Title/Abstract] OR gallimycin[Title/Abstract] OR ilosone[Title/Abstract] OR ilotycin[Title/Abstract] OR monomycin[Title/Abstract] OR romycin[Title/Abstract] OR staticin[Title/Abstract] OR stiemycin[Title/Abstract] OR theramycin[Title/Abstract] OR wyamycin[Title/Abstract] OR bethanechol[Title/Abstract] OR bethanecol[Title/Abstract] OR myotonine[Title/Abstract] OR urecholine[Title/Abstract] OR sucralfate[Title/Abstract] OR aluminum sucrose sulfate[Title/Abstract] OR antepsin[Title/Abstract] OR carafate[Title/Abstract] OR sutra[Title/Abstract] OR sulcrate[Title/Abstract] OR ulcogant[Title/Abstract] OR ulsanic[Title/Abstract] OR nizatidine[Title/Abstract] OR cisapride[Title/Abstract] OR azithromycin[Title/Abstract] OR clarithromycin[Title/Abstract] OR prokinetic agent*[Title/Abstract] OR prokinetic*[Title/Abstract] OR hydrotalcite[Title/Abstract] OR asilone[Title/Abstract] OR gastrocote[Title/Abstract] OR topal[Title/Abstract] OR sodium alginate*[Title/Abstract] OR altacite[Title/Abstract] OR sodium bicarbonate[Title/Abstract]) OR (sodium[Title/Abstract] AND alginate*[Title/Abstract]) | 204,102 |
| #4 | #1 AND #2 AND #3 | 3,021 |
| #5 | #4 AND ((clinicaltrial[Filter] OR controlledclinicaltrial[Filter] OR meta-analysis[Filter] OR randomizedcontrolledtrial[Filter] OR systematicreview[Filter]) AND english[Filter]) | 779 |

| Embase, 12/05/2024 | | |
| --- | --- | --- |
| Search | Query | Results |
| #1 | 'gastroesophageal reflux'/exp OR 'gastroesophageal reflux':ti,ab,kw OR 'gastro esophageal reflux':ti,ab,kw OR 'gastrooesophageal reflux':ti,ab,kw OR 'gastro oesophageal reflux':ti,ab,kw OR ger:ti,ab,kw OR gerd:ti,ab,kw OR gor:ti,ab,kw OR gord:ti,ab,kw OR (gastric:ti,ab,kw AND (acid:ti,ab,kw OR reflux:ti,ab,kw)) OR (reflux:ti,ab,kw AND (oesophagitis:ti,ab,kw OR esophagitis:ti,ab,kw)) OR (erosive:ti,ab,kw AND (oesophag*:ti,ab,kw OR esophag*:ti,ab,kw)) OR (regurgitation:ti,ab,kw NOT (aortic:ti,ab,kw OR mitral:ti,ab,kw OR tricuspid:ti,ab,kw OR valve:ti,ab,kw OR valvular:ti,ab,kw OR paravalvular:ti,ab,kw OR pulmonary:ti,ab,kw)) OR 'acid reflux':ti,ab,kw | 143,723 |
| #2 | child:ti,ab,kw OR children:ti,ab,kw OR pediatric:ti,ab,kw OR pediatrics:ti,ab,kw OR paediatric:ti,ab,kw OR paediatrics:ti,ab,kw OR infant:ti,ab,kw OR infants:ti,ab,kw OR infancy:ti,ab,kw OR newborn:ti,ab,kw OR newborns:ti,ab,kw OR adolescent:ti,ab,kw OR adolescents:ti,ab,kw OR juvenile:ti,ab,kw OR youth:ti,ab,kw OR toddler:ti,ab,kw OR toddlers:ti,ab,kw OR kid:ti,ab,kw OR kids:ti,ab,kw OR boy:ti,ab,kw OR boys:ti,ab,kw OR girl:ti,ab,kw OR girls:ti,ab,kw OR baby:ti,ab,kw OR babies:ti,ab,kw OR teen:ti,ab,kw OR teens:ti,ab,kw OR preteen:ti,ab,kw OR preteens:ti,ab,kw OR teenager:ti,ab,kw OR teenagers:ti,ab,kw OR pubescen*:ti,ab,kw OR prepubescen*:ti,ab,kw OR neonate:ti,ab,kw OR neonates:ti,ab,kw | 3,532,866 |
| #3 | 'antacid agent'/exp OR 'alginic acid'/exp OR 'proton pump inhibitor'/exp OR 'ranitidine'/exp OR 'nizatidine'/exp OR 'cimetidine'/exp OR 'famotidine'/exp OR 'lansoprazole'/exp OR 'omeprazole'/exp OR 'esomeprazole'/exp OR 'rabeprazole'/exp OR 'cisapride'/exp OR 'domperidone'/exp OR 'metoclopramide'/exp OR 'erythromycin'/exp OR 'azithromycin'/exp OR 'clarithromycin'/exp OR 'histamine H2 receptor antagonist'/exp OR 'aluminum hydroxide'/exp OR 'bicarbonate'/exp OR 'calcium carbonate'/exp OR (alginate*:ti,ab,kw OR gaviscon:ti,ab,kw OR almagate:ti,ab,kw OR almax:ti,ab,kw OR tisacid:ti,ab,kw OR magnesium hydroxide:ti,ab,kw OR brucite:ti,ab,kw OR magnesium hydrate:ti,ab,kw OR 'milk of magnesia':ti,ab,kw OR aluminum hydroxide:ti,ab,kw OR aldrox:ti,ab,kw OR algeldrate:ti,ab,kw OR alhydrogel:ti,ab,kw OR aloh-gel:ti,ab,kw OR alternagel:ti,ab,kw OR alu-cap:ti,ab,kw OR alu-tab:ti,ab,kw OR alugel:ti,ab,kw OR amphojel:ti,ab,kw OR andursil:ti,ab,kw OR brasivil:ti,ab,kw OR dialume:ti,ab,kw OR hydrated alumina:ti,ab,kw OR rocgel:ti,ab,kw OR maalox*:ti,ab,kw OR co-magaldrox:ti,ab,kw OR alucol:ti,ab,kw OR (alumina:ti,ab,kw AND magnesia:ti,ab,kw) OR aluminum hydroxide-magnesium hydroxide:ti,ab,kw OR aluminum magnesium hydroxide:ti,ab,kw OR co-magaldrox:ti,ab,kw OR magnesium aluminum hydroxide:ti,ab,kw OR maldroxal:ti,ab,kw OR mintox:ti,ab,kw OR novalucol:ti,ab,kw OR supralox:ti,ab,kw OR H2 antagonist*:ti,ab,kw OR ranitidin*:ti,ab,kw OR biotidin:ti,ab,kw OR pylorid:ti,ab,kw OR raniberl:ti,ab,kw OR ranisen:ti,ab,kw OR rantec:ti,ab,kw OR sostril:ti,ab,kw OR tritec:ti,ab,kw OR zantac:ti,ab,kw OR cimetidine:ti,ab,kw OR altramet:ti,ab,kw OR biomet:ti,ab,kw OR histodil:ti,ab,kw OR tagamet:ti,ab,kw OR zita:ti,ab,kw OR famotidine:ti,ab,kw OR pepcid:ti,ab,kw OR ym 11170:ti,ab,kw OR PPI:ti,ab,kw OR PPIs:ti,ab,kw OR proton pump inhibitor*:ti,ab,kw OR lansoprazol*:ti,ab,kw OR dexlansoprazole:ti,ab,kw OR agopton:ti,ab,kw OR lanzoprazol*:ti,ab,kw OR lanzor:ti,ab,kw OR prevacid:ti,ab,kw OR takepron:ti,ab,kw OR zoton:ti,ab,kw OR pantoprazole:ti,ab,kw OR protonix:ti,ab,kw OR pantozol:ti,ab,kw OR pantoloc:ti,ab,kw OR controloc:ti,ab,kw OR omeprazole:ti,ab,kw OR losec:ti,ab,kw OR nexium:ti,ab,kw OR prilosec:ti,ab,kw OR rapinex:ti,ab,kw OR zegerid:ti,ab,kw OR OMEZ:ti,ab,kw OR antra:ti,ab,kw OR mopral:ti,ab,kw OR rabeprazole:ti,ab,kw OR aciphex:ti,ab,kw OR dexrabeprazole:ti,ab,kw OR pariet:ti,ab,kw OR esomeprazol*:ti,ab,kw OR nexium:ti,ab,kw OR nexiam:ti,ab,kw OR metoclopramide:ti,ab,kw OR cerucal:ti,ab,kw OR degan:ti,ab,kw OR gastrobid continus:ti,ab,kw OR gastromax:ti,ab,kw OR maxolon:ti,ab,kw OR maxeran:ti,ab,kw OR metaclopramide:ti,ab,kw OR octamide:ti,ab,kw OR primperan:ti,ab,kw OR reglan:ti,ab,kw OR rimetin:ti,ab,kw OR domperidon*:ti,ab,kw OR motilium:ti,ab,kw OR kw 5338:ti,ab,kw OR erythromycin:ti,ab,kw OR aknemycin:ti,ab,kw OR T stat:ti,ab,kw OR e-base:ti,ab,kw OR emycin:ti,ab,kw OR emgel:ti,ab,kw OR eryc:ti,ab,kw OR eryderm:ti,ab,kw OR erythro:ti,ab,kw OR erythroped:ti,ab,kw OR gallimycin:ti,ab,kw OR ilosone:ti,ab,kw OR ilotycin:ti,ab,kw OR monomycin:ti,ab,kw OR romycin:ti,ab,kw OR staticin:ti,ab,kw OR stiemycin:ti,ab,kw OR theramycin:ti,ab,kw OR wyamycin:ti,ab,kw OR bethanechol:ti,ab,kw OR bethanecol:ti,ab,kw OR myotonine:ti,ab,kw OR urecholine:ti,ab,kw OR sucralfate:ti,ab,kw OR aluminum sucrose sulfate:ti,ab,kw OR antepsin:ti,ab,kw OR carafate:ti,ab,kw OR sutra:ti,ab,kw OR sulcrate:ti,ab,kw OR ulcogant:ti,ab,kw OR ulsanic:ti,ab,kw OR nizatidine:ti,ab,kw OR cisapride:ti,ab,kw OR azithromycin:ti,ab,kw OR clarithromycin:ti,ab,kw OR prokinetic agent*:ti,ab,kw OR prokinetic*:ti,ab,kw OR hydrotalcite:ti,ab,kw OR asilone:ti,ab,kw OR gastrocote:ti,ab,kw OR topal:ti,ab,kw OR sodium alginate*:ti,ab,kw OR altacite:ti,ab,kw OR sodium bicarbonate:ti,ab,kw) OR (sodium:ti,ab,kw AND alginate*:ti,ab,kw) | 506,616 |
| #4 | #1 AND #2 AND #3 | 3,666 |
| #5 | #1 AND #2 AND #3 AND ([article]/lim OR [article in press]/lim OR [review]/lim) AND [english]/lim AND ('clinical trial'/de OR 'clinical trial topic'/de OR 'comparative effectiveness'/de OR 'controlled clinical trial'/de OR 'controlled study'/de OR 'double blind procedure'/de OR 'meta analysis'/de OR 'meta analysis topic'/de OR 'randomized controlled trial'/de OR 'randomized controlled trial topic'/de OR 'systematic review'/de OR 'systematic review topic'/de) | 859 |

| Web of Science, 12/05/2024 | | |
| --- | --- | --- |
| Search | Query | Results |
| #1 | TS=(gastroesophageal reflux OR gastro esophageal reflux OR gastrooesophageal reflux OR gastro oesophageal reflux OR GER OR GERD OR GOR OR GORD OR (gastric AND (acid OR reflux)) OR (reflux AND (oesophagitis OR esophagitis)) OR (erosive AND (oesophag* OR esophag*)) OR (regurgitation NOT (aortic OR mitral OR tricuspid OR valve OR valvular OR paravalvular OR pulmonary )) OR "acid reflux") | 97,816 |
| #2 | TS=(child OR children OR pediatric OR pediatrics OR paediatric OR paediatrics OR infant OR infants OR infancy OR newborn OR newborns OR adolescent OR adolescents OR juvenile OR youth OR toddler OR toddlers OR kid OR kids OR boy OR boys OR girl OR girls OR baby OR babies OR teen OR teens OR preteen OR preteens OR teenager OR teenagers OR pubescen* OR prepubescen* OR neonate OR neonates) | 3,331,552 |
| #3 | TS=(antacids OR alginates OR proton pump inhibitors OR ranitidine OR nizatidine OR cimetidine OR famotidine OR lansoprazole OR omeprazole OR esomeprazole OR rabeprazole OR cisapride OR domperidone OR metoclopramide OR erythromycin OR azithromycin OR clarithromycin OR histamine H2 antagonists OR aluminum hydroxide OR sodium bicarbonate OR calcium carbonate OR (alginate* OR gaviscon OR almagate OR almax OR tisacid OR magnesium hydroxide OR brucite OR magnesium hydrate OR "milk of magnesia" OR aluminum hydroxide OR aldrox OR algeldrate OR alhydrogel OR aloh-gel OR alternagel OR alu-cap OR alu-tab OR alugel OR amphojel OR andursil OR brasivil OR dialume OR hydrated alumina OR rocgel OR maalox* OR co-magaldrox OR alucol OR (alumina AND magnesia) OR aluminum hydroxide-magnesium hydroxide OR aluminum magnesium hydroxide OR co-magaldrox OR magnesium aluminum hydroxide OR maldroxal OR mintox OR novalucol OR supralox OR H2 antagonist* OR ranitidin* OR biotidin OR pylorid OR raniberl OR ranisen OR rantec OR sostril OR tritec OR zantac OR cimetidine OR altramet OR biomet OR histodil OR tagamet OR zita OR famotidine OR pepcid OR ym 11170 OR PPI OR PPIs OR proton pump inhibitor* OR lansoprazol* OR dexlansoprazole OR agopton OR lanzoprazol* OR lanzor OR prevacid OR takepron OR zoton OR pantoprazole OR protonix OR pantozol OR pantoloc OR controloc OR omeprazole OR losec OR nexium OR prilosec OR rapinex OR zegerid OR OMEZ OR antra OR mopral OR rabeprazole OR aciphex OR dexrabeprazole OR pariet OR esomeprazol* OR nexium OR nexiam OR metoclopramide OR cerucal OR degan OR gastrobid continus OR gastromax OR maxolon OR maxeran OR metaclopramide OR octamide OR primperan OR reglan OR rimetin OR domperidon* OR motilium OR kw 5338 OR erythromycin OR aknemycin OR T stat OR e-base OR emycin OR emgel OR eryc OR eryderm OR erythro OR erythroped OR gallimycin OR ilosone OR ilotycin OR monomycin OR romycin OR staticin OR stiemycin OR theramycin OR wyamycin OR bethanechol OR bethanecol OR myotonine OR urecholine OR sucralfate OR aluminum sucrose sulfate OR antepsin OR carafate OR sutra OR sulcrate OR ulcogant OR ulsanic OR nizatidine OR cisapride OR azithromycin OR clarithromycin OR prokinetic agent* OR prokinetic* OR hydrotalcite OR asilone OR gastrocote OR topal OR sodium alginate* OR altacite OR sodium bicarbonate) OR (sodium AND alginate*)) | 326,314 |
| #4 | #1 AND #2 AND #3 | 1,662 |
| #5 | #1 AND #2 AND #3 AND Article or Review Article (Document Types) AND English (Languages) AND TS=(systematic review OR meta-analysis OR randomi* control* trial OR RCT OR clinical trial) | 276 |

### PICO 6 - What is the effectiveness of different non-pharmacological treatment options for GER and GERD in infants, children, and adolescents?

| PubMed, 12/05/2024 | | |
| --- | --- | --- |
| Search | Query | Results |
| #1 | gastroesophageal reflux OR gastro esophageal reflux OR gastrooesophageal reflux OR gastro oesophageal reflux OR GER OR GERD OR GOR OR GORD OR (gastric[Title/Abstract] AND (acid[Title/Abstract] OR reflux[Title/Abstract])) OR (reflux[Title/Abstract] AND (oesophagitis[Title/Abstract] OR esophagitis[Title/Abstract])) OR (erosive[Title/Abstract] AND (oesophag*[Title/Abstract] OR esophag*[Title/Abstract])) OR (regurgitation[Title/Abstract] NOT (aortic[Title/Abstract] OR mitral[Title/Abstract] OR tricuspid[Title/Abstract] OR valve[Title/Abstract] OR valvular[Title/Abstract] OR paravalvular[Title/Abstract] OR pulmonary [Title/Abstract])) OR "acid reflux"[Title/Abstract] | 95,575 |
| #2 | child OR children OR pediatric OR pediatrics OR paediatric OR paediatrics OR infant OR infants OR infancy OR newborn OR newborns OR adolescent OR adolescents OR juvenile OR youth OR toddler OR toddlers OR kid OR kids OR boy OR boys OR girl OR girls OR baby OR babies OR teen OR teens OR preteen OR preteens OR teenager OR teenagers OR pubescen* OR prepubescen* OR neonate OR neonates OR (allchild[Filter] OR newborn[Filter] OR allinfant[Filter] OR infant[Filter] OR child[Filter] OR adolescent[Filter] OR preschoolchild[Filter]) | 6,805,330 |
| #3 | "Complementary Therapies"[Mesh] OR "Food"[Mesh] OR "Food Additives"[Mesh] OR "food, formulated"[Mesh] OR "infant formula"[Mesh] OR "Milk"[Mesh] OR "Diet"[Mesh] OR "Posture"[Mesh] OR "dietary supplements"[Mesh] OR "Life Style"[Mesh] OR "weight loss"[Mesh] | 1,421,818 |
| #4 | (non-pharmacologic*[Title/Abstract] OR nonpharmacologic*[Title/Abstract] OR position*[Title/Abstract] OR postur*[Title/Abstract] OR alcohol[Title/Abstract] OR life style*[Title/Abstract] OR lifestyle*[Title/Abstract] OR diet*[Title/Abstract] OR milk*[Title/Abstract] OR soy[Title/Abstract] OR soya[Title/Abstract] OR homeopath*[Title/Abstract] OR massag*[Title/Abstract] OR oil[Title/Abstract] OR oils[Title/Abstract] OR acupunctur*[Title/Abstract] OR hypnother*[Title/Abstract]) | 2,270,978 |
| #5 | (parent*[Title/Abstract] AND (guid*[Title/Abstract] OR support*[Title/Abstract] OR educ*[Title/Abstract] OR teaching[Title/Abstract])) | 136,319 |
| #6 | ((complementary[Title/Abstract] OR alternative[Title/Abstract]) AND (therap*[Title/Abstract] OR treatment*[Title/Abstract] OR option*[Title/Abstract] OR intervention*[Title/Abstract] OR medicin*[Title/Abstract])) | 344,312 |
| #7 | ((food[Title/Abstract] OR feed*[Title/Abstract]) AND (modification*[Title/Abstract] OR advice*[Title/Abstract] OR intervention*[Title/Abstract] OR thicke*[Title/Abstract])) | 102,873 |
| #8 | "probiotics"[Mesh] OR "prebiotics"[Mesh] OR (probiotic*[Title/Abstract] OR prebiotic*[Title/Abstract] OR pro-biotic*[Title/Abstract] OR pre-biotic*[Title/Abstract]) | 56,205 |
| #9 | #3 OR #4 OR #5 OR #6 OR #7 OR #8 | 3,580,341 |
| #10 | #1 AND #2 AND #9 | 4,092 |
| #11 | #10 AND ((clinicalstudy[Filter] OR clinicaltrial[Filter] OR comparativestudy[Filter] OR controlledclinicaltrial[Filter] OR meta-analysis[Filter] OR randomizedcontrolledtrial[Filter] OR systematicreview[Filter]) AND english[Filter]) | 803 |

| Embase, 12/05/2024 | | |
| --- | --- | --- |
| Search | Query | Results |
| #1 | 'gastroesophageal reflux'/exp OR 'gastroesophageal reflux':ti,ab,kw OR 'gastro esophageal reflux':ti,ab,kw OR 'gastrooesophageal reflux':ti,ab,kw OR 'gastro oesophageal reflux':ti,ab,kw OR ger:ti,ab,kw OR gerd:ti,ab,kw OR gor:ti,ab,kw OR gord:ti,ab,kw OR (gastric:ti,ab,kw AND (acid:ti,ab,kw OR reflux:ti,ab,kw)) OR (reflux:ti,ab,kw AND (oesophagitis:ti,ab,kw OR esophagitis:ti,ab,kw)) OR (erosive:ti,ab,kw AND (oesophag*:ti,ab,kw OR esophag*:ti,ab,kw)) OR (regurgitation:ti,ab,kw NOT (aortic:ti,ab,kw OR mitral:ti,ab,kw OR tricuspid:ti,ab,kw OR valve:ti,ab,kw OR valvular:ti,ab,kw OR paravalvular:ti,ab,kw OR pulmonary:ti,ab,kw)) OR 'acid reflux':ti,ab,kw | 143,723 |
| #2 | child:ti,ab,kw OR children:ti,ab,kw OR pediatric:ti,ab,kw OR pediatrics:ti,ab,kw OR paediatric:ti,ab,kw OR paediatrics:ti,ab,kw OR infant:ti,ab,kw OR infants:ti,ab,kw OR infancy:ti,ab,kw OR newborn:ti,ab,kw OR newborns:ti,ab,kw OR adolescent:ti,ab,kw OR adolescents:ti,ab,kw OR juvenile:ti,ab,kw OR youth:ti,ab,kw OR toddler:ti,ab,kw OR toddlers:ti,ab,kw OR kid:ti,ab,kw OR kids:ti,ab,kw OR boy:ti,ab,kw OR boys:ti,ab,kw OR girl:ti,ab,kw OR girls:ti,ab,kw OR baby:ti,ab,kw OR babies:ti,ab,kw OR teen:ti,ab,kw OR teens:ti,ab,kw OR preteen:ti,ab,kw OR preteens:ti,ab,kw OR teenager:ti,ab,kw OR teenagers:ti,ab,kw OR pubescen*:ti,ab,kw OR prepubescen*:ti,ab,kw OR neonate:ti,ab,kw OR neonates:ti,ab,kw | 3,532,866 |
| #3 | 'alternative medicine'/exp OR 'food'/exp OR 'food additive'/exp OR 'elemental diet'/exp OR 'artificial milk'/exp OR 'milk'/exp OR 'diet'/exp OR 'body position'/exp OR 'dietary supplement'/exp OR 'lifestyle'/exp OR 'body weight loss'/exp | 2,283,556 |
| #4 | (non-pharmacologic*:ti,ab,kw OR nonpharmacologic*:ti,ab,kw OR position*:ti,ab,kw OR postur*:ti,ab,kw OR alcohol:ti,ab,kw OR life style*:ti,ab,kw OR lifestyle*:ti,ab,kw OR diet*:ti,ab,kw OR milk*:ti,ab,kw OR soy:ti,ab,kw OR soya:ti,ab,kw OR homeopath*:ti,ab,kw OR massag*:ti,ab,kw OR oil:ti,ab,kw OR oils:ti,ab,kw OR acupunctur*:ti,ab,kw OR hypnother*:ti,ab,kw) | 1,546,146 |
| #5 | (parent*:ti,ab,kw AND (guid*:ti,ab,kw OR support*:ti,ab,kw OR educ*:ti,ab,kw OR teaching:ti,ab,kw)) | 184,834 |
| #6 | ((complementary:ti,ab,kw OR alternative:ti,ab,kw) AND (therap*:ti,ab,kw OR treatment*:ti,ab,kw OR option*:ti,ab,kw OR intervention*:ti,ab,kw OR medicin*:ti,ab,kw)) | 498,345 |
| #7 | ((food:ti,ab,kw OR feed*:ti,ab,kw) AND (modification*:ti,ab,kw OR advice*:ti,ab,kw OR intervention*:ti,ab,kw OR thicke*:ti,ab,kw)) | 142,264 |
| #8 | 'probiotic agent'/exp OR 'prebiotic agent'/exp OR (probiotic*:ti,ab,kw OR prebiotic*:ti,ab,kw OR pro-biotic*:ti,ab,kw OR pre-biotic*:ti,ab,kw) | 78,864 |
| #9 | #3 OR #4 OR #5 OR #6 OR #7 OR #8 | 3,713,840 |
| #10 | #1 AND #2 AND #9 | 4,521 |
| #11 | #10 AND ([article]/lim OR [article in press]/lim OR [review]/lim) AND [english]/lim AND ('clinical trial'/de OR 'clinical trial topic'/de OR 'comparative effectiveness'/de OR 'controlled clinical trial'/de OR 'controlled study'/de OR 'double blind procedure'/de OR 'meta analysis'/de OR 'meta analysis topic'/de OR 'randomized controlled trial'/de OR 'randomized controlled trial topic'/de OR 'systematic review'/de OR 'systematic review topic'/de) | 868 |

| Web of Science, 12/05/2024 | | |
| --- | --- | --- |
| Search | Query | Results |
| #1 | TS=(gastroesophageal reflux OR gastro esophageal reflux OR gastrooesophageal reflux OR gastro oesophageal reflux OR GER OR GERD OR GOR OR GORD OR (gastric AND (acid OR reflux)) OR (reflux AND (oesophagitis OR esophagitis)) OR (erosive AND (oesophag* OR esophag*)) OR (regurgitation NOT (aortic OR mitral OR tricuspid OR valve OR valvular OR paravalvular OR pulmonary )) OR "acid reflux") | 97,816 |
| #2 | TS=(child OR children OR pediatric OR pediatrics OR paediatric OR paediatrics OR infant OR infants OR infancy OR newborn OR newborns OR adolescent OR adolescents OR juvenile OR youth OR toddler OR toddlers OR kid OR kids OR boy OR boys OR girl OR girls OR baby OR babies OR teen OR teens OR preteen OR preteens OR teenager OR teenagers OR pubescen* OR prepubescen* OR neonate OR neonates) | 3,331,552 |
| #3 | TS=(complementary therap* OR food OR infant formula OR milk OR diet OR posture OR dietary supplement* OR life style OR lifestyle OR weight loss) | 2,369,021 |
| #4 | TS=((non-pharmacologic* OR nonpharmacologic* OR position* OR postur* OR alcohol OR life style* OR lifestyle* OR diet* OR milk* OR soy OR soya OR homeopath* OR massag* OR oil OR oils OR acupunctur* OR hypnother*)) | 4,530,943 |
| #5 | TS=((parent* AND (guid* OR support* OR educ* OR teaching))) | 206,073 |
| #6 | TS=(((complementary OR alternative) AND (therap* OR treatment* OR option* OR intervention* OR medicin*))) | 461,633 |
| #7 | TS=(((food OR feed*) AND (modification* OR advice* OR intervention* OR thicke*))) | 157,677 |
| #8 | TS=("probiotics" OR "prebiotics" OR (probiotic* OR prebiotic* OR pro-biotic* OR pre-biotic*)) | 77,645 |
| #9 | #3 OR #4 OR #5 OR #6 OR #7 OR #8 | 6,199,990 |
| #10 | #1 AND #2 AND #9 | 3,228 |
| #11 | #10 AND Article or Review Article (Document Types) AND English (Languages) AND TS=(systematic review OR meta-analysis OR randomi* control* trial OR RCT OR clinical trial OR comparative) | 381 |

### PICO 7 - What are the indications and effectiveness of different surgical and endoscopic treatment options for GERD in infants, children, and adolescents?

| PubMed, 12/05/2024 | | |
| --- | --- | --- |
| Search | Query | Results |
| #1 | gastroesophageal reflux OR gastro esophageal reflux OR gastrooesophageal reflux OR gastro oesophageal reflux OR GER OR GERD OR GOR OR GORD OR (gastric[Title/Abstract] AND (acid[Title/Abstract] OR reflux[Title/Abstract])) OR (reflux[Title/Abstract] AND (oesophagitis[Title/Abstract] OR esophagitis[Title/Abstract])) OR (erosive[Title/Abstract] AND (oesophag*[Title/Abstract] OR esophag*[Title/Abstract])) OR (regurgitation[Title/Abstract] NOT (aortic[Title/Abstract] OR mitral[Title/Abstract] OR tricuspid[Title/Abstract] OR valve[Title/Abstract] OR valvular[Title/Abstract] OR paravalvular[Title/Abstract] OR pulmonary [Title/Abstract])) OR "acid reflux"[Title/Abstract] | 95,575 |
| #2 | child OR children OR pediatric OR pediatrics OR paediatric OR paediatrics OR infant OR infants OR infancy OR newborn OR newborns OR adolescent OR adolescents OR juvenile OR youth OR toddler OR toddlers OR kid OR kids OR boy OR boys OR girl OR girls OR baby OR babies OR teen OR teens OR preteen OR preteens OR teenager OR teenagers OR pubescen* OR prepubescen* OR neonate OR neonates OR (allchild[Filter] OR newborn[Filter] OR allinfant[Filter] OR infant[Filter] OR child[Filter] OR adolescent[Filter] OR preschoolchild[Filter]) | 6,805,330 |
| #3 | (redo-antireflux AND (surgery OR procedure OR procedures OR operation OR operations OR intervention OR interventions OR surgical)) OR ((antireflux OR anti-reflux) AND (surgery OR procedure OR procedures OR operation OR operations OR intervention OR interventions OR surgical)) OR ARS OR (nissen AND (repair OR repairs OR procedure OR procedures OR operation OR operations OR fundoplication OR fundoplications)) OR fundoplication OR (nissen-hill AND hybrid AND repairs) OR (hill AND (repair OR repairs OR procedure OR procedures OR operation OR operations OR fundoplication OR fundoplications)) OR total esophagogastric disconnection OR "Bianchi procedure" OR transpyloric feed*[Title/Abstract] OR jejunal feed*[Title/Abstract] OR duodenal feed*[Title/Abstract] OR radiofrequency ablation[Title/Abstract] OR catheter ablation[Title/Abstract] OR stretta[Title/Abstract] OR endoscopic full thickness plication OR endoluminal endoscopic gastroplication OR ((fundal[Title/Abstract] OR fundus[Title/Abstract] OR fundo[Title/Abstract]) AND plicat*[Title/Abstract]) | 208,438 |
| #4 | #1 AND #2 AND #3 | 2,712 |
| #5 | #1 AND #2 AND #3 AND ((casereports[Filter] OR clinicalstudy[Filter] OR clinicaltrial[Filter] OR comparativestudy[Filter] OR controlledclinicaltrial[Filter] OR meta-analysis[Filter] OR observationalstudy[Filter] OR randomizedcontrolledtrial[Filter] OR systematicreview[Filter]) AND (humans[Filter]) AND (english[Filter])) | 684 |

| Embase, 12/05/2024 | | |
| --- | --- | --- |
| Search | Query | Results |
| #1 | 'gastroesophageal reflux'/exp OR 'gastroesophageal reflux':ti,ab,kw OR 'gastro esophageal reflux':ti,ab,kw OR 'gastrooesophageal reflux':ti,ab,kw OR 'gastro oesophageal reflux':ti,ab,kw OR ger:ti,ab,kw OR gerd:ti,ab,kw OR gor:ti,ab,kw OR gord:ti,ab,kw OR (gastric:ti,ab,kw AND (acid:ti,ab,kw OR reflux:ti,ab,kw)) OR (reflux:ti,ab,kw AND (oesophagitis:ti,ab,kw OR esophagitis:ti,ab,kw)) OR (erosive:ti,ab,kw AND (oesophag*:ti,ab,kw OR esophag*:ti,ab,kw)) OR (regurgitation:ti,ab,kw NOT (aortic:ti,ab,kw OR mitral:ti,ab,kw OR tricuspid:ti,ab,kw OR valve:ti,ab,kw OR valvular:ti,ab,kw OR paravalvular:ti,ab,kw OR pulmonary:ti,ab,kw)) OR 'acid reflux':ti,ab,kw | 143,723 |
| #2 | child:ti,ab,kw OR children:ti,ab,kw OR pediatric:ti,ab,kw OR pediatrics:ti,ab,kw OR paediatric:ti,ab,kw OR paediatrics:ti,ab,kw OR infant:ti,ab,kw OR infants:ti,ab,kw OR infancy:ti,ab,kw OR newborn:ti,ab,kw OR newborns:ti,ab,kw OR adolescent:ti,ab,kw OR adolescents:ti,ab,kw OR juvenile:ti,ab,kw OR youth:ti,ab,kw OR toddler:ti,ab,kw OR toddlers:ti,ab,kw OR kid:ti,ab,kw OR kids:ti,ab,kw OR boy:ti,ab,kw OR boys:ti,ab,kw OR girl:ti,ab,kw OR girls:ti,ab,kw OR baby:ti,ab,kw OR babies:ti,ab,kw OR teen:ti,ab,kw OR teens:ti,ab,kw OR preteen:ti,ab,kw OR preteens:ti,ab,kw OR teenager:ti,ab,kw OR teenagers:ti,ab,kw OR pubescen*:ti,ab,kw OR prepubescen*:ti,ab,kw OR neonate:ti,ab,kw OR neonates:ti,ab,kw | 3,532,866 |
| #3 | 'antireflux operation'/exp OR 'antireflux operation' OR ((antireflux OR anti-reflux) AND (surgery OR procedure OR procedures OR operation OR operations OR intervention OR interventions OR surgical)) OR ARS OR 'Nissen fundoplication'/exp OR (nissen AND (repair OR repairs OR procedure OR procedures OR operation OR operations OR fundoplication OR fundoplications)) OR fundoplication OR (nissen-hill AND hybrid AND repairs) OR (hill AND (repair OR repairs OR procedure OR procedures OR operation OR operations OR fundoplication OR fundoplications)) OR (total esophagogastric disconnection) OR 'bianchi procedure'/exp OR (transpyloric feed*) OR (jejunal feed*) OR (duodenal feed*) OR (radiofrequency ablation) OR (catheter ablation) OR stretta OR (endoscopic full thickness plication) OR (endoluminal endoscopic gastroplication) OR ((fundal OR fundus OR fundo) AND plicat*) | 243,454 |
| #4 | #1 AND #2 AND #3 | 2,475 |
| #5 | #1 AND #2 AND #3 AND ([article]/lim OR [article in press]/lim OR [review]/lim) AND [english]/lim AND [humans]/lim AND ('case report'/de OR 'clinical article'/de OR 'clinical trial'/de OR 'comparative effectiveness'/de OR 'comparative study'/de OR 'controlled clinical trial'/de OR 'controlled study'/de OR 'double blind procedure'/de OR 'meta analysis'/de OR 'meta analysis topic'/de OR 'observational study'/de OR 'randomized controlled trial'/de OR 'randomized controlled trial topic'/de OR 'systematic review'/de OR 'systematic review topic'/de) | 1,061 |

| Web of Science, 12/05/2024 | | |
| --- | --- | --- |
| Search | Query | Results |
| #1 | TS=(gastroesophageal reflux OR gastro esophageal reflux OR gastrooesophageal reflux OR gastro oesophageal reflux OR GER OR GERD OR GOR OR GORD OR (gastric AND (acid OR reflux)) OR (reflux AND (oesophagitis OR esophagitis)) OR (erosive AND (oesophag* OR esophag*)) OR (regurgitation NOT (aortic OR mitral OR tricuspid OR valve OR valvular OR paravalvular OR pulmonary )) OR "acid reflux") | 98,356 |
| #2 | TS=(child OR children OR pediatric OR pediatrics OR paediatric OR paediatrics OR infant OR infants OR infancy OR newborn OR newborns OR adolescent OR adolescents OR juvenile OR youth OR toddler OR toddlers OR kid OR kids OR boy OR boys OR girl OR girls OR baby OR babies OR teen OR teens OR preteen OR preteens OR teenager OR teenagers OR pubescen* OR prepubescen* OR neonate OR neonates) | 3,338,582 |
| #3 | TS=((redo-antireflux AND (surgery OR procedure OR procedures OR operation OR operations OR intervention OR interventions OR surgical)) OR ((antireflux OR anti-reflux) AND (surgery OR procedure OR procedures OR operation OR operations OR intervention OR interventions OR surgical)) OR ARS OR (nissen AND (repair OR repairs OR procedure OR procedures OR operation OR operations OR fundoplication OR fundoplications)) OR fundoplication OR (nissen-hill AND hybrid AND repairs) OR (hill AND (repair OR repairs OR procedure OR procedures OR operation OR operations OR fundoplication OR fundoplications)) OR (total esophagogastric disconnection) OR "Bianchi procedure" OR (transpyloric feed*) OR (jejunal feed*) OR (duodenal feed*) OR (radiofrequency ablation) OR (catheter ablation) OR stretta OR (endoscopic full thickness plication) OR (endoluminal endoscopic gastroplication) OR ((fundal OR fundus OR fundo) AND plicat*)) | 102,945 |
| #4 | #1 AND #2 AND #3 | 1,344 |
| #5 | #1 AND #2 AND #3 AND Article or Review Article (Document Types) AND English (Languages) AND TS=(systematic review OR meta-analysis OR randomi* control* trial OR RCT OR clinical trial OR comparative OR observational OR cohort OR case series) | 277 |

### PICO 8 – What is the prognosis of GER and GERD in infants, children, and adolescents, and what are the prognostic factors?

| PubMed, 12/05/2024 | | |
| --- | --- | --- |
| Search | Query | Results |
| #1 | gastroesophageal reflux OR gastro esophageal reflux OR gastrooesophageal reflux OR gastro oesophageal reflux OR GER OR GERD OR GOR OR GORD OR (gastric[Title/Abstract] AND (acid[Title/Abstract] OR reflux[Title/Abstract])) OR (reflux[Title/Abstract] AND (oesophagitis[Title/Abstract] OR esophagitis[Title/Abstract])) OR (erosive[Title/Abstract] AND (oesophag*[Title/Abstract] OR esophag*[Title/Abstract])) OR (regurgitation[Title/Abstract] NOT (aortic[Title/Abstract] OR mitral[Title/Abstract] OR tricuspid[Title/Abstract] OR valve[Title/Abstract] OR valvular[Title/Abstract] OR paravalvular[Title/Abstract] OR pulmonary [Title/Abstract])) OR "acid reflux"[Title/Abstract] | 95,575 |
| #2 | child OR children OR pediatric OR pediatrics OR paediatric OR paediatrics OR infant OR infants OR infancy OR newborn OR newborns OR adolescent OR adolescents OR juvenile OR youth OR toddler OR toddlers OR kid OR kids OR boy OR boys OR girl OR girls OR baby OR babies OR teen OR teens OR preteen OR preteens OR teenager OR teenagers OR pubescen* OR prepubescen* OR neonate OR neonates OR (allchild[Filter] OR newborn[Filter] OR allinfant[Filter] OR infant[Filter] OR child[Filter] OR adolescent[Filter] OR preschoolchild[Filter]) | 6,805,345 |
| #3 | (prognosis OR prognoses[Text Word] OR prognostic factors[Text Word] OR prognostic factor[Text Word] OR "disease progression"[Text Word] OR "survival analysis"[Text Word]) OR ((natural AND (course OR history OR evolution OR evolve OR progress OR progression))) | 2,939,633 |
| #4 | #1 AND #2 AND #3 | 3,243 |
| #5 | #1 AND #2 AND #3 AND ((casereports[Filter] OR clinicalstudy[Filter] OR clinicaltrial[Filter] OR comparativestudy[Filter] OR controlledclinicaltrial[Filter] OR meta-analysis[Filter] OR observationalstudy[Filter] OR randomizedcontrolledtrial[Filter] OR systematicreview[Filter]) AND (humans[Filter]) AND (english[Filter])) | 1,003 |

| Embase, 12/05/2024 | | |
| --- | --- | --- |
| Search | Query | Results |
| #1 | 'gastroesophageal reflux'/exp OR 'gastroesophageal reflux':ti,ab,kw OR 'gastro esophageal reflux':ti,ab,kw OR 'gastrooesophageal reflux':ti,ab,kw OR 'gastro oesophageal reflux':ti,ab,kw OR ger:ti,ab,kw OR gerd:ti,ab,kw OR gor:ti,ab,kw OR gord:ti,ab,kw OR (gastric:ti,ab,kw AND (acid:ti,ab,kw OR reflux:ti,ab,kw)) OR (reflux:ti,ab,kw AND (oesophagitis:ti,ab,kw OR esophagitis:ti,ab,kw)) OR (erosive:ti,ab,kw AND (oesophag*:ti,ab,kw OR esophag*:ti,ab,kw)) OR (regurgitation:ti,ab,kw NOT (aortic:ti,ab,kw OR mitral:ti,ab,kw OR tricuspid:ti,ab,kw OR valve:ti,ab,kw OR valvular:ti,ab,kw OR paravalvular:ti,ab,kw OR pulmonary:ti,ab,kw)) OR 'acid reflux':ti,ab,kw | 143,723 |
| #2 | child:ti,ab,kw OR children:ti,ab,kw OR pediatric:ti,ab,kw OR pediatrics:ti,ab,kw OR paediatric:ti,ab,kw OR paediatrics:ti,ab,kw OR infant:ti,ab,kw OR infants:ti,ab,kw OR infancy:ti,ab,kw OR newborn:ti,ab,kw OR newborns:ti,ab,kw OR adolescent:ti,ab,kw OR adolescents:ti,ab,kw OR juvenile:ti,ab,kw OR youth:ti,ab,kw OR toddler:ti,ab,kw OR toddlers:ti,ab,kw OR kid:ti,ab,kw OR kids:ti,ab,kw OR boy:ti,ab,kw OR boys:ti,ab,kw OR girl:ti,ab,kw OR girls:ti,ab,kw OR baby:ti,ab,kw OR babies:ti,ab,kw OR teen:ti,ab,kw OR teens:ti,ab,kw OR preteen:ti,ab,kw OR preteens:ti,ab,kw OR teenager:ti,ab,kw OR teenagers:ti,ab,kw OR pubescen*:ti,ab,kw OR prepubescen*:ti,ab,kw OR neonate:ti,ab,kw OR neonates:ti,ab,kw | 3,532,866 |
| #3 | 'prognosis'/exp OR prognosis:ti,ab,kw OR prognoses:ti,ab,kw OR 'prognostic factors':ti,ab,kw OR 'prognostic factor':ti,ab,kw OR 'disease progression':ti,ab,kw OR 'disease exacerbation'/exp OR 'survival analysis':ti,ab,kw OR 'survival analysis'/exp OR (natural AND (course OR history OR evolution OR evolve OR progress OR progression)):ti,ab,kw | 1,902,238 |
| #4 | #1 AND #2 AND #3 | 973 |
| #5 | #1 AND #2 AND #3 AND ([article]/lim OR [article in press]/lim OR [review]/lim) AND [english]/lim AND [humans]/lim | 644 |

| Web of Science, 12/05/2024 | | |
| --- | --- | --- |
| Search | Query | Results |
| #1 | TS=(gastroesophageal reflux OR gastro esophageal reflux OR gastrooesophageal reflux OR gastro oesophageal reflux OR GER OR GERD OR GOR OR GORD OR (gastric AND (acid OR reflux)) OR (reflux AND (oesophagitis OR esophagitis)) OR (erosive AND (oesophag* OR esophag*)) OR (regurgitation NOT (aortic OR mitral OR tricuspid OR valve OR valvular OR paravalvular OR pulmonary )) OR "acid reflux") | 97,249 |
| #2 | TS=(child OR children OR pediatric OR pediatrics OR paediatric OR paediatrics OR infant OR infants OR infancy OR newborn OR newborns OR adolescent OR adolescents OR juvenile OR youth OR toddler OR toddlers OR kid OR kids OR boy OR boys OR girl OR girls OR baby OR babies OR teen OR teens OR preteen OR preteens OR teenager OR teenagers OR pubescen* OR prepubescen* OR neonate OR neonates) | 3,324,516 |
| #3 | TS=((prognosis OR prognoses OR prognostic factors OR prognostic factor OR disease progression OR survival analysis) OR (natural AND (course OR history OR evolution OR evolve OR progress OR progression))) | 1,747,295 |
| #4 | #1 AND #2 AND #3 | 497 |
| #5 | #1 AND #2 AND #3 and Article or Review Article (Document Types) and English (Languages) | 457 |

## PRISMA Flow diagrams

### Search strategy to identify guidelines and consensus statements on gastroesophageal reflux (GER) and gastroesophageal reflux disease (GERD) in infants, children, and adolescents

Records identified from:

Databases (n = 300)

- PubMed (n = 97)
- Embase (n = 110)
- Web of Science (n = 93)

Google Scholar (n = 50)

Records removed *before screening*:

Duplicate records removed (n = 133)

Records screened

(n = 217)

Records excluded

(n = 187)

Reports sought for retrieval

(n = 30)

Reports not retrieved

(n = 0)

Reports assessed for eligibility

(n = 30)

Reports excluded (n = 27):

- Wrong population (n = 2)
- Wrong study design (n= 22)
- Duplicates (n = 3)

Studies included in review

(n = 3)

Reports of included studies

(n = 3)

**Identification of studies via databases and registers**

**Identification**

**Screening**

**Included**

### PICO 5 - What is the evidence for the effectiveness of pharmacological treatments for GER and GERD in infants, children, and adolescents?

Records identified from:

Databases (n = 1,914)

- PubMed (n = 779)
- Embase (n = 859)
- Web of Science (n = 276)

Registers (n = 0)
Additional records identified through reference list (n=1)

Records removed *before screening*:

Duplicate records removed (n = 262)

Records screened

(n = 1,653)

Total records excluded (n = 1,542; including 84 duplicates)

Reports sought for retrieval

(n = 111)

Reports not retrieved (n = 1)

Reports assessed for eligibility

(n = 110)

Reports excluded (n = 65)

- Wrong population (n = 33)
- Wrong intervention (n = 1)
- Wrong comparator (n = 1)
- Wrong study design (n = 7)
- Systematic review including studies already in our selection or not meeting inclusion criteria (n = 19)
- Duplicate reports (n = 4)

Studies included in review

(n = 45)

Reports of included studies

(n = 45)

**Identification of studies via databases and registers**

**Identification**

**Screening**

**Included**

### PICO 6 - What is the effectiveness of different non-pharmacological treatment options for GER and GERD in infants, children, and adolescents?

Records identified from:

Databases (n = 2,052)

- PubMed (n = 803)
- Embase (n = 868)
- Web of Science (n = 381)

Registers (n = 0)

Records identified from reference lists (n = 6)

Records removed *before screening*:

Duplicate records removed (n = 297)

Records screened

(n = 1,761)

Total records excluded (n = 1,698; including 77 duplicates)

Reports sought for retrieval

(n = 63)

Reports not retrieved

(n = 0)

Reports assessed for eligibility

(n = 63)

Reports excluded (n = 23)

- Wrong population (n = 5)
- Wrong intervention (n = 3)
- Wrong comparator (n = 2)
- Wrong outcome (n = 1)
- Wrong study design (n = 5)
- Systematic review including studies already in our selection or not meeting inclusion criteria (n = 7)

Studies included in review

(n = 40)

Reports of included studies

(n = 40)

**Identification of studies via databases and registers**

**Identification**

**Screening**

**Included**

### PICO 7 - What are the indications and effectiveness of different surgical and endoscopic treatment options for GERD in infants, children, and adolescents?

Records identified from:

Databases (n = 2,022)

- PubMed (n = 684)
- Embase (n = 1,061)
- Web of Science (n = 277)

Registers (n = 0)

Records removed *before screening*:

Duplicate records removed (n = 309)

Records screened

(n = 1,713)

Records excluded

(n = 1,683; including 77 duplicates)

Reports sought for retrieval

(n = 30)

Reports not retrieved

(n = 0)

Reports assessed for eligibility

(n = 30)

Reports excluded (n = 20)

- Wrong population (n = 10)
- Wrong outcome (n = 2)
- Wrong study design (n = 7)
- Systematic review including studies already in our selection or not meeting inclusion criteria (n=1)

Studies included in review

(n = 5)

Reports of included studies

(n = 10)

**Identification of studies via databases and registers**

**Identification**

**Screening**

**Included**

### PICO 8 – What is the prognosis of GER and GERD in infants, children, and adolescents, and what are the prognostic factors?

Records identified from:

Databases (n = 2,104)

- PubMed (n = 1,003)
- Embase (n = 644)
- Web of Science (n = 457)

Registers (n = 0)

Records removed *before screening*:

Duplicate records removed (n = 189)

Records screened

(n = 1,915)

Records excluded

(n = 1,894; including 58 duplicates)

Reports sought for retrieval

(n = 20)

Reports not retrieved

(n = 2)

Reports assessed for eligibility

(n = 18)

Reports excluded (n = 14)

- Wrong study design (n = 8)
- Wrong population (n = 3)
- Wrong outcome (n = 3)

Studies included in review

(n = 4)

Reports of included studies

(n = 4)

**Identification of studies via databases and registers**

**Identification**

**Screening**

**Included**

## Excluded studies

### Search strategy to identify guidelines and consensus statements on gastroesophageal reflux (GER) and gastroesophageal reflux disease (GERD) in infants, children, and adolescents

| Study | Reason for exclusion |
| --- | --- |
| (2003). "IPEG guidelines for surgical treatment of pediatric gastroesophageal reflux disease (GERD)." Pediatric Endosurgery and Innovative Techniques 7(2): 210-213. | Wrong study design |
| (2008). "IPEG guidelines for the surgical treatment of pediatric gastroesophageal reflux disease (GERD)." J Laparoendosc Adv Surg Tech A 18(6): x-xiii. | Wrong study design |
| Ayerbe, J. I. G., et al. (2019). "Diagnosis and Management of Gastroesophageal Reflux Disease in Infants and Children: from Guidelines to Clinical Practice." Pediatric Gastroenterology Hepatology & Nutrition 22(2): 107-121. | Wrong study design |
| Barfield, E. and M. W. Parker (2019). "Management of Pediatric Gastroesophageal Reflux Disease." JAMA Pediatr 173(5): 485-486. | Wrong study design |
| Butler, N. (2009). "National Guidelines at a glance: GORD." SA Pharmaceutical Journal 76(9): 32-36. | Wrong study design |
| Chang, A. B., et al. (2019). "Chronic Cough and Gastroesophageal Reflux in Children: CHEST Guideline and Expert Panel Report." Chest 156(1): 131-140. | Wrong population |
| Davies, I., et al. (2015). "Gastro-oesophageal reflux disease in children: NICE guidance." Bmj 350: g7703. | Wrong study design |
| Daza, W., et al. (2017). "Methodological quality of clinical practice guidelines to management and/or treatment gastroesophageal reflux disease in paediatrics." J Pediatr Gastroenterol Nutr 64: 362. | Wrong study design |
| Fuchs, K. H., et al. (2014). "EAES recommendations for the management of gastroesophageal reflux disease." Surg Endosc 28(6): 1753-1773. | Wrong population |
| Gold, B. and P. Sherman (2008). "A global, evidence-based consensus on the definition of pediatric gastroesophageal reflux disease (GERD)." American Journal of Gastroenterology 103: S36-S36. | Wrong study design |
| Kane, T. D., et al. (2009). "Position paper on laparoscopic antireflux operations in infants and children for gastroesophageal reflux disease. American Pediatric Surgery Association." J Pediatr Surg 44(5): 1034-1040. | Wrong study design |
| Lightdale 2013. Gastroesophageal Reflux: Management Guidance for the Pediatrician. www.pediatrics.org/cgi/doi/10.1542/peds.2013-0421 doi:10.1542/peds.2013-0421 | Wrong study design |
| Lopez, R. N. and D. A. Lemberg (2020). "Gastro-oesophageal reflux disease in infancy: a review based on international guidelines." Med J Aust 212(1): 40-44. | Wrong study design |
| Mohan, N., et al. (2021). "Diagnosis and Management of Gastroesophageal Reflux Disease in Children: Recommendations of Pediatric Gastroenterology Chapter of Indian Academy of Pediatrics, Indian Society of Pediatric Gastroenterology, Hepatology and Nutrition (ISPGHAN)." Indian Pediatr 58(12): 1163-1170. | Wrong study design |
| Papachrisanthou, M. M. and R. L. Davis (2015). "Clinical Practice Guidelines for the Management of Gastroesophageal Reflux and Gastroesophageal Reflux Disease: Birth to 1 Year of Age." J Pediatr Health Care 29(6): 558-564. | Wrong study design |
| Papachrisanthou, M. M. and R. L. Davis (2016). "Clinical Practice Guidelines for the Management of Gastroesophageal Reflux and Gastroesophageal Reflux Disease: 1 Year to 18 Years of Age." J Pediatr Health Care 30(3): 289-294. | Wrong study design |
| Randel, A. (2014). "AAP releases guideline for the management of gastroesophageal reflux in children." Am Fam Physician 89(5): 395-397. | Wrong study design |
| Rerksuppaphol, S. and G. Barnes (2002). "Guidelines for evaluation and treatment of gastroesophageal reflux in infants and children: recommendations of the North American Society for Pediatric Gastroenterology and Nutrition." J Pediatr Gastroenterol Nutr 35(4): 583. | Wrong study design |
| Rodgers, A. (2021). "Gastro-oesophageal reflux in preterm infants: American Academy of Pediatrics guideline 2018." Arch Dis Child Educ Pract Ed 106(2): 107. | Wrong study design |
| Rudolph, C. D., et al. (2001). "Guidelines for evaluation and treatment of gastroesophageal reflux in infants and children: Recommendations of the North America Society for Pediatric Gastroenterology and Nutrition." J Pediatr Gastroenterol Nutr 32(SUPPL. 2): S1-S31. | Wrong study design |
| Sherman, P. (2009). "A global, evidence-based consensus on the definition of gastroesophageal reflux disease (GERD) in the pediatric population." Canadian Journal of Gastroenterology 23. | Wrong study design |
| Sherman, P. M., et al. (2009). "A Global, Evidence-Based Consensus on the Definition of Gastroesophageal Reflux Disease in the Pediatric Population." American Journal of Gastroenterology 104(5): 1278-1295. | Wrong study design |
| Vandenplas, Y., et al. (2023). "Infant gastroesophageal reflux disease management consensus." Acta Paediatrica. | Wrong study design |
| Vandenplas, Y., et al. (2009). "Pediatric gastroesophageal reflux clinical practice guidelines: Joint recommendations of the North American Society for Pediatric Gastroenterology, Hepatology, and Nutrition (NASPGHAN) and the European Society for Pediatric Gastroenterology, Hepatology, and Nutrition (ESPGHAN)." J Pediatr Gastroenterol Nutr 49(4): 498-547. | Wrong study design |
| (2009). "IPEG guidelines for the surgical treatment of pediatric gastroesophageal reflux disease (GERD)." J Laparoendosc Adv Surg Tech A 19 Suppl 1: x-xiii. | Duplicate |
| (2018). 2018 surveillance of gastro-oesophageal reflux disease in children and young people: diagnosis and management (NICE guideline NG1). London, National Institute for Health and Care Excellence (NICE). Copyright © NICE 2018. | duplicate |
| Gonzalez Ayerbe, J. I., et al. (2019). "Diagnosis and Management of Gastroesophageal Reflux Disease in Infants and Children: from Guidelines to Clinical Practice." Pediatr Gastroenterol Hepatol Nutr 22(2): 107-121. | Duplicate |

### PICO 5 - What is the evidence for the effectiveness of pharmacological treatments for GER and GERD in infants, children, and adolescents?

| Study | Reason for exclusion |
| --- | --- |
| (2006). "GERD therapy minimally effective for chronic cough." Journal of Family Practice 55(4): 288. | Wrong study design |
| Angelini, G., et al. (1990). "Treatment of reflux gastritis: double blind comparison between clebopride and domperidone. A preliminary report." Ital J Gastroenterol 22(1): 24-27. | Wrong population |
| Annibale, B., et al. (1998). "Omeprazole in patients with mild or moderate reflux esophagitis induces lower relapse rates than ranitidine during maintenance treatment." Hepatogastroenterology 45(21): 742-751. | Wrong population |
| Augood, C., et al. (2003). "Cisapride treatment for gastro-oesophageal reflux in children." Cochrane Database Syst Rev(4): Cd002300. | Systematic review including studies already in our selection or not meeting inclusion criteria |
| Bardhan, K. D., et al. (1994). "Rapid healing of gastric ulcers with lansoprazole." Aliment Pharmacol Ther 8(2): 215-220. | Wrong population |
| Bate, C. M., et al. (1993). "Does 40 mg omeprazole daily offer additional benefit over 20 mg daily in patients requiring more than 4 weeks of treatment for symptomatic reflux oesophagitis?" Aliment Pharmacol Ther 7(5): 501-507. | Wrong population |
| Bowrey, D. J., et al. (2000). "Gastroesophageal reflux disease in asthma. Effects of medical and surgical antireflux therapy on asthma control." Ann Surg 231(2): 161-172. | Wrong study design |
| Brun, J. and H. Sörngård (2000). "High dose proton pump inhibitor response as an initial strategy for a clinical diagnosis of gastro-oesophageal reflux disease (GERD). Swedish multi-centre group in primary health care." Fam Pract 17(5): 401-404. | Wrong population |
| Caos, A., et al. (2005). "Long-term prevention of erosive or ulcerative gastro-oesophageal reflux disease relapse with rabeprazole 10 or 20 mg vs. placebo: results of a 5-year study in the United States." Aliment Pharmacol Ther 22(3): 193-202. | Wrong population |
| Carling, L., et al. (1988). "Sucralfate versus placebo in reflux esophagitis. A double-blind multicenter study." Scand J Gastroenterol 23(9): 1117-1124. | Wrong population |
| Carlsson, R., et al. (1998). "Gastro-oesophageal reflux disease in primary care: an international study of different treatment strategies with omeprazole. International GORD Study Group." Eur J Gastroenterol Hepatol 10(2): 119-124. | Wrong population |
| Castell, D., et al. (2005). "Comparison of the effects of immediate-release omeprazole powder for oral suspension and pantoprazole delayed-release tablets on nocturnal acid breakthrough in patients with symptomatic gastro-oesophageal reflux disease." Aliment Pharmacol Ther 21(12): 1467-1474. | Wrong population |
| Chandan, S., et al. (2023). "Vonoprazan versus lansoprazole in erosive esophagitis - A systematic review and meta-analysis of randomized controlled trials." Indian J Gastroenterol 42(4): 475-484. | Wrong population |
| Chandra, M. A. and I. S. Gandhi (1989). "Comparative study of Riflux Forte and Riflux in cases of reflux oesophagitis." Br J Clin Pract 43(4): 144-147. | Wrong population |
| Chang, A. B., et al. (2011). "Gastro-oesophageal reflux treatment for prolonged non-specific cough in children and adults." Cochrane Database Syst Rev 2011(1): Cd004823. | Systematic review including studies already in our selection or not meeting inclusion criteria |
| Chang, A. B., et al. (2006). "Systematic review and meta-analysis of randomised controlled trials of gastro-oesophageal reflux interventions for chronic cough associated with gastro-oesophageal reflux." Bmj 332(7532): 11-17. | Systematic review including studies already in our selection or not meeting inclusion criteria |
| Chicella, M. F., et al. (2005). "Prokinetic drug therapy in children: A review of current options." Annals of Pharmacotherapy 39(4): 706-711. | Systematic review including studies already in our selection or not meeting inclusion criteria |
| Cibor, D., et al. (2006). "Optimal maintenance therapy in patients with non-erosive reflux disease reporting mild reflux symptoms--a pilot study." Adv Med Sci 51: 336-339. | Wrong population |
| Corazziari, E. S., et al. (2023). "Poliprotect vs Omeprazole in the Relief of Heartburn, Epigastric Pain, and Burning in Patients Without Erosive Esophagitis and Gastroduodenal Lesions: A Randomized, Controlled Trial." American Journal of Gastroenterology 118(11): 2014-2024. | Wrong population |
| Cucchiara, S., et al. (1990). "Effects of cisapride on parameters of oesophageal motility and on the prolonged intraoesophageal pH test in infants with gastro-oesophageal reflux disease." Gut 31(1): 21-25. | Wrong study design |
| Dakkak, M., et al. (1994). "Comparing the efficacy of cisapride and ranitidine in oesophagitis: a double-blind, parallel group study in general practice." Br J Clin Pract 48(1): 10-14. | Wrong population |
| de Mattos, Â. Z., et al. (2017). "Antisecretory treatment for pediatric gastroesophageal reflux disease – A systematic review." Arq Gastroenterol 54(4): 271-280. | Systematic review including studies already in our selection or not meeting inclusion criteria |
| Dermyshi, E., et al. (2018). "Antacid therapy for gastroesophageal reflux in preterm infants: a systematic review." BMJ Paediatr Open 2(1): e000287. | Systematic review including studies already in our selection or not meeting inclusion criteria |
| Dewan, K. and J. Lieu (2018). "A Clinical Trial of Proton Pump Inhibitors to Treat Children with Chronic Otitis Media with Effusion." J Int Adv Otol 14(2): 245-249. | Wrong population |
| Dossett, M. L., et al. (2015). "Patient-Provider Interactions Affect Symptoms in Gastroesophageal Reflux Disease: A Pilot Randomized, Double-Blind, Placebo-Controlled Trial." PLoS One 10(9): e0136855. | Wrong population |
| Eggleston, A., et al. (2009). "Clinical trial: the treatment of gastro-oesophageal reflux disease in primary care--prospective randomized comparison of rabeprazole 20 mg with esomeprazole 20 and 40 mg." Aliment Pharmacol Ther 29(9): 967-978. | Wrong population |
| Famouri, F., et al. (2017). "Comparison of hypoallergenic diet vs. ranitidine in treatment of gastroesophageal reflux disease of infants: A randomized clinical trial." Iranian Journal of Pediatrics 27(4). | Wrong comparator |
| Fernández-González, S. M., et al. (2024). "Proton Pump Inhibitors in Pediatric Gastroesophageal Reflux Disease: A Systematic Review of Randomized Controlled Trials." Children-Basel 11(3). | Systematic review including studies already in our selection or not meeting inclusion criteria |
| Fogleman, C. D. (2011). "GERD treatment for chronic nonspecific cough in children and adults." Am Fam Physician 84(5): 502-504. | Wrong study design |
| Gardner, J. D., et al. (2003). "Heartburn severity can predict pathologic oesophageal reflux in gastro-oesophageal reflux disease patients treated with a proton-pump inhibitor." Aliment Pharmacol Ther 18(1): 133-140. | Wrong population |
| Ghimire, J. J., et al. (2022). "Azithromycin for Poorly Controlled Asthma in Children: A Randomized Controlled Trial." Chest 161(6): 1456-1464. | Wrong population |
| Gibbons, T. E. and B. D. Gold (2003). "The use of proton pump inhibitors in children: A comprehensive review." Pediatric Drugs 5(1): 25-40. | Wrong study design |
| Gibson, P. G., et al. (2003). "Gastro-oesophageal reflux treatment for asthma in adults and children." Cochrane Database Syst Rev(2): Cd001496. | Systematic review including studies already in our selection or not meeting inclusion criteria |
| Gieruszczak-Bialek, D., et al. (2015). "No Effect of Proton Pump Inhibitors on Crying and Irritability in Infants: Systematic Review of Randomized Controlled Trials." Journal of Pediatrics 166(3): 767-U1086. | Systematic review including studies already in our selection or not meeting inclusion criteria |
| Gilbert, R. E., et al. (2000). "Cisapride treatment for gastro-oesoghageal reflux in children: A systematic review of randomized controlled trials." J Paediatr Child Health 36(6): 524-529. | Systematic review including studies already in our selection or not meeting inclusion criteria |
| Higginbotham, T. W. (2010). "Effectiveness and safety of proton pump inhibitors in infantile gastroesophageal reflux disease." Annals of Pharmacotherapy 44(3): 572-576. | Wrong study design |
| Holbrook, J. T., et al. (2012). "Lansoprazole for children with poorly controlled asthma: a randomized controlled trial." Jama 307(4): 373-381. | Wrong population |
| Howden, C. W., et al. (2009). "Control of 24-hour intragastric acidity with morning dosing of immediate-release and delayed-release proton pump inhibitors in patients with GERD." J Clin Gastroenterol 43(4): 323-326. | Wrong population |
| Illueca, M., et al. (2010). "Maintenance Treatment With Proton Pump Inhibitors for Reflux Esophagitis in Pediatric Patients: A Systematic Literature Analysis." J Pediatr Gastroenterol Nutr 51(6): 733-740. | Systematic review including studies already in our selection or not meeting inclusion criteria |
| Jørgensen, F. and L. Elsborg (1991). "Sucralfate versus cimetidine in reflux oesophagitis. The effect on oesophageal pH and motility." Scand J Gastroenterol 26(3): 263-268. | Wrong population |
| Jørgensen, F. and L. Elsborg (1991). "Sucralfate versus cimetidine in the treatment of reflux esophagitis, with special reference to the esophageal motor function." Am J Med 91(2a): 114s-118s. | Wrong population |
| Kaspari, S., et al. (2005). "Pantoprazole 20 mg on demand is effective in the long-term management of patients with mild gastro-oesophageal reflux disease." Eur J Gastroenterol Hepatol 17(9): 935-941. | Wrong population |
| Khorasani, E. N., et al. (2008). "The effect of omeprazole on asthmatic adolescents with gastroesophageal reflux disease." Allergy Asthma Proc 29(5): 517-520. | Duplicate |
| Kopsaftis, Z., et al. (2021). "Pharmacological and surgical interventions for the treatment of gastro-oesophageal reflux in adults and children with asthma." Cochrane Database Syst Rev 5(5): Cd001496. | Systematic review including studies already in our selection or not meeting inclusion criteria |
| Kumar, Y. and R. Sarvananthan (2008). "GORD in children." BMJ Clin Evid 2008. | Systematic review including studies already in our selection or not meeting inclusion criteria |
| Lin, M. L. M., et al. (2016). "The Safety of Metoclopramide in Children: A Systematic Review and Meta-Analysis." Drug Saf 39(7): 675-687. | Systematic review including studies already in our selection or not meeting inclusion criteria |
| Loots, C. M., et al. (2012). "Esophageal impedance baselines in infants before and after placebo and proton pump inhibitor therapy." Neurogastroenterol Motil 24(8): 758-+. | Wrong study design |
| Majewski, M., et al. (2016). "Gastric pH and Therapeutic Responses to Exsomeprazole in Patients With Functional Dyspepsia: Potential Clinical Implications." Am J Med Sci 352(6): 582-592. | Wrong population |
| Mattos Â, Z., et al. (2017). "ANTISECRETORY TREATMENT FOR PEDIATRIC GASTROESOPHAGEAL REFLUX DISEASE - A SYSTEMATIC REVIEW." Arq Gastroenterol 54(4): 271-280. | Duplicate |
| Mee, A. S. and J. L. Rowley (1996). "Rapid symptom relief in reflux oesophagitis: a comparison of lansoprazole and omeprazole." Aliment Pharmacol Ther 10(5): 757-763. | Wrong population |
| Miceli Sopo, S., et al. (2009). "Does treatment with proton pump inhibitors for gastroesophageal reflux disease (GERD) improve asthma symptoms in children with asthma and GERD? A systematic review." Journal of Investigational Allergology and Clinical Immunology 19(1): 1-5. | Systematic review including studies already in our selection or not meeting inclusion criteria |
| Miner, P., et al. (2010). "Effects of a single dose of rabeprazole 20 mg and pantoprazole 40 mg on 24-h intragastric acidity and oesophageal acid exposure: a randomized study in gastro-oesophageal reflux disease patients with a history of nocturnal heartburn." Aliment Pharmacol Ther 31(9): 991-1000. | Wrong population |
| Moraes-Filho, J. P., et al. (2014). "Randomised clinical trial: daily pantoprazole magnesium 40 mg vs. esomeprazole 40 mg for gastro-oesophageal reflux disease, assessed by endoscopy and symptoms." Aliment Pharmacol Ther 39(1): 47-56. | Wrong population |
| Oderda, G., et al. (1990). "Treatment of childhood peptic oesophagitis with famotidine or alginate-antacid." Ital J Gastroenterol 22(6): 346-349. | Wrong intervention |
| Pritchard, D. S., et al. (2005). "Should domperidone be used for the treatment of gastro-oesophageal reflux in children? Systematic review of randomized controlled trials in children aged 1 month to 11 years old." Br J Clin Pharmacol 59(6): 725-729. | Systematic review including studies already in our selection or not meeting inclusion criteria |
| Schmitt, C., et al. (2006). "A multicenter, randomized, double-blind, 8-week comparative trial of standard doses of esomeprazole (40 mg) and omeprazole (20 mg) for the treatment of erosive esophagitis." Dig Dis Sci 51(5): 844-850. | Wrong population |
| Sharma, P., et al. (2009). "Clinical trials: healing of erosive oesophagitis with dexlansoprazole MR, a proton pump inhibitor with a novel dual delayed-release formulation--results from two randomized controlled studies." Aliment Pharmacol Ther 29(7): 731-741. | Wrong population |
| Simon, T. J., et al. (1995). "Acid suppression by famotidine 20 mg twice daily or 40 mg twice daily in preventing relapse of endoscopic recurrence of erosive esophagitis." Clin Ther 17(6): 1147-1156. | Wrong population |
| Sopo, S. M., et al. (2009). "Does treatment with proton pump inhibitors for gastroesophageal reflux disease (GERD) improve asthma symptoms in children with asthma and GERD? A systematic review." J Investig Allergol Clin Immunol 19(1): 1-5. | Duplicate |
| Tan, V. P., et al. (2011). "Treatment of non-erosive reflux disease with a proton pump inhibitor in Chinese patients: a randomized controlled trial." J Gastroenterol 46(7): 906-912. | Wrong population |
| Van Der Pol, R., et al. (2014). "Efficacy and safety of histamine-2 receptor antagonists." JAMA Pediatrics 168(10): 947-954. | Systematic review including studies already in our selection or not meeting inclusion criteria |
| Van Der Pol, R. J., et al. (2011). "Efficacy of proton-pump inhibitors in children with gastroesophageal reflux disease: A systematic review." Pediatrics 127(5): 925-935. | Systematic review including studies already in our selection or not meeting inclusion criteria |
| Winter, H., et al. (2015). "Esomeprazole for the Treatment of GERD in Infants Ages 1-11 Months." J Pediatr Gastroenterol Nutr 60 Suppl 1: S9-15. | Duplicate |
| Zheng, Z. D., et al. (2021). "Randomised trials of proton pump inhibitors for gastro-oesophageal reflux disease in patients with asthma: an updated systematic review and meta-analysis." BMJ Open 11(8). | Systematic review including studies already in our selection or not meeting inclusion criteria |

### PICO 6 - What is the effectiveness of different non-pharmacological treatment options for GER and GERD in infants, children, and adolescents?

| Study | Reason for exclusion |
| --- | --- |
| Bailey, D. J., et al. (1987). "Lack of efficacy of thickened feeding as treatment for gastroesophageal reflux." J Pediatr 110(2): 187-189. | Wrong study design |
| Belei, O., et al. (2018). "Is it useful to administer probiotics together with proton pump inhibitors in children with gastroesophageal reflux?" Journal of Neurogastroenterology and Motility 24(1): 51-57. | Wrong intervention |
| Bhat, R. Y., et al. (2007). "Acid gastroesophageal reflux in convalescent preterm infants: effect of posture and relationship to apnea." Pediatr Res 62(5): 620-623. | Wrong population |
| Carroll, A. E., et al. (2002). "A systematic review of nonpharmacological and nonsurgical therapies for gastroesophageal reflux in infants." Arch Pediatr Adolesc Med 156(2): 109-113. | Systematic review including studies already in our selection or not meeting inclusion criteria |
| Chang, A. B., et al. (2011). "Gastro-oesophageal reflux treatment for prolonged non-specific cough in children and adults." Cochrane Database Syst Rev 2011(1): Cd004823. | Systematic review including studies already in our selection or not meeting inclusion criteria |
| Gerasimov, S., et al. (2018). "Role of lactobacillus rhamnosus (Floraactive™) 19070-2 and lactobacillus reuteri (floraactive™) 12246 in infant colic: A randomized dietary study." Nutrients 10(12). | Wrong population |
| Giglione, E., et al. (2016). "The Association of Bifidobacterium breve BR03 and B632 is Effective to Prevent Colics in Bottle-fed Infants." J Clin Gastroenterol 50: S164-S167. | Wrong population |
| Gouyon, J. B., et al. (1989). "Smectite reduces gastroesophageal reflux in newborn infants." Dev Pharmacol Ther 13(1): 46-50. | Wrong study design |
| Hill, D. J., et al. (2000). "Role of food protein intolerance in infants with persistent distress attributed to reflux esophagitis." J Pediatr 136(5): 641-647. | Wrong intervention |
| Horvath, A., et al. (2008). "The effect of thickened-feed interventions on gastroesophageal reflux in infants: systematic review and meta-analysis of randomized, controlled trials." Pediatrics 122(6): e1268-1277. | Systematic review including studies already in our selection or not meeting inclusion criteria |
| Kumar, Y. and R. Sarvananthan (2008). "GORD in children." BMJ Clin Evid 2008. | Systematic review including studies already in our selection or not meeting inclusion criteria |
| Kwok, T. C., et al. (2017). "Feed thickener for infants up to six months of age with gastro-oesophageal reflux." Cochrane Database Syst Rev 12(12): Cd003211. | Systematic review including studies already in our selection or not meeting inclusion criteria |
| Ling, W., et al. (2015). "Consistent Efficacy of Wendan Decoction for the Treatment of Digestive Reflux Disorders." Am J Chin Med 43(5): 893-913. | Wrong study design |
| Loots, C., et al. (2013). "Effect of lateral positioning on gastroesophageal reflux (GER) and underlying mechanisms in GER disease (GERD) patients and healthy controls." Neurogastroenterol Motil 25(3): 222-229, e161-222. | Wrong population |
| Meyer, R., et al. (2015). "Systematic review of the impact of feed protein type and degree of hydrolysis on gastric emptying in children." BMC Gastroenterol 15: 137. | Systematic review including studies already in our selection or not meeting inclusion criteria |
| Miyazawa, R., et al. (2008). "Effects of pectin liquid on gastroesophageal reflux disease in children with cerebral palsy." BMC Gastroenterol 8: 11. | Wrong population |
| Neu, M., et al. (2012). "A review of nonsurgical treatment for the symptom of irritability in infants with GERD." Journal for Specialists in Pediatric Nursing 17(3): 177-192. | Systematic review including studies already in our selection or not meeting inclusion criteria |
| Neu, M., et al. (2014). "Interactions during feeding with mothers and their infants with symptoms of gastroesophageal reflux." J Altern Complement Med 20(6): 493-499. | Wrong outcome |
| Orenstein, S. R. (1990). "Effects on behavior state of prone versus seated positioning for infants with gastroesophageal reflux." Pediatrics 85(5): 765-767. | Wrong study design |
| Orenstein, S. R. and J. D. McGowan (2008). "Efficacy of conservative therapy as taught in the primary care setting for symptoms suggesting infant gastroesophageal reflux." J Pediatr 152(3): 310-314. | No comparator |
| Orenstein, S. R., et al. (2006). "Natural history of infant reflux esophagitis: symptoms and morphometric histology during one year without pharmacotherapy." Am J Gastroenterol 101(3): 628-640. | Wrong intervention |
| Salehi, M., et al. (2017). "Medicinal plants for management of gastroesophageal reflux disease: A review of animal and human studies." Journal of Alternative and Complementary Medicine 23(2): 82-95. | Wrong study design |
| Yan, S., et al. (2015). "Preventative effect of massage on gastric volvulus in infants with gastroesophageal reflux-induced pneumonia." J Tradit Chin Med 35(5): 520-527. | Wrong comparator |

### PICO 7 - What are the indications and effectiveness of different surgical and endoscopic treatment options for GERD in infants, children, and adolescents?

| Study | Reason for exclusion |
| --- | --- |
| Alganabi, M., et al. (2021). "Surgical site infection after open and laparoscopic surgery in children: a systematic review and meta-analysis." Pediatr Surg Int 37(8): 973-981. | Wrong outcome |
| Analatos, A., et al. (2022). "Clinical Outcomes of a Laparoscopic Total vs a 270° Posterior Partial Fundoplication in Chronic Gastroesophageal Reflux Disease: A Randomized Clinical Trial." JAMA Surg 157(6): 473-480. | Wrong population |
| Aye, R. W., et al. (2012). "A randomized multiinstitution comparison of the laparoscopic Nissen and Hill repairs." Ann Thorac Surg 94(3): 951-957; discussion 957-958. | Wrong population |
| Balakrishna, P., et al. (2015). "Symptomatic outcome following laparoscopic Heller's cardiomyotomy with Dor fundoplication versus laparoscopic Heller's cardiomyotomy with angle of His accentuation: results of a randomized controlled trial." Surg Endosc 29(8): 2344-2351. | Wrong population |
| Booth, M. I., et al. (2008). "Randomized clinical trial of laparoscopic total (Nissen) versus posterior partial (Toupet) fundoplication for gastro-oesophageal reflux disease based on preoperative oesophageal manometry." Br J Surg 95(1): 57-63. | Wrong population |
| Broeders, J. A., et al. (2011). "Impact of surgeon experience on 5-year outcome of laparoscopic Nissen fundoplication." Arch Surg 146(3): 340-346. | Wrong population |
| Broeders, J. A., et al. (2009). "Ten-year outcome of laparoscopic and conventional nissen fundoplication: randomized clinical trial." Ann Surg 250(5): 698-706. | Wrong population |
| Broeders, J. A., et al. (2012). "Five-year outcome after laparoscopic anterior partial versus Nissen fundoplication: four randomized trials." Ann Surg 255(4): 637-642. | Wrong population |
| Cadière, G. B., et al. (2001). "Evaluation of telesurgical (robotic) NISSEN fundoplication." Surg Endosc 15(9): 918-923. | Wrong population |
| Eyre-Brook, I. A., et al. (1993). "Results of a prospective randomized trial of the Angelchik prosthesis and of a consecutive series of 119 patients." Br J Surg 80(5): 602-604. | Wrong population |
| Glen, P., et al. (2014). "Partial versus complete fundoplication for the correction of pediatric GERD: a systematic review and meta-analysis." PLoS One 9(11): e112417. | Wrong study design |
| Hambraeus, M., et al. (2013). "A literature review of the outcomes after robot-assisted laparoscopic and conventional laparoscopic Nissen fundoplication for gastro-esophageal reflux disease in children." Int J Med Robot 9(4): 428-432. | Wrong outcome |
| Lei, X., et al. (2017). "Outcome Evaluation of Laparoscopic and Open Nissen Fundoplication in Children-A Systematic Review and Meta-Analysis." Am Surg 83(1): 90-97. | Wrong study design |
| Li, G., et al. (2023). "Laparoscopic Nissen Versus Toupet Fundoplication for Short- and Long-Term Treatment of Gastroesophageal Reflux Disease: A Meta-Analysis and Systematic Review." Surg Innov 30(6): 745-757. | Wrong population |
| Mauritz, F. A., et al. (2013). "Complete versus partial fundoplication in children with gastroesophageal reflux disease: results of a systematic review and meta-analysis." J Gastrointest Surg 17(10): 1883-1892. | Wrong study design |
| McKinley, S. K., et al. (2021). "Surgical treatment of GERD: systematic review and meta-analysis." Surg Endosc 35(8): 4095-4123. | Wrong study design |
| Rosen, R., et al. (2018). "Pediatric Gastroesophageal Reflux Clinical Practice Guidelines: Joint Recommendations of the North American Society for Pediatric Gastroenterology, Hepatology, and Nutrition and the European Society for Pediatric Gastroenterology, Hepatology, and Nutrition." J Pediatr Gastroenterol Nutr 66(3): 516-554. | Systematic review including studies already in our selection or not meeting inclusion criteria |
| Siddiqui, M. R., et al. (2011). "A meta-analysis of outcomes after open and laparoscopic Nissen's fundoplication for gastro-oesophageal reflux disease in children." Pediatr Surg Int 27(4): 359-366. | Wrong study design |
| Sload, R. L. and M. T. Brigger (2014). "Surgery for reflux induced airway disease: a systematic review." Int J Pediatr Otorhinolaryngol 78(8): 1211-1215. | Wrong study design |
| Zhang, P., et al. (2016). "Laparoscopic vs. open Nissen's fundoplication for gastro-oesophageal reflux disease in children: A meta-analysis." Int J Surg 34: 10-16. | Wrong study design |

### PICO 8 – What is the prognosis of GER and GERD in infants, children, and adolescents, and what are the prognostic factors?

| Study | Reason for exclusion |
| --- | --- |
| Campanozzi, A., et al. (2009). "Prevalence and natural history of gastroesophageal reflux: Pediatric prospective survey." Pediatrics 123(3): 779-783. | Wrong outcome |
| Fass, R., et al. (2021). "Gastro-oesophageal reflux disease." Nature Reviews Disease Primers 7(1). | Wrong population |
| Glassman, M., et al. (1995). "GASTROESOPHAGEAL REFLUX IN CHILDREN - CLINICAL MANIFESTATIONS, DIAGNOSIS, AND THERAPY." Gastroenterology Clinics of North America 24(1): 71-98. | Wrong study design |
| Heine, R. G. (2008). "Management of gastro-oesophageal reflux disease in childhood." Paediatrics and Child Health 18(10): 448-452. | Wrong study design |
| Nelson, S. P., et al. (1998). "One-year follow-up of symptoms of gastroesophageal reflux during infancy. Pediatric Practice Research Group." Pediatrics 102(6): E67. | Wrong study design |
| Salvatore, S., et al. (2004). "The natural course of gastro-oesophageal reflux." Acta Paediatrica, International Journal of Paediatrics 93(8): 1063-1069. | Wrong study design |
| Sontag, S. J. (2005). "The spectrum of pulmonary symptoms due to gastroesophageal reflux." Thorac Surg Clin 15(3): 353-368. | Wrong study design |
| Sontag, S. J. (2007). "The spectrum of gastroesophageal reflux disease." J Clin Gastroenterol 41(6): S118-S128. | Wrong study design |
| Suwandhi, E., et al. (2006). "Gastroesophageal reflux in infancy and childhood." Pediatr Ann 35(4): 259-266. | Wrong study design |
| Vakil, N. (2010). "Disease definition, clinical manifestations, epidemiology and natural history of GERD." Best Practice and Research: Clinical Gastroenterology 24(6): 759-764. | Wrong study design |
| van der Pol, R. J., et al. (2015). "Follow-Up After pH-Metry and pH Impedance in Pediatric Gastroesophageal Reflux Disease." J Pediatr Gastroenterol Nutr 60(2): 224-229. | Wrong outcome |
| Winter, H. S., et al. (2011). "Review of the persistence of gastroesophageal reflux disease in children, adolescents and adults: Does gastroesophageal reflux disease in adults sometimes begin in childhood?" Scand J Gastroenterol 46(10): 1157-1168. | Wrong population |
| Young, R. J., et al. (2007). "A retrospective, case-control pilot study of the natural history of pediatric gastroesophageal reflux." Dig Dis Sci 52(2): 457-462. | Wrong outcome |
| Zeiter, D. K. and J. S. Hyams (1999). "Gastroesophageal reflux: pathogenesis, diagnosis, and treatment." Allergy and asthma proceedings : the official journal of regional and state allergy societies 20(1): 45-49. | Wrong population |

## Extended Methods for the Systematic Review

### Objective

The objective of this guideline is to provide evidence-based recommendations for the diagnosis and management of gastroesophageal reflux (GER) and gastroesophageal reflux disease (GERD) in infants, children, and adolescents. The guideline aims to support healthcare professionals in delivering consistent and effective care to pediatric patients with GER or GERD, by integrating current evidence and expert consensus.

The primary health intents of this guideline are diagnosis, treatment, and management of GER/GERD in the pediatric population.

The expected benefits include improved accuracy of diagnosis, appropriate use of pharmacological and non-pharmacological treatments, reduction of unnecessary testing, and optimized patient outcomes.

The guideline is intended for use by pediatricians, pediatric gastroenterologists, general practitioners, and other healthcare professionals involved in the care of children with suspected or confirmed GER or GERD.

### Participants and structure

A multidisciplinary panel of 16 physicians with expertise in the diagnosis and management of pediatric GER and GERD and pediatric guidelines was convened by the Presidents of the Italian Society of Pediatrics (SIP) (A.S.) and of Italian Society of Pediatric Gastroenterology Hepatology and Nutrition (SIGENP) (C.R.). The President (S.C.) of the Italian Association of Neonates with reflux (AINER) also participated to the first online meetings providing comments on the content of this guideline, on the identified PICOs and on unmet needs of young patients. She also read and commented on the draft of the document and the final manuscript. An external Agency (CREA Sanità) with experience in data analysis and applied healthcare research participated to all the meetings and the Evidence Review Team (ERT), composed of experts in evidence synthesis conducted a preliminary search of international guidelines on the diagnosis and management of GER and GERD in children to support the panel in the development of PICO questions. A systematic literature search was performed in PubMed, Embase, Web of Science, and Google Scholar on February 22, 2024 (Additional File 1). The quality of the identified guidelines was assessed using the AGREE II tool [1]. PICO questions from the included guidelines were extracted, analyzed, and presented to the panel, which then used a Delphi process to prioritize them and define a final list of key questions considered critical to address. The panel identified eight key questions concerning infants, children and adolescents (Table 1):

**Table 1. List of clinical questions identified as relevant for this guideline**

| Questions | PICO |
| --- | --- |
| 1. What is the definition of GER and GERD? | NO |
| 2. What are the signs and symptoms associated with GER and GERD? | YES |
| 3. What are the risk factors for GERD? | YES |
| 4. What is the value of different diagnostic testing for GERD? | YES |
| 5. What is the evidence of effectiveness of pharmacologic treatment for GER and GERD? | YES |
| 6. What is the effectiveness of different non-pharmacologic treatment options for GER and GERD? | YES |
| 7. What is the indication and the effectiveness of different surgical/endoscopic treatment options for GERD? | YES |
| 8. What is the prognosis of GER and GERD and what are prognostic factors? | NO |

Questions 2 through 6 were structured using the PICO format, while questions 1 and 8 were formulated as narrative clinical questions, due to their descriptive nature.

Subsequently, the ERT, in collaboration with the panel, developed search strategies tailored to each key question. Systematic literature searches were conducted in PubMed, Embase, and Web of Science on May 11–12, 2024. The complete search strategies and strings with MESH terms are provided in Additional File 1.

Manual retrieval of additional original studies or reviews from references of the papers identified by the systematic review was also performed by all authors and, whenever deemed useful for the purpose of this document the studies were included in the related PICO evidence summary.

### Systematic review of international guidelines on GER and GERD

The study selection process was carried out independently by two reviewers in two phases. Initially, titles and abstracts were screened according to predefined inclusion criteria: English-language guidelines focusing on the diagnosis of GER and GERD in pediatric populations. Subsequently, potentially eligible full-text articles were assessed. Any disagreements between reviewers were resolved through discussion. Data extraction was performed by one reviewer and verified by a second. The methodological quality of guidelines was assessed by three independent reviewers through the AGREE II tool. The literature selection process is illustrated in Additional File 1 (PRISMA 2020 Flow Diagram). A list of excluded studies with reasons for exclusion is provided in the Additional File 1.

The literature search initially identified 350 records. After removing duplicates, 217 records were screened by title and abstract, and 30 full-text articles were assessed for eligibility. Of these, 27 were excluded, and 3 [2-4] were included in the final analysis. The first document [3] dated 2018, was a clinical practice guideline on pediatric GER and GERD, jointly developed by the North American Society for Pediatric Gastroenterology, Hepatology, and Nutrition (NASPGHAN) and the European Society for Pediatric Gastroenterology, Hepatology, and Nutrition (ESPGHAN). This work represents an update of their previous joint guideline published in 2009 [5].

The second guideline identified was developed by the National Institute for Health and Care Excellence (NICE) in 2015 [2], and updated in 2019 [6]. It addresses the diagnosis and management of GERD in children and young people.

The third document [4] was a guideline by the Society of American Gastrointestinal and Endoscopic Surgeons (SAGES), focusing on the surgical treatment of GERD in both adult and pediatric patients.

The methodological quality of the three guidelines varied. The NASPGHAN/ESPGHAN guideline was rated as low quality (AGREE II total score: 55/100; Domain 3 score: 40/100). The SAGES guideline was considered to be of moderate quality (AGREE II total score: 60/100; Domain 3 score: 59/100). The NICE guideline received the highest rating, judged to be of good quality (AGREE II total score: 73/100; Domain 3 score: 72/100).

### Systematic reviews on the eight key questions

Eight systematic reviews were conducted, one for each key question. A study protocol for these systematic reviews was registered in the PROSPERO database (CRD420251041380). All reviews were conducted following Cochrane methodology [7], and reported in accordance with the PRISMA 2020 statement [8, 9]. The guideline itself was reported in accordance with the AGREE Reporting Checklist [10].

#### Eligibility criteria

**Population**: for key questions 1-4, we included infants, children, and adolescents (0–18 years) presenting with signs and symptoms suggestive of GER or GERD, irrespective of formal diagnosis. For key questions 5-8, we included infants, children, and adolescents (0–18 years) with a confirmed diagnosis of GER or GERD, according to any recognized clinical or diagnostic criteria.

**Interventions or exposures**:

Key Question 1 (Definition): no specific intervention or exposure. We included studies providing definitions of GER or GERD.

PICO Question 2 (Signs and symptoms): the exposure was the presence of specific signs or symptoms potentially associated with GER/GERD, including: heartburn, difficulty swallowing, nausea, chronic cough, bloating, refusal to feed, regurgitation, chest pain, wheezing, sore throat, dysphagia, laryngitis, vomiting, weight loss, pneumonia, dental erosion, otitis, apnea, and Sandifer syndrome.

PICO Question 3 (Risk factors): the exposure was the presence of potential risk factors associated with GERD development, including: neurodevelopmental disorders, cerebral palsy, prematurity, family history of GERD, surgical or congenital abnormalities, congenital heart disease, chronic lung disease, and obesity.

PICO Question 4 (Diagnostic testing): the intervention was the use of diagnostic tests for GERD (e.g., esophageal pH monitoring, pH-impedance (pH-MII), endoscopy, esophageal manometry, scintigraphy, ultrasonography, and clinical diagnostic tools or questionnaires).

PICO Question 5 (Pharmacological treatment): the intervention was pharmacologic therapy for GERD (e.g., proton pump inhibitors, H2-receptor antagonists, prokinetic agents, antacids).

PICO Question 6 (Non-pharmacological treatment): the intervention included non-pharmacological approaches (e.g., dietary modifications, feeding interventions, positional therapy, behavioral therapy, alginates, massage therapy, and complementary medicine).

PICO Question 7 (Surgical/endoscopic treatment): the intervention consisted of surgical or endoscopic treatments for GERD (e.g., fundoplication, endoscopic procedures).

Key Question 8 (Prognosis): no specific intervention. We included studies evaluating the natural history or prognostic factors of GER/GERD.

**Comparators or controls**

Where applicable, comparators included placebo, no treatment, usual care, or alternative diagnostic or therapeutic interventions.

**Study design**

Key Question 1 (Definition): Guidelines, consensus statements, and observational studies (cohort, case-control, cross-sectional).

PICO Questions 2-3 (Signs/symptoms, Risk factors): Systematic reviews; observational studies (cohort, case-control, cross-sectional).

PICO Question 4 (Diagnostic testing): Systematic reviews/meta-analyses of diagnostic accuracy studies or RCTs; primary diagnostic accuracy studies (cross-sectional or cohort) and RCTs.

PICO Questions 5–7 (Pharmacologic, Non-Pharmacologic, Surgical/Endoscopic): Systematic reviews/meta-analyses of RCTs; RCTs.

Key Question 8 (Prognosis): Systematic reviews and observational studies (cohort, case-control, cross-sectional).

**Outcomes**

Key Question 1 (Definition): Definitions and criteria used to define GER and GERD in infants, children, and adolescents.

PICO Question 2 (Signs and symptoms): Diagnostic accuracy (e.g., sensitivity, specificity) of individual signs and symptoms associated with GER/GERD.

PICO Question 3 (Risk factors): Association between potential risk factors and GERD development (measured as odds ratios [OR], relative risks [RR], or hazard ratios [HR]).

PICO Question 4 (Diagnostic testing): Diagnostic accuracy of different tests (e.g., sensitivity, specificity, positive and negative predictive values, likelihood ratios).

PICO Question 5 (Pharmacological treatment): Effectiveness and safety of pharmacological treatments in improving GERD symptoms or healing esophagitis (measured by symptom resolution rates, esophagitis healing rates, adverse events).

PICO Question 6 (Non-pharmacological treatment): Effectiveness and safety of non-pharmacological treatments in reducing GERD symptoms or preventing GERD complications (measured by symptom improvement rates, quality of life scores, adverse events).

PICO Question 7 (Surgical/endoscopic treatment): Effectiveness and safety of surgical and endoscopic interventions (e.g., symptom resolution, esophagitis healing, reintervention rates, adverse events).

Key Question 8 (Prognosis): Natural history and prognosis of GER/GERD (e.g., rates of spontaneous resolution, persistence, or progression; factors associated with prognosis).

**Context**

Studies conducted in any healthcare setting (e.g., hospital, outpatient clinic, primary care, community settings) were included. No restrictions were applied based on geographic location, healthcare system, or country income level.

#### Review process

The literature selection process was performed independently by pairs of reviewers for each key question. The first selection was based on title and abstract screening. Articles selected in this phase were subsequently assessed for eligibility. In both phases, disagreements were resolved by consensus. Data extraction and quality assessment were performed by one reviewer and verified by another reviewer. Quality assessment was performed using tools specific to study design. The quality of observational studies and systematic reviews was assessed by the JBI checklists [11], while RCTs were evaluated using the Cochrane RoB 2 tool [12].

Subgroups of at least two participating authors focused on the different PICOs and topics, reviewed the provided list of documents and related references, produced a written text on the summary of evidence with recommendations that were discussed by all authors during the meetings and voted online.

#### Statistical analysis

Data for all key questions were synthesized using GRADE evidence profile tables and narrative summaries. When studies were sufficiently homogeneous in terms of population, interventions, comparators, and outcomes for each comparison, meta-analyses were performed using random-effects models. Effect measures included risk ratios (RR), odds ratios (OR), hazard ratios (HR), mean differences (MD), or standardized mean differences (SMD), as reported in the included studies. When meta-analyses were conducted, statistical heterogeneity was assessed using the I² statistic. Subgroup and sensitivity analyses were not performed due to insufficient data being available. P-values < 0.05 were considered statistically significant.

#### Certainty assessment

The GRADE (Grading of Recommendations Assessment, Development and Evaluation) approach [13] was applied to assess the certainty of evidence for PICO questions 2 through 7. The certainty of evidence was rated as high, moderate, low, or very low, based on the domains of risk of bias, inconsistency, indirectness, imprecision, and publication bias.

Evidence from RCTs was initially considered high certainty and downgraded by one level for serious limitations, or by two levels for very serious limitations in any of the above domains. Conversely, evidence from observational studies started at low certainty.

For the risk of bias domain, we downgraded by one level if at least one study was judged having some concerns, while we downgraded by two levels if most of studies had a high risk of bias in more than one domain. Inconsistency was evaluated based on the presence of unexplained heterogeneity across study results. For indirectness, we evaluated whether the populations, interventions, comparators and outcomes considered in the included studies correspond to those planned in the PICO questions. We downgraded for imprecision if the effect estimates are from studies with a small sample size and wide confidence intervals. Publication bias was not assessed due to the limited number of studies for each comparison.

Factors that may increase the certainty level, such as large magnitude of an effect, dose-response gradient, and effect of plausible residual confounding, were also considered. GRADE Evidence profile tables were created by GRADEpro GDT software [14].

#### Evidence to Decision framework

A formal Evidence to Decision (EtD) framework was applied for PICO questions 5 to 7. This approach was not deemed applicable to the remaining key questions. In accordance with the EtD methodology, the following domains were systematically evaluated:

- Problem: Is the health problem a priority?
- Desirable Effects: How substantial are the desirable anticipated effects?
- Undesirable Effects: How substantial are the undesirable anticipated effects?
- Certainty of evidence: What is the overall certainty of the evidence of effects?
- Values: Is there important uncertainty about or variability in how much people value the main outcomes?
- Balance of effects: Does the balance between desirable and undesirable effects favor the intervention or the comparison?
- Resources required: What resources are needed to implement the intervention?
- Certainty of evidence of required resources: What is the certainty of the evidence of resource requirements (costs)?
- Cost effectiveness: Does the cost-effectiveness of the intervention favor the intervention or the comparison?
- Equity: What would be the impact on health equity?
- Acceptability: Is the intervention acceptable to key stakeholders?
- Feasibility: Is the intervention feasible to implement?

Based on the judgements given on these domains, the expert panel proposed the strength of recommendations (SoR) as strong or weak against or weak or strong in favor of the intervention.

#### Consensus process

Recommendations, including their direction and strength, were developed through a combination of structured discussion and iterative voting. A two-round Delphi process was conducted between April and May 2025. Consensus was defined as ≥80% agreement or disagreement (scores 7–9 or scores 1-3 on a 9-point Likert scale).

The strength and direction of each recommendation were also explored through voting, with participants selecting one of the four predefined categories:

- Strong recommendation against the intervention
- Weak recommendation against the intervention
- Weak recommendation for the intervention
- Strong recommendation for the intervention

Final consensus on both the content and the strength of each recommendation was achieved through discussion during the plenary session, with all panel members participating.

A total of 40 recommendations related to PICO questions 2 through 7 were formulated and finalized through this combined process.

#### External review

An external review was conducted to ensure the validity, applicability, and clarity of both the guideline text and the recommendations. The draft guideline was shared with three pediatric external experts on GERD (Osvaldo Borrelli, Yvan Vandenplas, Mario C. Vieira) and with the Presidents of Italian Federation of Societies of Digestive Diseases (FISMAD), of the Italian Pediatric Society of Neonatology (SIN) and of Respiratory Diseases (SIMRI) and of an Association of Italian Family Pediatricians who were not involved in the development process. These included primary care and hospital pediatricians, pediatric gastroenterologists, adult gastroenterologists and representatives from relevant Italian scientific societies.

Reviewers were selected based on their clinical or methodological expertise and institutional role.

They were invited to provide comments and suggestions, focusing on the clarity, relevance, feasibility, and potential impact of each recommendation.

All feedback received was summarized and discussed during a plenary meeting of the guideline panel. Where appropriate, relevant suggestions and criticisms were incorporated into the final version of the recommendations.

#### Facilitators and Barriers to Application

Facilitators and barriers to the implementation of the guideline recommendations were considered qualitatively by the panel during the development process, particularly within the Evidence to Decision (EtD) framework for PICO questions 5 to 7. Although no formal pilot testing or surveys with stakeholders were conducted, the panel drew on their clinical experience across diverse healthcare settings in Italy, including tertiary hospitals, community hospitals, and outpatient pediatric services.

Facilitators identified included:

- Broad availability of diagnostic tools (e.g., esophageal pH-impedance, endoscopy) in referral centers
- Existing clinical awareness of GER/GERD in pediatrics
- High relevance and clarity of the structured recommendations
- Multidisciplinary interest in standardized management approaches

Barriers noted included:

- Limited access to specialized testing in some geographic areas
- Variability in healthcare professionals' familiarity with non-pharmacological management strategies
- Potential overuse or misuse of pharmacological treatments
- Lack of awareness of updated evidence among general practitioners

These considerations were discussed during the formulation of recommendations and helped guide decisions on the strength of recommendations, as well as the inclusion of non-pharmacological and context-sensitive options where evidence supported them. Where barriers were identified, recommendations were worded to allow flexibility based on available resources and clinical judgment.

#### Implementation Tools and Advice

To support the practical application of this guideline, a summary table was developed (Table…) presenting each PICO question alongside the corresponding recommendations and their strength and direction. Two figures (Fig… and Fig …) were created to represent diagnostic and therapeutic algorithm for pediatric GERD and strength of recommendations for or against different treatment in infants, children and adolescents. These tools are intended to facilitate quick reference and integration of the guideline content into clinical decision-making.

The summary was designed for use by pediatric healthcare professionals across various settings, including hospitals and outpatient services. It can assist in translating evidence-based recommendations into practice and in promoting consistency in the management of GER and GERD in infants, children, and adolescents.

#### Resource Implications

No formal systematic search of economic evaluations or cost-effectiveness studies was conducted or retrieved as part of this guideline. However, considerations regarding potential resource implications were discussed by the panel during the formulation of recommendations related to PICO questions 5–7 (pharmacological, non-pharmacological, and surgical/endoscopic treatments). These aspects were addressed within the Evidence to Decision (EtD) tables, which included a domain specifically focused on resource use. The panel considered, when applicable, the availability and costs of interventions in the Italian healthcare context, including drug acquisition, hospital-based procedures, and access to specialized services. While no quantitative cost data were formally incorporated, qualitative judgments about the feasibility and sustainability of implementing each intervention were explicitly documented and contributed to the final formulation of the recommendations.

#### Monitoring and Auditing Criteria

At the time of publication, no formal set of monitoring or auditing tools has been developed for this guideline. However, the panel acknowledges the importance of tracking the implementation and real-world impact of the recommendations. To this end, a set of core process and outcome indicators will be proposed during the dissemination phase, in collaboration with national pediatric and gastroenterology societies and patients’ associations.

Potential indicators could include:

- Adherence to recommended diagnostic pathways (e.g., appropriate use of pH-impedance monitoring or endoscopy)
- Appropriate prescription of pharmacological treatments (e.g., proton pump inhibitors limited to evidence-based indications and durations)
- Reduction in the use of ineffective or non-recommended interventions
- Surgical referrals based on agreed clinical criteria

These indicators can be used by institutions for local audits, quality improvement initiatives, and benchmarking practices. Data may be collected through surveys, retrospective chart reviews, electronic medical record queries, or standardized data collection forms.

Where feasible, annual or biennial audits are encouraged to assess adherence and identify implementation barriers. The panel supports the integration of these indicators into national and international networks, to facilitate continuous monitoring.

#### Updating procedure

This guideline will be updated regularly to ensure its continued relevance in light of emerging evidence and evolving clinical practice. The development group has established a plan to review and update the guideline every five years, or earlier if substantial new evidence becomes available that may warrant changes to current recommendations.

The same methodology used for the present guideline—based on systematic reviews, GRADE assessments, and the Evidence to Decision (EtD) framework—will be applied during the updating process.

The literature searches will be updated starting from the date of the last search conducted for this guideline. In the update process, the working group will also assess the continued relevance of the existing PICO questions, and may decide to revise, remove, or introduce new questions based on clinical need and stakeholder input.

The update will ideally be carried out by the current guideline development group or an appointed successor team, in collaboration with methodological experts.

# References

1. Brouwers MC, Kho ME, Browman GP, Burgers JS, Cluzeau F, Feder G, et al. AGREE II: advancing guideline development, reporting and evaluation in health care. Cmaj. 2010;182(18):E839-42.

2. Davies I, Burman-Roy S, Murphy MS. Gastro-oesophageal reflux disease in children: NICE guidance. Bmj. 2015;350:g7703.

3. Rosen R, Vandenplas Y, Singendonk M, Cabana M, DiLorenzo C, Gottrand F, et al. Pediatric Gastroesophageal Reflux Clinical Practice Guidelines: Joint Recommendations of the North American Society for Pediatric Gastroenterology, Hepatology, and Nutrition and the European Society for Pediatric Gastroenterology, Hepatology, and Nutrition. J Pediatr Gastroenterol Nutr. 2018;66(3):516-54.

4. Slater BJ, Dirks RC, McKinley SK, Ansari MT, Kohn GP, Thosani N, et al. SAGES guidelines for the surgical treatment of gastroesophageal reflux (GERD). Surg Endosc. 2021;35(9):4903-17.

5. Vandenplas Y, Rudolph CD, Di Lorenzo C, Hassall E, Liptak G, Mazur L, et al. Pediatric gastroesophageal reflux clinical practice guidelines: Joint recommendations of the North American Society for Pediatric Gastroenterology, Hepatology, and Nutrition (NASPGHAN) and the European Society for Pediatric Gastroenterology, Hepatology, and Nutrition (ESPGHAN). J Pediatr Gastroenterol Nutr. 2009;49(4):498-547.

6. NICE. Gastro-oesophageal reflux disease in children and young people: diagnosis and management. NICE guideline. Reference number: NG1. Published: 14 January 2015. Last updated: 09 October 2019. Available at: <https://www.nice.org.uk/guidance/ng1> (accessed: 25.05.2025). 2015.

7. Higgins JPT, Thomas J, Chandler J, Cumpston M, Li T, Page MJ, Welch VA (editors). Cochrane Handbook for Systematic Reviews of Interventions version 6.5 (updated August 2024). Cochrane, 2024. Available from [www.training.cochrane.org/handbook](http://www.training.cochrane.org/handbook) (accessed: 25.05.2025).

8. Page MJ, McKenzie JE, Bossuyt PM, Boutron I, Hoffmann TC, Mulrow CD, et al. The PRISMA 2020 statement: an updated guideline for reporting systematic reviews. Bmj. 2021;372:n71.

9. Page MJ, Moher D, Bossuyt PM, Boutron I, Hoffmann TC, Mulrow CD, et al. PRISMA 2020 explanation and elaboration: updated guidance and exemplars for reporting systematic reviews. Bmj. 2021;372:n160.

10. Brouwers MC, Kerkvliet K, Spithoff K. The AGREE Reporting Checklist: a tool to improve reporting of clinical practice guidelines. Bmj. 2016;352:i1152.

11. Aromataris E, Lockwood C, Porritt K, Pilla B, Jordan Z, editors. JBI Manual for Evidence Synthesis. JBI; 2024. Available from: <https://synthesismanual.jbi.global>. <https://doi.org/10.46658/JBIMES-24-01> (accessed: 25.05.2025).

12. Sterne JAC, Savović J, Page MJ, Elbers RG, Blencowe NS, Boutron I, et al. RoB 2: a revised tool for assessing risk of bias in randomised trials. Bmj. 2019;366:l4898.

13. Schünemann HJ, Higgins JP, Vist GE, Glasziou P, Akl EA, Skoetz N, et al. Chapter 14: Completing ‘Summary of findings’ tables and grading the certainty of the evidence. In: Higgins JPT, Thomas J, Chandler J, Cumpston M, Li T, Page MJ, Welch VA (editors). Cochrane Handbook for Systematic Reviews of Interventions version 6.5 (updated August 2024). Cochrane, 2024. Available from [www.training.cochrane.org/handbook](http://www.training.cochrane.org/handbook), accessed 10 May 2025.

14. McMaster University (developed by Evidence Prime) GRADEpro GDT. Hamilton (ON): McMaster University (developed by Evidence Prime), accessed 10 May 2025. Available at [www.gradepro.org](http://www.gradepro.org).
