# Supplementary material for: Guidelines on diagnosis and management of gastroesophageal reflux disease in infants, children and adolescents: a joint consensus from Italian pediatric societies (SIP and SIGENP) -Part II: management
Source: Ital J Pediatr. 2026 Apr 10;52:90. doi: 10.1186/s13052-026-02255-0 (PMC13182049; doi:10.1186/s13052-026-02255-0)
Supplement: Supplementary file 2 — Additional File 2 [file 13052_2026_2255_MOESM2_ESM.docx]

# Additional File 2

## Characteristics of included studies

### PICO 5 - What is the evidence for the effectiveness of pharmacological treatments for GER and GERD in infants, children, and adolescents?

| Study ID | Country | Study Design | Population (condition, age) | Sample Size (N, sex %) | Intervention (drug, posology) | Drug class | Comparator (drug, posology) | Drug class | Outcome efficacy | Outcome safety |
| --- | --- | --- | --- | --- | --- | --- | --- | --- | --- | --- |
| Alla 2024 | Not applicable | Systematic review | Children (GERD; 7.39 years ± 4.69) | 18 RCT and 12 cohort studies (762,505; 51.2% ♂, 48.8% ♀) | Esomeprazole (n = 7, 20.5%), rabeprazole (n = 4, 11.7%), dexlamoprazole (n = 2, 5.8%), lansoprazole (n = 8, 23.5%), pantoprazole (n = 7, 20.5%), and omeprazole (n = 6, 17.6%) | Proton Pump Inhibitors (PPI) | NR | - | NR | "A total of 53 309 side effects were reported, accounting for 6.99% of the total. Diarrhea was the most predominant GI side effect (0.12% of total effects), followed by vomiting.  (0.11% of total effects), abdominal pain (0.09% of total effects), and less frequently, nausea (0.02% of total effects). Dermatological side effects were skin rash (0.02% of total effects), urticaria (0.004% of total effects), eczema (0.03% of total effects), and dermatitis (0.037% of total effects)." |
| Arabpour 2023 | Not applicable | Systematic review | Children and adults (GERD, 7.1 months-10.0 years in pediatric studies) | 6 pediatric RCT (228, 50% ♂, 50% ♀) | Baclofen (1 study had Baclofen + PPI) | Prokinetic agents | Placebo (1 study had PPI) | - | Four studies reported changes in clinical status. All of them confirmed Baclofen’s significant efficacy in the improvement of clinical status by symptom remission, weight gain, or reduction in crying and restlessness. Three studies evaluated the efficacy of baclofen in children using invasive GI procedures (pH monitoring or esophageal manometry). | Adverse effects were reported only in 2 studies. Breathlessness (n=2; 1 placebo group, 1 baclofen group); tiredness (n=2; 1 placebo group, 1 baclofen group) and nausea (n=1; baclofen group). None of these events was considered significant. These symptoms were judged to most likely be reflux disease itself or discomfort caused by the invasive GI procedures rather than baclofen. A slight reduction in muscle tone in 12.5% of patients. |
| Argüelles-Martin 1989 | Spain | RCT | Pediatric patients (GERD, 6.1 ± 3.6 years) | 75 (54% ♂, 46% ♀) | Group 1: Sucralfate tablet (0.5-1 mg every 6h); Group 2: Sucralfate suspension (0.5-1 mg every 6h) | Sucralfate | Cimetidine (20 mg/kg/die) | Histamine H2 antagonists | Improvement in esophageal lesions was found endoscopically in 44% of patients Group 1, in 50% of Group 2, and in 42% of cimetidine group | No adverse reactions were reported by any of the subjects. |
| Azizollahi 2016 | Iran | RCT | Pediatric patients (suspected GERD, 6.4±3.10 months Group A, 5.2±2.75 months in Group B) | 60 (30 in Group A, 60% ♂ 40% ♀; 20 in Group B, 66.7% ♂ 33.3% ♀) | Group A: Ranitidine (2–4 mg/kg/day) | Histamine H2 antagonists | Group B: Omeprazole (0.5 mg/kg/day) | Proton Pump Inhibitors (PPI) | GERD symptom questionnaire (GSQ) was 2.47±0.58 in Group A and in Group B 2.43±1.15 with (p = 0.98) after treatment compared to baseline GSQ (p = 0.57) | NR |
| Ballengee 2018 | USA | RCT | Premature infants (GERD, age NR) | 31 (16 in Placebo Group 44% ♂ 56% ♀; 15 in EES Group, 67% ♂ 33% ♀) | Group B: Erythromycin ethylsuccinate (EES, 50 mg/kg/day) | Other | Placebo | Not applicable (placebo) | Reflux events measured with impedance test decreased by 4 in the EES group to19(15–33) and by 10 events in the placebo group to 19 (11–26) per 24 hours (p=0.09). Apnea, bradycardia, or oxygen desaturation events decreased to 14 (4.75–19.5) in the EES group and to 10 (6–21) in the placebo group, which was not a statistically significant change (p=0.429). | NR |
| Barnett 2001 | Australia | Randomized cross-over trial | Premature infants (resistant feed intolerance, 33 ± 5 days) | 16 (9 in Group A, 7 in Group B; gender NR) | Cisapride (0.2 mg/kg four times per day) first, then placebo | Prokinetic agents | Placebo first, then Cisapride (0.2 mg/kg four times per day) | Prokinetic agents | Gastric emptying parameters did not differ for cisapride and placebo treatment periods (t1/2 31.9 ± 4.7 vs 34.2 ± 3.9 min for cisapride vs placebo, respectively; P=0.65) | NR |
| Bellissant 1997 | France | RCT | Infants (GER, 105 ± 74 days) | 39 (19 in Metoclopramide group, 20 in placebo group; 69.2% ♂, 30.8% ♀) | Metoclopramide (0.2 mg/kg three times daily before a meal) | Prokinetic agents | Placebo | Not applicable (placebo) | The percentage of time at pH <4 evaluated with pH-metry was 7.4% ± 10.4% (43 ± 26 in placebo group and 63 ± 136 in the Metoclopramide group). The numbers of reflux episodes >5 minutes, at pH <2, and at pH <4 wereodes >5 minutes, at pH <2, and at pH <4 were 2.5 ± 3.3, 16.0 ± 62.2, and 53 ± 96, respectively. | No adverse events recorded. |
| Bines 1992 | USA | RCT | Children (GERD, 5 months - 11.3 years) | 17 (8 in Domperidone group, 9 in placebo group; 71% ♂, 29% ♀) | Domperidone (0.6 mg/kg three times daily before a meal) | Prokinetic agents | Placebo | Not applicable (placebo) | Reflux episodes measured with pH-monitoring decreased >25% in 100% of children in Domperidone group and 12% in placebo group (p<0.01) | Diarrhea in 50% of Domperidone group and 22% of placebo group. Transient neutropenia in 12% of Domperidone group. |
| Boccia 2007 | Italy | RCT | Children and adolescents (reflux esophagitis, medianage105months, range32–170) | 46 (16 in Group A, 16 in Group B and 14 in Group C; 54% ♂, 46% ♀) | Group A: Omeprazole (1.4 mg/kg daily) | Proton Pump Inhibitors (PPI) | Group B: Ranitidine (10 mg/kg/day); Group C: No treatment | Histamine H2 antagonists | The endoscopic, histological, and symptomatic scores significantly decreased from baseline (T0) to healing phase (T1) and manteinance phase (T2)(P<0.0001 vs baseline, each). No statistically significant difference was found among groupA, groupB, and group C regarding the symptomatic, endoscopic, and histological scores at the end of the healing phase and 3 months after the end of the maintenance therapy. | NR |
| Carroccio 1994 | Italy | RCT | Children (GER, 1-18 months) | 80 (20 in Group A, 20 in Group B, 20 in Group C, 20 in Group D; 55% ♂, 45% ♀) | Group C: Domperidone (drops, 0.3 mg/kg dose, 15 minutes befor meals) + placebo | Prokinetic agents | 2 different kind of placebo | Not applicable (placebo) | In Group C patients there was a significant reduction in the number of reflux episodes (p<0.009) and in the Jolley score (p<0.04). In Group D patients was recorded a significant reduction only in the duration of the longest episode (p<0.05). | No patients in the four groups complained of adverse side effects during the treatment. |
| Clara 1979 | Belgium | Multicenter, double-blind, randomized, placebo-controlled trial | "Chronic excessive regurgitation or vomiting in infants and children Age range: 2.5 months – 10 years" | 32 children enrolled Domperidone Group: 14 children Placebo Group: 18 children Sex (%): Domperidone group, 71% female; placebo group, 83% female | Domperidone (Motilium®, R 33 812) Posology: 0.3 mg/kg per dose, three times daily (TID) before meals Dose doubled to 0.6 mg/kg per dose TID after two weeks if symptoms persisted | Dopamine receptor antagonist (prokinetic agent, antiemetic) | Placebo | Not applicable (placebo) | Global improvement at 4 weeks (investigator assessment): - Domperidone group: 93% showed good/excellent results - Placebo group: 33% showed good/excellent results (p < 0.001) Need for dose increase at 2 weeks (poor response with initial dose): - Domperidone group: 7/14 (50%) needed dose increase - Placebo group: 16/18 (89%) needed dose increase (p = 0.021) Symptom Resolution (Cure Rate, % of infants with severe symptoms improving to score 0-1): - Vomiting improvement at 2 weeks: Domperidone > Placebo (p < 0.05) - Regurgitation improvement at 2 weeks: Domperidone > Placebo (p < 0.05) - All symptoms improved significantly by 4 weeks in the domperidone group (p < 0.01) | No adverse events were reported in either group. No discontinuations due to side effects. No reports of extrapyramidal symptoms, drowsiness, or gastrointestinal intolerance. |
| Cohen 1999 | Australia | RCT | Infants (GERD, 7.3 ±5.9 months in intervention group, 8.3 ±7.0 months in the control group) | 95 (50 in intervention group, 45 in control group; 66% ♂, 34% ♀) | Cisapride (0.2 mg/kg 4 times per day) | Prokinetic agents | Placebo | Not applicable (placebo) | No significant differences could be detected in any symptom parameter between the cisapride and placebo groups. | Adverse events occurred in 84% of subjects in the cisapride group and 71% of subjects in the placebo group and in 30 of 38 subjects who completed cisapride and 22 of 30 subjects who received placebo (no significant difference at either time point). |
| Cresi 2008 | Italy | RCT | Preterm neonates (suspected GERD; 24.7 ± 13.7 in intervention group, 29.5 ± 7.4 days in placebo group) | 26 (13 in intervention group, 13 in control group; 54% ♂, 46% ♀) | Domperidone ( 0.3 mg per kg twice a day before meals) | Prokinetic agents | Intervention in control group NR | - | "The treatment group displayed significant increase in reflux frequency after treatment compared to baseline (4.06±1.16 vs 2.8±1.42; p=0.001), and a decrease in duration (16.68±4.49 vs 20.18±7.83; p=0.043)." | NR |
| Cucchiara 1989 | Italy | RCT | Children (GER; 37.6 ± 45.7 months) | 32 (17 in Cimetidine group, 15 in placebo group; 53% ♂, 47% ♀) | Cimetidine (30-40 mg/kg/day) | Histamine H2 antagonists | Placebo | Not applicable (placebo) | 70% patients in Cimetidine group and 20% in placebo group were healed (p < 0.01), the condition of 23% patients in cimetidine group and 20% in placebo group had improved (not statistically significant), and the condition of 6% patients in Cimetidine group and 60% in placebo group had worsened (p < 0.01). Both clinical and esophagitis scores significantly decreased only in the Cimetidine group, as compared with placebo group. Improvement of esophagitis was seen in all (100%) of Cimetidine patients with mild or moderate esophagitis versus 57.14% of placebo group (p < 0.01) and in 87.5% of Cimetidine group with severe esophagitis as compared with 25% of the placebo group (p < 0.01). | NR |
| Cucchiara 1993 | Italy | RCT | Children (GERD; 6 months-13 4 years) | 32 (16 in Omeprazole group, 16 in Ranitidine Group; 50% ♂, 50% ♀) | Omeprazole (40 mg/day/1*73 m2 surface area) | Proton Pump Inhibitors (PPI) | Ranitidine (20 mg/kg/day) | Histamine H2 antagonists | "Both therapeutic regimens were effective in decreasing clinical score (omeprazole before mean 24.0, after mean 9.0; ranitidine before mean 19.5, after 9.0, in improving the histological degree of oesophagitis (omeprazole before mean 8.0, after mean 2.0; ranitidine before mean 8.0, after mean 2.0), and in reducing oesophageal acid exposure,measured as minutes ofreflux at 24 hour pH monitoring (omeprazole before 129.4, after 44.6; ranitidine before 207.3, after 58.4) as well as intragastric acidity, measured as median intragastric pH (omeprazole before 2.1, after 5.1; ranitidine before 1.9, after 3.4)" | No serious adverse events to require discontinuing treatment and no abnormalities in the laboratory were observed. |
| Cucchiara 1987 | Italy | RCT | Children (GERD; 75 days-47 months) | 17 (8 in Cimetidine group, 9 in placebo group; 55% ♂, 45% ♀) | Cisapride (1 mg/ml at a dose of 0-3 mg/kg three times a day before meals) | Prokinetic agents | Placebo | - | Results were evaluated with clinical scores, pH monitoring and endoscopy before and after treatment and showed a statistically significant difference only in Cisapride group. 50% of patients on Cisapride and 11% on placebo were healed, 25% patients on Cisapride and 11% on placebo were considered to have improved, and 25% on Cisapride and 78% on placebo remained unchanged or worsened. | NR |
| Dalby-Payne 2003 | Not applicable | Systematic review | Children (GERD, 75 days - 5 years) | 10 RCT (415, gender NR) | Cisapride | Prokinetic agents | Placebo/Non pharmacological interventions | Not applicable (placebo) | Meta-analysis of the five trials with adequate data reported for inclusion, involving 156 children, showed that there was no significant reduction in vomiting frequency with cisapride treatment compared with placebo/non-pharmacological treatment. For studies measuring GER with pH monitoring, meta-analysis of these five studies, involving 176 children, demonstrated a benefit of cisapride treatment compared with placebo/non-pharmacological control on the reduction of the mean reflux index at final follow up. | " Seven studies reported adverse events or side effects. The adverse events or side-effects reported included fever, insomnia, nervousness, irritability, diarrhea, vomiting, eructations, cough, upper respiratory tract infection, and asthma. One study compared mean corrected QT(c) on electrocardiograph and found no significant difference between children receiving cisapride and placebo. Meta-analysis of six studies17–20,22 providing adequate data also demonstrated no increased reporting of these events with cisapride treatment (RR 1.16; 95% CI 0.95, 1.41; Q 3df=1.1; P=0.78)." |
| Davidson 2013 | Australia, Germany, United Kingdom | RCT | Neonates (suspected GERD; 48.1 ± 29.8 days in esomeprazole group; 46.5 ± 31.2 days in placebo group) | 52 (25 in esomeprazole group; 26 in placebo group; 40% ♂, 60% ♀) | Esomeprazole (0.5 mg/kg once daily) | Proton Pump Inhibitors (PPI) | Placebo | Not applicable (placebo) | There was no statistically significant difference between the esomeprazole and placebo groups in the percentage of change from baseline after 14 days of treatment in the total number of GERD-related signs and symptoms observed by video and cardiorespiratory monitoring ( - 14.7% vs - 14.1%, respectively; 95% CI: - 14.2% - 14.9%; p = 0.92) | Adverse events occurred at similar rates between groups. No serious adverse events (SAEs) in the esomeprazole group. |
| De Loore 1979 | Belgium | RCT | Children (frequent regurgitation; 3 weeks - 8 years) | 47 (15 in Domperidone Group, 17 in Metoclopramide group, 15 in placebo group; 49% ♂ 51% ♀) | Domperidone (0.3 mg/kg 3 times daily); Metoclopramide (0.3 mg/kg 3 times daily) | Prokinetic agents | Placebo | Not applicable (placebo) | Statistically significant improvement in nausea was recorded only in the two active treatment groups compared to placebo group (p<0.001), but there was no statistical difference between the two active treatment groups. | NR |
| Euler 1980 | USA | Randomized cross-over trial | Children (GERD; 3 weeks -13 years) | 45 (gender NR) | Bethanechol (2.9 mg/m^2) first, then placebo | Other | Placebo first, then Bethanechol (2.9 mg/m^2) | - | All participants symptoms decreased during the intervention phase and returned during placebo phase. During esophageal pH probe testing, there was a significant difference in the number of episodes of gastroesophageal reflux per hour before and after the oral administration of bethanechol (x + SEM = 5.8 _+ 1.1 vs 1.8 _+ 1.3; P < 0.01). The duration of the episodes was also shorter after betbanechol was given (4.3 _+ 1.2 vs. 1.5 _+ 1.3; P < 0.01). | 2% had dizziness and headaches during the first week of administration |
| Fallahi 2008 | Iran | RCT | Adolescents (GERD, 13-20 years) | 36 (gender NR) | Omeprazole (20 mg twice a day) | Proton Pump Inhibitors (PPI) | Placebo | Not applicable (placebo) | The symptoms of GERD were significantly improved with omeprazole after 6 weeks in the intervention group. Heartburn was resolved in 97% of patients (p 0.040). No case experienced regurgitation after treatment (p 0.019) | NR |
| Forbes 1986 | Australia | RCT | Children and adolescents (GER; 4 months - 17 years) | 30 (10 in Metoclopramide group, 10 in Gaviscon Liquid Infant, 10 in placebo group; gender NR) | Metoclopramide (0.5 mg/kg divided in 3 times daily before a meal) | Prokinetic agent | Placebo | Not applicable (placebo) | Metoclopramide did not decrease the number of episodes of GER or the total duration of GER during pH monitoring. The placebo group had a decline in the total number of episodes of GER. | No side effects were observed. |
| Gremse 2019 | Multinational (USA, Poland, Portugal, Mexico) | Phase 2, multicenter, randomized, double-blind, placebo-controlled study | "Erosive esophagitis (EE) in adolescents with gastroesophageal reflux disease (GERD) Mean Age ± SD: 14.8 ± 1.64 years (60% aged 15–17)" | 62 adolescents (Dexlansoprazole 60 mg open label: 62; Maintenance phase: Dexlansoprazole 30 mg: 25, Placebo: 26) Sex (%): 61.3% Male (38/62) | Dexlansoprazole Posology: 60 mg once daily (open-label healing phase); 30 mg once daily (maintenance phase) | Proton Pump Inhibitor (PPI) | Placebo (during maintenance phase) | Not applicable (placebo) | EE healing achieved in 88% of patients after 8 weeks of dexlansoprazole 60 mg. Maintenance of healed EE observed in 82% with dexlansoprazole 30 mg and 58% with placebo after 16 weeks. Median percentage of days without heartburn: 86.6% (Dexlansoprazole) vs. 68.1% (Placebo). | Most treatment-emergent adverse events (TEAEs) were mild to moderate. Common TEAEs: Headache (12.9%), abdominal pain (12.0%), nasopharyngitis (12.0%), pharyngitis (12.0%), sinusitis (12.0%). TEAEs were reported by 72% of the Dexlansoprazole group and 61.5% of the placebo group in the maintenance phase. |
| Gustafsson 1992 | Sweden | Randomized, double-blind, placebo-controlled, cross-over study | "Bronchial asthma with or without pathological gastro-oesophageal reflux (GOR) Mean Age ± SD: 14 years (range: 9.5–20.8 years)" | 37 children and adolescents (18 with pathological GOR, 19 with normal GOR) Sex (%): 59% Male (22/37) | Ranitidine Posology: 150 mg for patients <40 kg, 300 mg for patients ≥40 kg, administered as a single evening dose for 4 weeks | H2-receptor antagonist | Placebo | Not applicable (placebo) | Modest reduction (30%) in nocturnal asthma symptoms in patients with pathological GOR compared to those with normal GOR. Significant correlation between asthma symptom improvement and degree of acid reflux (r=0.46; p<0.01). No significant changes in lung function (FEV1 or PEFR) or morning asthma symptoms. | Mild side effects in 3 patients during the ranitidine period (loose stools, abdominal pain, morning fatigue). No systematic or significant changes in blood chemistry or urinalysis between treatment periods. |
| Hibbs 2006 | Not applicable | Systematic review | "Gastroesophageal reflux disease (GERD) in infants Mean Age: Varies by study, ranging from 0–24 months” | 6 RCTs included Sample size: range 10-41 infants | Metoclopramide Dosing range: 0.1 - 1 mg/kg per dose Dosing frequency: QID (4x/day), TID (3x/day), or single dose in some studies | Dopamine receptor antagonist (prokinetic agent) | Placebo | Not applicable (placebo) | "- 1 RCT (Leung & Lai, 1984) found a reduction in vomiting and improved acid clearance. - 2 RCTs (Hyman 1985, 1988) found improved gastric emptying. - 3 RCTs (Tolia 1989, Pons 1993, Bellissant 1997) found no benefit. Placebo effects were observed in multiple trials.” | Most RCTs did not systematically assess adverse events. Bellissant 1997 reported apnea, emesis, and irritability in the metoclopramide group. |
| Hussain 2014 | Multinational (10 countries including the USA, Poland, Belgium, South Africa) | Randomized, double-blind, placebo-controlled withdrawal trial with an open-label (OL) lead-in phase | "Symptomatic gastroesophageal reflux disease (GERD) in infants Mean Age ± SD: 4.7 ± 2.65 months (1–11 months old)" | 344 infants entered OL phase, 268 randomized into double-blind (DB) phase (90 in placebo, 90 in rabeprazole 5 mg, 88 in rabeprazole 10 mg) Sex (%): 65% Male in combined rabeprazole-treated groups (115/178), 53% Male in placebo (48/90) | Rabeprazole Posology: 5 mg or 10 mg, once daily, delivered via sprinkle capsules with food/formula | Proton Pump Inhibitor (PPI) | Placebo (identical capsules) | Not applicable (placebo) | No significant differences between placebo and combined rabeprazole groups in the frequency of regurgitation, weight-for-age z-scores, or GERD symptom scores during the DB phase. Both placebo and rabeprazole groups showed continued symptom improvement after the OL phase, suggesting natural resolution or placebo effect. | Treatment-emergent adverse events (TEAEs) reported by 47% in both placebo and combined rabeprazole groups. Common TEAEs: pyrexia (7%), upper respiratory infections (5%), and increased serum gastrin levels (5%). Serious adverse events (SAEs) occurred in 4.5% of the rabeprazole-treated group and 2.2% of the placebo group, mainly infection-related. |
| Levy 2001 | USA (Multicenter study) | Randomized, double-blind, placebo-controlled | "Gastroesophageal reflux (GER) not responding to medical therapy for at least 6 weeks Mean Age: 14.4 (range: 6 months to 4 years)" | 68 enrolled; 49 analyzed (cisapride: 24; placebo: 25) Sex (%): Not reported | Cisapride Posology: 0.2 mg/kg three times daily, administered 30 minutes before meals | Prokinetic agent | Placebo (matched) | Not applicable (placebo) | No significant differences between placebo and cisapride in terms of cardiac QTc intervals after 3–8 weeks of treatment. Mean QTc intervals: 408 ± 18 ms (cisapride) vs. 399 ± 21 ms (placebo). Changes from baseline were also nonsignificant. | No significant cardiac abnormalities or arrhythmias were observed in either group. QTc intervals remained within normal ranges (<450 ms) in all subjects. Study emphasized the drug's safety in patients without cardiac or electrolyte abnormalities but noted limitations due to small sample size. |
| Machida 1988 | Canada | Randomized, double-blind, placebo-controlled trial | "Gastroesophageal reflux (GER) in infants Mean Age ± SD: 9 ± 11 months" | 28 infants Sex (%): 20 males (71%), 8 females (29%) 8 infants entered in the double-blind trial: 3 in the metoclopramide group and 5 in the placebo group. | "Metoclopramide Posology: Single intravenous dose: 0.125 mg/kg Oral administration: 0.125 mg/kg per dose, QID (four times daily) for 24 hours” | Dopamine receptor antagonist (prokinetic agent) | Placebo | Not applicable (placebo) | Parental assessment of improvement: Metoclopramide: 0% (none of the 3 infants improved, all worsened) Placebo: 80% (4 out of 5 infants improved) | Adverse Effects in the Metoclopramide Group (n = 3): - Marked irritability in all 3 infants (p = 0.01 vs. placebo) - Increased vomiting - All 3 infants in the metoclopramide group were withdrawn due to worsening GER symptoms and adverse effects Adverse Effects in the Placebo Group (n = 5): No significant adverse effects reported |
| Maclennan 2010 | Not applicable | Systematic Review of Randomized Controlled Trials (RCTs) | "Gastroesophageal reflux (GER) in children Age Range: 5 days – 5 years (varied across included studies)" | 10 RCTs Sample size: range 6 to 134 participants | Cisapride Posology: Most studies used 0.8 mg/kg/day Some variations: 0.45 – 0.9 mg/kg/day, divided into multiple daily doses | Prokinetic agent | - Placebo (9 studies) - Gaviscon (1 study - Greally 1992) | Not applicable (placebo) - Alginate-Antacid Combination | No significant improvement in GER symptoms compared to placebo: Odds Ratio (OR): 0.34 (95% CI: 0.10–1.19), p = NS Parental evaluation in 5 trials showed no consistent symptom relief. Reduction in reflux index (pH < 4) compared to placebo: Weighted Mean Difference (WMD): -6.49 (95% CI: -10.13 to -2.85), p = 0.0005 However, other pH-monitoring parameters did not show significant differences. No significant difference in weight gain (2 studies reported weight data, no benefit for cisapride). No significant benefit when compared with dietary interventions (thickened feeds). No difference between cisapride and Gaviscon in symptom resolution (Greally 1992). | Adverse Events: 4 studies (190 participants) reported adverse events. Diarrhea was the most common AE, but the difference was not statistically significant (OR: 1.86, 95% CI: 0.88–3.93). No significant difference in QTc prolongation (Levy 2001, n=134). Severe Safety Concern: Cisapride was linked to fatal cardiac arrhythmias and sudden death in post-marketing surveillance. Due to safety concerns, cisapride was withdrawn from the market in the USA and Europe in 2000. |
| Moore 2003 | Australia | Randomized, double-blind, placebo-controlled, crossover study | "Irritability in infants with gastroesophageal reflux (GER) and/or esophagitis Mean Age ± SD: 5.4 ± 2.1 months" | 30 infants (15 started with omeprazole, 15 with placebo) Sex (%): 76.7% Male (23/30) | Omeprazole Posology: 10 mg daily for infants 5–10 kg; 10 mg twice daily for infants >10 kg, administered as microspheres with applesauce | Proton Pump Inhibitor (PPI) | Placebo | Not applicable (placebo) | Reflux Index (RI): Significant reduction in esophageal acid exposure with omeprazole (mean RI change: -8.9% ± 5.6%) compared to placebo (-1.9% ± 2.0%; p<0.001). Cry/Fuss Time: No significant difference between omeprazole and placebo (191 ± 120 min/day vs. 201 ± 100 min/day; p=0.400). Irritability improved over time in all infants, regardless of treatment, suggesting self-resolution of symptoms. | No adverse events reported for either omeprazole or placebo. Treatment was well tolerated, and there were no reports of serious side effects or withdrawals due to safety concerns. |
| Omari 2007 | Australia | Randomized, double-blind, placebo-controlled, crossover study | "Pathological acid gastroesophageal reflux (GER) in preterm infants Mean Postmenstrual Age (PMA): 36.1 ± 0.7 weeks (range: 34–40 weeks) Mean Weight ± SD: 2217 ± 112 g (range: 1810–2700 g)" | 10 preterm infants Sex (%): Not specified | Omeprazole Posology: 0.7 mg/kg/day, administered via nasogastric tube with an antacid solution (Mylanta) | Proton Pump Inhibitor (PPI) | Placebo (sterile water as the vehicle) | Not applicable (placebo) | Significant reductions in gastric acidity (%time pH<4: 54% placebo vs. 14% omeprazole; p<0.0005). Reduction in esophageal acid exposure (%time pH<4: 19% placebo vs. 5% omeprazole; p<0.01). Decrease in acid GER episodes (119 episodes placebo vs. 60 episodes omeprazole; p<0.05). | No significant changes in blood biochemistry or complete blood picture between omeprazole and placebo. No serious adverse events reported. Symptom frequency (vomiting, apnea, bradycardia, choking, and behavioral changes) did not significantly differ between groups. |
| Orenstein 2009 | Multinational (United States, Poland) | Multicenter, double-blind, randomized, placebo-controlled trial | Symptoms attributed to gastroesophageal reflux disease (GERD) in infants aged 1–12 months  Mean age: 16 weeks (range: 4–51 weeks) | 162 infants randomized (81 lansoprazole, 81 placebo) Sex (%): 50% Male | Lansoprazole Posology: 0.2–0.3 mg/kg/day (for infants ≤10 weeks old), 1.0–1.5 mg/kg/day (for infants >10 weeks old) | Proton Pump Inhibitor (PPI) | Placebo (identical formulation without active ingredient) | Not applicable (placebo) | Primary Outcome: No difference in responder rates between lansoprazole and placebo groups (54% in both). Secondary Outcomes: No significant difference in reduction of feeding-associated crying, regurgitation, or other GERD symptoms. Global assessments by parents and investigators also showed no significant differences. | Treatment-emergent adverse events (TEAEs) were reported in 62% of lansoprazole-treated infants vs. 46% of placebo (p = 0.058). Serious adverse events (SAEs), particularly lower respiratory tract infections, were significantly more frequent in lansoprazole (10 events) compared to placebo (2 events; p = 0.032). No deaths occurred, and no clinically significant laboratory findings were reported. |
| Orenstein 2003 | USA | Multi-center, randomized, placebo-controlled, withdrawal trial | Gastroesophageal reflux disease (GERD) in infants  Age Range: 1.3 – 10.5 months  Mean Age: 5.3 months (Median) | 35 infants enrolled Part I (4-week observer-blind phase, famotidine dose comparison): 27 completed Part II (4-week double-blind placebo withdrawal phase): 8 infants complete Sex (%): 57% female, 43% male | Famotidine Posology: 0.5 mg/kg per dose (once daily if <3 months old, twice daily if ≥3 months old) 1.0 mg/kg per dose (same administration schedule) | Histamine-2 receptor antagonist (H2RA) | Placebo | Not applicable (placebo) | Famotidine 0.5 mg/kg: - Significant reduction in regurgitation frequency compared to baseline (p = 0.04) Famotidine 1.0 mg/kg: - Significant reduction in regurgitation frequency (p = 0.004) - Significant reduction in regurgitation volume (p = 0.01) -Reduction in crying time (p = 0.027) Double-blind withdrawal phase (Part II, placebo vs continued famotidine treatment): - Sample size too small (n=8) for meaningful comparisons | No serious adverse events reported. 11 infants experienced 16 non-serious, possibly drug-related adverse events: - Agitation/Irritability (n=6) (two with head-rubbing, possibly indicating headache) - Somnolence (n=3) - Anorexia (n=2) - Headache (n=2) - Vomiting (n=1) - Hiccups (n=1) - Oral candidiasis (n=1) Adverse event-related discontinuation: - 1 infant discontinued due to agitation No significant impact on growth or laboratory values |
| Saye 1987 | Belgium | Double-blind, randomized, cross-over pH-monitoring study | Chronic bronchopulmonary disease (CBPD) with suspected gastroesophageal reflux (GER)  Mean Age (Range): Median 29 months (range: 4 months to 11 years) | 14 children (3 boys, 11 girls) | Cisapride Posology: Initial dose of 0.3 mg/kg orally, followed by 0.15 mg/kg every 4 hours for 12 hours | Gastrointestinal prokinetic agent | Placebo | Not applicable (placebo) | Significant reduction in GER variables with cisapride compared to placebo: - Percentage of time with pH <4 reduced by 60% over 24 hours and 80% during sleep. - GER episodes lasting ≥5 minutes reduced by 64% overall and 92% during sleep. - Number of GER episodes reduced by 21% over 24 hours and 55% during sleep. - Longest GER episode reduced by 60% over 24 hours. Marked improvements were observed in patients with severe GER, with near-normal pH metrics achieved in most cases. | No adverse effects were reported during cisapride administration. |
| Scott 1997 | Multinational (Canada and Europe) | Randomized, double-blind, placebo-controlled study | Gastroesophageal reflux disease (GERD) in infants  Cisapride group: 8.4 ± 3.2 months; Placebo group: 8.3 ± 4.2 months | 45 evaluable patients (21 cisapride; 24 placebo) Sex (%): Cisapride: 57% Male (12/21); Placebo: 50% Male (12/24) | Cisapride Posology: 0.2 mg/kg every 6 hours for 6 weeks | Prokinetic agent | Placebo (identical suspension) | Not applicable (placebo) | Significant decrease in average duration of longest reflux episode with cisapride compared to placebo (62 ± 61 min vs. 85 ± 132 min; p = 0.035). Significant reduction in duration of supine reflux (p = 0.030). No significant differences between cisapride and placebo for percentage time pH <4, number of reflux episodes, or global evaluations. Parental global response rates ("good" or "excellent"): Cisapride 52.4%, Placebo 54.6%. | Adverse events reported in 60.8% (cisapride) and 50.0% (placebo). Common events: diarrhea, fever, upper respiratory infections, all mild to moderate. No significant differences in adverse events or laboratory findings between groups. |
| Simeone 1997 | Italy | Randomized, double-blind, placebo-controlled trial | Peptic esophagitis in children with reflux esophagitis  Median age (range): Nizatidine group: 2.08 years (range: 0.5–12 years); Placebo group: 1.16 (0.5-9.5 years) | 26 enrolled, 24 completed the 8-week protocol (13 nizatidine, 11 placebo) Sex (%): Nizatidine: 69.2% Male (9/13); Placebo: 61.5% Male (8/13) | Nizatidine Posology: 10 mg/kg/day in two doses, administered as an oral solution (15 mg/mL) for 8 weeks | H2-receptor antagonist | Placebo (matching solution) | Not applicable (placebo) | Healing rate: Nizatidine group: 9/13 (69%) vs. Placebo group: 2/13 (15%) (p < 0.007). Significant improvement in esophageal pH metrics (total acid exposure, number of reflux episodes, etc.) observed only in the nizatidine group (p < 0.01). Clinical score improvements were significant for all symptoms in the nizatidine group (p < 0.01), except for vomiting, which improved in both groups. | One patient (nizatidine group) discontinued due to urticarioid rash. No significant differences in hematology or biochemistry parameters between groups. No severe adverse events reported. |
| Størdal 2005 | Norway | Randomized, double-blind, placebo-controlled trial | Asthma and gastro-oesophageal reflux disease (GORD) in children  Mean Age: 10.8 years (range: 7.2–16.8 years) | 38 randomized (omeprazole: 19; placebo: 19) Sex (%): 29 males (76%) | Omeprazole Posology: 20 mg daily for 12 weeks | Proton Pump Inhibitor (PPI) | Placebo | Not applicable (placebo) | No significant improvement in asthma symptoms or quality of life (measured by PAQLQ) between omeprazole and placebo groups. Mean change in asthma symptom score: -1.28 (omeprazole) vs. -1.28 (placebo) (p = 1.00). PAQLQ improvement: 0.62 (omeprazole) vs. 0.50 (placebo) (p = 0.51). Acid suppression confirmed effective (reflux index <5) in 7/8 omeprazole-treated patients but not in the placebo group. Spirometry values (FEV1% and FEF25-75) and use of bronchodilators showed no significant differences. | Two withdrawals due to adverse effects: one with headache (omeprazole group), one with worsened asthma (placebo group). |
| Tighe 2023 | Not applicable | Systematic review | Infants and children with gastro-oesophageal reflux disease (GORD)  Nineteen studies assessed infants only, six studies assessed infants and children, and 11 assessed children aged one year or older. | 36 RCTs involving 2251 children and infants of both sexes | Main comparisons Infants: - Omeprazole versus placebo - Omeprazole versus ranitidine - Esomeprazole versus placebo Children: - Rabeprazole given at dfferent doses (0.5 mg/kg and 1 mg/kg) - Pantoprazole given at dfferent doses (0.3 mg/kg, 0.6 mg/kg, and 1.2 mg/kg) | Proton Pump Inhibitor (PPI) | Placebo, other drugs or different doses (see Intervention) | Not applicable (placebo); H2-receptor antagonist; PPI | Infants: - Omeprazole versus placebo: there is no clear effect on symptoms from omeprazole (one study, 30 infants; very low-certainty evidence); - Omeprazole versus ranitidine: one study (76 infants; very low-certainty evidence) showed omeprazole may or may not provide symptomatic benefit equivalent to ranitidine; - Esomeprazole versus placebo: esomeprazole appeared to show no additional reduction in the number of GORD symptoms compared to placebo (1 study, 52 neonates; very low certainty evidence). Children: - Rabeprazole given at different doses (0.5 mg/kg and 1 mg/kg) may provide similar symptom improvement (127 children in total; very low-certainty evidence); - Pantoprazole may or may not improve symptom scores at 0.3 mg/kg, 0.6 mg/kg, and 1.2 mg/kg in children aged one to five years (one study, 60 children; very low-certainty evidence). | Infants: - Omeprazole versus placebo: no adverse events in both groups; - Omeprazole versus ranitidine: insufficient data described in the included studies; - Esomeprazole versus placebo: insufficient data described in the included studies. Children: - Rabeprazole given at different doses (0.5 mg/kg and 1 mg/kg): is likely to give some adverse events, based on a single study (1 study, 127 children; very low-certainty evidence): Haddad 2013 noted 95 (84%) children had adverse events, including abdominal pain, nausea, vomiting, bronchopneumonia, gastroenteritis, cough, and choking ; - Pantoprazole may or may not improve symptom scores at 0.3 mg/kg, 0.6 mg/kg, and 1.2 mg/kg: Three studies reported data on adverse events, the more common adverse events were headache, diarrhoea, rhinitis, nausea. |
| Tolia 1989 | USA | Randomized prospective, double-blind, placebo-controlled, crossover trial | Gastroesophageal reflux (GER) in infants  Mean Age ± SD: Median age: 2 months (Range: 1–9 months) | 30 infants Group A (Metoclopramide first, then placebo): 15 infants Group B (Placebo first, then metoclopramide): 15 infants Sex (%): 17 males (56.7%), 13 females (43.3%) | Metoclopramide Posology: 0.1 mg/kg per dose, four times a day (QID), 30 minutes before feeding | Dopamine receptor antagonist (prokinetic agent) | Placebo | Not applicable (placebo) | "Symptom scores significantly improved during placebo compared to pretreatment (p < 0.005), suggesting a strong placebo effect. No significant difference in symptom score between placebo and metoclopramide periods. Metoclopramide significantly reduced the percentage of time esophageal pH was <4.0 (p < 0.001), but did not normalize it. No significant difference between metoclopramide and placebo in: - Total number of reflux episodes - Number of episodes lasting >5 minutes No significant difference in gastric emptying rate between metoclopramide and placebo. Infants older than 3 months had greater weight gain with metoclopramide (34.3 g/day) vs. placebo (6.6 g/day, p = 0.05).” | No reported adverse events in either the metoclopramide or placebo group. |
| Ummarino 2012 | Italy | Prospective, randomized, open-label, comparative study | Patients presenting both esophageal and extraesophageal GERD symptoms  Mean Age ± SD: 40.6 ± 36.4 months (range: 1–181 months) | 35 children (19 treated with PPIs, 16 with H2RAs) Sex (%): Not reported | Proton Pump Inhibitor (PPI: Omeprazole) Posology: 1.4 mg/kg/day for 12 weeks | Proton Pump Inhibitor (PPI) | H2-receptor antagonist (H2RA: Ranitidine) Posology: 15 mg/kg/day for 12 weeks | H2-receptor antagonist | Complete resolution of symptoms: 57.9% in PPI group vs. 31.2% in H2RA group after the first 3 months (p < 0.05). After switching non-responders from H2RA to PPI: 7/10 achieved complete resolution. Symptom score significantly improved in the PPI group compared to the H2RA group for vomiting (p = 0.0003), cough (p = 0.0001), and overall respiratory symptoms (p < 0.000001). | No adverse events reported in either group. |
| Van Eygen 1989 | Belgium | Randomized, double-blind, placebo-controlled trial (three-phase study: open-label, placebo-controlled, and dose-response) | Infants <12 months old with regurgitation or vomiting at least twice a day  Median age: 3.5 months (25th percentile: 2 months; 75th percentile: 6 months) | 137 infants (Trial I: 69; Trial II: 23, 12 cisparide and 11 placebo; Trial III: 45) Sex (%): 70 boys (51%), 67 girls (49%) | Cisapride Posology: 0.1–0.2 mg/kg three times daily (TID) for 4 weeks | "Gastrointestinal prokinetic agent” | Placebo (Trials II and III) | Not applicable (placebo) | Placebo-Controlled Trial (Trial II): - Significant reduction in severity and frequency of regurgitation with cisapride compared to placebo (p = 0.05 and p = 0.03, respectively). - 83% of cisapride-treated patients showed improvement vs. 55% on placebo. | Mild gastrointestinal side effects reported (e.g., diarrhea, abdominal cramps in 2–4% of cases). No severe adverse events observed. |
| Vandenplas 1991 | Belgium | Randomized, double-blind, placebo-controlled trial | Gastroesophageal reflux (GER) in infants  Mean Age: 2–4 months | 42 infants randomized Cisapride Group: 21 infants Placebo Group: 21 infants | Cisapride Posology: 0.2 mg/kg per dose, four times a day (QID), 15 minutes before feeding | Prokinetic agent | Placebo | Not applicable (placebo) | Cisapride significantly reduced reflux episodes lasting >5 minutes compared to placebo (p < 0.01). All esophageal pH monitoring parameters improved significantly in the cisapride group (p < 0.05 - 0.001). No significant reduction in total reflux episodes in the placebo group. | No adverse events reported. |
| Winter 2012 | Multinational (United States, France, Germany, and Poland) | Multicenter, randomized, double-blind, placebo-controlled, parallel-group, treatment-withdrawal study | Gastroesophageal reflux disease (GERD) in infants aged 1–11 months  Mean Age ± SD: 4.8 ± 2.9 months (range: 1–11 months) | 98 infants enrolled; 80 randomized (39 esomeprazole, 41 placebo) Sex (%): 64.3% Male (63/98 overall) | Esomeprazole Posology: 2.5–10 mg/day, weight-adjusted, once daily for 4 weeks | Proton Pump Inhibitor (PPI) | Placebo | Not applicable (placebo) | No significant difference in discontinuation rates due to symptom worsening: 38.5% in esomeprazole group vs. 48.8% in placebo group (p = 0.28). Subgroup analysis showed significant benefit in infants with symptomatic but unverified GERD (HR 0.24; p = 0.01). Symptom improvement observed in 83% during the open-label phase, particularly in vomiting and irritability scores. | Adverse events (AEs) during the double-blind phase: 59% esomeprazole group vs. 66% placebo group. Common AEs: Upper respiratory tract infections (esomeprazole: 15.4%, placebo: 9.8%), diarrhea, rhinitis, pyrexia, nasopharyngitis. No treatment-related serious adverse events reported. |
| Winter 2010 | Multinational (United States, South Africa, and Poland) | Multicenter, randomized, double-blind, placebo-controlled, treatment-withdrawal study | Symptomatic gastroesophageal reflux disease (GERD) in infants aged 1–11 months  Mean Age ± SD: 5.1 ± 2.81 months | 106 infants (Pantoprazole: 52; Placebo: 54) Sex (%): 64.3% Male | Pantoprazole (delayed-release granules for oral suspension) Posology: 1.2 mg/kg/day (5 mg for infants weighing 2.5–7 kg, 10 mg for 7–15 kg) for 4 weeks (OL phase), followed by 4 weeks in DB phase | Proton Pump Inhibitor (PPI) | Placebo | Not applicable (placebo) | No significant difference in withdrawal rates due to lack of efficacy during DB phase (Pantoprazole: 11.5%; Placebo: 11.2%). Weekly GERD symptom scores (WGSSs) significantly improved during OL phase (p < 0.001) and remained stable in both groups during DB phase. Pantoprazole showed significant improvement in reducing arching back episodes during DB phase compared to placebo (p = 0.028). | Treatment-emergent adverse events (TEAEs): 63% in Pantoprazole group, 62% in Placebo group. Common TEAEs: Upper respiratory infections, rash, diarrhea, and fever. Serious adverse events (SAEs) occurred in 8 patients, but none were treatment-related. |
| Yagoubi 2022 | Algeria | Prospective, randomized, controlled trial | Poorly controlled asthma with gastroesophageal reflux (GER) in children aged 4–16 years  Mean Age : 88.4 months (PPI group); 88.7 months (no treatment group) | 59 children with GER and poorly controlled asthma (PPI group: 33; control group: 26) Sex (%): 72.7% Male in the PPI group; 65.4% Male in the control group | Omeprazole Posology: 1 mg/kg/day for 6 months; increased to 2 mg/kg/day in patients with persistent GER after 6 months | Proton Pump Inhibitor (PPI) | No anti-reflux treatment | Not applicable (no treatment) | Asthma control significantly improved in the PPI group (66.7%) compared to the control group (11.5%) after 6 months (p < 0.001). GER resolved in 77.3% of treated patients, with further improvement after dose escalation (84.8% asthma control achieved). Temporal relationship between GER and asthma not confirmed by pH-metric analysis (Symptom Association Probability <95%). | No adverse events reported. |
| Abbreviations AE: Adverse events; CBPD: Chronic bronchopulmonary disease ; DB: Double blind; EES: Erythromycin ethylsuccinate ; FEF: Forced expiratory flow; FEV: Forced Expiratory Volume; GER: Gastroesophageal reflux ; GERD: Gastroesophageal reflux disease ; GI: Gastrointestinal; GOR: Gastro-oesophageal reflux; GORD: Gastro-oesophageal reflux disease; GSQ: Guilt sensitivity questionnaire; I-GERQ-R: Infant Gastro-Esophageal Reflux Questionnaire Revised ; NR: Not reported; OL: Open label; OR: Odds Ratio; PAQLQ: Pediatric asthma quality of life questionnaire; PPI: Proton pump inhibitors ; RCT: Randomized controlled trial; SAEs: Serious adverse events ; SD: Standard deviation; SEM: Scanning electron microscopy; TEAEs: Treatment-emergent adverse events ; TID: Three times daily ; WMD: Weighted mean difference. | | | | | | | | | | |

### PICO 6 - What is the effectiveness of different non-pharmacological treatment options for GER and GERD in infants, children, and adolescents?

| Study ID | Country | Study Design | Population (condition, age) | Sample Size (N, sex %) | Intervention | Intervention group (Dietary modifications; Positioning therapy; others) | Comparator | Comparator group (Dietary modifications; Positioning therapy; others) | Efficacy Outcomes | Safety Outcomes |
| --- | --- | --- | --- | --- | --- | --- | --- | --- | --- | --- |
| Baldassarre 2019 | Italy | Randomized cross-over trial | Pediatric patients (suspected GERD, 51.8±41.1 days Group A, 84.4±57.2 days in Group B) | 53 (27 in Group A, 26 in Group B; gender NR) | Group A: Magnesium-Alginate (1 mL/kg/day) first, then Thickened Formula (150 mL/kg/day) | Alginates | Group B: Thickened Formula (150 mL/kg/day) first, then Magnesium-Alginate (1 mL/kg/day) | Alginates | A significant variation of Infant Gastro-Esophageal Reflux Questionnaire Revised (I-GERQ-R) scores over time (visits 2–3–4–5) (F = 55.387; p < 0.001) were evident, independent of the sequence of administration (magnesium-alginate—thickened formula or thickened formula—magnesium-alginate) (interaction e ect: F = 0.268; p = 0.848) | NR |
| Baldassarre 2022 | Italy | RCT | Infants (GERD; age NR) | 960 (499 in Group A, 461 in Group B; gender NR) | Group A: probiotic BB-12 (ABINAT12®) was administered once daily (six drops = 1x109 CFU) | Probiotics | No treatment | - | At baseline, 25.8% in Group A and 31.7% in Group B responded positively to the e Infant Gastroesophageal Reflux Questionnaire-Revised (I-GERQ-R). At T1, 16% in Group A and 45.8% in Group B (p<0.001) had a positive I-GERQ-R. At T2, 14.7% in Group A and 50.7% in Group B (p<0.001) had a positive I-GEQ-R. Consistently, the total scores significantly decreased in Group A. | NR |
| Bellaiche 2021 | Poland, France, Germany | RCT | Infants (uncomplicated regurgitation, 21-91 days) | 182 (92 Test group, 90 control group; gender NR) | Novel anti-regurgitation formula (0.4g/ 100 mL LBG, 0.4g/100mL scGOS/lcFOS (ratio 9:1) and 26% fermented formula with postbiotics derived from the LactofidusTM fermentation process, including 30-galactosyllactose) | Dietary modification | Regular anti-regurgitation formula (0.4g/ 100mL LBG and 11% fermented formula with postbiotics) | Dietary modification | IGSQ (Infant Gastrointestinal Symptom Questionnaire) sum scores improved from on average 36 at Baseline to 23 at Week 4 (Fig. 2), which was statistically significant after 1 week of intervention (P<0.001). There was no significant difference between groups at any time point. | The number of subjects with any AE was relatively higher in the Control versus Test group (n=33 [37.1%] vs n=29 [31.5%]; P=0.439). In the Test group, three serious AEs (pneumonia due to Respiratory Syncytial Virus, upper respiratory tract infection, and exanthema) were reported in three subjects (3.3%). In the Control group, eight serious AEs(pylorus hypertrophy,difficulty in feeding, gastroesophageal reflux disease without esophagitis, bronchitis, bronchiolitis, pneumonia (two cases), and skull fracture) were reported in seven subjects (7.9%). There were more skin disorders (dermatitis, eczema, miliaria, rash, skin irritation) in the Test versus Control group (n=9 [9.8%] vs n=1 [1.1%]; Fisher exact test, P=0.018) . |
| Borrelli 1997 | Italy | Randomized cross-over trial | Infants (frequent regurgitation; 5-11 months) | 24 (67% ♂, 33% ♀) | Anti-regurgitation thickened formula (Nutrilon AR) with a casein/whey ratio similar to cow's milk containing carob flour as a thickening agent first and then traditional formula thickened with rice flour at a concentration of 5% | Dietary modification | Traditional formula thickened with rice flour at a concentration of 5% first and then Anti-regurgitation thichened formula (Nutrilon AR) with a casein/whey ratio similar to cow's milk containing carob flour as a thickening agent | Dietary modification | Intraoesophageal acid exposure was significantly lower in the periods following the new formula than after traditional formula; at the end of the treatment period patients receiving the new formula had a more significant decrease of both symptomatic score and number of episodes of emesis than patients on traditional formula. | NR |
| Buts 1987 | Belgium | RCT | Children (GER, 2-144 months) | 20 (10 in intervention group, 10 in placebo group; 45% ♂, 55%♀) | Sodium alginate (Gaviscon, posology NR) | Alginates | Placebo | Not applicable (placebo) | All the pH monitoring variables were significantly (P< 0.05) reduced (from - 35% to - 61%) after 8 days of Gaviscon treatment, while in the placebo group the mean values of the pH monitoring parameters were changed from -9.5% to +8.2% compared to the initial values recorded prior to the trial. | No side-effects reported in the intervention group. |
| Chao 2007 (Comparison of the effect of a cornstarch thickened formula) | Belgium | RCT | Infants (frequent regurgitation; 90.2 ± 26.9 days in Group A, 90.5 ± 27.4 days in Group B) | 81 (41 in Group A, 40 in Group B; 52% ♂, 48% ♀) | Group A: Cornstarch-thickened AR-formula (Novalac AR®) | Dietary modification | Group B: 25% strengthened regular infant formula (Novalac 1®) | Dietary modification | After 2 months of intervention, the difference in T1/ 2 gastric emptying time, residual radioactivity at 60 and 90 min during scintigraphy between groups A and B had become significant (P < 0.001, P < 0.017, P < 0.001, respectively, Student’s t-test). | NR |
| Chao 2007 (Effect of cereal-thickened formula) | Belgium | RCT | Infants (frequent regurgitation; 130.7 ± 26.5 days in Group A, 129.1 ± 26.2 days in Group B) | 63 (31 in Group A, 32 in Group B; gender NR) | Group A: cereal-thickened regular formula | Dietary modification | Regular formula and antireflux postural therapy | Positioning therapy | "The mean incidence of regurgitation and/or vomiting per day after 4 wk (mean over a period of 3 d) decreased significantly in group A (from 3.71 ± 0.69 to 2.39 ± 0.86, P < 0.001) and group B (from 3.69 ± 0.74 to 2.84 ± 0.81, P < 0.001). The difference in frequency of episodes of regurgitation and/or vomiting per day after the 4-wk intervention was significant (P = 0.039). The cereal-thickened formula was more effective than positional intervention because the difference in frequency of regurgitation/vomiting between groups was statistically significant." | NR |
| Corvaglia 2006 | Italy | Randomized cross-over trial | Preterm infants (frequent regurgitation; age NR) | 5 (60% ♂, 40% ♀) | Milk B: Expressed human milk fortified with FM85 Nestlè (3%) and tolerated at least 100 mL/kg per day of milk, thickened with precooked starch (70% from maize, 30% from potato; 1.5 g per 100 mL of milk) | Dietary modification | Milk A: Expressed human milk fortified with FM85 Nestlè (3%) and tolerated at least 100 mL/kg per day of milk | Dietary modification | The number of acidic and buffered episodes of gastroesophageal reflux did not differ. | NR |
| Corvaglia 2013 | Italy | Randomized cross-over trial | Preterm infants (feeding intolerance and GER; median age of 43 days, range 12–82 days) | 18 (61% ♂, 39% ♀) | Hydrolyzed protein formulas (HPFs) | Dietary modification | Standard protein formulas (SPF) | Dietary modification | A significantly lower number of pH-GERs and a significantly lower RI pH were detected after eHPF compared to SPF. No difference between eHPF and SPF was shown in a MII-GERs, a MII-BEI, non-acid GER indexes or GER height. | No adverse event was recorded during the study period. |
| Cresi 2020 | Italy | RCT | Very low birth weight and very preterm infants (GER; 30 ± 1.3 weeks) | 10 (5 in the donkey milk group, 5 in the bovine milk group; 50% ♂; 50% ♀) | Donkey milk-derived human milk fortifier (DF) | Dietary modification | Bovine milk-derived human milk fortifier (BF) | Dietary modification | DF arm infants had a significant lower frequency of MII-GER with a median of 2.02 (1.95–3.26) versus 4.82 (2.84–5.94) in BF arm infants (p = 0.036). | NR |
| Del Buono 2005 | United Kingdom | RCT | Infants (GER; mean age: 163.5 days, range 34–319 days) | 20 (55% ♂, 45% ♀) | Sodium and magnesium alginate (Gaviscon, 625 mg mixed in 225 ml milk, administered 6 times daily) | Alginates | Placebo | Not applicable (placebo) | No significant difference in the number of reflux events/hour, acid reflux events/hour, or total acid clearance time/hour between Gaviscon Infant and placebo. Gaviscon Infant showed a marginally lower average reflux height in the esophagus compared to placebo (p < 0.001) | NR |
| Ewer 1999 | United Kingdom | Randomized cross-over trial | Preterm infants (excessive regurgitation of feeds and xanthine resistant apnoea and bradycardia; 11 ± 73 days) | 18 (66% ♂, 34% ♀) | Nursing in three positions (prone, left, and right lateral) for 8 hours in each position | Positioning therapy | Nursing in three positions (prone, left, and right lateral) for 8 hours in each position | Positioning therapy | For reflux index, the effect of position was highly significant (p<0.001). Prone position was significantly less than left, and both were significantly less than right position. | NR |
| Foster 2022 | Australia | Systematic review | Infants (frequent regurgitation, 1 day - 5 months) | 6 studies (736, gender NR) | Lactobacillus reuteri DSM 17938 or L. reuteri ATCC 55730 | Probiotics | Placebo | - | Meta‐analysis showed a statistically significant reduction in regurgitation in the probiotic group compared to the placebo group (MD: −1.79 episodes/day, 95% CI: −3.30 to −0.27, N=560,p=0.02). | There are no indications from the available data that probiotics have any adverse effects. |
| Hegar 2008 | Indonesia | RCT | Infants (regurgitation and/or vomiting, age NR) | 60 (gender NR) | Group B: 5 g of rice cereal added to 100 mL standard formula; Group C: formula manufactured with bean gum as a thickening agent | Dietary modification | Group A: standard infant formula | - | After the 1-month intervention, regurgitation/vomiting decreased significantly in all 3 groups (P <0.0005). Although the decrease was largest in group C ( 4.2±2.1 episodes/day), the incidence did not differ significantly with groups A or B. | NR |
| Iacono 2002 | Italy | RCT | Infants (frequent regurgitation/vomiting due to uncomplicated GER, median age 1.5 months) | 166 (82 in Group 1, 84 in Group 2; 53% ♂, 47% ♀) | Group 1: anti-regurgitation formula thickened with carob flour (locus bean gum) | Dietary modification | Group 2: common, adapted formula with a similar composition without any thickening agents | Dietary modification | There was a significant reduction in the regurgitation score in all patients, both after 4 weeks (p<0.0001 for both groups; Wilcoxon rank sum test) and after 8 weeks (p<0.0001 for both groups). In total, two-thirds of the patients were asymptomatic or improved after 8 weeks of treatment in both groups (χ2 for contingency table: 0.4; not statistically significant). However, in Group 1 (patients treated with the thickened formula) there was a higher frequency of asymptomatic infants (34%) than in the control group (14%) (χ2=7.9; p<0.01), | NR |
| Indrio 2017 | Italy | RCT | Infants (GERD; 59 ± 8.2 days in intervention group, 60 ± 5.3 in the control group) | 72 (35 in the intervention group, 37 in the control group; gender NR) | Partially hydrolysed 100% whey formula (NAN A.R.) thickened with starch, providing 1.9 g protein per 100 kcal, and supplemented with a mixture of potato, corn starch (4 g/100 kcal) and Lactobacillus reuteri DSM 17938 (2.8 106 CFU/g powder) | Dietary modification + probiotics | Starter formula that included 70% whey protein and 30% casein, providing 1.85 g of protein per 100 kcal (NAN 1) | Dietary modification | Infants receiving the test formula showed a significant reduction in the frequency of daily regurgitations compared to the control group. | No adverse events related to both the study formulas were reported. |
| Indrio 2011 | Italy | RCT | Infants (uncomplicated regurgitation; 39 ± 8.2 days in intervention group, 40 ± 5.3 days in placebo group) | 55 (34 in intervention group, 21 in control group; gender NR) | Lactobacillus reuteri (Five drops of the formulation, delivering a dose of 1 · 108 colony-forming units) | Probiotics | Placebo | - | At the end of the intervention period, the fasting antral area was significantly reduced and the delta in gastric emptying rate was significantly increased in infants receiving probiotics compared to placebo (P =0.01). Besides, the formula-fed infants receiving the probiotic had a significant decrease in the frequency of regurgitation per day compared to placebo (P <0.001) | No infants had any reported adverse events related to the trial. |
| Kenari 2020 | Iran | RCT | Infants (GERD, 1-12 months) | 90 (45 in the intervention group, 45 in the control group; 40% males, 60% females) | Omeprazole (20 mg/12 h) and abdominal massage with mastic gum oil (every 12 h) | Manual therapy | Omeprazole (20 mg/12 h) and abdominal massage without mastic gum oil (every 12 h) | Manual therapy | In both groups, the individual symptom scores for regurgitation, irritability/fussiness, arching back, choking/gagging, refusal to feed and hiccups decreased during the two weeks of treatment. The treatment was effective in both groups. The abdominal massage with mastic oil may not be more effective than massage only. | NR |
| Loots 2014 | Australia | RCT | Infants (GER, mean age 13.6 weeks) | 51 (26 treated with PPI and positioning therapy, 13 treated with antiacid and left side positioning, 12 treated with antiacid and head of cot elevation positioning; 62% males, 38% females) | Left lateral positioning (LLP) + Magnesium hydroxide and calcium carbonate (Mylanta) | Positioning therapy | Head of cot elevation (HE) + Magnesium hydroxide and calcium carbonate (Mylanta) | Positioning therapy | No treatment group showed improvement in crying/irritability, although vomiting was reduced in AA + LLP (from 7 to 2 episodes P=0.042). Gastric emptying halftime was significantly delayed compared with baseline in LLP patients (39 ± 19 minutes slower) vs HE patients (10 ± 8 minutes faster, P=0.038) regardless of medication. | No adverse events for antiacids + positioning groups. |
| Miller 1999 | United Kingdom | Phase III, double-blind, randomized, parallel-group study | Recurrent gastro-oesophageal reflux (GOR) in infants | 90 enrolled (42 alginate, 48 placebo*) Sex (%): 60.2% Male (28/42 in alginate group; 25/48 in placebo group) * two patients did not receive study medication and were excluded from analysis | Aluminium-free paediatric alginate preparation (Gaviscon Infant®) Posology: Sodium alginate 225 mg + Magnesium alginate 87.5 mg per sachet, dosed by weight and feeding method (e.g., 1 sachet in 115 ml for bottle-fed infants <4.54 kg; 2 sachets for larger infants) | Alginate-based reflux suppressant | Placebo (matched sachet) | Not applicable (placebo) | Primary Outcome: Statistically significant reduction in vomiting/regurgitation episodes in the alginate group (median reduction: from 8.5 to 3.0 episodes; placebo: from 7.0 to 5.0; p 0.009). Secondary Outcome: Severity of vomiting showed a favorable trend for alginate (p=0.061). Investigator and parent/guardian assessments rated alginate as significantly more effective than placebo (p=0.008 and p=0.002, respectively). 31% of alginate-treated infants had at least 10% symptom-free days versus 11% in placebo (p=0.027). | 57% of all patients reported adverse events: alginate group (55%), placebo group (59%) (p > 0.2). Common events: functional diarrhea (14.3% alginate, 10.9% placebo), teething syndrome (11.9% alginate, 6.5% placebo), and constipation (9.5% alginate, 2.2% placebo). Serious adverse events requiring hospitalization occurred in two patients from each group but were deemed unrelated to the treatment. |
| Miyazawa 2007 | Japan | Randomized, crossover, controlled trial | Gastroesophageal reflux (GER) in infants Sample Composition: Infants <2 months old with ≥3 regurgitation or vomiting episodes/day Mean Age ± SD: 36 ± 13 days | 20 infants (Group A: 10; Group B: 10) Sex (%): 40% Male (8/20), 60% Female (12/20) | Thickened formula with locust bean gum (HL-350, 0.35 g/100 mL) | Dietary modification | Non-thickened control formula (HL-00, 0.0 g/100 mL locust bean gum) | Dietary modification | Regurgitation reduction: HL-350 significantly reduced regurgitation episodes (median 2.3/day) compared to control formula (5.2/day) (p = 0.00048). No significant differences in feeding volume, time, body weight gain, or gastric emptying rate between HL-350 and control formula. | No adverse events reported. Slight but significant increase in bowel movements in the HL-350 group. |
| Miyazawa 2004 | Japan | Randomized, crossover trial | Uncomplicated gastroesophageal reflux (GER) with daily regurgitation Mean Age ± SD: Group A: 130.9 ± 20.8 days, Group B: 124.5 ± 17.7 days | 30 infants enrolled (14 males, 16 females) Group A: 16 infants (fed HL-450 & HL-00 in a crossover design) Group B: 14 infants (fed HL-350 & HL-00 in a crossover design, 3 excluded due to viral infections, leaving n=11 for analysis) | Anti-regurgitant (AR) milk with different concentrations of locust bean gum (LBG) Details: HL-450: Standard LBG concentration (0.45 g/100 mL) HL-350: Reduced LBG concentration (0.35 g/100 mL) Each formula was given for 1 week in a crossover design. | Dietary modification | "Non-thickened formula (HL-00, no LBG) Details: Standard infant formula without LBG (identical nutritional content to the intervention formulas except for thickener absence)." | Dietary modification | Both AR milks (HL-450 and HL-350) reduced regurgitation episodes by ~50% compared to the control formula (HL-00). Number of regurgitation episodes per day: HL-450: 1.6 (0.8–2.0) vs. HL-00: 3.5 (2.3–4.9), p = 0.0003 HL-350: 1.3 (0.6–2.3) vs. HL-00: 2.9 (2.0–3.2), p = 0.021 No significant difference in regurgitation volume between AR formulas and control milk. Mothers' preference: 81.3% (HL-450) and 81.8% (HL-350) preferred AR milk over control. | No serious adverse effects were reported. Feeding difficulties: 5 mothers in HL-450 group noted difficulty in sucking the thicker formula, while none in HL-350 group reported this issue. Bowel movements: HL-350 slightly increased bowel movement frequency compared to HL-00 (p = 0.02), but the change was clinically insignificant. |
| Miyazawa 2006 | Japan | Randomized, crossover, controlled trial | Gastroesophageal reflux (GER) in infants Sample Composition: Infants <6 months old with ≥3 regurgitation episodes/day Mean Age ± SD: 132 ± 26 days (combined groups) | 39 infants (Group A: 14; Group B: 13; Group C: 12) Sex (%): 50% Female in Group A, 46.2% in Group B, 60% in Group C | Thickened formula with locust bean gum (HL-350: 0.35 g/100 mL; HL-450: 0.45 g/100 mL) | Dietary modification | Standard non-thickened infant formula (HL-00: 0.0 g/100 mL locust bean gum) | Dietary modification | Gastric emptying: HL-450 had significantly lower gastric emptying at 120 min (52.8%) than HL-00 (97.9%, p = 0.0019). HL-350 (80.3%) did not differ significantly from HL-00. Regurgitation reduction: HL-350: 12.9 ± 3.5 episodes/week vs. HL-00: 22.6 ± 3.9 (p = 0.018). HL-450: 12.8 ± 3.0 episodes/week vs. HL-00: 29.8 ± 3.6 (p = 0.0015). | No severe adverse events reported. A few infants in the HL-350 and HL-450 groups had increased bowel movements, but none developed severe diarrhea. |
| Moukarzel 2007 | Lebanon and USA | Randomized, open-label, prospective trial | Gastroesophageal reflux (GER) in infants Mean Age ± SD: 3.24 ± 1.28 months | 74 infants enrolled After randomization: Prethickened Formula (AR) Group: 28 infants Regular Formula (R) Group: 32 infants 14 infants were excluded post-randomization due to worsening GER symptoms requiring medical therapy. Sex (%): 40 males (54%), 34 females (46%) | Prethickened formula with pregelatinized cornstarch Details: Formula thickened with pregelatinized cornstarch (designed to increase viscosity in the stomach, not in the bottle). | Dietary modification | Regular non-thickened infant formula Details: Standard infant formula with the same caloric density and nutritional composition as the intervention formula, except for thickener absence. | Dietary modification | Esophageal pH Monitoring Results (Acid Exposure Reduction): Reflux Index (RI, % time pH <4): Prethickened Formula (AR) Group: 5.64%; Regular Formula (R) Group: 7.77%; p < 0.01 (significant reduction in acid exposure with prethickened formula). Number of reflux episodes >5 min: AR Group: 1.61; R Group: 1.37 (p = 0.43, NS). Longest reflux episode duration: AR Group: 5.86 min; R Group: 11.35 min (p < 0.0001). Esophageal clearance time: AR Group: 2.10 min; R Group: 2.92 min (p < 0.0001)- Improvement in Clinical Symptoms (Parental Diary Data): Regurgitation episodes/day: Baseline: AR = 7.1, R = 6.5 (p = 0.54, NS); After 4 weeks: AR = 2.3, R = 5.2 (p = 0.0009, significant reduction in regurgitation with AR formula). Vomiting episodes/day: Baseline: AR = 2.6, R = 2.1 (p = 0.49, NS); After 4 weeks: AR = 0.5, R = 1.2 (p = 0.0003, significant reduction in vomiting with AR formula). | No serious adverse events reported. No difference in weight gain, stool frequency, or crying duration between groups. Fewer infants in the AR group had movement artifacts during electrogastrography (EGG) recordings, suggesting improved comfort during feeding. |
| Neu 2014 (Benefits of massage therapy) | USA | Randomized, controlled pilot trial | Gastroesophageal reflux disease (GERD) in infants Mean Age ± SD: 7.3 ± 1.6 weeks (massage group) vs. 7.6 ± 2.3 weeks (non-massage group) | 36 infants randomized (Massage group: 18; Non-massage group: 18) Sex (%): 67% Male (massage group), 61% Male (non-massage group) | Massage therapy administered by a professional therapist Details: 30-minute massage sessions, twice weekly for 6 weeks, targeting face, head, chest, abdomen, legs, arms, and back | Manual therapy | Non-massage sham therapy Details: 30-minute session involving light touch with minimal pressure and holding the infant upright | Manual therapy | GERD symptom reduction (I-GERQ-R score): No significant difference between groups (p = 0.58) Crying duration: Significant reduction in the massage group (p = 0.025 for <10 min/day; p = 0.0047 for <1 hr/day) Weight gain and sleep duration: No significant differences between groups | No adverse events reported. Massage was well tolerated, though therapists had to adjust techniques due to infant irritability. |
| Orenstein 1990 (Prone positioning) | USA | Randomized, controlled, crossover trial | Gastroesophageal reflux (GER) in infants Median age: 10 weeks (Range: -4 to 26 weeks) | 100 infants enrolled Infants with confirmed abnormal reflux: 90 infants (based on pH probe or histologic findings) Sex (%): not reported | Prone, head-elevated positioning (30° incline using a cloth harness) Details: Infants were placed prone with the head elevated at 30°. A cloth harness was used to secure positioning. Infants were monitored using esophageal pH probe to measure reflux parameters. | Positioning therapy | Flat prone positioning (without elevation) Details: Infants were placed flat prone (horizontal mattress, no elevation). Same monitoring protocol as the intervention group. | Positioning therapy | Postprandial Esophageal pH Monitoring Results: % Time pH <4 per 120 min: Flat prone: 32.1 ± 3.1% Head-elevated prone: 25.8 ± 3.0% (Not significant) Number of reflux episodes per 120 min: Flat prone: 7.5 ± 0.7 Head-elevated prone: 5.9 ± 0.5 (p < 0.05) Number of reflux episodes >5 min per 120 min: Flat prone: 1.4 ± 0.1 Head-elevated prone: 1.2 ± 0.1 (p < 0.005) | Not reported |
| Orenstein 1983 (Positioning for prevention) | USA | Controlled, prospective, randomized crossover trial | Gastroesophageal reflux (GER) in infants Mean Age ± SD: 2.5 months (Range: 2 weeks – 6 months) | 15 infants with documented GER (by overnight pH probe monitoring) Sex (%): not reported | "Prone, head-elevated positioning using a cloth harness Details: - Infants were positioned prone with head elevated 30-45° in a harness. - The harness was attached to the mattress, allowing for an adjustable incline” | Positioning therapy | Infant seat (semi-upright, 60° angle) Details: Infants were placed in a standard infant seat (Infanseat Babycarrier) positioned at 60° inclination. | Positioning therapy | Esophageal pH Monitoring Results: % Time pH <4 (Reflux Index): Harness: 7.9% ± 2.3%; Infant Seat: 37.4% ± 6.2% (p < 0.001). Number of GER Episodes: Harness: 5.2 ± 1.1 episodes; Infant Seat: 19.6 ± 3.5 episodes (p < 0.001). Number of GER Episodes >5 minutes: Harness: 0.6 ± 0.2 episodes; Infant Seat: 1.9 ± 0.6 episodes (p < 0.05). Longest GER Episode Duration (min): Harness: 5.0 ± 1.7 min; Infant Seat: 13.1 ± 5.0 min (p < 0.05). | Not reported |
| Orenstein 1983 (The infant seat) | USA | Prospective, randomized, controlled, crossover trial | Gastroesophageal reflux (GER) in infants Mean Age ± SD: 2.2 months (range: 0.5–4.2 months) | 9 infants Sex (%): Not specified | Positioning in an infant seat ("chalasia chair") at 60° inclination Details: Infants placed in an infant seat for two-hour postprandial periods | Positioning therapy | Prone positioning (lying flat on the stomach) Details: Infants placed in a horizontal prone position for two-hour postprandial periods | Positioning therapy | Worse GER in the infant seat compared to prone positioning: Postprandial time with esophageal pH <4: 28.2% (seat) vs. 12.8% (prone) (p = 0.023) Number of GER episodes: 16.0 (seat) vs. 10.1 (prone) (p = 0.002) Duration of longest GER episode: 6.7 min (seat) vs. 4.0 min (prone) (p = 0.079) Number of GER episodes >5 min: 1.7 (seat) vs. 0.6 (prone) (p = 0.093) Conclusion: The infant seat worsened GER compared to simple prone positioning. | No adverse events reported. Infants in the seat were more irritable and remained awake longer compared to prone positioning. |
| Ostrom 2006 | USA | Randomized, double-blind, parallel-group, controlled trial | Frequent regurgitation in healthy formula-fed infants Mean Age ± SD: 19 ± 0.5 days at enrollment | 179 infants randomized (Soy formula: 89; Cow’s milk formula: 90) Sex (%): Not reported | Soy-based formula with added soy fiber (6 g/L) (Isomil® DF, Ross Products Division) Details: Formula containing soy protein isolate, corn syrup solids and sucrose as carbohydrate sources, and soy/coconut oils as fat sources. | Dietary modification | Standard cow’s milk-based formula (Similac® with Iron, Ross Products Division) without fiber Details: Formula containing nonfat milk and whey protein concentrate as protein sources, lactose as carbohydrate source, and safflower/coconut/soy oils as fat sources. | Dietary modification | Primary Outcome: Reduction in regurgitation episodes after 7 days: Soy formula: 2.3 ± 0.2 episodes/day Cow’s milk formula: 3.4 ± 0.2 episodes/day (p = 0.001) Sustained effect at 28 days: Soy formula: 2.0 ± 0.2 episodes/day Cow’s milk formula: 2.4 ± 0.3 episodes/day (p = 0.029) Percentage of feedings with regurgitation after 7 days: Soy formula: 31.0 ± 2.4% Cow’s milk formula: 48.3 ± 4.2% (p = 0.001) At 28 days: Soy formula: 28.8 ± 3.8% Cow’s milk formula: 36.0 ± 4.2% (p = 0.015) | Adverse events: Similar rates between groups. Serious adverse events (SAEs): 5 infants experienced 6 SAEs (4 in soy formula group, 1 in cow’s milk group). Events included hospitalization for respiratory syncytial virus (RSV) pneumonia, fever, respiratory distress, and thalassemia. One infant in the soy formula group died from sudden infant death syndrome (SIDS), but it was deemed unrelated to the study feeding. No significant difference in weight gain between groups. |
| Ramirez-Mayans 2003 | Mexico | Single-blind, randomized, controlled trial | Gastroesophageal reflux (GER) in infants Mean Age ± SD: AR formula group: 2.52 ± 1.62 months RC formula group: 1.93 ± 2.23 months | 52 infants randomized (AR formula: 28; RC formula: 24) Sex (%): AR formula group: 64.3% Male (18/28) RC formula group: 54.2% Male (13/24) | Pre-thickened anti-regurgitation (AR) formula with pre-gelatinized cornstarch Details: Commercially available AR formula manufactured by Wyeth Nutrition, fed every 3 hours for 3 weeks. | Dietary modification | Standard infant formula thickened with 5% rice cereal (RC) Details: Standard whey-based infant formula with added rice cereal, fed every 3 hours for 3 weeks. | Dietary modification | Reduction in vomiting episodes: AR formula: Significant decrease over time (p = 0.04) RC formula: No significant reduction Reduction in regurgitation episodes: Both groups showed significant improvement (p = 0.03 for AR; p = 0.027 for RC). Esophageal pH improvement (24-hour pH monitoring): Reflux index (% time pH < 4): Decreased significantly in AR group, no change in RC group. Longest reflux episode: Decreased significantly in AR group, no change in RC group. Number of reflux episodes per hour: Decreased significantly in AR group, no change in RC group. | No serious adverse events. Constipation reported in 3 infants (RC group only). No reports of cough or irritability in either group. |
| Salvatore 2024 | Italy, Poland, Ukraine | Multicenter, prospective, randomized controlled trial (RCT) | Regurgitation in formula-fed infants Mean Age ± SD: 36.9 ± 12.9 days | 103 infants randomized (Test group: 52; Control group: 51) Sex (%): Test formula group: 62.7% Female Control formula group: 50.0% Female | Anti-regurgitation (AR) formula containing locust bean gum (LBG), prebiotics, and postbiotics. Details: AR formula containing locust bean gum (0.4 g/100 mL), short-chain galacto-oligosaccharides (scGOS) and long-chain fructo-oligosaccharides (lcFOS) (9:1 ratio, 0.4 g/100 mL), and postbiotics Duration: 8 weeks | Dietary modification | Unthickened control formula containing prebiotics and postbiotics but without locust bean gum (LBG) Details: Control formula contained scGOS/lcFOS (0.8 g/100 mL) and postbiotics, but no locust bean gum | Dietary modification | Primary outcome (stool consistency non-inferiority): Stool consistency in the test group was not looser or more watery than in the control group (non-inferiority confirmed). Secondary outcomes: Regurgitation frequency: Significantly lower in the test group compared to control at all time points (p ≤ 0.028). Parental satisfaction: Higher in the test group regarding regurgitation reduction (score: 8.9 ± 1.8 vs. 7.5 ± 2.8, p < 0.05). Infant well-being: No significant differences in general well-being scores between groups. | Safety Outcomes (Main Results): No severe adverse events related to the study formulas. Adverse events (AEs): Similar incidence between groups (Test: 23.5%, Control: 21.2%). Mild diarrhea: Reported in 3.9% (Test) vs. 3.8% (Control). Flatulence: Reported in 2.0% (Test) vs. 1.9% (Control). No constipation or serious gastrointestinal events. Adequate growth observed in both groups. |
| Tobin 1997 | Australia | Prospective, randomized, controlled crossover trial | Symptomatic gastroesophageal reflux (GER) in infants Mean Age ± SD: 2 months (range: <5 months) | 24 infants Sex (%): 54% Female (13/24), 46% Male (11/24) | Left lateral positioning Details: Infants were placed in the left lateral position for 6-hour periods, with and without 30° head elevation. | Positioning therapy | Supine, prone, and right lateral positioning Details: Infants rotated through four positions (supine, prone, left lateral, right lateral), both horizontally and with 30° head elevation. | Positioning therapy | Reflux Index (% time pH <4): Supine: 15.3%; Right lateral: 12.0%; Left lateral: 7.7%; Prone: 6.7%; p < 0.001 (left lateral significantly better than right lateral and supine, comparable to prone). Number of GER episodes: Supine: 7.1; Right lateral: 5.5; Left lateral: 5.8; Prone: 4.3; p = 0.007 (prone significantly better than supine). Longest reflux episode (min): Supine: 22.8; Right lateral: 21.4; Left lateral: 12.5; Prone: 10.6; p < 0.001 (prone and left lateral significantly better than supine and right lateral). Head elevation (30°) had no significant effect on GER outcomes. | No adverse events reported. The study suggests left lateral positioning is a safer alternative to prone positioning, which is associated with sudden infant death syndrome (SIDS). |
| Ummarino 2015 | Italy | Prospective, randomized, open-label, controlled trial | Gastroesophageal reflux (GER) in formula-fed infants Mean Age ± SD: Median age 5 months (range: 1–10 months) | 75 infants randomized (Group A: 25; Group B: 25; Group C: 25) Overall Population: 41 males, 34 females (55% Male) | Group B: Rice starch-thickened formula Details: Standard non-hydrolyzed milk-based formula thickened with 14.3 g rice starch/100 mL for infants <6 months and 14.2 g/100 mL for older infants. Note: The original study included a third group receiving magnesium alginate + simethicone (Group A). However, since this intervention is considered pharmacological, it was excluded from our analysis in accordance with our PICO criteria, which focus solely on non-pharmacological interventions. | Dietary modification | Group C: Reassurance + lifestyle modifications Category: Parental counseling Details: Parents received verbal and written instructions on GER management (e.g., smaller, more frequent feedings; supine sleeping position). | Parental counseling | After 4 weeks: Thickened formula (B): 16% symptom-free Reassurance (C): 0% symptom-free p < 0.0001 (B vs. C) After 8 weeks: Thickened formula (B): 65.2% symptom-free Reassurance (C): 11.76% symptom-free p < 0.002 (B vs. C) | No serious adverse events in either group. Compliance: Higher in Group B (92%) compared to Group C (68%). |
| Vandenplas 2014 | Belgium, Greece, Kuwait, Lebanon and Slovenia | RCT | Infants (suspected cow’s milk protein allergy; age 87.5 ± 46.2 days) | 72 (35 in the intervention group, 37 in the control group 50% males, 50% females) | Thickened extensive casein hydrolysate (T-eCH) | Dietary modification | Non thickened extensive casein hydrolysate (NT-eCH) | Dietary modification | Regurgitation was reduced in all infants (6.4 ± 3.2–2.8 ± 2.9, p < 0.001), but fell more with the T-eCH (4.2 ± 3.2 regurgitations/day vs. 3.0 ± 4.5, not significant), especially in infants with a negative challenge (3.9 ± 4.0 vs. 1.9 ± 3.4, not significant). | NR |
| Vandenplas 2008 | Belgium | Randomized cross-over trial | Infants (frequent crying and regurgitation; Age: NR) | 12 (6 in Formula G1 group, 6 in Formula G3 group; sex: NR) | Formula G3 (Novalac AR Digest) | Dietary modification | Formula G1 (80/20 casein/lactalbumin formula thickened with bean gum and starch) | Dietary modification | Formula G3 produced a significant decrease in regurgitation (p=0.002) and crying (p=0.003), an overall subjective well-being amelioration of the baby (p=0.005) and enhanced gastric emptying (p<0.001) compared to formula G1. | NR |
| Vandenplas 1994 | Belgium | Double-blind, randomised trial | Infants with uncomplicated Gastroesophageal reflux (GER) Age, inclusion criteria: 1 week - 4 months | 20 infants Sex (%): not reported | Parental reassurance, positional treatment, and the anti-regurgitation formula containing a bean gum preparation (thickened formula) | Dietary modification | Parental reassurance, positional treatment, and the control formula (formula without thickening product) | Dietary modification | The number of regurgitations decreased significantly from baseline in both the treatment group (P = 0.002 and P = 0.032, respectively), but no differences between groups were observed (P = 0.14). The results of a 24-h oesophageal pH monitoring, performed before and during treatment, showed a significant decrease in the percentage of time oesophageal pH was < 4.0 in the treatment group (tickened formula) (P = 0.001), but not in the control group (P = 0.09). | Not reported |
| Vandenplas 2013 | France, Belgium | Prospective, randomized,controlled, double-blind, crossover trial | Frequent regurgitation in formula-fed infants Mean Age ± SD: 9.1 ± 5.1 weeks (ARF-1) vs. 9.4 ± 4.7 weeks (ARF-2) | 115 infants (ARF-1 group: 56; ARF-2 group: 59) Sex (%): ARF-1 group: 60.7% males, 39.3% females ARF-2 group: 64.3% males, 35.7% females | Anti-regurgitation formula 1 (ARF-1) - Locust bean gum-thickened formula with non-hydrolyzed protein Details: Casein-predominant formula thickened with 3.0 g/100 g locust bean gum | Dietary modification | Anti-regurgitation formula 2 (ARF-2) - Partially hydrolyzed whey-based formula thickened with locust bean gum and starch Details: Partially hydrolyzed whey protein, thickened with 3.3 g/100 g locust bean gum + 1.9 g/100 g starch | Dietary modification | Reduction in regurgitation frequency: Baseline: 8.25 episodes/day ARF-1: 2.32 episodes/day ARF-2: 1.89 episodes/day (p = 0.0091; ARF-2 more effective) Reduction in regurgitated volume (Regurgitation Score): Baseline: 2.85 ARF-1: 1.59 ARF-2: 1.51 (p = 0.0265; ARF-2 more effective) Pediatrician assessment: ARF-1 effective/highly effective: 71.9% ARF-2 effective/highly effective: 83.5% (p < 0.0001 in favor of ARF-2) | No serious adverse events in either group. Dropout rate: 7% (ARF-1), 7.8% (ARF-2) (not significant). Adverse effects: Some infants experienced mild diarrhea, fussiness, or colic, but rates were similar in both groups. |
| Vanderhoof 2003 | United States, Canada | Multicenter, double-blind, randomized, placebo-controlled, parallel-group trial | Symptomatic gastroesophageal reflux (GER) in formula-fed infants Mean Age ± SD: 9.1 ± 5.1 weeks | 104 infants randomized (Enfamil AR: 55; Control formula: 49) Sex (%): not reported | Pre-thickened formula (Enfamil AR®) with high amylopectin rice starch Details: Formula thickened with 2.3 g/100 mL pre-gelatinized high amylopectin rice starch Designed to maintain caloric density, osmolality, and nutrient profile similar to standard formula Administered as the sole nutrition source for 5 weeks | Dietary modification | Standard cow’s milk-based formula without thickening (placebo formula) Details: Identical to Enfamil AR in composition except for the absence of rice starch | Dietary modification | Reduction in regurgitation frequency (% of feedings with regurgitation): Baseline: 87% (Enfamil AR) vs. 85% (Control) (NS) Week 1: 34% (Enfamil AR) vs. 22% (Control), p = 0.045 Week 5: 38% (Enfamil AR) vs. 24% (Control), p = 0.036 Reduction in regurgitation volume (Total Regurgitation Volume Score): Baseline: 8.9 (Enfamil AR) vs. 7.6 (Control), p = 0.042 Week 1: -4.5 (Enfamil AR) vs. -3.4 (Control), p = 0.035 Week 5: -4.6 (Enfamil AR) vs. -3.4 (Control), p = 0.050 Improvement in associated symptoms: Choking/gagging/coughing episodes: Significantly reduced with Enfamil AR at Week 1 (p = 0.004) and Week 5 (p = 0.049) Sleep disturbances: Infants with severe symptoms at baseline had greater sleep improvement in Enfamil AR group (p = 0.030) | No serious adverse events directly linked to formula intake. Study dropouts: 16% (Enfamil AR) vs. 27% (Control) (NS). Adverse events: No significant differences in constipation, diarrhea, fussiness, or gas between groups. Weight gain: Similar between groups, with Enfamil AR infants showing higher caloric intake without excess weight gain. |
| Wenzl 2003 | Germany | Randomized, placebo-controlled, crossover trial | Gastroesophageal reflux (GER) in infants Mean Age ± SD: 42 ± 32 days | 14 infants Sex (%): 64.3% Male (9 males, 5 females) | Carob bean gum-thickened formula (Formula A) Details: Formula thickened with 0.4% carob bean gum, otherwise identical to the control formula | Dietary modification | "Non-thickened standard formula (Formula B) Details: Identical composition to Formula A but without carob bean gum | Placebo | Reduction in regurgitation frequency: Formula A: 15 regurgitation episodes Formula B: 68 regurgitation episodes (p = 0.0003) Reduction in regurgitation severity score: Formula A: 0.6 Formula B: 1.8 (p = 0.003) Reduction in total GER episodes detected by intraluminal impedance: Formula A: 536 episodes Formula B: 647 episodes (p = 0.02) No significant difference in mean GER duration or number of acid reflux episodes (pH < 4) Maximum refluxate height in the esophagus was slightly lower after thickened formula, but not statistically significant (p = 0.08) | No serious adverse events reported. The thickened formula was well tolerated by all infants. |
| Xinias 2005 | Greece, Morocco, France, Belgium | Prospective, randomized, controlled trial | Regurgitation and vomiting in formula-fed infants with abnormal esophageal pH monitoring Mean Age ± SD: 93 ± 35 days | 96 infants randomized (Cornstarch-thickened formula: 51; Regular formula: 45) Sex (%): not reported | Cornstarch-thickened formula (casein-predominant, re-gelatinized cornstarch as thickener) Details: Formula thickened with cornstarch (moderate viscosity, thickens mainly in the stomach) Duration: 28 days | Dietary modification | Standard infant formula (non-thickened, casein/whey ratio 50/50) Details: Standard formula with the same caloric content as the thickened formula | Dietary modification | Reduction in regurgitation frequency: Baseline: 5.60 ± 4.15 episodes/day (thickened formula) vs. 4.77 ± 2.35 (regular formula) (p = 0.69) After 4 weeks: 2.57 ± 2.71 (thickened formula) vs. 4.31 ± 2.01 (regular formula), p < 0.0001 Reduction in vomiting frequency: Baseline: 4.34 ± 2.42 episodes/day (thickened formula) vs. 3.09 ± 1.24 (regular formula) (p = 0.04) After 4 weeks: 1.45 ± 1.65 (thickened formula) vs. 2.74 ± 1.37 (regular formula), p = 0.0011 Reduction in esophageal acid exposure time (pH-metry results): Reflux index (% time pH < 4): Baseline: 14.9 ± 10.2 (thickened) vs. 13.3 ± 6.4 (regular) (p = 0.30) After 4 weeks: 6.8 ± 6.2 (thickened) vs. 11.4 ± 7.0 (regular), p < 0.01 | No serious adverse events recorded. Weight gain (g/day): Thickened formula: 28.5 ± 12.1 g/day Regular formula: 24.3 ± 8.1 g/day (p = 0.06, not significant) No reported side effects related to the thickened formula. |
| Abbreviations: AE: Adverse events; AR: Anti-regurgitant ; ARF- 1: Anti-regurgitation formula - 1; ARF- 2: Anti-regurgitation formula - 2; BF: Bovine milk-derived human milk fortifier ; DF: Donkey milk-derived human milk fortifier ; DSM: Diagnostic and Statistical Manual of Mental Disorders; EGG: electrogastrography ; GER: Gastroesophageal reflux ; GERD: Gastroesophageal reflux disease ; I-GERQ-R: Infant Gastro-Esophageal Reflux Questionnaire Revised ; IGSQ : Infant Gastrointestinal Symptom Questionnaire; LBG: Locust bean gum; lcFOS: long-chain fructo-oligosaccharides ; NR: Not reported; RC: Rice cereal; RCT: Randomized controlled trial; RI: Resistive Index; RSV: respiratory syncytial virus ; SAEs: Serious adverse events ; scGOS: short-chain galacto-oligosaccharides ; SIDS: sudden infant death syndrome; SPF: Standard protein formulas . | | | | | | | | | | |

### PICO 7 - What are the indications and effectiveness of different surgical and endoscopic treatment options for GERD in infants, children, and adolescents?

| Study ID | Country | Study Design | Population | Sample Size | Comorbidities | Indications for surgery/inclusion criteria | Surgical procedure | Outcome | Follow-up |
| --- | --- | --- | --- | --- | --- | --- | --- | --- | --- |
| Knatten 2012 | Norway | RCT | Children  Mean age: 4.7 Y LNF; 3.5 Y ONF  Male: 57% LNF; 70% ONF  Female: 43% LNF; 30%ONF | 88 children (44 LNF; 44 ONF) | 46/88 (52%) were neurologically impaired:  - Cerebral palsy (n=18)  - Various syndromes (n=17)  - Central nervous system disorders (n=6)  - Brain damage caused by perinatal asphyxia (n=5)  42/88 (48%) were neurologically normal, but 8 of them had:  - Repaired esophageal atresia (n=4),  - Paraesophageal hernia (n=1),  - Hydrocephalus with no developmental delay (n=1),  - Heart disease (n=1),  - Kidney disease (n = 1). | Indications for fundoplication were symptoms of GER disease despite optimal medical antireflux therapy. | Laparoscopic Nissen Fundoplication vs Open Nissen Fundoplication | Patients with complications: 55% LNF, 55% ONF | 30 days |
| Fynh 2015 | Norwey | RCT | Children  Median age: 4.7 Y LNF; 3.7 Y ONF;  Male: 57% LNF; 72% ONF;  Female: 43% LNF; 28% ONF | 88 children (44 LNF; 44 ONF) | See Knatten 2012 | See Knatten 2012 | Laparoscopic Nissen Fundoplication vs Open Nissen Fundoplication | GERD in 37% after LNF vs 7% after ONF | 4 y |
| Fynh 2023 | Norwey | RCT | Children  Mean age: 4.4 Y | 88 children (44 LNF; 44 ONF) | See Knatten 2012 | See Knatten 2012 | Laparoscopic Nissen Fundoplication vs Open Nissen Fundoplication | GERD in 56% after LNF vs 31% after ONF | 4 y |
| McHoney 2011 | UK | RCT | Children  Mean age (month): 66.9 LNF; 47.6 ONF | 44 children randomized (23 LNF; 21 ONF)  39 children received allocated intervention (19LNF; 20 ONF) | 30/39 (77%) were neurologically impaired.  1/39 (3%) had a congenital anomaly. | Infants and children with GER. Exclusion criteria: patients with sepsis, multiorgan dysfunction syndrome, cardiac, renal, immunological, metabolic abnormalities, and children requiring O2 therapy. | Laparoscopic Nissen Fundoplication vs Open Nissen Fundoplication | Infection: 16% LNF vs 5% ONF;  Dysphagia: 6.3% LNF vs 0% ONF;  Recurrence: 7.1% LNF vs 16.7% ONF | 22 months |
| Pacili 2014 | UK | RCT | Children  Mean age: 4.3 Y LNF; 7.5 Y ONF | 44 children randomized ( 23 LNF; 21 ONF)  31 analyzed at long term follow-up (16 LNF; 15 ONF) | See McHoney 2011 | See McHoney 2011 | Laparoscopic Nissen Fundoplication vs Open Nissen Fundoplication | Incidence of recurrent GER: 20% LNF vs 12.5% ONF | 4.1 Y |
| Papandria 2014 | USA | RCT | Children  Age < 2 Y | 39 children (17 LNF; 22 ONF) | - Prior surgery or major procedure: 14/39 (36%)​  - Prior abdominal surgery or major procedure: 5/39 (13%) | Children under two years of age who were referred for surgical management of GERD. Patients were excluded if any concomitant abdominal surgical procedure (apart from placement or revision of a feeding gastrostomy) was to be undertaken under the same anesthetic. | Laparoscopic Nissen Fundoplication vs Open Nissen Fundoplication | Mortality: 18% LNF vs 14% ONF  Reoperation: 12% LNF vs 4% ONF  Continued symptomatic reflux: 6% LNF vs 4% ONF  Antiacid use at last follow - up: 76% LNF vs 68% ONF | 42 months |
| Kubiak 2010 | UK | RCT | Children  Mean age: 4.7 Years LNF: 5.7 LTF;  Male: 52.8% LNF; 61.6% LTF;  Female: 47.2% LNF; 38.4% LTF | 175 children (89 LNF; 86 LTF) | 121/175 (69.1%) patients had a variety of underlying neurological disorders, 13 had esophageal pathologies (e.g., congenital diaphragmatic hernia or esophageal atresia).  41/175 (23.4%) patients were classified as normal children without other significant medical conditions contributing toward the gastroesophageal reflux (GOR). | Inclusion criteria for entry into the study included proven GOR unresponsive to medical treatment, patients who had failed medical treatment or who had serious complications (i.e., apnea, aspiration pneumonia, esophagitis, or failure to thrive), or those who had a hiatus hernia.  Patients were excluded if they had had previous antireflux surgery or previous open abdominal surgery. | Laparoscopic Nissen fundoplication vs Laparoscopic Thal fundoplication | Dysphagia: 13.5% LNF vs 11.6% LTF  Severe Dysphagia: 10.1% LNF vs 1.7% LTF  Death: 1.1% LNF vs 1.7% LTF | 6 weeks |
| Kubiak 2011 | UK | RCT | Children  Mean age: 4.6 Years LNF: 5.5 LTF;  Male: 52.9% LNF; 59.8% LTF;  Female: 47.1% LNF; 40.2% LTF | 167 children (85 LNF; 82 LTF) | See Kubiak 2010 | See Kubiak 2010​ | Laparoscopic Nissen fundoplication vs Laparoscopic Thal fundoplication | Dysphagia: 23.5% Nissen vs 2.4% Thal  Severe Dysphagia: 11.8% Nissen vs 2.4% Thal  Death: 24.7% Nissen vs 12.2% Thal | 30 months |
| Skerrit 2022 | UK | RCT | Children  Mean age: 4.6 Years LNF: 5.5 LTF;  Male: 53% LNF; 61% LTF;  Female: 47% LNF; 39% LTF | 175 children (89 LNF; 86 LTF) | See Kubiak 2010 | See Kubiak 2010 | Laparoscopic Nissen Fundoplication vs Laparoscopic Thal Fundoplication | Absolute failure rate: 9.4% LNF vs 18% LTF  Relative failure: 8.2% LNF vs 14% LTF  Symptoms of GORD: 12.1% LNF vs 25.5% LTF  Dysphagia: 12% LNF, 2.4% LTF;  Death: 43% LNF, 27% LTF | 5 Y |
| Gad 2022 | Egypt | RCT | Children  Median (month): 25 M; 18.5;  Male: 50% LNF; 70% Hill-Snow;  Female: 50% LNF; 30% Hill-Snow | 40 children (20 Nissen; 20 Hill-Snow) | 5/40 (12.5%) were neurologically impaired.  About 95% of cases suffered from hiatal hernias. Almost two-thirds of the patients (70%) had type I hiatus hernia, and 25% had type III.  Presentations of GERD:  - Persistent vomiting only: n=13 (32.5%)  - Hematemesis only: n=9 (22.5%)  - Vomiting and hematemesis: n=8 (20%)  - Hematemesis and melena: n=3 (7.5%)  - Dysphagia (esophageal stricture): n=3 (7.5%)  - Anemia, melena: n=1 (2.5%)  - Chocking with chest symptoms and feeding problems: n=1 (2.5%)  - Fever and bleeding and vomiting: n=1 (2.5%)  - Pulmonary symptoms: n=1 (2.5%) | Patients were included if they exhibited a poor response to medical treatment, had a symptomatic hiatus hernia, and/or presented with esophageal stricture or life-threatening symptoms.​  Exclusion criteria: Cases with grade IV hiatus hernia, para-esophageal hernia, or recurrent GERD. | Laparoscopic Nissen fundoplication vs Hill-Snow technique | Late postoperative assessment  Dysphagia: 25% LNF, 15% Hill-Snow;  Bloating: 55% LNF, 0% Hill-Snow;  Dumping: 25% LNF, 5% Hill-Snow  Vomiting: 30% LNF, 40% Hill-Snow  Ability to blench: 60% LNF, 80% Hill-Snow  Recurrence: 10% LNF, 15% Hill-Snow  Need for PPIs: 25% LNF, 40% Hill-Snow | 6 months |
| Abbreviations: GER: Gastroesophageal reflux; GERD: Gastroesophageal reflux disease; LNF: Laparoscopic nissen fundoplication; LTF: Laparoscopic thal fundoplication; ONF: Open nissen fundoplication; RCT: Randomized controlled trial. | | | | | | | | | |

### PICO 8 – What is the prognosis of GER and GERD in infants, children, and adolescents, and what are the prognostic factors?

| Study ID | Country | Study design | Population (age, sex) | Sample size | Prognostic factors |
| --- | --- | --- | --- | --- | --- |
| Curien-Chotard 2020 | France | Prospective cohort study | M 54.6 % F 45.4%  Range 1-12 months | 141 | Two risk factors were identified for GER and GERD at 1 month of age: family history of GER (OR: 2.9–95% CI [1.2–7.1] and OR: 4.8–95% CI [1.6–14.4]- respectively) and exposure to paternal smoking (Table 2). |
| Ghiga 2020 | Romania | retro-prospective case-control study | M 65.2% F 34.8%  Mean Age: 65.05 months | 267 | Biochemical modifications in gastroesophageal reflux disease are correlated with disease progression (Serum Iron, Aminotransferases, Protein and Electrophoresis, Immunoglobulins, Blood glucose and Magnesium) |
| Ruigomez 2010 | Spain | Cohort Study | M 50,0% F 50,0%  Range age: 1-17 years | 1242 | Compared with children with no diagnosis of GERD, those with a diagnosis of GERD had an increased risk of a subsequent diagnosis of an extra-esophageal condition, particularly asthma, pneumonia, laryngitis, cough, or chest pain) |
| Singendonk 2018 | Netherlands | Systematic Review | Age range: 0-17 years | 1594 | Results of unfavorable outcome with regards to resolution/occurrence of clinical GERD symptoms and endoscopic complications are summarized in Table 1. |
| Abbreviations: GER: Gastroesophageal reflux ; GERD: Gastroesophageal reflux disease. | | | | | |
