# Supplementary material for: Guidelines on diagnosis and management of gastroesophageal reflux disease in infants, children and adolescents: a joint consensus from Italian pediatric societies (SIP and SIGENP) -Part II: management
Source: Ital J Pediatr. 2026 Apr 10;52:90. doi: 10.1186/s13052-026-02255-0 (PMC13182049; doi:10.1186/s13052-026-02255-0)
Supplement: Supplementary file 3 — Additional File 3 [file 13052_2026_2255_MOESM3_ESM.docx]

# Additional File 3

## Quality assessment tables

### PICO 5 - What is the evidence of effectiveness of pharmacologic treatment for GER/GERD in pediatric patients?

#### RCTs

| **Proton Pump Inhibitors (PPI) vs Placebo** | | | | | | |
| --- | --- | --- | --- | --- | --- | --- |
| Study | **Adverse events** | | | | | |
|  | Domain 1 | Domain 2 | Domain 3 | Domain 4 | Domain 5 | Overall |
| Omeprazole vs Placebo | | | | | | |
| Moore 2003 | S | L | L | L | S | S |
| Omari 2007 | L | L | L | L | S | S |
| Størdal 2005 | L | L | L | L | S | S |
| Esomeprazole vs Placebo | | | | | | |
| Davidson 2003 | L | L | L | L | L | L |
| Winter 2012 | S | L | L | L | S | S |
| Dexlansoprazole vs Placebo | | | | | | |
| Gremse 2019 | S | L | L | L | L | S |
| Lansoprazole vs Placebo | | | | | | |
| Orenstein 2009 | S | L | L | L | L | S |
| Pantoprazole vs Placebo | | | | | | |
| Winter 2010 | S | L | L | L | S | S |
| Rabeprazole vs Placebo | | | | | | |
| Hussain 2014 | S | L | L | L | L | S |
| Study | **Vomiting/regurgitation (frequency)** | | | | | |
|  | Domain 1 | Domain 2 | Domain 3 | Domain 4 | Domain 5 | Overall |
| Omeprazole vs Placebo | | | | | | |
| Omari 2007 | L | L | L | L | S | S |
| Esomeprazole vs Placebo | | | | | | |
| Davidson 2003 | L | L | L | L | L | L |
| Lansoprazole vs Placebo | | | | | | |
| Orenstein 2009 | S | L | L | L | L | S |
| Pantoprazole vs Placebo | | | | | | |
| Winter 2010 | S | L | L | L | S | S |
| Rabeprazole vs Placebo | | | | | | |
| Hussain 2014 | S | L | L | L | L | S |
| Study | **Infant Gastro-Esophageal Reflux Questionnaire Revised (I-GERQ-R)** | | | | | |
|  | Domain 1 | Domain 2 | Domain 3 | Domain 4 | Domain 5 | Overall |
| Esomeprazole vs Placebo | | | | | | |
| Winter 2012 | S | L | L | L | S | S |
| Rabeprazole vs Placebo | | | | | | |
| Hussain 2014 | S | L | L | L | L | S |
| Study | **Heartburn (frequency)** | | | | | |
|  | Domain 1 | Domain 2 | Domain 3 | Domain 4 | Domain 5 | Overall |
| Omeprazole vs Placebo | | | | | | |
| Fallahi 2008 | S | L | L | L | S | S |
| Dexlansoprazole vs Placebo | | | | | | |
| Gremse 2019 | S | L | L | L | L | S |
| Study | **Epigastric pain (frequency)** | | | | | |
|  | Domain 1 | Domain 2 | Domain 3 | Domain 4 | Domain 5 | Overall |
| Omeprazole vs Placebo | | | | | | |
| Fallahi 2008 | S | L | L | L | S | S |
| Lansoprazole vs Placebo | | | | | | |
| Orenstein 2009 | S | L | L | L | L | S |
| Study | **Respiratory symptoms, nocturnal cough, asthma (frequency)** | | | | | |
|  | Domain 1 | Domain 2 | Domain 3 | Domain 4 | Domain 5 | Overall |
| Esomeprazole vs Placebo | | | | | | |
| Winter 2012 | S | L | L | L | S | S |
| Lansoprazole vs Placebo | | | | | | |
| Orenstein 2009 | S | L | L | L | L | S |
| Legend:  L = low risk  S = some concerns  H = high risk Domain 1: Bias arising from the randomization process Domain 2: Bias due to deviations from intended interventions Domain 3: Bias due to missing outcome data Domain 4: Bias in measurement of the outcome Domain 5: Bias in selection of the reported result | | | | | | |

| **Proton Pump Inhibitors (PPI) vs H2-Antagonists** | | | | | | |
| --- | --- | --- | --- | --- | --- | --- |
| Study | **Adverse events** | | | | | |
|  | Domain 1 | Domain 2 | Domain 3 | Domain 4 | Domain 5 | Overall |
| Omeprazole vs Ranitidine | | | | | | |
| Cucchiara 1993 | S | H | H | H | H | H |
| Ummarino 2012 | S | L | L | H | S | H |
| Study | **Vomiting/regurgitation (frequency)** | | | | | |
|  | Domain 1 | Domain 2 | Domain 3 | Domain 4 | Domain 5 | Overall |
| Omeprazole vs Ranitidine | | | | | | |
| Azizollahi 2016 | S | L | L | L | L | S |
| Boccia 2007 | L | L | L | L | L | L |
| Ummarino 2012 | S | L | L | H | S | H |
| Study | **Heartburn (frequency)** | | | | | |
|  | Domain 1 | Domain 2 | Domain 3 | Domain 4 | Domain 5 | Overall |
| Omeprazole vs Ranitidine | | | | | | |
| Boccia 2007 | L | L | L | L | L | L |
| Study | **Epigastric pain (frequency)** | | | | | |
|  | Domain 1 | Domain 2 | Domain 3 | Domain 4 | Domain 5 | Overall |
| Omeprazole vs Ranitidine | | | | | | |
| Boccia 2007 | L | L | L | L | L | L |
| Study | **Respiratory symptoms, nocturnal cough, asthma (frequency)** | | | | | |
|  | Domain 1 | Domain 2 | Domain 3 | Domain 4 | Domain 5 | Overall |
| Omeprazole vs Ranitidine | | | | | | |
| Boccia 2007 | L | L | L | L | L | L |
| Ummarino 2012 | S | L | L | H | S | H |
| Study | **Severity of esophagitis** | | | | | |
|  | Domain 1 | Domain 2 | Domain 3 | Domain 4 | Domain 5 | Overall |
| Omeprazole vs Ranitidine | | | | | | |
| Cucchiara 1993 | S | H | H | L | S | H |
| Legend:  L = low risk  S = some concerns  H = high risk Domain 1: Bias arising from the randomization process Domain 2: Bias due to deviations from intended interventions Domain 3: Bias due to missing outcome data Domain 4: Bias in measurement of the outcome Domain 5: Bias in selection of the reported result | | | | | | |

| **Proton Pump Inhibitors (PPI) vs No Treatment** | | | | | | |
| --- | --- | --- | --- | --- | --- | --- |
| Study | **Respiratory symptoms, nocturnal cough, asthma (frequency)** | | | | | |
|  | Domain 1 | Domain 2 | Domain 3 | Domain 4 | Domain 5 | Overall |
| Omeprazole vs No treatment | | | | | | |
| Yagoubi 2022 | S | S | L | H | S | H |
| Legend:  L = low risk  S = some concerns  H = high risk Domain 1: Bias arising from the randomization process Domain 2: Bias due to deviations from intended interventions Domain 3: Bias due to missing outcome data Domain 4: Bias in measurement of the outcome Domain 5: Bias in selection of the reported result | | | | | | |

| **H2-Antagonists vs Placebo** | | | | | | |
| --- | --- | --- | --- | --- | --- | --- |
| Study | **Adverse events** | | | | | |
|  | Domain 1 | Domain 2 | Domain 3 | Domain 4 | Domain 5 | Overall |
| Cimetidine vs Placebo | | | | | | |
| Cucchiara 1989 | S | L | L | H | S | H |
| Ranitidine vs Placebo | | | | | | |
| Gustafsson 1992 | S | L | L | L | S | S |
| Famotidine vs Placebo | | | | | | |
| Orenstein 2003 | L | L | L | L | L | L |
| Study | **Vomiting/regurgitation (frequency)** | | | | | |
|  | Domain 1 | Domain 2 | Domain 3 | Domain 4 | Domain 5 | Overall |
| Cimetidine vs Placebo | | | | | | |
| Cucchiara 1989 | S | L | L | H | L | H |
| Famotidine vs Placebo | | | | | | |
| Orenstein 2003 | L | L | L | H | L | H |
| Nizatidine vs Placebo | | | | | | |
| Simeone 1997 | S | L | L | L | S | S |
| Study | **Heartburn (frequency)** | | | | | |
|  | Domain 1 | Domain 2 | Domain 3 | Domain 4 | Domain 5 | Overall |
| Cimetidine vs Placebo | | | | | | |
| Cucchiara 1989 | S | L | L | H | L | H |
| Nizatidine vs Placebo | | | | | | |
| Simeone 1997 | S | L | L | L | S | S |
| Study | **Respiratory symptoms, nocturnal cough, asthma (frequency)** | | | | | |
|  | Domain 1 | Domain 2 | Domain 3 | Domain 4 | Domain 5 | Overall |
| Cimetidine vs Placebo | | | | | | |
| Cucchiara 1989 | S | L | L | H | L | H |
| Ranitidine vs Placebo | | | | | | |
| Gustafsson 1992 | L | L | L | S | S | L |
| Study | **Severity of esophagitis** | | | | | |
|  | Domain 1 | Domain 2 | Domain 3 | Domain 4 | Domain 5 | Overall |
| Cimetidine vs Placebo | | | | | | |
| Cucchiara 1989 | S | L | L | H | L | H |
| Nizatidine vs Placebo | | | | | | |
| Simeone 1997 | S | L | L | L | S | S |
| Legend:  L = low risk  S = some concerns  H = high risk Domain 1: Bias arising from the randomization process Domain 2: Bias due to deviations from intended interventions Domain 3: Bias due to missing outcome data Domain 4: Bias in measurement of the outcome Domain 5: Bias in selection of the reported result | | | | | | |

| **H2-Antagonists vs Sucralfate** | | | | | | |
| --- | --- | --- | --- | --- | --- | --- |
| Study | **Adverse events** | | | | | |
|  | Domain 1 | Domain 2 | Domain 3 | Domain 4 | Domain 5 | Overall |
| Cimetidine vs Sucralfate | | | | | | |
| Argüelles-Martin 1989 | S | L | L | H | S | H |
| Study | **Epigastric pain (frequency)** | | | | | |
|  | Domain 1 | Domain 2 | Domain 3 | Domain 4 | Domain 5 | Overall |
| Cimetidine vs Sucralfate | | | | | | |
| Argüelles-Martin 1989 | S | L | L | H | S | H |
| Legend:  L = low risk  S = some concerns  H = high risk Domain 1: Bias arising from the randomization process Domain 2: Bias due to deviations from intended interventions Domain 3: Bias due to missing outcome data Domain 4: Bias in measurement of the outcome Domain 5: Bias in selection of the reported result | | | | | | |

| **Prokinetic agents vs Placebo** | | | | | | |
| --- | --- | --- | --- | --- | --- | --- |
| Study | **Adverse events** | | | | | |
|  | Domain 1 | Domain 2 | Domain 3 | Domain 4 | Domain 5 | Overall |
| Cisapride vs Placebo | | | | | | |
| Cohen 1999 | S | L | H | L | S | H |
| Cucchiara 1987 | S | H | H | L | S | H |
| Saye 1987 | S | L | L | L | S | S |
| Scott 1997 | L | L | L | L | S | S |
| Van Eygen 1989 | S | L | L | L | S | S |
| Domperidone vs Placebo | | | | | | |
| Bines 1992 | S | L | L | L | L | S |
| Carroccio 1994 | L | L | L | L | S | S |
| Clara 1979 | S | L | L | L | S | S |
| De Loore 1979 | S | L | L | L | S | S |
| Metoclopramide vs Placebo | | | | | | |
| Bellissant 1997 | S | H | H | L | S | H |
| Forbes 1986 | S | L | L | L | S | S |
| Machida 1988 | S | L | H | L | S | H |
| Tolia 1989 | L | L | L | L | S | S |
| De Loore 1979 | S | L | L | L | S | S |
| Study | **Vomiting/regurgitation (frequency)** | | | | | |
|  | Domain 1 | Domain 2 | Domain 3 | Domain 4 | Domain 5 | Overall |
| Cisapride vs Placebo | | | | | | |
| Cohen 1999 | S | L | H | L | S | H |
| Cucchiara 1987 | S | H | H | L | S | H |
| Van Eygen 1989 | S | L | L | L | S | S |
| Domperidone vs Placebo | | | | | | |
| Clara 1979 | S | L | L | L | L | S |
| De Loore 1979 | S | L | L | L | S | S |
| Metoclopramide vs Placebo | | | | | | |
| De Loore 1979 | S | L | L | L | S | S |
| Study | **Infant Gastro-Esophageal Reflux Questionnaire Revised (I-GERQ-R)** | | | | | |
|  | Domain 1 | Domain 2 | Domain 3 | Domain 4 | Domain 5 | Overall |
| Cisapride vs Placebo | | | | | | |
| Barnett 2001 | S | L | L | L | L | S |
| Study | **Respiratory symptoms, nocturnal cough, asthma (frequency)** | | | | | |
|  | Domain 1 | Domain 2 | Domain 3 | Domain 4 | Domain 5 | Overall |
| Cisapride vs Placebo | | | | | | |
| Cohen 1999 | S | L | H | L | S | H |
| Study | **Severity of esophagitis** | | | | | |
|  | Domain 1 | Domain 2 | Domain 3 | Domain 4 | Domain 5 | Overall |
| Cisapride vs Placebo | | | | | | |
| Cucchiara 1987 | S | H | H | L | S | H |
| Legend:  L = low risk  S = some concerns  H = high risk Domain 1: Bias arising from the randomization process Domain 2: Bias due to deviations from intended interventions Domain 3: Bias due to missing outcome data Domain 4: Bias in measurement of the outcome Domain 5: Bias in selection of the reported result | | | | | | |

| **Bethanechol vs Placebo** | | | | | | |
| --- | --- | --- | --- | --- | --- | --- |
| Study | **Adverse events** | | | | | |
|  | Domain 1 | Domain 2 | Domain 3 | Domain 4 | Domain 5 | Overall |
| Euler 1980 | L | L | L | L | S | S |
| Legend:  L = low risk  S = some concerns  H = high risk Domain 1: Bias arising from the randomization process Domain 2: Bias due to deviations from intended interventions Domain 3: Bias due to missing outcome data Domain 4: Bias in measurement of the outcome Domain 5: Bias in selection of the reported result | | | | | | |

#### Systematic reviews

| Studies | 1 | 2 | 3 | 4 | 5 | 6 | 7 | 8 | 9 | 10 | 11 | Total N. of “yes” |
| --- | --- | --- | --- | --- | --- | --- | --- | --- | --- | --- | --- | --- |
| Alla 2024 | Yes | Yes | Yes | Yes | Yes | Yes | No | Yes | No | Not Applicable | No | 7/11 |
| Arabpour 2023 | Yes | Yes | Yes | Yes | Yes | No | No | Yes | Yes | Not Applicable | No | 7/11 |
| Dalby-Payne 2003 | Yes | Yes | Yes | Yes | Yes | Yes | No | Yes | Yes | Not Applicable | No | 8/11 |
| Hibbs 2006 | Yes | Yes | No | No | Yes | No | No | Yes | Yes | Not Applicable | No | 5/11 |
| Maclennan 2010 | Yes | Yes | Yes | Yes | Yes | Yes | Yes | Yes | Yes | Yes | Yes | 11/11 |
| Tighe 2023 | Yes | Yes | Yes | Yes | Yes | Yes | Yes | Yes | Yes | Yes | Yes | 11/11 |

1. Is the review question clearly and explicitly stated?
2. Were the inclusion criteria appropriate for the review question?
3. Was the search strategy appropriate?
4. Were the sources and resources used to search for studies adequate?
5. Were the criteria for appraising studies appropriate?
6. Was critical appraisal conducted by two or more reviewers independently?
7. Were there methods to minimize errors in data extraction?
8. Were the methods used to combine studies appropriate?
9. Was the likelihood of publication bias assessed?
10. Were recommendations for policy and/or practice supported by the reported data?
11. Were the specific directives for new research appropriate?

### PICO 6 - What is the effectiveness of different non-pharmacologic treatment options for GER/GERD?

#### RCTs

| **Dietary modifications** | | | | | | | | | | | | |
| --- | --- | --- | --- | --- | --- | --- | --- | --- | --- | --- | --- | --- |
| Study ID | **Adverse events** | | | | | | | | | | | |
|  | Domain 1 | | Domain 2 | | Domain 3 | | | Domain 4 | | Domain 5 | | Overall |
| Novel anti-regurgitation formula with fermented formula VS Regular anti-regurgitation formula | | | | | | | | | | | | |
| Bellaiche 2021 | S | | L | | L | | | L | | L | | S |
| Cornstarch-thickened AR-formula VS Regular formula | | | | | | | | | | | | |
| Moukarzel 2007 | S | | S | | L | | | H | | L | | H |
| Ramirez-Mayans 2003 | S | | L | | L | | | L | | L | | S |
| Xinias 2005 | L | | L | | L | | | L | | L | | L |
| Hydrolyzed protein formulas (HPFs) VS Standard protein formula (SPF) | | | | | | | | | | | | |
| Corvaglia 2013 | S | | L | | L | | | L | | S | | S |
| Thickened formula with locust bean gum VS Regular formula | | | | | | | | | | | | |
| Miyazawa 2007 | S | | S | | L | | | H | | L | | H |
| Miyazawa 2004 | S | | L | | L | | | H | | L | | H |
| Miyazawa 2006 | S | | S | | L | | | H | | L | | H |
| Soy-based formula VS Bovine milk-based formula | | | | | | | | | | | | |
| Ostrom 2006 | S | | L | | H | | | L | | L | | H |
| Anti-regurgitation (AR) formula containing locust bean gum (LBG), prebiotics, and postbiotics VS Regular formula | | | | | | | | | | | | |
| Salvatore 2024 | L | | L | | L | | | L | | L | | L |
| Mg alginate plus simethicone vs Formula thickened with rice cereals vs Parental counselling | | | | | | | | | | | | |
| Ummarino 2015 | S | | S | | L | | | L | | L | | S |
| Thickened formula with locust bean gum (ARF1) VS Thickened formula with locust bean gum (ARF2) | | | | | | | | | | | | |
| Vandenplas 2013 | L | | L | | H | | | H | | L | | H |
| Formula thickened with rice starch (Enfamil AR®) VS Bovine milk-based formula | | | | | | | | | | | | |
| Vanderhoof 2003 | L | | L | | H | | | L | | L | | H |
| Carob bean thickened formula VS Regular formula | | | | | | | | | | | | |
| Wenzl 2003 | L | | L | | S | | | L | | L | | S |
| Study ID | **Vomiting/regurgitation (frequency)** | | | | | | | | | | | |
|  | Domain 1 | | Domain 2 | | Domain 3 | | | Domain 4 | | Domain 5 | | Overall |
| Cornstarch-thickened AR-formula VS Regular formula | | | | | | | | | | | | |
| Chao 2007 (Comparison) | L | | L | | L | | | H | | L | | H |
| Moukarzel 2007 | S | | S | | L | | | H | | L | | H |
| Ramirez-Mayans 2003 | S | | L | | L | | | L | | L | | S |
| Xinias 2005 | L | | L | | L | | | L | | L | | L |
| Cereal-thickened AR-formula VS Regular formula and positional therapy | | | | | | | | | | | | |
| Chao 2007 (Effect) | S | | L | | L | | | H | | L | | H |
| Formula thickened with rice cereals VS Formula thickened with bean gum VS Regular formula | | | | | | | | | | | | |
| Hegar 2008 | S | | L | | H | | | L | | S | | H |
| Thickened formula with locust bean gum VS Regular formula | | | | | | | | | | | | |
| Miyazawa 2007 | S | | S | | L | | | H | | L | | H |
| Miyazawa 2004 | S | | L | | L | | | H | | L | | H |
| Miyazawa 2006 | S | | S | | L | | | H | | L | | H |
| Soy-based formula VS Bovine milk-based formula | | | | | | | | | | | | |
| Ostrom 2006 | S | | L | | L | | | L | | L | | S |
| Anti-regurgitation (AR) formula containing locust bean gum (LBG), prebiotics, and postbiotics VS Regular formula | | | | | | | | | | | | |
| Salvatore 2024 | L | | L | | L | | | L | | L | | L |
| Mg alginate plus simethicone vs Formula thickened with rice cereals vs Parental counselling | | | | | | | | | | | | |
| Ummarino 2015 | S | | S | | L | | | L | | L | | S |
| Thickened formula with locust bean gum (ARF1) VS Thickened formula with locust bean gum (ARF2) | | | | | | | | | | | | |
| Vandenplas 2013 | L | | L | | L | | | L | | L | | L |
| Formula thickened with rice starch (Enfamil AR®) VS Bovine milk-based formula | | | | | | | | | | | | |
| Vanderhoof 2003 | L | | L | | L | | | L | | L | | L |
| Carob bean thickened formula VS Regular formula | | | | | | | | | | | | |
| Wenzl 2003 | L | | L | | L | | | L | | L | | L |
| Study ID | **Infant Gastro-Esophageal Reflux Questionnaire Revised (I-GERQ-R)** | | | | | | | | | | | |
|  | Domain 1 | | Domain 2 | | Domain 3 | | | Domain 4 | | Domain 5 | | Overall |
| Novel anti-regurgitation formula with fermented formula VS Regular anti-regurgitation formula | | | | | | | | | | | | |
| Bellaiche 2021 | S | | L | | L | | | L | | L | | S |
| Anti-regurgitation (AR) formula containing locust bean gum (LBG), prebiotics, and postbiotics VS Regular formula | | | | | | | | | | | | |
| Salvatore 2024 | L | | L | | L | | | L | | L | | L |
| Mg alginate plus simethicone vs Formula thickened with rice cereals vs Parental counselling | | | | | | | | | | | | |
| Ummarino 2015 | S | | S | | L | | | L | | L | | S |
| Study ID | **Total number of reflux events** | | | | | | | | | | | |
|  | Domain 1 | Domain 2 | | Domain 3 | | | Domain 4 | | Domain 5 | | Overall | |
| Cornstarch-thickened AR-formula VS Regular formula | | | | | | | | | | | | |
| Xinias 2005 | L | | L | | L | | | L | | L | | L |
| Fortified milk thickened with starch VS Fortified milk | | | | | | | | | | | | |
| Corvaglia 2006 | S | | L | | S | | | L | | S | | S |
| Hydrolyzed protein formulas (HPFs) VS Standard protein formula (SPF) | | | | | | | | | | | | |
| Corvaglia 2013 | S | | L | | L | | | L | | S | | S |
| Carob bean thickened formula VS Regular formula | | | | | | | | | | | | |
| Wenzl 2003 | L | | L | | L | | | L | | L | | L |
| Anti-regurgitation formula (Novalac AR Digest) VS Thickened formula with bean gum and starch | | | | | | | | | | | | |
| Vandenplas 2008 | H | | L | | L | | | H | | L | | H |
| Thickened extensive casein hydrolysate (T-eCH) VS Non thickened extensive casein hydrolysate (NT-eCH) | | | | | | | | | | | | |
| Vandenplas 2014 | L | | L | | L | | | L | | L | | L |
| Study ID | **Estimated volume regurgitated** | | | | | | | | | | | |
|  | Domain 1 | | Domain 2 | | | Domain 3 | | Domain 4 | | Domain 5 | | Overall |
| Thickened formula with locust bean gum VS Regular formula | | | | | | | | | | | | |
| Miyazawa 2004 | S | | L | | L | | | H | | L | | H |
| Miyazawa 2006 | S | | S | | L | | | H | | L | | H |
| Anti-regurgitation (AR) formula containing locust bean gum (LBG), prebiotics, and postbiotics VS Regular formula | | | | | | | | | | | | |
| Salvatore 2024 | L | | L | | S | | | L | | L | | S |
| Thickened formula with locust bean gum (ARF1) VS Thickened formula with locust bean gum (ARF2) | | | | | | | | | | | | |
| Vandenplas 2013 | L | | L | | L | | | L | | L | | L |
| Formula thickened with rice starch (Enfamil AR®) VS Bovine milk-based formula | | | | | | | | | | | | |
| Vanderhoof 2003 | L | | L | | L | | | L | | L | | L |
| Study ID | **Respiratory symptoms, nocturnal cough, asthma (frequency)** | | | | | | | | | | | |
|  | Domain 1 | | Domain 2 | | | Domain 3 | | Domain 4 | | Domain 5 | | Overall |
| Cornstarch-thickened AR-formula VS Regular formula | | | | | | | | | | | | |
| Chao 2007 (Comparison) | L | | L | | L | | | H | | L | | H |
| Mg alginate plus simethicone vs Formula thickened with rice cereals vs Parental counselling | | | | | | | | | | | | |
| Ummarino 2015 | S | | S | | L | | | L | | L | | S |
| Study ID | **Weight Gain** | | | | | | | | | | | |
|  | Domain 1 | | Domain 2 | | Domain 3 | | | Domain 4 | | Domain 5 | | Overall |
| Cornstarch-thickened AR-formula VS Regular formula | | | | | | | | | | | | |
| Chao 2007 (Comparison) | L | | L | | L | | | H | | L | | H |
| Xinias 2005 | L | | L | | L | | | L | | L | | L |
| Cereal-thickened AR-formula VS Regular formula and positional therapy | | | | | | | | | | | | |
| Chao 2007 (Effect) | S | | L | | L | | | H | | L | | H |
| Formula thickened with rice cereals VS Formula thickened with bean gum VS Regular formula | | | | | | | | | | | | |
| Hegar 2008 | S | | L | | H | | | L | | S | | H |
| Thickened formula with locust bean gum VS Regular formula | | | | | | | | | | | | |
| Miyazawa 2007 | S | | S | | L | | | H | | L | | H |
| Miyazawa 2004 | S | | L | | L | | | H | | L | | H |
| Soy-based formula VS Bovine milk-based formula | | | | | | | | | | | | |
| Ostrom 2006 | S | | L | | L | | | L | | L | | S |
| Thickened formula with locust bean gum (ARF1) VS Thickened formula with locust bean gum (ARF2) | | | | | | | | | | | | |
| Vandenplas 2013 | L | | L | | L | | | L | | L | | L |
| Thickened extensive casein hydrolysate (T-eCH) VS Non thickened extensive casein hydrolysate (NT-eCH) | | | | | | | | | | | | |
| Vandenplas 2014 | L | | L | | L | | | L | | L | | L |
| Legend:  L = low risk  S = some concerns  H = high risk Domain 1: Bias arising from the randomization process Domain 2: Bias due to deviations from intended interventions Domain 3: Bias due to missing outcome data Domain 4: Bias in measurement of the outcome Domain 5: Bias in selection of the reported result | | | | | | | | | | | | |

| **Probiotics** | | | | | | |
| --- | --- | --- | --- | --- | --- | --- |
| Study ID | **Adverse events** | | | | | |
|  | Domain 1 | Domain 2 | Domain 3 | Domain 4 | Domain 5 | Overall |
| *Lactobacillus reuteri* VS placebo | | | | | | |
| Indrio 2011 | S | L | L | L | L | S |
| Study ID | **Vomiting/regurgitation (frequency)** | | | | | |
|  | Domain 1 | Domain 2 | Domain 3 | Domain 4 | Domain 5 | Overall |
| *Lactobacillus reuteri* VS placebo | | | | | | |
| Indrio 2011 | S | L | L | L | L | S |
| Study ID | **Infant Gastro-Esophageal Reflux Questionnaire Revised (I-GERQ-R)** | | | | | |
|  | Domain 1 | Domain 2 | Domain 3 | Domain 4 | Domain 5 | Overall |
| BB-12 (ABINAT12®) VS No treatment | | | | | | |
| Baldassarre 2022 | S | L | L | H | L | H |
| Legend:  L = low risk  S = some concerns  H = high risk Domain 1: Bias arising from the randomization process Domain 2: Bias due to deviations from intended interventions Domain 3: Bias due to missing outcome data Domain 4: Bias in measurement of the outcome Domain 5: Bias in selection of the reported result | | | | | | |

| **Positioning therapy** | | | | | | |
| --- | --- | --- | --- | --- | --- | --- |
| Study ID | **Adverse events** | | | | | |
|  | Domain 1 | Domain 2 | Domain 3 | Domain 4 | Domain 5 | Overall |
| Infant seat (semi-upright, 60° angle) VS Flat prone positioning | | | | | | |
| Orenstein 1983 (The infant seat) | S | H | L | H | S | H |
| Study ID | **Total number of reflux events** | | | | | |
|  | Domain 1 | Domain 2 | Domain 3 | Domain 4 | Domain 5 | Overall |
| Prone feeding position VS lateral left VS lateral right | | | | | | |
| Ewer 1999 | S | S | L | H | S | H |
| Prone, 30-45° head-elevated positioning VS Infant seat (semi-upright, 60° angle) | | | | | | |
| Orenstein 1983 (Positioning for prevention) | S | H | L | H | S | H |
| Infant seat (semi-upright, 60° angle) VS Flat prone positioning | | | | | | |
| Orenstein 1983 (The infant seat) | S | H | L | H | S | H |
| Prone feeding position VS lateral left VS lateral right VS Supine position (all with 30° elevated head) | | | | | | |
| Tobin 1997 | S | S | L | H | L | H |
| Legend:  L = low risk  S = some concerns  H = high risk Domain 1: Bias arising from the randomization process Domain 2: Bias due to deviations from intended interventions Domain 3: Bias due to missing outcome data Domain 4: Bias in measurement of the outcome Domain 5: Bias in selection of the reported result | | | | | | |

| **Dietary modifications + Probiotics** | | | | | | |
| --- | --- | --- | --- | --- | --- | --- |
| Study ID | **Adverse events** | | | | | |
|  | Domain 1 | Domain 2 | Domain 3 | Domain 4 | Domain 5 | Overall |
| Partially hydrolysed 100% whey formula (NAN A.R.) thickened with starch + Lactobacillus reuteri VS Regular starter formula (NAN 1) | | | | | | |
| Indrio 2017 | S | L | L | L | L | S |
| Study ID | **Vomiting/regurgitation (frequency)** | | | | | |
|  | Domain 1 | Domain 2 | Domain 3 | Domain 4 | Domain 5 | Overall |
| Indrio 2017 | S | L | L | L | L | S |
| Study ID | **Weight Gain** | | | | | |
|  | Domain 1 | Domain 2 | Domain 3 | Domain 4 | Domain 5 | Overall |
| Indrio 2017 | S | L | L | L | L | S |
| Legend:  L = low risk  S = some concerns  H = high risk Domain 1: Bias arising from the randomization process Domain 2: Bias due to deviations from intended interventions Domain 3: Bias due to missing outcome data Domain 4: Bias in measurement of the outcome Domain 5: Bias in selection of the reported result | | | | | | |

| **Positioning therapy + antiacids** | | | | | | |
| --- | --- | --- | --- | --- | --- | --- |
| Study ID | **Adverse events** | | | | | |
|  | Domain 1 | Domain 2 | Domain 3 | Domain 4 | Domain 5 | Overall |
| Left lateral position + antiacids VS Elevated head + antiacids | | | | | | |
| Loots 2014 | L | L | L | L | L | L |
| Study ID | **Vomiting/regurgitation (frequency)** | | | | | |
|  | Domain 1 | Domain 2 | Domain 3 | Domain 4 | Domain 5 | Overall |
| Loots 2014 | L | L | L | L | L | L |
| Study ID | **Total number of reflux events** | | | | | |
|  | Domain 1 | Domain 2 | Domain 3 | Domain 4 | Domain 5 | Overall |
| Loots 2014 | L | L | L | L | L | L |
| Study ID | **Respiratory symptoms, nocturnal cough, asthma (frequency)** | | | | | |
|  | Domain 1 | Domain 2 | Domain 3 | Domain 4 | Domain 5 | Overall |
| Loots 2014 | L | L | L | L | L | L |
| Legend:  L = low risk  S = some concerns  H = high risk Domain 1: Bias arising from the randomization process Domain 2: Bias due to deviations from intended interventions Domain 3: Bias due to missing outcome data Domain 4: Bias in measurement of the outcome Domain 5: Bias in selection of the reported result | | | | | | |

| **Alginates vs Placebo** | | | | | | |
| --- | --- | --- | --- | --- | --- | --- |
| Study ID | **Adverse events** | | | | | |
|  | Domain 1 | Domain 2 | Domain 3 | Domain 4 | Domain 5 | Overall |
| Alginate (Gaviscon) vs Placebo | | | | | | |
| Buts 1987 | S | H | L | H | S | H |
| Miller 1999 | S | L | L | L | S | S |
| Study ID | **Vomiting/regurgitation (frequency)** | | | | | |
|  | Domain 1 | Domain 2 | Domain 3 | Domain 4 | Domain 5 | Overall |
| Alginate (Gaviscon) vs Placebo | | | | | | |
| Miller 1999 | S | L | L | L | S | S |
| Legend:  L = low risk  S = some concerns  H = high risk Domain 1: Bias arising from the randomization process Domain 2: Bias due to deviations from intended interventions Domain 3: Bias due to missing outcome data Domain 4: Bias in measurement of the outcome Domain 5: Bias in selection of the reported result | | | | | | |

| **Alginates vs Thickened feed** | | | | | | |
| --- | --- | --- | --- | --- | --- | --- |
| Study ID | **Infant Gastro-Esophageal Reflux Questionnaire Revised (I-GERQ-R)** | | | | | |
|  | Domain 1 | Domain 2 | Domain 3 | Domain 4 | Domain 5 | Overall |
| Magnesium-Alginate vs Thickened Formula | | | | | | |
| Baldassarre 2019 | S | L | L | H | L | H |
| Legend:  L = low risk  S = some concerns  H = high risk Domain 1: Bias arising from the randomization process Domain 2: Bias due to deviations from intended interventions Domain 3: Bias due to missing outcome data Domain 4: Bias in measurement of the outcome Domain 5: Bias in selection of the reported result | | | | | | |

| **Massage therapy** | | | | | | | | | | | |
| --- | --- | --- | --- | --- | --- | --- | --- | --- | --- | --- | --- |
| Study ID | **Vomiting/regurgitation (frequency)** | | | | | | | | | | |
|  | Domain 1 | Domain 2 | | Domain 3 | | Domain 4 | | Domain 5 | | Overall | |
| Abdominal massage with mastic gum oil VS abdominal massage without mastic gum oil | | | | | | | | | | | |
| Kenari 2020 | L | L | | L | | S | | H | | H | |
| Study ID | **Infant Gastro-Esophageal Reflux Questionnaire Revised (I-GERQ-R)** | | | | | | | | | | |
|  | Domain 1 | | Domain 2 | | Domain 3 | | Domain 4 | | Domain 5 | | Overall |
| Massage therapy VS Sham non-massage therapy | | | | | | | | | | | |
| Neu 2014 | L | | L | | L | | L | | L | | L |
| Study ID | **Weight Gain** | | | | | | | | | | |
|  | Domain 1 | | Domain 2 | | Domain 3 | | Domain 4 | | Domain 5 | | Overall |
| Massage therapy VS Sham non-massage therapy | | | | | | | | | | | |
| Neu 2014 | L | | L | | L | | H | | L | | H |
| Legend:  L = low risk  S = some concerns  H = high risk Domain 1: Bias arising from the randomization process Domain 2: Bias due to deviations from intended interventions Domain 3: Bias due to missing outcome data Domain 4: Bias in measurement of the outcome Domain 5: Bias in selection of the reported result | | | | | | | | | | | |

#### Systematic reviews

| Study ID | 1 | 2 | 3 | 4 | 5 | 6 | 7 | 8 | 9 | 10 | 11 | Total N. of “yes” |
| --- | --- | --- | --- | --- | --- | --- | --- | --- | --- | --- | --- | --- |
| Foster 2022 | yes | yes | yes | yes | yes | no | no | yes | no | NA | no | 6/11 |

NA, Not Applicable

1. Is the review question clearly and explicitly stated?
2. Were the inclusion criteria appropriate for the review question?
3. Was the search strategy appropriate?
4. Were the sources and resources used to search for studies adequate?
5. Were the criteria for appraising studies appropriate?
6. Was critical appraisal conducted by two or more reviewers independently?
7. Were there methods to minimize errors in data extraction?
8. Were the methods used to combine studies appropriate?
9. Was the likelihood of publication bias assessed?
10. Were recommendations for policy and/or practice supported by the reported data?
11. Were the specific directives for new research appropriate?

### PICO 7 - What is the indication and the effectiveness of different surgical/endoscopic treatment options for GERD?

#### Risk of bias

| **Laparoscopic Nissen fundoplication vs Open Nissen Fundoplication** | | | | | | |
| --- | --- | --- | --- | --- | --- | --- |
| Study | **GERD recurrence** | | | | | |
|  | Domain 1 | Domain 2 | Domain 3 | Domain 4 | Domain 5 | Overall |
| Fynh 2015 | L | L | L | L | L | L |
| Fynh 2023 | L | L | L | L | L | L |
| McHoney 2011 | L | S | L | L | L | S |
| Pacili 2014 | L | S | L | L | L | S |
| Papandria 2014 | L | L | L | L | L | L |
| Study | **Mortality** | | | | | |
|  | Domain 1 | Domain 2 | Domain 3 | Domain 4 | Domain 5 | Overall |
| Knatten 2012 | L | L | L | L | L | L |
| Fynh 2015 | L | L | L | L | L | L |
| Fynh 2023 | L | L | L | L | L | L |
| Papandria 2014 | L | L | L | L | L | L |
| Study | **Dysphagia** | | | | | |
|  | Domain 1 | Domain 2 | Domain 3 | Domain 4 | Domain 5 | Overall |
| Fynh 2015 | L | L | L | L | L | L |
| Fynh 2023 | L | L | L | L | L | L |
| McHoney 2011 | L | S | L | L | L | S |
| Study | **Anti-secretory drug use** | | | | | |
|  | Domain 1 | Domain 2 | Domain 3 | Domain 4 | Domain 5 | Overall |
| Fynh 2023 | L | L | L | L | L | L |
| Papandria 2014 | L | L | L | L | L | L |
| Study | **Retching** | | | | | |
|  | Domain 1 | Domain 2 | Domain 3 | Domain 4 | Domain 5 | Overall |
| Fynh 2015 | L | L | L | L | L | L |
| Fynh 2023 | L | L | L | L | L | L |
| McHoney 2011 | L | S | L | L | L | S |
| Pacili 2014 | L | S | L | L | L | S |
| Study | **Dumping syndrome** | | | | | |
|  | Domain 1 | Domain 2 | Domain 3 | Domain 4 | Domain 5 | Overall |
| Pacili 2014 | L | S | L | L | L | S |
| Study | **Gas bloat syndrome** | | | | | |
|  | Domain 1 | Domain 2 | Domain 3 | Domain 4 | Domain 5 | Overall |
| Pacili 2014 | L | S | L | L | L | S |
| Legend:  L = low risk  S = some concerns  H = high risk Domain 1: Bias arising from the randomization process Domain 2: Bias due to deviations from intended interventions Domain 3: Bias due to missing outcome data Domain 4: Bias in measurement of the outcome Domain 5: Bias in selection of the reported result | | | | | | |

| **Laparoscopic Nissen fundoplication vs Thal Nissen Funduplication** | | | | | | |
| --- | --- | --- | --- | --- | --- | --- |
| Study | **Mortality** | | | | | |
|  | Domain 1 | Domain 2 | Domain 3 | Domain 4 | Domain 5 | Overall |
| Kubiak 2010 | S | L | L | L | L | S |
| Kubiak 2011 | S | L | L | L | L | S |
| Skerrit 2022 | S | L | L | L | L | S |
| Study | **Dysphagia** | | | | | |
|  | Domain 1 | Domain 2 | Domain 3 | Domain 4 | Domain 5 | Overall |
| Kubiak 2010 | S | L | L | L | L | S |
| Kubiak 2011 | S | L | L | L | H | H |
| Skerrit 2022 | S | L | L | L | H | H |
| Study | **Anti-secretory drug use** | | | | | |
|  | Domain 1 | Domain 2 | Domain 3 | Domain 4 | Domain 5 | Overall |
| Kubiak 2011 | S | L | L | L | H | H |
| Skerrit 2022 | S | L | L | L | H | H |
| Legend:  L = low risk  S = some concerns  H = high risk Domain 1: Bias arising from the randomization process Domain 2: Bias due to deviations from intended interventions Domain 3: Bias due to missing outcome data Domain 4: Bias in measurement of the outcome Domain 5: Bias in selection of the reported result | | | | | | |

| **Laparoscopic Nissen fundoplication vs Hill – Snow Tecnique** | | | | | | |
| --- | --- | --- | --- | --- | --- | --- |
| Study | **GERD recurrence** | | | | | |
|  | Domain 1 | Domain 2 | Domain 3 | Domain 4 | Domain 5 | Overall |
| Gad 2022 | L | L | L | L | L | L |
| Study | **Dysphagia** | | | | | |
|  | Domain 1 | Domain 2 | Domain 3 | Domain 4 | Domain 5 | Overall |
| Gad 2022 | L | L | L | L | L | L |
| Study | **Anti-secretory drug use** | | | | | |
|  | Domain 1 | Domain 2 | Domain 3 | Domain 4 | Domain 5 | Overall |
| Gad 2022 | L | L | L | L | L | L |
| Study | **Dumping syndrome** | | | | | |
|  | Domain 1 | Domain 2 | Domain 3 | Domain 4 | Domain 5 | Overall |
| Gad 2022 | L | L | L | L | L | L |
| Study | **Gas bloat syndrome** | | | | | |
|  | Domain 1 | Domain 2 | Domain 3 | Domain 4 | Domain 5 | Overall |
| Gad 2022 | L | L | L | L | L | L |
| Legend:  L = low risk  S = some concerns  H = high risk Domain 1: Bias arising from the randomization process Domain 2: Bias due to deviations from intended interventions Domain 3: Bias due to missing outcome data Domain 4: Bias in measurement of the outcome Domain 5: Bias in selection of the reported result | | | | | | |
