# Supplementary material for: Guidelines on diagnosis and management of gastroesophageal reflux disease in infants, children and adolescents: a joint consensus from Italian pediatric societies (SIP and SIGENP) -Part II: management
Source: Ital J Pediatr. 2026 Apr 10;52:90. doi: 10.1186/s13052-026-02255-0 (PMC13182049; doi:10.1186/s13052-026-02255-0)
Supplement: Supplementary file 4 — Additional File 4 [file 13052_2026_2255_MOESM4_ESM.docx]

# Additional File 4

## GRADE

### PICO 5 - What is the evidence of effectiveness of pharmacologic treatment for GER/GERD in pediatric patients?

#### Proton Pump Inhibitors (PPI) vs Placebo

##### **Question:** Omeprazole vs Placebo for GERD

| **Certainty assessment** | | | | | | | | | | | | **№ of patients** | | | | **Effect** | | | | **Certainty** | | **Importance** | |
| --- | --- | --- | --- | --- | --- | --- | --- | --- | --- | --- | --- | --- | --- | --- | --- | --- | --- | --- | --- | --- | --- | --- | --- |
| **Studies** | **Study design** | | **Risk of bias** | **Inconsistency** | | **Indirectness** | | **Imprecision** | | **Other considerations** | | **Omeprazole** | | **Placebo** | | **Relative (95% CI)** | | **Absolute (95% CI)** | |  |  |  |  |
| **Vomiting/regurgitation (frequency)** | | | | | | | | | | | | | | | | | | | | | | | |
| Omari 2007 | RCT | | serious ^a^ | not serious | | not serious | | serious ^b^ | | none | | n=10  Median 6.5 (IQR 3 to 14.3) | | n=10  Median 8.5 (IQR 7 to 22.8) | | not significant | | not available | | ⨁⨁◯◯ Low | | CRITICAL | |
| **Infant Gastro-Esophageal Reflux Questionnaire Revised (I-GERQ-R)** | | | | | | | | | | | | | | | | | | | | | | | |
| Not reported | | | | | | | | | | | | | | | | | | | | | | | |
| **Heartburn (frequency)** | | | | | | | | | | | | | | | | | | | | | | | |
| Fallahi 2008 | RCT | | serious ^c^ | not serious | | not serious | | serious ^b^ | | none | | n=18  7 patients before treatment, 1 patient after treatment | | n=18 | | p = 0.040 | | not available | | ⨁⨁◯◯ Low | | CRITICAL | |
| **Epigastric pain (frequency)** | | | | | | | | | | | | | | | | | | | | | | | |
| Fallahi 2008 | RCT | | serious ^c^ | not serious | | not serious | | serious ^b^ | | none | | n=18  4 patients before treatment, 1 patient after treatment | | n=18 | | not reported | | not available | | ⨁⨁◯◯ Low | | CRITICAL | |
| **Respiratory symptoms, nocturnal cough, asthma (frequency)** | | | | | | | | | | | | | | | | | | | | | | | |
| Not reported | | | | | | | | | | | | | | | | | | | | | | | |
| **Severity of esophagitis** | | | | | | | | | | | | | | | | | | | | | | | |
| Not reported | | | | | | | | | | | | | | | | | | | | | | | |
| **Adverse events** | | | | | | | | | | | | | | | | | | | | | | | |
| Moore 2003 | RCT | serious ^c^ | | | not serious | | not serious | | serious ^b^ | | none | | n=15  No adverse events reported | | n=15  No adverse events reported | | not reported | | not available | | ⨁⨁◯◯ Low | | CRITICAL |
| Omari 2007 | RCT | not serious | | | serious ^a^ | | not serious | | serious ^b^ | | none | | n=10  No adverse events reported | | n=10  No adverse events reported | | not reported | | not available | | ⨁⨁◯◯ Low | | CRITICAL |
| Størdal 2005 | RCT | not serious | | | serious ^a^ | | not serious | | serious ^b^ | | none | | n=19  1 patient with adverse events | | n=19  1 patient with adverse events | | not significant | | not available | | ⨁⨁◯◯ Low | | CRITICAL |

**CI:** confidence interval; **RR:** risk ratio; **RCT**: randomized controlled trial

Explanations

a. Some concerns in domain 5 of RoB 2

b. Small sample size

c. Some concerns in domains 1 and 5 of RoB 2

##### **Question:** Esomeprazole vs Placebo for GERD

| **Certainty assessment** | | | | | | | **№ of patients** | | **Effect** | | **Certainty** | **Importance** |
| --- | --- | --- | --- | --- | --- | --- | --- | --- | --- | --- | --- | --- |
| **Studies** | **Study design** | **Risk of bias** | **Inconsistency** | **Indirectness** | **Imprecision** | **Other considerations** | **Esomeprazole** | **Placebo** | **Relative (95% CI)** | **Absolute (95% CI)** |  |  |
| **Vomiting/regurgitation (frequency)** | | | | | | | | | | | | |
| Davidson 2003 | RCT | not serious | not serious | not serious | serious ^a^ | none | n=25  Change from baseline, Mean (SD) -0.58 (4.68) | n=26  Change from baseline, Mean (SD) 0.70 (6.46) | P = 0.42 | not available | ⨁⨁⨁◯ Moderate | CRITICAL |
| **Infant Gastro-Esophageal Reflux Questionnaire Revised (I-GERQ-R)** | | | | | | | | | | | | |
| Winter 2012 | RCT | serious ^b^ | not serious | not serious | serious ^a^ | none | n=37  Change from baseline (vomiting), Mean (SD) -0.04 (0.56) | n=40  Change from baseline (vomiting), Mean (SD) 0.09 (0.61) | not reported | not available | ⨁⨁◯◯ Low | CRITICAL |
| **Heartburn (frequency)** | | | | | | | | | | | | |
| Not reported | | | | | | | | | | | | |
| **Epigastric pain (frequency)** | | | | | | | | | | | | |
| Not reported | | | | | | | | | | | | |
| **Respiratory symptoms, nocturnal cough, asthma (frequency)** | | | | | | | | | | | | |
| Winter 2012 | RCT | serious ^b^ | not serious | not serious | serious ^a^ | none | n=37  Change from baseline (vomiting), Mean (SD) -0.12 (0.48) | n=40  Change from baseline (vomiting), Mean (SD) 0.03 (0.58) | not reported | not available | ⨁⨁◯◯ Low | CRITICAL |
| **Severity of esophagitis** | | | | | | | | | | | | |
| Not reported | | | | | | | | | | | | |
| **Adverse events** | | | | | | | | | | | | |
| Davidson 2003 | RCT | not serious | not serious | not serious | serious ^a^ | none | n=25  6 patients with adverse events | n=26  9 patients with adverse events | not reported | not available | ⨁⨁⨁◯ Moderate | CRITICAL |
| Winter 2012 | RCT | serious ^b^ | not serious | not serious | serious ^a^ | none | 23 of 39 (59%) | 27 of 41 (66%) | not reported | not available | ⨁⨁◯◯ Low | CRITICAL |

**CI:** confidence interval; **RR:** risk ratio; **RCT**: randomized controlled trial

Explanations

a. Small sample size

b. Some concerns in domains 1 and 5 of RoB 2

##### **Question:** Dexlansoprazole vs Placebo for GERD

| **Certainty assessment** | | | | | | | **№ of patients** | | **Effect** | | **Certainty** | **Importance** |
| --- | --- | --- | --- | --- | --- | --- | --- | --- | --- | --- | --- | --- |
| **Studies** | **Study design** | **Risk of bias** | **Inconsistency** | **Indirectness** | **Imprecision** | **Other considerations** | **Dexlansoprazole** | **Placebo** | **Relative (95% CI)** | **Absolute (95% CI)** |  |  |
| **Vomiting/regurgitation (frequency)** | | | | | | | | | | | | |
| Not reported | | | | | | | | | | | | |
| **Infant Gastro-Esophageal Reflux Questionnaire Revised (I-GERQ-R)** | | | | | | | | | | | | |
| Not reported | | | | | | | | | | | | |
| **Heartburn (frequency)** | | | | | | | | | | | | |
| Gremse 2019 | RCT | serious ^a^ | not serious | not serious | serious ^b^ | none | n=25  The median percentage of days without daytime heartburn was 89.7% | n=26  The median percentage of days without daytime heartburn was 82.7% | not reported | not available | ⨁⨁◯◯ Low | CRITICAL |
| **Epigastric pain (frequency)** | | | | | | | | | | | | |
| Not reported | | | | | | | | | | | | |
| **Respiratory symptoms, nocturnal cough, asthma (frequency)** | | | | | | | | | | | | |
| Not reported | | | | | | | | | | | | |
| **Severity of esophagitis** | | | | | | | | | | | | |
| Not reported | | | | | | | | | | | | |
| **Adverse events** | | | | | | | | | | | | |
| Gremse 2019 | RCT | serious ^a^ | not serious | not serious | serious ^b^ | none | n=25  18 patients with adverse events | n=26  16 patients with adverse events | not reported | not available | ⨁⨁◯◯ Low | CRITICAL |

**CI:** confidence interval; **RR:** risk ratio; **RCT**: randomized controlled trial

Explanations

a. Some concerns in domains 1 and 5 of RoB 2

b. Small sample size

##### **Question:** Lansoprazole vs Placebo for GERD

| **Certainty assessment** | | | | | | | **№ of patients** | | **Effect** | | **Certainty** | **Importance** |
| --- | --- | --- | --- | --- | --- | --- | --- | --- | --- | --- | --- | --- |
| **Studies** | **Study design** | **Risk of bias** | **Inconsistency** | **Indirectness** | **Imprecision** | **Other considerations** | **Lansoprazole** | **Placebo** | **Relative (95% CI)** | **Absolute (95% CI)** |  |  |
| **Vomiting/regurgitation (frequency)** | | | | | | | | | | | | |
| Orenstein 2009 | RCT | serious ^a^ | not serious | not serious | not serious | none | n = 81  Regurgitate,  -14 % of feeds/week | n = 81  Regurgitate,  -11 % of feeds/week | not significant | not available | ⨁⨁⨁◯ Moderate | CRITICAL |
| **Infant Gastro-Esophageal Reflux Questionnaire Revised (I-GERQ-R)** | | | | | | | | | | | | |
| Not reported | | | | | | | | | | | | |
| **Heartburn (frequency)** | | | | | | | | | | | | |
| Not reported | | | | | | | | | | | | |
| **Epigastric pain (frequency)** | | | | | | | | | | | | |
| Not reported | | | | | | | | | | | | |
| **Respiratory symptoms, nocturnal cough, asthma (frequency)** | | | | | | | | | | | | |
| Orenstein 2009 | RCT | serious ^a^ | not serious | not serious | not serious | none | n = 81  Coughing, 0% of days/week  Wheezing,  -5% of days/week | n = 81  Coughing, -9% of days/week  Wheezing,  -6% of days/week | not significant | not available | ⨁⨁⨁◯ Moderate | CRITICAL |
| **Severity of esophagitis** | | | | | | | | | | | | |
| Not reported | | | | | | | | | | | | |
| **Adverse events** | | | | | | | | | | | | |
| Orenstein 2009 | RCT | serious ^a^ | not serious | not serious | not serious | none | n = 81  Treatment-emergent AEs occurred in 62% of patients | n = 81  Treatment-emergent AEs occurred in 46% of patients | P = 0.058 | not available | ⨁⨁⨁◯ Moderate | CRITICAL |

**CI:** confidence interval; **RR:** risk ratio; **RCT**: randomized controlled trial

Explanations

a. Some concerns in domain 1 of RoB 2

##### **Question:** Pantoprazole vs Placebo for GERD

| **Certainty assessment** | | | | | | | **№ of patients** | | **Effect** | | **Certainty** | **Importance** |
| --- | --- | --- | --- | --- | --- | --- | --- | --- | --- | --- | --- | --- |
| **Studies** | **Study design** | **Risk of bias** | **Inconsistency** | **Indirectness** | **Imprecision** | **Other considerations** | **Pantoprazole** | **Placebo** | **Relative (95% CI)** | **Absolute (95% CI)** |  |  |
| **Vomiting/regurgitation (frequency)** | | | | | | | | | | | | |
| Winter 2010 | RCT | serious ^a^ | not serious | not serious | not serious | none | n = 52  Mean (SD) change from  baseline, week 4: -0.45 (0.68)  Mean (SD) change from  baseline, week 8: -0.62 (0.72) | n = 54  Mean (SD) change from  baseline, week 4: -0.41 (0.52)  Mean (SD) change from  baseline, week 8: -0.48 (0.87) | p <0.001 | not available | ⨁⨁⨁◯ Moderate | CRITICAL |
| **Infant Gastro-Esophageal Reflux Questionnaire Revised (I-GERQ-R)** | | | | | | | | | | | | |
| Not reported | | | | | | | | | | | | |
| **Heartburn (frequency)** | | | | | | | | | | | | |
| Not reported | | | | | | | | | | | | |
| **Epigastric pain (frequency)** | | | | | | | | | | | | |
| Not reported | | | | | | | | | | | | |
| **Respiratory symptoms, nocturnal cough, asthma (frequency)** | | | | | | | | | | | | |
| Not reported | | | | | | | | | | | | |
| **Severity of esophagitis** | | | | | | | | | | | | |
| Not reported | | | | | | | | | | | | |
| **Adverse events** | | | | | | | | | | | | |
| Winter 2010 | RCT | serious ^a^ | not serious | not serious | not serious | none | n = 52  Upper respiratory infection: 7 (13.0%);  Rash/contact dermatitis: 4 (7.4%) | n = 54  Upper respiratory infection: 7 (13.0%);  Rash/contact dermatitis: 2 (3.7%) | not reported | not available | ⨁⨁⨁◯ Moderate | CRITICAL |

**CI:** confidence interval; **RR:** risk ratio; **RCT**: randomized controlled trial

Explanations

a. Some concerns in domains 1 and 5 of RoB 2

##### **Question:** Rabeprazole vs Placebo for GERD

| **Certainty assessment** | | | | | | | **№ of patients** | | **Effect** | | **Certainty** | **Importance** |
| --- | --- | --- | --- | --- | --- | --- | --- | --- | --- | --- | --- | --- |
| **Studies** | **Study design** | **Risk of bias** | **Inconsistency** | **Indirectness** | **Imprecision** | **Other considerations** | **Rabeprazole** | **Placebo** | **Relative (95% CI)** | **Absolute (95% CI)** |  |  |
| **Vomiting/regurgitation (frequency)** | | | | | | | | | | | | |
| Hussain 2014 | RCT | serious ^a^ | not serious | not serious | not serious | none | Rabeprazole 5 mg: n = 90; rabeprazole 10 mg: n = 88  No differences in the frequency of regurgitation from baseline to the end of follow-up between the three groups | n = 90 | not significant | not available | ⨁⨁⨁◯ Moderate | CRITICAL |
| **Infant Gastro-Esophageal Reflux Questionnaire Revised (I-GERQ-R)** | | | | | | | | | | | | |
| Hussain 2014 | RCT | serious ^a^ | not serious | not serious | not serious | none | Rabeprazole 5 mg: n = 90; rabeprazole 10 mg: n = 88  No differences in the I-GERQ scores from baseline to the end of follow-up between the three groups | n = 90 | not significant | not available | ⨁⨁⨁◯ Moderate | CRITICAL |
| **Heartburn (frequency)** | | | | | | | | | | | | |
| Not reported | | | | | | | | | | | | |
| **Epigastric pain (frequency)** | | | | | | | | | | | | |
| Not reported | | | | | | | | | | | | |
| **Respiratory symptoms, nocturnal cough, asthma (frequency)** | | | | | | | | | | | | |
| Not reported | | | | | | | | | | | | |
| **Severity of esophagitis** | | | | | | | | | | | | |
| Not reported | | | | | | | | | | | | |
| **Adverse events** | | | | | | | | | | | | |
| Hussain 2014 | RCT | serious ^a^ | not serious | not serious | not serious | none | Rabeprazole 5 mg: n = 90; rabeprazole 10 mg: n = 88  Treatment-emergent AEs occurred in 43% (Rabeprazole 5 mg) and 50% (Rabeprazole 10 mg) of patients | n = 90  Treatment-emergent AEs occurred in 47% of patients | not significant | not available | ⨁⨁⨁◯ Moderate | CRITICAL |

**CI:** confidence interval; **RR:** risk ratio; **RCT**: randomized controlled trial

Explanations

a. Some concerns in domain 1 of RoB 2

#### Proton Pump Inhibitors (PPI) vs H2-Antagonists

##### **Question:** Omeprazole vs Ranitidine for GERD

| **Certainty assessment** | | | | | | | **№ of patients** | | **Effect** | | **Certainty** | **Importance** |
| --- | --- | --- | --- | --- | --- | --- | --- | --- | --- | --- | --- | --- |
| **Studies** | **Study design** | **Risk of bias** | **Inconsistency** | **Indirectness** | **Imprecision** | **Other considerations** | **Omeprazole** | **Ranitidine** | **Relative (95% CI)** | **Absolute (95% CI)** |  |  |
| **Vomiting/regurgitation (frequency)** | | | | | | | | | | | | |
| Azizollahi 2016 | RCT | serious ^a^ | not serious | not serious | serious ^b^ | none | n = 30  Change from baseline score, week 1: 21.74–32.21  Change from baseline score, week 2: 5.01–11.25 | n = 30  Change from baseline score, week 1: 17.25–24.53  Change from baseline score, week 2: 7.5–13.6 | p = 0.019 | not available | ⨁⨁◯◯ Low | CRITICAL |
| Boccia 2007 | RCT | not serious | not serious | not serious | serious ^b^ | none | n = 16 | n = 16 | No significant differences between groups | not available | ⨁⨁⨁◯ Moderate | CRITICAL |
| Ummarino 2012 | RCT | very serious ^c^ | not serious | not serious | serious ^b^ | none | n = 19  Before therapy: 2.26 (1.24); After therapy: 0.21 (3.9) | n = 16  Before therapy: 2.75 (3.86); After therapy: 1.75 (3.61) | p = 0.0003 | not available | ⨁◯◯◯ Very low | CRITICAL |
| **Infant Gastro-Esophageal Reflux Questionnaire Revised (I-GERQ-R)** | | | | | | | | | | | | |
| Not reported | | | | | | | | | | | | |
| **Heartburn (frequency)** | | | | | | | | | | | | |
| Boccia 2007 | RCT | not serious | not serious | not serious | serious ^b^ | none | n = 16 | n = 16 | No significant differences between groups | not available | ⨁⨁⨁◯ Moderate | CRITICAL |
| **Epigastric pain (frequency)** | | | | | | | | | | | | |
| Boccia 2007 | RCT | not serious | not serious | not serious | serious ^b^ | none | n = 16 | n = 16 | No significant differences between groups | not available | ⨁⨁⨁◯ Moderate | CRITICAL |
| **Respiratory symptoms, nocturnal cough, asthma (frequency)** | | | | | | | | | | | | |
| Boccia 2007 | RCT | not serious | not serious | not serious | serious ^b^ | none | n = 16 | n = 16 | No significant differences between groups | not available | ⨁⨁⨁◯ Moderate | CRITICAL |
| Ummarino 2012 | RCT | very serious ^c^ | not serious | not serious | serious ^b^ | none | n = 19  Before therapy: 5.84 (2.91); After therapy: 0.79 (2.42) | n = 16  Before therapy: 5.5 (3.76); After therapy: 2.5 (3.92) | p < 0.001 | not available | ⨁◯◯◯ Very low | CRITICAL |
| **Severity of esophagitis** | | | | | | | | | | | | |
| Cucchiara 1993 | RCT | very serious ^c^ | not serious | not serious | serious ^b^ | none | n = 12  Histological degree of esophagitis: before mean 8.0, after mean 2.0 | n = 13  Histological degree of esophagitis: before mean 8.0, after mean 2.0 | No significant differences between groups | not available | ⨁◯◯◯ Very low | CRITICAL |
| **Adverse events** | | | | | | | | | | | | |
| Cucchiara 1993 | RCT | very serious ^c^ | not serious | not serious | serious ^b^ | none | n = 12  No adverse events observed | n = 13  No adverse events observed | not available | not available | ⨁◯◯◯ Very low | CRITICAL |
| Ummarino 2012 | RCT | very serious ^c^ | not serious | not serious | serious ^b^ | none | n = 19  No adverse events observed | n = 16  No adverse events observed | not available | not available | ⨁◯◯◯ Very low | CRITICAL |

**CI:** confidence interval; **RR:** risk ratio; **RCT**: randomized controlled trials

Explanations

a. Some concerns in domain 1 of RoB 2

b. Small sample size

c. High overall risk of bias in RoB 2

#### Proton Pump Inhibitors (PPI) vs No Treatment

##### **Question:** Omeprazole vs No treatment for GERD

| **Certainty assessment** | | | | | | | **№ of patients** | | **Effect** | | **Certainty** | **Importance** |
| --- | --- | --- | --- | --- | --- | --- | --- | --- | --- | --- | --- | --- |
| **Studies** | **Study design** | **Risk of bias** | **Inconsistency** | **Indirectness** | **Imprecision** | **Other considerations** | **Omeprazole** | **No treatment** | **Relative (95% CI)** | **Absolute (95% CI)** |  |  |
| **Vomiting/regurgitation (frequency)** | | | | | | | | | | | | |
| Not reported | | | | | | | | | | | | |
| **Infant Gastro-Esophageal Reflux Questionnaire Revised (I-GERQ-R)** | | | | | | | | | | | | |
| Not reported | | | | | | | | | | | | |
| **Heartburn (frequency)** | | | | | | | | | | | | |
| Not reported | | | | | | | | | | | | |
| **Epigastric pain (frequency)** | | | | | | | | | | | | |
| Not reported | | | | | | | | | | | | |
| **Respiratory symptoms, nocturnal cough, asthma (frequency)** | | | | | | | | | | | | |
| Yagoubi 2022 | RCT | very serious ^a^ | not serious | not serious | serious ^b^ | none | n = 33  Asthma control improved in 22 children (66.7%) over 6 months | n = 26  Asthma control improved in 3 children (11.5%) over 6 months | p < 0.001 | not available | ⨁◯◯◯ Very low | CRITICAL |
| **Severity of esophagitis** | | | | | | | | | | | | |
| Not reported | | | | | | | | | | | | |
| **Adverse events** | | | | | | | | | | | | |
| Not reported | | | | | | | | | | | | |

**CI:** confidence interval; **RR:** risk ratio; **RCT**: randomized controlled trial

Explanations

a. High overall risk of bias in RoB 2

b. Small sample size

#### H2-Antagonists vs Placebo

##### **Question:** Cimetidine vs Placebo for GERD

| **Certainty assessment** | | | | | | | **№ of patients** | | **Effect** | | **Certainty** | **Importance** |
| --- | --- | --- | --- | --- | --- | --- | --- | --- | --- | --- | --- | --- |
| **Studies** | **Study design** | **Risk of bias** | **Inconsistency** | **Indirectness** | **Imprecision** | **Other considerations** | **Cimetidine** | **Placebo** | **Relative (95% CI)** | **Absolute (95% CI)** |  |  |
| **Vomiting/regurgitation (frequency)** | | | | | | | | | | | | |
| Cucchiara 1989 | RCT | very serious ^a^ | not serious | not serious | serious ^b^ | none | n = 17  Clinical score (including vomiting)  Pretrial: Mean 14.6  (SD 3.7)  Post-trial: Mean 5.0  (SD 4.4) | n = 15  Clinical score (including vomiting)  Pretrial: Mean 13.4  (SD 3.8)  Post-trial: Mean 9.5  (SD 4.9) | cimetidine, within group comparison: p< 0.01  placebo, within group comparison: not significant | not estimable | ⨁◯◯◯ Very low | CRITICAL |
| **Infant Gastro-Esophageal Reflux Questionnaire Revised (I-GERQ-R)** | | | | | | | | | | | | |
| Not reported | | | | | | | | | | | | |
| **Heartburn (frequency)** | | | | | | | | | | | | |
| Cucchiara 1989 | RCT | very serious ^a^ | not serious | not serious | serious ^b^ | none | n = 17  Clinical score (including heartburn)  Pretrial: Mean 14.6  (SD 3.7)  Post-trial: Mean 5.0  (SD 4.4) | n = 15  Clinical score (including heartburn)  Pretrial: Mean 13.4  (SD 3.8)  Post-trial: Mean 9.5  (SD 4.9) | cimetidine, within group comparison: p< 0.01  placebo, within group comparison: not significant | not estimable | ⨁◯◯◯ Very low | CRITICAL |
| **Epigastric pain (frequency)** | | | | | | | | | | | | |
| Not reported | | | | | | | | | | | | |
| **Respiratory symptoms, nocturnal cough, asthma (frequency)** | | | | | | | | | | | | |
| Cucchiara 1989 | RCT | very serious ^a^ | not serious | not serious | serious ^b^ | none | n = 17  Clinical score (including pneumonia or asthma)  Pretrial: Mean 14.6  (SD 3.7)  Post-trial: Mean 5.0  (SD 4.4) | n = 15  Clinical score (including pneumonia or asthma)  Pretrial: Mean 13.4  (SD 3.8)  Post-trial: Mean 9.5  (SD 4.9) | cimetidine, within group comparison: p< 0.01  placebo, within group comparison: not significant | not estimable | ⨁◯◯◯ Very low | CRITICAL |
| **Severity of esophagitis** | | | | | | | | | | | | |
| Cucchiara 1989 | RCT | very serious ^a^ | not serious | not serious | serious ^b^ | none | n = 17  Clinical score (including esophagitis)  Pretrial: Mean 14.6  (SD 3.7)  Post-trial: Mean 5.0  (SD 4.4) | n = 15  Clinical score (including esophagitis)  Pretrial: Mean 13.4  (SD 3.8)  Post-trial: Mean 9.5  (SD 4.9) | cimetidine, within group comparison: p< 0.01  placebo, within group comparison: not significant | not estimable | ⨁◯◯◯ Very low | CRITICAL |
| **Adverse events** | | | | | | | | | | | | |
| Cucchiara 1989 | RCT | very serious ^a^ | not serious | not serious | serious ^b^ | none | n = 17  No adverse events observed | n = 15  No adverse events observed | not estimable | not estimable | ⨁◯◯◯ Very low | CRITICAL |

**CI:** confidence interval; **RR:** risk ratio; **RCT**: randomized controlled trial

Explanations

a. High overall risk of bias in RoB 2

b. Small sample size

##### **Question:** Ranitidine vs Placebo for GERD

| **Certainty assessment** | | | | | | | **№ of patients** | | **Effect** | | **Certainty** | **Importance** |
| --- | --- | --- | --- | --- | --- | --- | --- | --- | --- | --- | --- | --- |
| **Studies** | **Study design** | **Risk of bias** | **Inconsistency** | **Indirectness** | **Imprecision** | **Other considerations** | **Ranitidine** | **Placebo** | **Relative (95% CI)** | **Absolute (95% CI)** |  |  |
| **Vomiting/regurgitation (frequency)** | | | | | | | | | | | | |
| Not reported | | | | | | | | | | | | |
| **Infant Gastro-Esophageal Reflux Questionnaire Revised (I-GERQ-R)** | | | | | | | | | | | | |
| Not reported | | | | | | | | | | | | |
| **Heartburn (frequency)** | | | | | | | | | | | | |
| Not reported | | | | | | | | | | | | |
| **Epigastric pain (frequency)** | | | | | | | | | | | | |
| Not reported | | | | | | | | | | | | |
| **Respiratory symptoms, nocturnal cough, asthma (frequency)** | | | | | | | | | | | | |
| Gustafsson 1992 | RCT, cross-over | serious ^a^ | not serious | not serious | serious ^b^ | none | n = 37  No significant reduction of asthma symptoms compared to placebo | n = 37 | not significant | not estimable | ⨁⨁◯◯ Low | CRITICAL |
| **Severity of esophagitis** | | | | | | | | | | | | |
| Not reported | | | | | | | | | | | | |
| **Adverse events** | | | | | | | | | | | | |
| Gustafsson 1992 | RCT, cross-over | serious ^a^ | not serious | not serious | serious ^b^ | none | n = 37  Three patients reported adverse events | n = 37  Five patients reported adverse events | not significant | not estimable | ⨁⨁◯◯ Low | CRITICAL |

**CI:** confidence interval; **RR:** risk ratio; **RCT**: randomized controlled trial

Explanations

a. Some concerns in domains 1 and 5 of RoB 2

b. Small sample size

##### **Question:** Famotidine vs Placebo for GERD

| **Certainty assessment** | | | | | | | **№ of patients** | | **Effect** | | **Certainty** | **Importance** |
| --- | --- | --- | --- | --- | --- | --- | --- | --- | --- | --- | --- | --- |
| **Studies** | **Study design** | **Risk of bias** | **Inconsistency** | **Indirectness** | **Imprecision** | **Other considerations** | **Famotidine** | **Placebo** | **Relative (95% CI)** | **Absolute (95% CI)** |  |  |
| **Vomiting/regurgitation (frequency)** | | | | | | | | | | | | |
| Orenstein 2003 | RCT | very serious ^a^ | not serious | not serious | very serious ^b^ | none | n = 4  no significant differences between famotidine and placebo in regurgitation frequency | n = 4 | not significant | not estimable | ⨁◯◯◯ Very low | CRITICAL |
| **Infant Gastro-Esophageal Reflux Questionnaire Revised (I-GERQ-R)** | | | | | | | | | | | | |
| Not reported | | | | | | | | | | | | |
| **Heartburn (frequency)** | | | | | | | | | | | | |
| Not reported | | | | | | | | | | | | |
| **Epigastric pain (frequency)** | | | | | | | | | | | | |
| Not reported | | | | | | | | | | | | |
| **Respiratory symptoms, nocturnal cough, asthma (frequency)** | | | | | | | | | | | | |
| Not reported | | | | | | | | | | | | |
| **Severity of esophagitis** | | | | | | | | | | | | |
| Not reported | | | | | | | | | | | | |
| **Adverse events** | | | | | | | | | | | | |
| Orenstein 2003 | RCT | very serious ^a^ | not serious | not serious | very serious ^b^ | none | n = 4  One child discontinued due to one adverse event | n = 4 | not significant | not estimable | ⨁◯◯◯ Very low | CRITICAL |

**CI:** confidence interval; **RR:** risk ratio; **RCT**: randomized controlled trial

Explanations

a. High overall risk of bias in RoB 2

b. Very small sample size

##### **Question:** Nizatidine vs Placebo for GERD

| **Certainty assessment** | | | | | | | **№ of patients** | | **Effect** | | **Certainty** | **Importance** |
| --- | --- | --- | --- | --- | --- | --- | --- | --- | --- | --- | --- | --- |
| **Studies** | **Study design** | **Risk of bias** | **Inconsistency** | **Indirectness** | **Imprecision** | **Other considerations** | **Nizatidine** | **Placebo** | **Relative (95% CI)** | **Absolute (95% CI)** |  |  |
| **Vomiting/regurgitation (frequency)** | | | | | | | | | | | | |
| Simeone 1997 | RCT | serious ^a^ | not serious | not serious | serious ^b^ | none | n = 12  Regurgitation, mean (SD): baseline: 2.4 (1.0); at 4 weeks: 1.3 (1.1); at 8 weeks: 0.3 (0.7).  Vomiting, mean (SD): baseline: 2.4 (0.7); at 4 weeks: 0.8 (0.9); at 8 weeks: 0.4 (0.7). | n = 12  Regurgitation, mean (SD): baseline: 2.5 (0.8); at 4 weeks: 2.2 (1.3); at 8 weeks: 1.7 (1.4).  Vomiting, mean (SD): baseline: 2.6 (0.5); at 4 weeks: 2.1 (1.1); at 8 weeks: 1.6 (1.7). | At 8 weeks, compared to baseline, p<0.01 for nizatidine in reducing regurgitation and vomiting; for placebo, p<0.01 only for vomiting. | not estimable | ⨁⨁◯◯  Low | CRITICAL |
| **Infant Gastro-Esophageal Reflux Questionnaire Revised (I-GERQ-R)** | | | | | | | | | | | | |
| Not reported | | | | | | | | | | | | |
| **Heartburn (frequency)** | | | | | | | | | | | | |
| Simeone 1997 | RCT | serious ^a^ | not serious | not serious | serious ^b^ | none | n = 12  Pyrosis, chest pain, mean (SD): baseline: 2.3 (1.2); at 4 weeks: 1.7 (1.1); at 8 weeks: 1.0 (1.7). | n = 12  Pyrosis, chest pain, mean (SD): baseline: 2.2 (0.8); at 4 weeks: 1.8 (0.8); at 8 weeks: 1.6 (0.9). | At 8 weeks, compared to baseline, p<0.01 for nizatidine in reducing pyrosis, chest pain; for placebo, p=not significant. | not estimable | ⨁⨁◯◯  Low | CRITICAL |
| **Epigastric pain (frequency)** | | | | | | | | | | | | |
| Not reported | | | | | | | | | | | | |
| **Respiratory symptoms, nocturnal cough, asthma (frequency)** | | | | | | | | | | | | |
| Not reported | | | | | | | | | | | | |
| **Severity of esophagitis** | | | | | | | | | | | | |
| Simeone 1997 | RCT | serious ^a^ | not serious | not serious | serious ^b^ | none | n = 12  Mild esophagitis pre-treatment: n=8; post-treatment: n=3; Moderate esophagitis pre-treatment: n=4; post-treatment: n=0; | n = 12  Mild esophagitis pre-treatment: n=7; post-treatment: n=4; Moderate esophagitis pre-treatment: n=5; post-treatment: n=5; | not reported | not estimable | ⨁⨁◯◯  Low | CRITICAL |
| **Adverse events** | | | | | | | | | | | | |
| Not reported | | | | | | | | | | | | |

**CI:** confidence interval; **RR:** risk ratio; **RCT**: randomized controlled trial

Explanations

a. Some concerns in domains 1 and 5 of RoB 2

b. Small sample size

#### H2-Antagonists vs Sucralfate

##### **Question:** Cimetidine vs Sucralfate for GERD

| **Certainty assessment** | | | | | | | | | | **№ of patients** | | **Effect** | | **Certainty** | **Importance** |
| --- | --- | --- | --- | --- | --- | --- | --- | --- | --- | --- | --- | --- | --- | --- | --- |
| **Studies** | **Study design** | **Risk of bias** | **Inconsistency** | | **Indirectness** | | **Imprecision** | | **Other considerations** | **Cimetidine** | **Sucralfate** | **Relative (95% CI)** | **Absolute (95% CI)** |  |  |
| **Vomiting/regurgitation (frequency)** | | | | | | | | | | | | | | | |
| Not reported | | | | | | | | | | | | | | | |
| **Infant Gastro-Esophageal Reflux Questionnaire Revised (I-GERQ-R)** | | | | | | | | | | | | | | | |
| Not reported | | | | | | | | | | | | | | | |
| **Heartburn (frequency)** | | | | | | | | | | | | | | | |
| Not reported | | | | | | | | | | | | | | | |
| **Epigastric pain (frequency)** | | | | | | | | | | | | | | | |
| Argüelles-Martin 1989 | RCT | very serious ^a^ | not serious | not serious | | serious ^b^ | | none | | n = 25  Epigastric pain occurred in 15% of patients | Tablets: n = 25  Suspension: n = 25  Epigastric pain occurred in 4% of patients taking the tablet form and in 0% of patients taking the suspension. | p=not significant | not assessable | ⨁◯◯◯ Very low | CRITICAL |
| **Respiratory symptoms, nocturnal cough, asthma (frequency)** | | | | | | | | | | | | | | | |
| Not reported | | | | | | | | | | | | | | | |
| **Severity of esophagitis** | | | | | | | | | | | | | | | |
| Not reported | | | | | | | | | | | | | | | |
| **Adverse events** | | | | | | | | | | | | | | | |
| Argüelles-Martin 1989 | RCT | very serious ^a^ | not serious | | not serious | | serious ^b^ | | none | n = 25  No adverse events reported. | Tablets: n = 25  Suspension: n = 25  No adverse events reported. | not assessable | not assessable | ⨁◯◯◯ Very low | CRITICAL |

**CI:** confidence interval; **RR:** risk ratio; **RCT**: randomized controlled trial

Explanations

a. High overall risk of bias in RoB 2

b. Small sample size

#### Prokinetic agents vs Placebo

##### **Question:** Cisapride vs Placebo for GERD

| **Certainty assessment** | | | | | | | | | | | | | | **№ of patients** | | | | | **Effect** | | | **Certainty** | | **Importance** | |  |  |
| --- | --- | --- | --- | --- | --- | --- | --- | --- | --- | --- | --- | --- | --- | --- | --- | --- | --- | --- | --- | --- | --- | --- | --- | --- | --- | --- | --- |
| **Studies** | | **Study design** | | **Risk of bias** | | **Inconsistency** | | **Indirectness** | | **Imprecision** | | **Other considerations** | | **Cisapride** | **Placebo** | | | | **Relative (95% CI)** | **Absolute (95% CI)** | |  |  |  |  |  |  |
| **Vomiting/regurgitation (frequency)** | | | | | | | | | | | | | | | | | | | | | | | | | |  |  |
| Cohen 1999 | | RCT | | very serious ^a^ | | not serious | | not serious | | serious ^b^ | | none | | n = 38  Mean (SD): 1.4 (0.8) | | | n = 30  Mean (SD): 1.3 (0.8) | | not significant | not reported | | ⨁◯◯◯ Very low | | CRITICAL | |  |  |
| Cucchiara 1987 | | RCT | | very serious ^a^ | | not serious | | not serious | | serious ^b^ | | none | | n = 8  Clinical score (including vomiting or regurgitation): Before treatment, mean (SD): 16.3 (7.1); After treatment 7.9 (7.1) | | | n = 9  Clinical score (including vomiting or regurgitation): Before treatment, mean (SD): 13.5 (4.2); After treatment 10.8 (4.0) | | Cisapride: p<0.01.  Placebo: p=NS | not estimable | | ⨁◯◯◯ Very low | | CRITICAL | |  |  |
| Van Eygen 1989 | | RCT | | serious ^c^ | | not serious | | not serious | | serious ^b^ | | none | | n = 12  Mean Frequency Score: baseline: 2.8; week 4: 0.6 | | | n = 11  Mean Frequency Score: baseline: 2.8; week 4: 1.5 | | p = 0.05 | not estimable | | ⨁⨁◯◯ Low | | CRITICAL | |  |  |
| **Infant Gastro-Esophageal Reflux Questionnaire Revised (I-GERQ-R)** | | | | | | | | | | | | | | | | | | | | | | | | | | | |
| Barnett 2001 | | RCT | | serious ^d^ | | not serious | | not serious | | serious ^b^ | | none | | n = 16  Symptom score, mean (SD): 7.9 (0.7) | | | n = 16  Symptom score, mean (SD): 7.9 (0.5) | | not significant | | | not reported | | ⨁⨁◯◯ Low | | CRITICAL | |
| **Heartburn (frequency)** | | | | | | | | | | | | | | | | | | | | | | | | | | | |
| Not reported | | | | | | | | | | | | | | | | | | | | | | | | | | | |
| **Epigastric pain (frequency)** | | | | | | | | | | | | | | | | | | | | | | | | | | | |
| Not reported | | | | | | | | | | | | | | | | | | | | | | | | | | | |
| **Respiratory symptoms, nocturnal cough, asthma (frequency)** | | | | | | | | | | | | | | | | | | | | | | | | | | | |
| Cohen 1999 | | RCT | | very serious ^a^ | | not serious | | not serious | | serious ^b^ | | none | | n = 38  No significant difference between the two groups in the percentage of days with cough or wheezing symptoms. | | | n = 30 | | not significant | | | not estimable | | ⨁◯◯◯ Very low | | CRITICAL | |
| **Severity of esophagitis** | | | | | | | | | | | | | | | | | | | | | | | | | | | |
| Cucchiara 1987 | | RCT | | very serious ^a^ | | not serious | | not serious | | serious ^b^ | | none | | n = 8  Histological score for oesophagitis: Before treatment, mean (SD): 6.4 (1.9); After treatment 3.0 (3.6) | | | n = 9  Histological score for oesophagitis: Before treatment, mean (SD): 5.7 (1.8); After treatment 4.3 (2.6) | | Cisapride: p<0.05.  Placebo: p=NS  (within-group comparisons) | | | not estimable | | ⨁◯◯◯ Very low | | CRITICAL | |
| **Adverse events** | | | | | | | | | | | | | | | | | | | | | | | | | | | |
| Cohen 1999 | | RCT | | very serious ^a^ | | not serious | | not serious | | serious ^b^ | | none | | n = 50  Adverse events occurred in 42 patients | | | n = 45  Adverse events occurred in 32 patients | | RR 1.18  (0.95 to 1.47) | | | 128 more per 1.000  (from 36 fewer to 334 more) | | ⨁◯◯◯ Very low | | CRITICAL | |
| Cucchiara 1987 | | RCT | | very serious ^a^ | | not serious | | not serious | | serious ^b^ | | none | | n = 8  No adverse events reported. | | | n = 9  No adverse events reported. | | not estimable | | | not estimable | | ⨁◯◯◯ Very low | | RCT | |
| Saye 1987 | | RCT | | serious ^c^ | | not serious | | not serious | | serious ^b^ | | none | | n = 14  No adverse events reported. | | | n = 14  No adverse events reported. | | not estimable | | | not estimable | | ⨁⨁◯◯ Low | | CRITICAL | |
| Scott 1997 | | RCT | | serious ^e^ | | not serious | | not serious | | serious ^b^ | | none | | n = 23  Adverse events occurred in 14 patients | | | n = 26  Adverse events occurred in 13 patients | | not significant | | | not reported | | ⨁⨁◯◯ Low | | CRITICAL | |
| Van Eygen 1989 | | RCT | | serious ^c^ | | not serious | | not serious | | serious ^b^ | | none | | n = 12  1 patient had diarrhea | | | n = 11  2 patients had diarrhea | | not reported | | | not reported | | ⨁⨁◯◯ Low | | CRITICAL | |

**CI:** confidence interval; **RR:** risk ratio; **RCT**: randomized controlled trial

Explanations

a. High overall risk of bias in RoB 2

b. Small sample size

c. Some concerns in domains 1 and 5 of RoB 2

d. Some concerns in domain 1 of RoB 2

e. Some concerns in domain 5 of RoB 2

##### **Question:** Domperidone vs Placebo for GERD

| **Certainty assessment** | | | | | | | | **№ of patients** | | **Effect** | | | **Certainty** | **Importance** |
| --- | --- | --- | --- | --- | --- | --- | --- | --- | --- | --- | --- | --- | --- | --- |
| **Studies** | **Study design** | | **Risk of bias** | **Inconsistency** | **Indirectness** | **Imprecision** | **Other considerations** | **Domperidone** | **Placebo** | **Relative (95% CI)** | | **Absolute (95% CI)** |  |  |
| **Vomiting/regurgitation (frequency)** | | | | | | | | | | | | | | |
| Clara 1979 | | RCT | serious ^a^ | not serious | not serious | serious ^b^ | none | n = 14  Number of patients without vomiting at 4 weeks: 10  Number of patients without regurgitation at 4 weeks: 12 | n = 18  Number of patients without vomiting at 4 weeks: 3  Number of patients without regurgitation at 4 weeks: 7 | Vomiting: p<0.05  Regurgitation: p<0.01 | not reported | | ⨁⨁◯◯ Low | CRITICAL |
| De Loore 1979 | | RCT | serious ^c^ | not serious | not serious | serious ^b^ | none | n = 15  Number of patients without vomiting at 2 weeks: 11 | n = 15  Number of patients without vomiting at 2 weeks: 1 | p<0.001 | not reported | | ⨁⨁◯◯ Low | CRITICAL |
| **Infant Gastro-Esophageal Reflux Questionnaire Revised (I-GERQ-R)** | | | | | | | | | | | | | | |
| Not reported | | | | | | | | | | | | | | |
| **Heartburn (frequency)** | | | | | | | | | | | | | | |
| Not reported | | | | | | | | | | | | | | |
| **Epigastric pain (frequency)** | | | | | | | | | | | | | | |
| Not reported | | | | | | | | | | | | | | |
| **Respiratory symptoms, nocturnal cough, asthma (frequency)** | | | | | | | | | | | | | | |
| Not reported | | | | | | | | | | | | | | |
| **Severity of esophagitis** | | | | | | | | | | | | | | |
| Not reported | | | | | | | | | | | | | | |
| **Adverse events** | | | | | | | | | | | | | | |
| Bines 1992 | RCT | | serious ^a^ | not serious | not serious | serious ^b^ | none | n = 8  Diarrhea occurred in 4 patients | n = 9  Diarrhea occurred in 2 patients | not reported | | not reported | ⨁⨁◯◯ Low | CRITICAL |
| Carroccio 1994 | RCT | | serious ^d^ | not serious | not serious | serious ^b^ | none | n = 20  No adverse events reported. | n = 20  No adverse events reported. | not estimable | | not reported | ⨁⨁◯◯ Low | CRITICAL |
| Clara 1979 | RCT | | serious ^c^ | not serious | not serious | serious ^b^ | none | n = 14  No adverse events reported. | n = 18  No adverse events reported. | not estimable | | not reported | ⨁⨁◯◯ Low | CRITICAL |
| De Loore 1979 | RCT | | serious ^c^ | not serious | not serious | serious ^b^ | none | n = 15  No adverse events reported. | n = 15  No adverse events reported. | not estimable | | not reported | ⨁⨁◯◯ Low | CRITICAL |

**CI:** confidence interval; **RR:** risk ratio; **RCT**: randomized controlled trial

Explanations

a. Some concerns in domain 1 of RoB 2

b. Small sample size

c. Some concerns in domains 1 and 5 of RoB 2

d. Some concerns in domain 5 of RoB 2

##### **Question:** Metoclopramide vs Placebo for GERD

| **Certainty assessment** | | | | | | | | | | | **№ of patients** | | | **Effect** | | **Certainty** | **Importance** |
| --- | --- | --- | --- | --- | --- | --- | --- | --- | --- | --- | --- | --- | --- | --- | --- | --- | --- |
| **Studies** | | **Study design** | **Risk of bias** | **Inconsistency** | | | **Indirectness** | **Imprecision** | **Other considerations** | | **Metoclopramide** | | **Placebo** | **Relative (95% CI)** | **Absolute (95% CI)** |  |  |
| **Vomiting/regurgitation (frequency)** | | | | | | | | | | | | | | | | | |
| De Loore 1979 | RCT | | serious ^a^ | | not serious | not serious | | serious ^b^ | none | n = 17  Number of patients without vomiting at 2 weeks: 7 | | n = 15  Number of patients without vomiting at 2 weeks: 1 | | p<0.001 | not reported | ⨁⨁◯◯ Low | CRITICAL |
| **Infant Gastro-Esophageal Reflux Questionnaire Revised (I-GERQ-R)** | | | | | | | | | | | | | | | | | |
| Not reported | | | | | | | | | | | | | | | | | |
| **Heartburn (frequency)** | | | | | | | | | | | | | | | | | |
| Not reported | | | | | | | | | | | | | | | | | |
| **Epigastric pain (frequency)** | | | | | | | | | | | | | | | | | |
| Not reported | | | | | | | | | | | | | | | | | |
| **Respiratory symptoms, nocturnal cough, asthma (frequency)** | | | | | | | | | | | | | | | | | |
| Not reported | | | | | | | | | | | | | | | | | |
| **Severity of esophagitis** | | | | | | | | | | | | | | | | | |
| Not reported | | | | | | | | | | | | | | | | | |
| **Adverse events** | | | | | | | | | | | | | | | | | |
| Bellissant 1997 | | RCT | very serious ^c^ | not serious | | | not serious | serious ^b^ | none | | n = 19  Adverse events occurred in 3 patients | | n = 20  Adverse events occurred in 1 patient | not significant | not reported | ⨁◯◯◯ Very low | CRITICAL |
| Forbes 1986 | | RCT | serious ^a^ | not serious | | | not serious | serious ^b^ | none | | n = 10  No adverse events reported. | | n = 10  No adverse events reported. | not estimable | not reported | ⨁⨁◯◯ Low | CRITICAL |
| Machida 1988 | | RCT | very serious ^c^ | not serious | | | not serious | serious ^b^ | none | | n = 3  Adverse events occurred in 3 patients | | n = 5  No adverse events reported. | not significant | not reported | ⨁◯◯◯ Very low | CRITICAL |
| Tolia 1989 | | RCT | serious ^d^ | not serious | | | not serious | serious ^b^ | none | | n = 30  No adverse events reported. | | n = 30  No adverse events reported. | not estimable | not reported | ⨁⨁◯◯ Low | CRITICAL |
| De Loore 1979 | | RCT | serious ^a^ | not serious | | | not serious | serious ^b^ | none | | n = 17  No adverse events reported. | | n = 15  No adverse events reported. | not estimable | not reported | ⨁⨁◯◯ Low | CRITICAL |

**CI:** confidence interval; **RR:** risk ratio; **RCT**: randomized controlled trial

Explanations

a. Some concerns in domains 1 and 5 of RoB 2

b. Small sample size

c. High overall risk of bias in RoB 2

d. Some concerns in domain 5 of RoB 2

#### Bethanechol vs Placebo

##### **Question:** Bethanechol vs Placebo for GERD

| **Certainty assessment** | | | | | | | **№ of patients** | | **Effect** | | **Certainty** | **Importance** |
| --- | --- | --- | --- | --- | --- | --- | --- | --- | --- | --- | --- | --- |
| **Studies** | **Study design** | **Risk of bias** | **Inconsistency** | **Indirectness** | **Imprecision** | **Other considerations** | **Bethanechol** | **Placebo** | **Relative (95% CI)** | **Absolute (95% CI)** |  |  |
| **Vomiting/regurgitation (frequency)** | | | | | | | | | | | | |
| Not reported | | | | | | | | | | | | |
| **Infant Gastro-Esophageal Reflux Questionnaire Revised (I-GERQ-R)** | | | | | | | | | | | | |
| Not reported | | | | | | | | | | | | |
| **Heartburn (frequency)** | | | | | | | | | | | | |
| Not reported | | | | | | | | | | | | |
| **Epigastric pain (frequency)** | | | | | | | | | | | | |
| Not reported | | | | | | | | | | | | |
| **Respiratory symptoms, nocturnal cough, asthma (frequency)** | | | | | | | | | | | | |
| Not reported | | | | | | | | | | | | |
| **Severity of esophagitis** | | | | | | | | | | | | |
| Not reported | | | | | | | | | | | | |
| **Adverse events** | | | | | | | | | | | | |
| Euler 1980 | RCT | serious ^a^ | not serious | not serious | serious ^b^ | none | n = 15  Adverse events occurred in 1 patients | n = 15  Adverse events occurred in 13 patients | not significant | not reported | ⨁⨁◯◯ Low | CRITICAL |

**CI:** confidence interval; **RR:** risk ratio; **RCT**: randomized controlled trial

Explanations

a. Some concerns in domain 5 of RoB 2

b. Small sample size

### PICO 6 - What is the effectiveness of different non-pharmacologic treatment options for GER/GERD?

#### Dietary modifications

##### **Question:** Novel anti-regurgitation formula with fermented formula vs. Regular anti-regurgitation formula for GERD

| **Certainty assessment** | | | | | | | **№ of patients** | | **Effect** | | **Certainty** | **Importance** |  |
| --- | --- | --- | --- | --- | --- | --- | --- | --- | --- | --- | --- | --- | --- |
| **Studies** | **Study design** | **Risk of bias** | **Inconsistency** | **Indirectness** | **Imprecision** | **Other considerations** | **Novel formula** | **Standard formula** | **Relative (95% CI)** | **Absolute (95% CI)** |  |  |  |
| **Vomiting/regurgitation (frequency)** | | | | | | | | | | | | |  |
| Not reported | | | | | | | | | | | | |  |
| **Infant Gastrointestinal Symptom Questionnaire (IGSQ)** | | | | | | | | | | | | |  |
| Bellaiche 2021 | RCT | serious ^a^ | not serious | not serious | not serious | none | n = 86  IGSQ sum scores improved from on average 36 at Baseline to 23 at Week 4.  Difference in estimated means between formulas: -1.41, 90% CI [-3.04; 0.22] | n = 80 | not significant | not estimable | ⨁⨁⨁◯ Moderate | CRITICAL |  |
| **Total number of reflux events** | | | | | | | | | | | | |  |
| Not reported | | | | | | | | | | | | |  |
| **Estimated volume regurgitated** | | | | | | | | | | | | |  |
| Not reported | | | | | | | | | | | | |  |
| **Respiratory symptoms, nocturnal cough, asthma (frequency)** | | | | | | | | | | | | |  |
| Not reported | | | | | | | | | | | | |  |
| **Weight gain** | | | | | | | | | | | | |  |
| Not reported | | | | | | | | | | | | |  |
| **Adverse events** | | | | | | | | | | | | |  |
| Bellaiche 2021 | RCT | serious ^a^ | not serious | not serious | not serious | none | n = 86  Number of children with any adverse events: n = 29 (31.5%) | n = 80  Number of children with any adverse events: n = 33 (37.1%) | p = 0.439 | not reported | ⨁⨁⨁◯ Moderate | CRITICAL |  |

**CI:** confidence interval; **RCT**: randomized controlled trial

Explanations

a. Some concerns in domain 1 of RoB 2

##### **Question:** Cornstarch-thickened AR-formula vs. Regular formula for GERD

| **Certainty assessment** | | | | | | | **№ of patients** | | **Effect** | | **Certainty** | **Importance** |
| --- | --- | --- | --- | --- | --- | --- | --- | --- | --- | --- | --- | --- |
| **Studies** | **Study design** | **Risk of bias** | **Inconsistency** | **Indirectness** | **Imprecision** | **Other considerations** | **Cornstarch-thickened AR-formula** | **Regular formula** | **Relative (95% CI)** | **Absolute (95% CI)** |  |  |
| **Vomiting/regurgitation (frequency)** | | | | | | | | | | | | |
| Chao 2007  (Diseases of the Esophagus (2007) 20, 155–160) | RCT | very serious ^a^ | not serious | not serious | serious ^b^ | none | n = 41  Frequency of regurgitation / vomiting before trial, mean (SD): 4.19 (1.71);  After trial (8 weeks): 0.93 (0.42) | n = 40  Frequency of regurgitation / vomiting before trial, mean (SD): 4.15 (1.68);  After trial (8 weeks): 2.89 (1.16) | p < 0.001 | not estimable | ⨁◯◯◯ Very low | CRITICAL |
| Moukarzel 2007 | RCT | very serious ^a^ | not serious | not serious | serious ^b^ | none | n = 28  Vomiting, baseline, mean (SD): 2.6 (2.6); After treatment: 0.5 (0.8).  Regurgitation, mean (SD): 7.1 (3.9); After treatment: 2.3 (2.0). | n = 32  Vomiting, baseline, mean (SD): 2.1 (3.0); After treatment: 1.2 (1.1).  Regurgitation, mean (SD): 6.5 (3.7); After treatment: 5.2 (3.1). | Intervention: Vomiting: p = 0.0003; regurgitation: p = 0.0009  Control: Vomiting: p = 0.11; regurgitation: p = 0.13 | not estimable | ⨁◯◯◯ Very low | CRITICAL |
| Ramirez-Mayans 2003 | RCT | serious ^c^ | not serious | not serious | serious ^b^ | none | n = 28  Vomit, baseline, episodes / 24h: 2.8 / 2.6; After treatment: 1.5 / 7.4.  Regurgitation, episodes / 24h: 4.3 / 2.8; After treatment: 2.5 / 4.6. | n = 24  Vomit, baseline, episodes / 24h: 3.7 / 2.2; After treatment: 5.4 / 8.3.  Regurgitation, episodes / 24h: 4.9 / 2.5; After treatment: 3.4 / 6.0. | Intervention: Vomit: p = 0.04; regurgitation: p = 0.03  Control: Vomit: p = NS; regurgitation: p = 0.027 | not estimable | ⨁⨁◯◯ Low | CRITICAL |
| Xinias 2005 | RCT | not serious | not serious | not serious | serious ^b^ | none | n = 51  Episodes of regurgitation/day, mean (SD)  Baseline 5.60 (4.15); After 4 weeks: 2.57 (2.71).  Episodes of vomiting/day, mean (SD)  Baseline 4.34 (2.42); After 4 weeks: 1.45 (1.65) | n = 45  Episodes of regurgitation/day, mean (SD)  Baseline, 4.77 (2.35); After 4 weeks: 4.31 (2.01).  Episodes of vomiting/day, mean (SD)  Baseline 3.09 (1.24); After 4 weeks: 2.74 (1.37) | Regurgitation: thickened, p<0.0001; regular, p=0.36.  Vomiting: thickened, p<0.0001; regular, p=0.39. | not estimable | ⨁⨁⨁◯ Moderate | CRITICAL |
| **Infant Gastro-Esophageal Reflux Questionnaire Revised (I-GERQ-R)** | | | | | | | | | | | | |
| Not reported | | | | | | | | | | | | |
| **Total number of reflux events** | | | | | | | | | | | | |
| Xinias 2005 | RCT | not serious | not serious | not serious | serious ^b^ | none | n = 51  Episodes of reflux/hour, mean (SD)  Baseline 11.5 (13.3); After 4 weeks: 6.2 (10.2). | n = 45  Episodes of reflux/hour, mean (SD)  Baseline, 10.6 (5.8); After 4 weeks: 8.7 (4.9). | Thickened, p<0.001; Regular, p=0.09. | not estimable | ⨁⨁⨁◯ Moderate | CRITICAL |
| **Estimated volume regurgitated** | | | | | | | | | | | | |
| Not reported | | | | | | | | | | | | |
| **Respiratory symptoms, nocturnal cough, asthma (frequency)** | | | | | | | | | | | | |
| Chao 2007  (Diseases of the Esophagus (2007) 20, 155–160) | RCT | very serious ^a^ | not serious | not serious | serious ^b^ | none | n = 41  Cough: before trial: n = 5;  After trial: n = 0 | n = 40  Cough: before trial: n = 4;  After trial: n = 2 | not reported | not estimable | ⨁◯◯◯ Very low | CRITICAL |
| **Weight gain** | | | | | | | | | | | | |
| Chao 2007  (Diseases of the Esophagus (2007) 20, 155–160) | RCT | very serious ^a^ | not serious | not serious | serious ^b^ | none | n = 41  Weight gain after two months, mean (SD), grams: 1629.5 (215.8) | n = 40  Weight gain after two months, mean (SD), grams: 1321.7 (193.7) | p < 0.001 | not estimable | ⨁◯◯◯ Very low | CRITICAL |
| Xinias 2005 | RCT | not serious | not serious | not serious | serious ^b^ | none | n = 51  Mean (SD), grams:746 (246) | n = 45  Mean (SD), grams: 642 (229) | p = 0.08 | not estimable | ⨁⨁⨁◯ Moderate | CRITICAL |
| **Adverse events** | | | | | | | | | | | | |
| Moukarzel 2007 | RCT | very serious ^a^ | not serious | not serious | serious ^b^ | none | n = 28  No adverse events reported | n = 32  No adverse events reported | not estimable | not estimable | ⨁◯◯◯ Very low | CRITICAL |
| Ramirez-Mayans 2003 | RCT | serious ^c^ | not serious | not serious | serious ^b^ | none | n = 28  No adverse events reported | n = 24  Constipation: n = 3 | not reported | not estimable | ⨁⨁◯◯ Low | CRITICAL |
| Xinias 2005 | RCT | not serious | not serious | not serious | serious ^b^ | none | n = 51  No adverse events reported | n = 45  No adverse events reported | not estimable | not estimable | ⨁⨁⨁◯ Moderate | CRITICAL |

**CI:** confidence interval; **SD:** standard deviation; **RCT**: randomized controlled trial; **NS**: not significant.

Explanations

a. High overall risk of bias in RoB 2

b. Small sample size

c. Some concerns in domain 1 of RoB 2

##### **Question:** Cereal-thickened AR-formula vs. Regular formula and positional therapy for GERD

| **Certainty assessment** | | | | | | | **№ of patients** | | **Effect** | | **Certainty** | **Importance** |
| --- | --- | --- | --- | --- | --- | --- | --- | --- | --- | --- | --- | --- |
| **Studies** | **Study design** | **Risk of bias** | **Inconsistency** | **Indirectness** | **Imprecision** | **Other considerations** | **Cereal-thickened AR-formula** | **Regular formula and positional therapy** | **Relative (95% CI)** | **Absolute (95% CI)** |  |  |
| **Vomiting/regurgitation (frequency)** | | | | | | | | | | | | |
| Chao 2007  (Nutrition 23 (2007) 23–28) | RCT | very serious ^a^ | not serious | not serious | serious ^b^ | none | n = 31  Vomiting / regurgitation, baseline, mean (SD): 3.71 (0.69); After treatment (8 weeks): 1.61 (0.76). | n = 32  Vomiting / regurgitation, baseline, mean (SD): 3.69 (0.74); After treatment (8 weeks): 2.38 (0.83). | Intervention: p < 0.001  Control: p = 0.028 | not estimable | ⨁◯◯◯ Very low | CRITICAL |
| **Infant Gastro-Esophageal Reflux Questionnaire Revised (I-GERQ-R)** | | | | | | | | | | | | |
| Not reported | | | | | | | | | | | | |
| **Total number of reflux events** | | | | | | | | | | | | |
| Not reported | | | | | | | | | | | | |
| **Estimated volume regurgitated** | | | | | | | | | | | | |
| Not reported | | | | | | | | | | | | |
| **Respiratory symptoms, nocturnal cough, asthma (frequency)** | | | | | | | | | | | | |
| Not reported | | | | | | | | | | | | |
| **Weight gain** | | | | | | | | | | | | |
| Chao 2007  (Nutrition 23 (2007) 23–28) | RCT | very serious ^a^ | not serious | not serious | serious ^b^ | none | n = 31  Weight gain after 8 weeks, mean (SD), grams: 1261 (131) | n = 32  Weight gain after 8 weeks, mean (SD), grams: 1121 (137) | p < 0.001 | not estimable | ⨁◯◯◯ Very low | CRITICAL |
| **Adverse events** | | | | | | | | | | | | |
| Not reported | | | | | | | | | | | | |

**CI:** confidence interval; **SD:** standard deviation; **RCT**: randomized controlled trial

Explanations

a. High overall risk of bias in RoB 2

b. Small sample size

##### **Question:** Fortified milk thickened with starch vs. Fortified milk for GERD

| **Certainty assessment** | | | | | | | **№ of patients** | | **Effect** | | **Certainty** | **Importance** |
| --- | --- | --- | --- | --- | --- | --- | --- | --- | --- | --- | --- | --- |
| **Studies** | **Study design** | **Risk of bias** | **Inconsistency** | **Indirectness** | **Imprecision** | **Other considerations** | **Fortified milk thickened with starch** | **Fortified milk** | **Relative (95% CI)** | **Absolute (95% CI)** |  |  |
| **Vomiting/regurgitation (frequency)** | | | | | | | | | | | | |
| Not reported | | | | | | | | | | | | |
| **Infant Gastro-Esophageal Reflux Questionnaire Revised (I-GERQ-R)** | | | | | | | | | | | | |
| Not reported | | | | | | | | | | | | |
| **Total number of reflux events** | | | | | | | | | | | | |
| Corvaglia 2006 | RCT | serious ^a^ | not serious | not serious | very serious ^b^ | none | n = 5  Mean (SD): 29.2 (5.7) | n = 5  Mean (SD): 34.0 (12.1) | not significant | not estimable | ⨁◯◯◯ Very low | CRITICAL |
| **Estimated volume regurgitated** | | | | | | | | | | | | |
| Not reported | | | | | | | | | | | | |
| **Respiratory symptoms, nocturnal cough, asthma (frequency)** | | | | | | | | | | | | |
| Not reported | | | | | | | | | | | | |
| **Weight gain** | | | | | | | | | | | | |
| Not reported | | | | | | | | | | | | |
| **Adverse events** | | | | | | | | | | | | |
| Not reported | | | | | | | | | | | | |

**CI:** confidence interval; **SD:** standard deviation; **RCT**: randomized controlled trial

Explanations

a. Some concerns in domains 1, 3, and 5 of RoB 2

b. Very small sample size

##### **Question:** Hydrolyzed protein formulas (HPFs) vs. Standard protein formula (SPF) for GERD

| **Certainty assessment** | | | | | | | **№ of patients** | | **Effect** | | **Certainty** | **Importance** |
| --- | --- | --- | --- | --- | --- | --- | --- | --- | --- | --- | --- | --- |
| **Studies** | **Study design** | **Risk of bias** | **Inconsistency** | **Indirectness** | **Imprecision** | **Other considerations** | **Hydrolyzed protein formulas** | **Standard protein formula** | **Relative (95% CI)** | **Absolute (95% CI)** |  |  |
| **Vomiting/regurgitation (frequency)** | | | | | | | | | | | | |
| Not reported | | | | | | | | | | | | |
| **Infant Gastro-Esophageal Reflux Questionnaire Revised (I-GERQ-R)** | | | | | | | | | | | | |
| Not reported | | | | | | | | | | | | |
| **Total number of reflux events** | | | | | | | | | | | | |
| Corvaglia 2013 | RCT | serious ^a^ | not serious | not serious | serious ^b^ | none | n = 18  Median (range): 54.5 (25–142) | n = 18  Median (range): 67.5 (18–192) | not significant | not estimable | ⨁⨁◯◯ Low | CRITICAL |
| **Estimated volume regurgitated** | | | | | | | | | | | | |
| Not reported | | | | | | | | | | | | |
| **Respiratory symptoms, nocturnal cough, asthma (frequency)** | | | | | | | | | | | | |
| Not reported | | | | | | | | | | | | |
| **Weight gain** | | | | | | | | | | | | |
| Not reported | | | | | | | | | | | | |
| **Adverse events** | | | | | | | | | | | | |
| Corvaglia 2013 | RCT | serious ^a^ | not serious | not serious | serious ^b^ | none | n = 18  No adverse events reported | n = 18  No adverse events reported | not estimable | not estimable | ⨁⨁◯◯ Low | CRITICAL |

**CI:** confidence interval; **RCT**: randomized controlled trial

Explanations

a. Some concerns in domains 1 and 5 of RoB 2

b. Small sample size

##### **Question:** Formula thickened with rice cereals vs. Formula thickened with bean gum vs. Regular formula for GERD

| **Certainty assessment** | | | | | | | **№ of patients** | | **Effect** | | **Certainty** | **Importance** |
| --- | --- | --- | --- | --- | --- | --- | --- | --- | --- | --- | --- | --- |
| **Studies** | **Study design** | **Risk of bias** | **Inconsistency** | **Indirectness** | **Imprecision** | **Other considerations** | **Formula thickened with rice cereals** | **Formula thickened with bean gum / Regular formula** | **Relative (95% CI)** | **Absolute (95% CI)** |  |  |
| **Vomiting/regurgitation (frequency)** | | | | | | | | | | | | |
| Hegar 2008 | RCT | very serious ^a^ | not serious | not serious | serious ^b^ | none | n = 20  Regurgitation, baseline, mean (SD): 5.7 (1.9); After treatment (week 4): 2.1 (2.1). | Bean gum: n = 20  Regurgitation, baseline, mean (SD): 5.9 (1.7); After treatment (week 4): 3.3 (2.3).  Regular: n = 20  Regurgitation, baseline, mean (SD): 5.5 (1.8); After treatment (week 4): 1.3 (1.4). | Within-group comparisons: all significant (p<0.005)  Between-group comparisons: not significant | not estimable | ⨁◯◯◯ Very low | CRITICAL |
| **Infant Gastro-Esophageal Reflux Questionnaire Revised (I-GERQ-R)** | | | | | | | | | | | | |
| Not reported | | | | | | | | | | | | |
| **Total number of reflux events** | | | | | | | | | | | | |
| Not reported | | | | | | | | | | | | |
| **Estimated volume regurgitated** | | | | | | | | | | | | |
| Not reported | | | | | | | | | | | | |
| **Respiratory symptoms, nocturnal cough, asthma (frequency)** | | | | | | | | | | | | |
| Not reported | | | | | | | | | | | | |
| **Weight gain** | | | | | | | | | | | | |
| Hegar 2008 | RCT | very serious ^a^ | not serious | not serious | serious ^b^ | none | n = 20  Weight gain after 8 weeks, mean (SD), grams: 575 (97) | Bean gum: n = 20  Weight gain after 8 weeks, mean (SD), grams: 965 (166)  Regular: n = 20  Weight gain after 8 weeks, mean (SD), grams: 828 (160) | Bean gum vs rice cereals or regular formula: p < 0.001 | not estimable | ⨁◯◯◯ Very low | CRITICAL |
| **Adverse events** | | | | | | | | | | | | |
| Not reported | | | | | | | | | | | | |

**CI:** confidence interval; **SD:** standard deviation; **RCT**: randomized controlled trial

Explanations

a. High overall risk of bias in RoB 2

b. Small sample size

##### **Question:** Thickened formula with locust bean gum vs. Regular formula for GERD

| **Certainty assessment** | | | | | | | **№ of patients** | | **Effect** | | **Certainty** | **Importance** |
| --- | --- | --- | --- | --- | --- | --- | --- | --- | --- | --- | --- | --- |
| **Studies** | **Study design** | **Risk of bias** | **Inconsistency** | **Indirectness** | **Imprecision** | **Other considerations** | **Thickened formula with locust bean gum** **(HL-450) vs Regular formula (HL-00)** | **Thickened formula with locust bean gum** **(HL-350) vs Regular formula (HL-00)** | **Relative (95% CI)** | **Absolute (95% CI)** |  |  |
| **Vomiting/regurgitation (frequency)** | | | | | | | | | | | | |
| Miyazawa 2004 | RCT | very serious ^a^ | not serious | not serious | serious ^b^ | none | HL-450/HL-00: n = 16  Regurgitation episodes, median (IQR): HL-450: 1.6 (0.8-2.0).  HL-00: 3.5 (2.3-4.9) | HL-350/HL-00: n = 11  Regurgitation episodes, median (IQR): HL-450: 1.3 (0.6-2.3).  HL-00: 2.9 (2.0-3.2) | not reported | not estimable | ⨁◯◯◯ Very low | CRITICAL |
| Miyazawa 2006 | RCT | very serious ^a^ | not serious | not serious | serious ^b^ | none | HL-450/HL-00: n = 14  Regurgitation episodes, mean (SD): HL-450: 12.8 (3.0).  HL-00: 29.8 (3.6) | HL-350/HL-00: n = 13  Regurgitation episodes, mean (SD): HL-450: 12.9 (3.5).  HL-00: 22.6 (3.9) | HL-450 vs HL-00: p = 0.0015  HL-350 vs HL-00: p = 0.018 | not estimable | ⨁◯◯◯ Very low | CRITICAL |
| Miyazawa 2007 | RCT | very serious ^a^ | not serious | not serious | serious ^b^ | none | HL-350: n = 10  Regurgitation episodes, median (IQR): 2.3 (1.6-3.6). | HL-00: n = 10  Regurgitation episodes, median (IQR): 5.2 (3.7-7.8). | p < 0.01 | not estimable | ⨁◯◯◯ Very low | CRITICAL |
| **Infant Gastro-Esophageal Reflux Questionnaire Revised (I-GERQ-R)** | | | | | | | | | | | | |
| Not reported | | | | | | | | | | | | |
| **Total number of reflux events** | | | | | | | | | | | | |
| Not reported | | | | | | | | | | | | |
| **Estimated volume regurgitated** | | | | | | | | | | | | |
| Miyazawa 2004 | RCT | very serious ^a^ | not serious | not serious | serious ^b^ | none | HL-450/HL-00: n = 16  Score 1 = minimal regurgitation, was less in HL-450 than HL-00; Scores 2-4 were similar between groups. | HL-350/HL-00: n = 11  Scores 1-4 were similar between groups. | HL.450 vs HL-00, score 1: p = 0.03 | not estimable | ⨁◯◯◯ Very low | CRITICAL |
| Miyazawa 2006 | RCT | very serious ^a^ | not serious | not serious | serious ^b^ | none | HL-450/HL-00: n = 14  No differences | HL-350/HL-00: n = 13  No differences | Not significant | not estimable | ⨁◯◯◯ Very low | CRITICAL |
| **Respiratory symptoms, nocturnal cough, asthma (frequency)** | | | | | | | | | | | | |
| Not reported | | | | | | | | | | | | |
| **Weight gain** | | | | | | | | | | | | |
| Miyazawa 2004 | RCT | very serious ^a^ | not serious | not serious | serious ^b^ | none | HL-450/HL-00: n = 16  Weight gain, grams/day, median (IQR): HL-450: 20.7 (14.3-26.1).  HL-00: 20.7 (10.5-31.0) | HL-350/HL-00: n = 11  Weight gain, grams/day, median (IQR): HL-350: 29.3 (23.2-32.8).  HL-00: 13.2 (7.9-21.5) | HL-450 vs HL-00: p = 0.76  HL-350 vs HL-00: p = 0.03 | not estimable | ⨁◯◯◯ Very low | CRITICAL |
| Miyazawa 2007 | RCT | very serious ^a^ | not serious | not serious | serious ^b^ | none | HL-350: n = 10  Weight gain, grams/day, median (IQR): 30.6 (20.4-37.9). | HL-00: n = 10  Weight gain, grams/day, median (IQR): 20.8 (13.2-29.6). | not significant | not estimable | ⨁◯◯◯ Very low | CRITICAL |
| **Adverse events** | | | | | | | | | | | | |
| Miyazawa 2007 | RCT | very serious ^a^ | not serious | not serious | serious ^b^ | none | HL-350: n = 10  No adverse events reported | HL-00: n = 10  No adverse events reported | p < 0.01 | not estimable | ⨁◯◯◯ Very low | CRITICAL |
| Miyazawa 2004 | RCT | very serious ^a^ | not serious | not serious | serious ^b^ | none | HL-450/HL-00: n = 16  No adverse events reported | HL-350/HL-00: n = 11  No adverse events reported | HL-450 vs HL-00: p = 0.76  HL-350 vs HL-00: p = 0.03 | not estimable | ⨁◯◯◯ Very low | CRITICAL |
| Miyazawa 2006 | RCT | very serious ^a^ | not serious | not serious | serious ^b^ | none | HL-450/HL-00: n = 14  Increase in bowel movements: n = 1 | HL-350/HL-00: n = 13  Increase in bowel movements: n = 2 | not significant | not reported | ⨁◯◯◯ Very low | CRITICAL |

**CI:** confidence interval; **IQR:** inter-quartile range; **RCT**: randomized controlled trial

Explanations

a. High overall risk of bias in RoB 2

b. Small sample size

##### **Question:** Soy-based formula vs. Bovine milk-based formula for GERD

| **Certainty assessment** | | | | | | | **№ of patients** | | **Effect** | | **Certainty** | **Importance** |
| --- | --- | --- | --- | --- | --- | --- | --- | --- | --- | --- | --- | --- |
| **Studies** | **Study design** | **Risk of bias** | **Inconsistency** | **Indirectness** | **Imprecision** | **Other considerations** | **Soy-based formula** | **Bovine milk-based formula** | **Relative (95% CI)** | **Absolute (95% CI)** |  |  |
| **Vomiting/regurgitation (frequency)** | | | | | | | | | | | | |
| Ostrom 2006 | RCT | serious ^a^ | not serious | not serious | not serious | none | n = 89  Number of daily regurgitations, mean (SE): Baseline: 3.9 (0.2): 4 weeks: 2.0 (0.2) | n = 90  Number of daily regurgitations, mean (SE): Baseline: 3.6 (0.2): 4 weeks: 2.4 (0.4) | not significant | not estimable | ⨁⨁⨁◯ Moderate | CRITICAL |
| **Infant Gastro-Esophageal Reflux Questionnaire Revised (I-GERQ-R)** | | | | | | | | | | | | |
| Not reported | | | | | | | | | | | | |
| **Total number of reflux events** | | | | | | | | | | | | |
| Not reported | | | | | | | | | | | | |
| **Estimated volume regurgitated** | | | | | | | | | | | | |
| Not reported | | | | | | | | | | | | |
| **Respiratory symptoms, nocturnal cough, asthma (frequency)** | | | | | | | | | | | | |
| Not reported | | | | | | | | | | | | |
| **Weight gain** | | | | | | | | | | | | |
| Ostrom 2006 | RCT | serious ^a^ | not serious | not serious | not serious | none | n = 89  Weight gain: 32-33 grams/day | n = 90  Weight gain: 32-33 grams/day | not significant | not estimable | ⨁⨁⨁◯ Moderate | CRITICAL |
| **Adverse events** | | | | | | | | | | | | |
| Ostrom 2006 | RCT | very serious ^b^ | not serious | not serious | not serious | none | n = 89  Serious adverse events: n = 4 | n = 90  Serious adverse events: n = 1 | not significant | not estimable | ⨁⨁◯◯ Low | CRITICAL |

**CI:** confidence interval; **RR:** risk ratio; **RCT**: randomized controlled trial

Explanations

a. Some concerns in domain 1 of RoB 2

b. High overall risk of bias in RoB 2

##### **Question:** Anti-regurgitation (AR) formula containing locust bean gum (LBG), prebiotics, and postbiotics vs. Regular formula for GERD

| **Certainty assessment** | | | | | | | **№ of patients** | | **Effect** | | **Certainty** | **Importance** |
| --- | --- | --- | --- | --- | --- | --- | --- | --- | --- | --- | --- | --- |
| **Studies** | **Study design** | **Risk of bias** | **Inconsistency** | **Indirectness** | **Imprecision** | **Other considerations** | **AR formula containing LBG, prebiotics, and postbiotics** | **Regular formula** | **Relative (95% CI)** | **Absolute (95% CI)** |  |  |
| **Vomiting/regurgitation (frequency)** | | | | | | | | | | | | |
| Salvatore 2024 | RCT | not serious | not serious | not serious | not serious | none | n = 48  Vomiting, baseline, mean (SD): 3.9 (0.7); After treatment (week 8): 1.5 (0.6). | n = 52  Vomiting, baseline, mean (SD): 3.6 (0.8); After treatment (week 8): 2.2 (1.0). | p < 0.001 | not reported | ⨁⨁⨁⨁ High | CRITICAL |
| **Infant Gastrointestinal Symptom Questionnaire (IGSQ)** | | | | | | | | | | | | |
| Salvatore 2024 | RCT | not serious | not serious | not serious | not serious | none | n = 51  Overall population, Baseline, mean (SD): 31.8 (8.7);  4 weeks: 23.5 (6.3) | n = 52 | not significant | not reported | ⨁⨁⨁⨁ High | CRITICAL |
| **Total number of reflux events** | | | | | | | | | | | | |
| Not reported | | | | | | | | | | | | |
| **Estimated volume regurgitated** | | | | | | | | | | | | |
| Salvatore 2024 | RCT | serious ^a^ | not serious | not serious | not serious | none | n = 48  Vomiting, baseline, mean (SD): 1.7 (0.7); After treatment (week 8): 1.0 (0.2). | n = 52  Vomiting, baseline, mean (SD): 1.8 (0.8); After treatment (week 8): 1.2 (0.4). | p = 0.088 | not reported | ⨁⨁⨁◯ Moderate | CRITICAL |
| **Respiratory symptoms, nocturnal cough, asthma (frequency)** | | | | | | | | | | | | |
| Not reported | | | | | | | | | | | | |
| **Weight gain** | | | | | | | | | | | | |
| Not reported | | | | | | | | | | | | |
| **Adverse events** | | | | | | | | | | | | |
| Salvatore 2024 | RCT | not serious | not serious | not serious | not serious | none | n = 51  Number of children with adverse events: n = 12 (23.5%) | n = 52  Number of children with adverse events: n = 11 (21.2%) | not significant | not reported | ⨁⨁⨁⨁ High | CRITICAL |

**CI:** confidence interval; **SD:** standard deviation; **RCT**: randomized controlled trial

Explanations

a. Some concerns in domain 3 of RoB 2

##### **Question:** Mg alginate plus simethicone vs. Formula thickened with rice cereals vs. Parental counselling for GERD

| **Certainty assessment** | | | | | | | **№ of patients** | | **Effect** | | **Certainty** | **Importance** |
| --- | --- | --- | --- | --- | --- | --- | --- | --- | --- | --- | --- | --- |
| **Studies** | **Study design** | **Risk of bias** | **Inconsistency** | **Indirectness** | **Imprecision** | **Other considerations** | **Mg alginate plus simethicone** | **Formula thickened with rice cereals / Parental counselling** | **Relative (95% CI)** | **Absolute (95% CI)** |  |  |
| **Vomiting/regurgitation (frequency)** | | | | | | | | | | | | |
| Ummarino 2015 | RCT | serious ^a^ | not serious | not serious | serious ^b^ | none | n = 25  Regurgitation and vomiting, n (%): Baseline: 25 (100);  8 weeks: 6 (25) | Formula: n = 25  Regurgitation and vomiting, n (%): Baseline: 25 (100);  8 weeks: 13 (57)  Counselling: n = 25  Regurgitation and vomiting, n (%): Baseline: 25 (100);  8 weeks: 15 (88) | p < 0.01 | not reported | ⨁⨁◯◯ Low | CRITICAL |
| **Infant Gastro-Esophageal Reflux Questionnaire Revised (I-GERQ-R)** | | | | | | | | | | | | |
| Ummarino 2015 | RCT | serious ^a^ | not serious | not serious | serious ^b^ | none | n = 25  Symptom score, median (range): Baseline: 15 (8–24);  8 weeks: 1 (0–19) | Formula: n = 25  Symptom score, median (range): Baseline: 13 (8–19);  8 weeks: 5 (0–15)  Counselling: n = 25  Symptom score, median (range): Baseline: 13 (7–19);  8 weeks: 8 (2–14) | p = 0.01 | not reported | ⨁⨁◯◯ Low | CRITICAL |
| **Total number of reflux events** | | | | | | | | | | | | |
| Not reported | | | | | | | | | | | | |
| **Estimated volume regurgitated** | | | | | | | | | | | | |
| Not reported | | | | | | | | | | | | |
| **Respiratory symptoms, nocturnal cough, asthma (frequency)** | | | | | | | | | | | | |
| Ummarino 2015 | RCT | serious ^a^ | not serious | not serious | serious ^b^ | none | n = 25  Cough, n (%): Baseline: 7 (28);  8 weeks: 0 (0) | Formula: n = 25  Cough, n (%): Baseline: 6 (24);  8 weeks: 2 (8.7)  Counselling: n = 25  Cough, n (%): Baseline:7 (28);  8 weeks: 2 (11.7) | p = 0.3 | not reported | ⨁⨁◯◯ Low | CRITICAL |
| **Weight gain** | | | | | | | | | | | | |
| Not reported | | | | | | | | | | | | |
| **Adverse events** | | | | | | | | | | | | |
| Ummarino 2015 | RCT | serious ^a^ | not serious | not serious | serious ^b^ | none | n = 25  No adverse events reported, except one child with constipation | Formula: n = 25  No adverse events reported  Counselling: n = 25  No adverse events reported | p < 0.01 | not reported | ⨁⨁◯◯ Low | CRITICAL |

**CI:** confidence interval; **RCT**: randomized controlled trial

Explanations

a. Some concerns in domains 1 and 2 of RoB 2

b. Small sample size

##### **Question:** Thickened formula with locust bean gum (ARF1) vs. Thickened formula with locust bean gum (ARF2) for GERD

| **Certainty assessment** | | | | | | | **№ of patients** | | **Effect** | | **Certainty** | **Importance** |
| --- | --- | --- | --- | --- | --- | --- | --- | --- | --- | --- | --- | --- |
| **Studies** | **Study design** | **Risk of bias** | **Inconsistency** | **Indirectness** | **Imprecision** | **Other considerations** | **Thickened formula with locust bean gum (ARF1)** | **Thickened formula with locust bean gum (ARF2)** | **Relative (95% CI)** | **Absolute (95% CI)** |  |  |
| **Vomiting/regurgitation (frequency)** | | | | | | | | | | | | |
| Vandenplas 2013 | RCT | not serious | not serious | not serious | not serious | none | n = 56  Number of regurgitations, baseline, mean (SD): 8.25 (4.11);  after treatment: 2.32 (2.91) | n = 59  Number of regurgitations, baseline, mean (SD): 8.25 (4.11);  after treatment: 1.89 (2.33) | p (ARF-1, ARF-2) = 0.0091 | not reported | ⨁⨁⨁⨁ High | CRITICAL |
| **Infant Gastro-Esophageal Reflux Questionnaire Revised (I-GERQ-R)** | | | | | | | | | | | | |
| Not reported | | | | | | | | | | | | |
| **Total number of reflux events** | | | | | | | | | | | | |
| Not reported | | | | | | | | | | | | |
| **Estimated volume regurgitated** | | | | | | | | | | | | |
| Vandenplas 2013 | RCT | not serious | not serious | not serious | not serious | none | n = 56  Score of regurgitated volume, baseline, mean (SD): 2.85 (0.76);  after treatment: 1.59 (0.61) | n = 59  Score of regurgitated volume, baseline, mean (SD): 2.85 (0.76);  after treatment: 1.51 (0.56) | p (ARF-1, ARF2) = 0.026 | not reported | ⨁⨁⨁⨁ High | CRITICAL |
| **Respiratory symptoms, nocturnal cough, asthma (frequency)** | | | | | | | | | | | | |
| Not reported | | | | | | | | | | | | |
| **Weight gain** | | | | | | | | | | | | |
| Vandenplas 2013 | RCT | not serious | not serious | not serious | not serious | none | n = 56  Weight, baseline, mean (SD): 5.20 (1.07);  after treatment: 5.97 (0.86) | n = 59  Weight, baseline, mean (SD): 5.18 (0.97);  after treatment: 5.98 (1.01) | not significant | not reported | ⨁⨁⨁⨁ High | CRITICAL |
| **Adverse events** | | | | | | | | | | | | |
| Vandenplas 2013 | RCT | very serious ^a^ | not serious | not serious | not serious | none | n = 56  Low number of adverse events | n = 59  Low number of adverse events | not reported | not reported | ⨁⨁◯◯ Low | CRITICAL |

**CI:** confidence interval; **SD:** standard deviation; **RCT**: randomized controlled trial

Explanations

a. High overall risk of bias in RoB 2

##### **Question:** Formula thickened with rice starch (Enfamil AR®) vs. Bovine milk-based formula for GERD

| **Certainty assessment** | | | | | | | **№ of patients** | | **Effect** | | **Certainty** | **Importance** |
| --- | --- | --- | --- | --- | --- | --- | --- | --- | --- | --- | --- | --- |
| **Studies** | **Study design** | **Risk of bias** | **Inconsistency** | **Indirectness** | **Imprecision** | **Other considerations** | **Formula thickened with rice starch (Enfamil AR®)** | **Bovine milk-based formula** | **Relative (95% CI)** | **Absolute (95% CI)** |  |  |
| **Vomiting/regurgitation (frequency)** | | | | | | | | | | | | |
| Vanderhoof 2003 | RCT | not serious | not serious | not serious | not serious | none | n = 55  Number of regurgitations, baseline, mean (SD): 13 (1);  after treatment, difference: -7 (1) | n = 48  Number of regurgitations, baseline, mean (SD): 11 (1);  after treatment, difference: -5 (1) | not significant | not reported | ⨁⨁⨁⨁ High | CRITICAL |
| **Infant Gastro-Esophageal Reflux Questionnaire Revised (I-GERQ-R)** | | | | | | | | | | | | |
| Not reported | | | | | | | | | | | | |
| **Total number of reflux events** | | | | | | | | | | | | |
| Not reported | | | | | | | | | | | | |
| **Estimated volume regurgitated** | | | | | | | | | | | | |
| Vanderhoof 2003 | RCT | not serious | not serious | not serious | not serious | none | n = 55  Regurgitation volume, baseline, mean (SD): 8.9 (0.5);  after treatment, difference: -4.6 (0.5) | n = 48  Regurgitation volume, baseline, mean (SD): 7.6 (0.5);  after treatment, difference: -3.4 (0.5) | p = 0.05 | not reported | ⨁⨁⨁⨁ High | CRITICAL |
| **Respiratory symptoms, nocturnal cough, asthma (frequency)** | | | | | | | | | | | | |
| Not reported | | | | | | | | | | | | |
| **Weight gain** | | | | | | | | | | | | |
| Not reported | | | | | | | | | | | | |
| **Adverse events** | | | | | | | | | | | | |
| Vanderhoof 2003 | RCT | very serious ^a^ | not serious | not serious | not serious | none | n = 55  One child had 1 SAE | n = 49  Two children had 1 SAE | not significant | not reported | ⨁⨁◯◯ Low | CRITICAL |

**CI:** confidence interval; **SD:** standard deviation; **RCT**: randomized controlled trial; **SAE**: serious adverse event

Explanations

a. High overall risk of bias in RoB 2

##### **Question:** Carob bean thickened formula vs. Regular formula for GERD

| **Certainty assessment** | | | | | | | **№ of patients** | | **Effect** | | **Certainty** | **Importance** |
| --- | --- | --- | --- | --- | --- | --- | --- | --- | --- | --- | --- | --- |
| **Studies** | **Study design** | **Risk of bias** | **Inconsistency** | **Indirectness** | **Imprecision** | **Other considerations** | **Carob bean thickened formula** | **Regular formula** | **Relative (95% CI)** | **Absolute (95% CI)** |  |  |
| **Vomiting/regurgitation (frequency)** | | | | | | | | | | | | |
| Wenzl 2003 | RCT | not serious | not serious | not serious | serious ^a^ | none | n = 7  Regurgitation frequency: 15 | n = 7  Regurgitation frequency: 68 | P < 0.0003 | not estimable | ⨁⨁⨁◯ Moderate | CRITICAL |
| **Infant Gastro-Esophageal Reflux Questionnaire Revised (I-GERQ-R)** | | | | | | | | | | | | |
| Not reported | | | | | | | | | | | | |
| **Total number of reflux events** | | | | | | | | | | | | |
| Wenzl 2003 | RCT | not serious | not serious | not serious | serious ^a^ | none | n = 7  GER episodes: 536 | n = 7  GER episodes: 647 | P < 0.02 | not estimable | ⨁⨁⨁◯ Moderate | CRITICAL |
| **Estimated volume regurgitated** | | | | | | | | | | | | |
| Not reported | | | | | | | | | | | | |
| **Respiratory symptoms, nocturnal cough, asthma (frequency)** | | | | | | | | | | | | |
| Not reported | | | | | | | | | | | | |
| **Weight gain** | | | | | | | | | | | | |
| Not reported | | | | | | | | | | | | |
| **Adverse events** | | | | | | | | | | | | |
| Wenzl 2003 | RCT | serious ^b^ | not serious | not serious | serious ^a^ | none | n = 7  No adverse events | n = 7  No adverse events | not estimable | not estimable | ⨁⨁◯◯ Low | CRITICAL |

**CI:** confidence interval; **RCT**: randomized controlled trial

Explanations

a. Small sample size

b. Some concerns in domain 3 of RoB 2

#### Probiotics

##### **Question:** BB-12 (ABINAT12®) vs. No treatment for GERD

| **Certainty assessment** | | | | | | | **№ of patients** | | **Effect** | | **Certainty** | **Importance** |
| --- | --- | --- | --- | --- | --- | --- | --- | --- | --- | --- | --- | --- |
| **Studies** | **Study design** | **Risk of bias** | **Inconsistency** | **Indirectness** | **Imprecision** | **Other considerations** | **BB-12** | **No treatment** | **Relative (95% CI)** | **Absolute (95% CI)** |  |  |
| **Vomiting/regurgitation (frequency)** | | | | | | | | | | | | |
| Not reported | | | | | | | | | | | | |
| **Infant Gastro-Esophageal Reflux Questionnaire Revised (I-GERQ-R)** | | | | | | | | | | | | |
| Baldassarre 2022 | RCT | very serious ^a^ | not serious | not serious | not serious | none | n = 499  Mean total scores (SD): baseline: 22.3 (5.3); at 30 days: 14.6 (5.2); at 60 days: 11.0 (4.5) | n = 461  Mean total scores (SD): baseline: 23.0 (5.3); at 30 days: 22.3 (7.8); at 60 days: 21.0 (7.6) | p<0.001 | not estimable | ⨁⨁◯◯ Low | CRITICAL |
| **Total number of reflux events** | | | | | | | | | | | | |
| Not reported | | | | | | | | | | | | |
| **Estimated volume regurgitated** | | | | | | | | | | | | |
| Not reported | | | | | | | | | | | | |
| **Respiratory symptoms, nocturnal cough, asthma (frequency)** | | | | | | | | | | | | |
| Not reported | | | | | | | | | | | | |
| **Weight gain** | | | | | | | | | | | | |
| Not reported | | | | | | | | | | | | |
| **Adverse events** | | | | | | | | | | | | |
| Not reported | | | | | | | | | | | | |

**CI:** confidence interval; **RR:** risk ratio; **RCT**: randomized controlled trial

Explanations

a. High overall risk of bias in RoB 2

##### **Question:** *Lactobacillus reuteri* vs. Placebo for GERD

| **Certainty assessment** | | | | | | | **№ of patients** | | **Effect** | | **Certainty** | **Importance** |
| --- | --- | --- | --- | --- | --- | --- | --- | --- | --- | --- | --- | --- |
| **Studies** | **Study design** | **Risk of bias** | **Inconsistency** | **Indirectness** | **Imprecision** | **Other considerations** | ***Lactobacillus reuteri*** | **Placebo** | **Relative (95% CI)** | **Absolute (95% CI)** |  |  |
| **Vomiting/regurgitation (frequency)** | | | | | | | | | | | | |
| Indrio 2011 | RCT | serious ^a^ | not serious | not serious | serious ^b^ | none | n = 19  Median number (5-95 percentiles) of regurgitation episodes per day over the last 7 days of treatment: 1.0 (1.0-2.0) | n = 15  Median number (5-95 percentiles) of regurgitation episodes per day over the last 7 days of treatment: 4.0 (3.0-5.0) | P < 0.001 | not estimable | ⨁⨁◯◯ Low | CRITICAL |
| **Infant Gastro-Esophageal Reflux Questionnaire Revised (I-GERQ-R)** | | | | | | | | | | | | |
| Not reported | | | | | | | | | | | | |
| **Total number of reflux events** | | | | | | | | | | | | |
| Not reported | | | | | | | | | | | | |
| **Estimated volume regurgitated** | | | | | | | | | | | | |
| Not reported | | | | | | | | | | | | |
| **Respiratory symptoms, nocturnal cough, asthma (frequency)** | | | | | | | | | | | | |
| Not reported | | | | | | | | | | | | |
| **Weight gain** | | | | | | | | | | | | |
| Not reported | | | | | | | | | | | | |
| **Adverse events** | | | | | | | | | | | | |
| Indrio 2011 | RCT | serious ^a^ | not serious | not serious | serious ^b^ | none | n = 19  No adverse events reported | n = 15  No adverse events reported | not estimable | not estimable | ⨁⨁◯◯ Low | CRITICAL |

**CI:** confidence interval; **RCT**: randomized controlled trial

Explanations

a. Some concerns in domain 1 of RoB 2

b. Small sample size

#### Alginates vs Placebo

##### **Question:** Alginate (Gaviscon) vs Placebo for GERD

| **Certainty assessment** | | | | | | | **№ of patients** | | **Effect** | | **Certainty** | **Importance** |
| --- | --- | --- | --- | --- | --- | --- | --- | --- | --- | --- | --- | --- |
| **Studies** | **Study design** | **Risk of bias** | **Inconsistency** | **Indirectness** | **Imprecision** | **Other considerations** | **Alginate** | **Placebo** | **Relative (95% CI)** | **Absolute (95% CI)** |  |  |
| **Vomiting/regurgitation (frequency)** | | | | | | | | | | | | |
| Miller 1999 | RCT | serious ^a^ | not serious | not serious | serious ^b^ | none | n = 42  Number of vomiting / regurgitation episodes at baseline, median (range): 8.5 (2-50); end of treatment: 3.0 (0-22) | n = 46  Number of vomiting / regurgitation episodes at baseline, median (range): 7.0 (2-36); end of treatment: 5.0 (0-37) | p = 0.009 | not reported | ⨁⨁◯◯ Low | CRITICAL |
| **Infant Gastro-Esophageal Reflux Questionnaire Revised (I-GERQ-R)** | | | | | | | | | | | | |
| Not reported | | | | | | | | | | | | |
| **Heartburn (frequency)** | | | | | | | | | | | | |
| Not reported | | | | | | | | | | | | |
| **Epigastric pain (frequency)** | | | | | | | | | | | | |
| Not reported | | | | | | | | | | | | |
| **Respiratory symptoms, nocturnal cough, asthma (frequency)** | | | | | | | | | | | | |
| Not reported | | | | | | | | | | | | |
| **Severity of esophagitis** | | | | | | | | | | | | |
| Not reported | | | | | | | | | | | | |
| **Adverse events** | | | | | | | | | | | | |
| Buts 1987 | RCT | very serious ^a^ | not serious | not serious | serious ^b^ | none | n = 10  No adverse events reported. | n = 10  No adverse events reported. | not estimable | not estimable | ⨁◯◯◯ Very low | CRITICAL |
| Miller 1999 | RCT | serious ^a^ | not serious | not serious | serious ^b^ | none | n = 42  Adverse events occurred in 23 patients | n = 48  Adverse events occurred in 28 patients | not significant | not reported | ⨁⨁◯◯ Low | CRITICAL |

**CI:** confidence interval; **RR:** risk ratio; **RCT**: randomized controlled trial

Explanations

a. Some concerns in domains 1 and 5 of RoB 2

b. Small sample size

c. High overall risk of bias in RoB 2

#### Alginates vs Thickened feed

##### **Question:** Magnesium-Alginate vs Thickened Formula for GERD

| **Certainty assessment** | | | | | | | **№ of patients** | | **Effect** | | **Certainty** | **Importance** |
| --- | --- | --- | --- | --- | --- | --- | --- | --- | --- | --- | --- | --- |
| **Studies** | **Study design** | **Risk of bias** | **Inconsistency** | **Indirectness** | **Imprecision** | **Other considerations** | **Magnesium-Alginate** | **Thickened Formula** | **Relative (95% CI)** | **Absolute (95% CI)** |  |  |
| **Vomiting/regurgitation (frequency)** | | | | | | | | | | | | |
| Not reported | | | | | | | | | | | | |
| **Infant Gastro-Esophageal Reflux Questionnaire Revised (I-GERQ-R)** | | | | | | | | | | | | |
| Baldassarre 2019 | RCT | very serious ^a^ | not serious | not serious | serious ^b^ | none | n = 27  I-GERQ-R mean (SD) reduction: -8.96 (6.93) | n = 26  I-GERQ-R mean (SD) reduction: -9.74 (7.66) | p = 0.48 | not reported | ⨁◯◯◯ Very low | CRITICAL |
| **Heartburn (frequency)** | | | | | | | | | | | | |
| Not reported | | | | | | | | | | | | |
| **Epigastric pain (frequency)** | | | | | | | | | | | | |
| Not reported | | | | | | | | | | | | |
| **Respiratory symptoms, nocturnal cough, asthma (frequency)** | | | | | | | | | | | | |
| Not reported | | | | | | | | | | | | |
| **Severity of esophagitis** | | | | | | | | | | | | |
| Not reported | | | | | | | | | | | | |
| **Adverse events** | | | | | | | | | | | | |
| Not reported | | | | | | | | | | | | |

**CI:** confidence interval; **RR:** risk ratio; **RCT**: randomized controlled trial

Explanations

a. High overall risk of bias in RoB 2

b. Small sample size

#### Positioning therapy

##### **Question:** Prone feeding position vs. Lateral left vs. Lateral right for GERD

| **Certainty assessment** | | | | | | | **№ of patients** | | **Effect** | | **Certainty** | **Importance** |
| --- | --- | --- | --- | --- | --- | --- | --- | --- | --- | --- | --- | --- |
| **Studies** | **Study design** | **Risk of bias** | **Inconsistency** | **Indirectness** | **Imprecision** | **Other considerations** | **Prone position** | **Left or right position** | **Relative (95% CI)** | **Absolute (95% CI)** |  |  |
| **Vomiting/regurgitation (frequency)** | | | | | | | | | | | | |
| Not reported | | | | | | | | | | | | |
| **Infant Gastro-Esophageal Reflux Questionnaire Revised (I-GERQ-R)** | | | | | | | | | | | | |
| Not reported | | | | | | | | | | | | |
| **Total number of reflux events** | | | | | | | | | | | | |
| Ewer 1999 | RCT | very serious ^a^ | not serious | not serious | serious ^b^ | none | Prone position: n = 18  Mean number of episodes (SD): 15.4 (2.8) | Left position: n = 18  Right position: n = 18  Mean number of episodes (SD): Left position: 24.6 (3.5); Right position: 41.6 (4.6) | p<0.001 | not estimable | ⨁◯◯◯ Very low | CRITICAL |
| **Estimated volume regurgitated** | | | | | | | | | | | | |
| Not reported | | | | | | | | | | | | |
| **Respiratory symptoms, nocturnal cough, asthma (frequency)** | | | | | | | | | | | | |
| Not reported | | | | | | | | | | | | |
| **Weight gain** | | | | | | | | | | | | |
| Not reported | | | | | | | | | | | | |
| **Adverse events** | | | | | | | | | | | | |
| Not reported | | | | | | | | | | | | |

**CI:** confidence interval; **SD:** standard deviation; **RCT**: randomized controlled trial

Explanations

a. High overall risk of bias in RoB 2

b. Small sample size

##### **Question:** Prone, 30-45° head-elevated positioning vs. Infant seat (semi-upright, 60° angle) for GERD

| **Certainty assessment** | | | | | | | **№ of patients** | | **Effect** | | **Certainty** | **Importance** |
| --- | --- | --- | --- | --- | --- | --- | --- | --- | --- | --- | --- | --- |
| **Studies** | **Study design** | **Risk of bias** | **Inconsistency** | **Indirectness** | **Imprecision** | **Other considerations** | **Prone, 30-45° head-elevated positioning** | **Infant seat** | **Relative (95% CI)** | **Absolute (95% CI)** |  |  |
| **Vomiting/regurgitation (frequency)** | | | | | | | | | | | | |
| Not reported | | | | | | | | | | | | |
| **Infant Gastro-Esophageal Reflux Questionnaire Revised (I-GERQ-R)** | | | | | | | | | | | | |
| Not reported | | | | | | | | | | | | |
| **Total number of reflux events** | | | | | | | | | | | | |
| Orenstein 1983  (J PEDIATR 103:534, 1983) | RCT | very serious ^a^ | not serious | not serious | serious ^b^ | none | n = 15  Mean number (SD) of episodes of pH <4: 19.6 (3.5) | n = 15  Mean number (SD) of episodes of pH <4: 5.2 (1.1) | p < 0.001 | not reported | ⨁◯◯◯ Very low | CRITICAL |
| **Estimated volume regurgitated** | | | | | | | | | | | | |
| Not reported | | | | | | | | | | | | |
| **Respiratory symptoms, nocturnal cough, asthma (frequency)** | | | | | | | | | | | | |
| Not reported | | | | | | | | | | | | |
| **Weight gain** | | | | | | | | | | | | |
| Not reported | | | | | | | | | | | | |
| **Adverse events** | | | | | | | | | | | | |
| Not reported | | | | | | | | | | | | |

**CI:** confidence interval; **SD:** standard deviation; **RCT**: randomized controlled trial

Explanations

a. High overall risk of bias in RoB 2

b. Small sample size

##### **Question:** Infant seat (semi-upright, 60° angle) vs. Flat prone positioning for GERD

| **Certainty assessment** | | | | | | | **№ of patients** | | **Effect** | | **Certainty** | **Importance** |
| --- | --- | --- | --- | --- | --- | --- | --- | --- | --- | --- | --- | --- |
| **Studies** | **Study design** | **Risk of bias** | **Inconsistency** | **Indirectness** | **Imprecision** | **Other considerations** | **Infant seat** | **Flat prone positioning** | **Relative (95% CI)** | **Absolute (95% CI)** |  |  |
| **Vomiting/regurgitation (frequency)** | | | | | | | | | | | | |
| Not reported | | | | | | | | | | | | |
| **Infant Gastro-Esophageal Reflux Questionnaire Revised (I-GERQ-R)** | | | | | | | | | | | | |
| Not reported | | | | | | | | | | | | |
| **Total number of reflux events** | | | | | | | | | | | | |
| Orenstein 1983 (N Engl J Med 1983; 309:760-3.) | RCT | very serious ^a^ | not serious | not serious | serious ^b^ | none | n = 9  Mean number (SD) of episodes of pH <4: 16.0 (2.4) | n = 9  Mean number (SD) of episodes of pH <4: 10.1 (2.3) | p = 0.002 | not reported | ⨁◯◯◯ Very low | CRITICAL |
| **Estimated volume regurgitated** | | | | | | | | | | | | |
| Not reported | | | | | | | | | | | | |
| **Respiratory symptoms, nocturnal cough, asthma (frequency)** | | | | | | | | | | | | |
| Not reported | | | | | | | | | | | | |
| **Weight gain** | | | | | | | | | | | | |
| Not reported | | | | | | | | | | | | |
| **Adverse events** | | | | | | | | | | | | |
| Orenstein 1983 (N Engl J Med 1983; 309:760-3.) | RCT | very serious ^a^ | not serious | not serious | serious ^b^ | none | n = 9  Irritability and greater proportion of time awake was noted in this group | n = 9 | not estimable | not estimable | ⨁◯◯◯ Very low | CRITICAL |

**CI:** confidence interval; **SD:** standard deviation; **RCT**: randomized controlled trial

Explanations

a. High overall risk of bias in RoB 2

b. Small sample size

##### **Question:** Prone feeding position vs. Lateral left vs. Lateral right vs. Supine position (all with 30° elevated head) for GERD

| **Certainty assessment** | | | | | | | **№ of patients** | | **Effect** | | **Certainty** | **Importance** |
| --- | --- | --- | --- | --- | --- | --- | --- | --- | --- | --- | --- | --- |
| **Studies** | **Study design** | **Risk of bias** | **Inconsistency** | **Indirectness** | **Imprecision** | **Other considerations** | **Prone position** | **Supine, left, right position** | **Relative (95% CI)** | **Absolute (95% CI)** |  |  |
| **Vomiting/regurgitation (frequency)** | | | | | | | | | | | | |
| Not reported | | | | | | | | | | | | |
| **Infant Gastro-Esophageal Reflux Questionnaire Revised (I-GERQ-R)** | | | | | | | | | | | | |
| Not reported | | | | | | | | | | | | |
| **Total number of reflux events** | | | | | | | | | | | | |
| Tobin 1997 | RCT | very serious ^a^ | not serious | not serious | serious ^b^ | none | n = 24  Number of episodes/mean time: 4.3 | n = 24  Number of episodes/mean time: supine: 7.1; left: 5.8; right: 5.5 | p < 0.007  LSD: 1.6 prone < supine | not estimable | ⨁◯◯◯ Very low | CRITICAL |
| **Estimated volume regurgitated** | | | | | | | | | | | | |
| Not reported | | | | | | | | | | | | |
| **Respiratory symptoms, nocturnal cough, asthma (frequency)** | | | | | | | | | | | | |
| Not reported | | | | | | | | | | | | |
| **Weight gain** | | | | | | | | | | | | |
| Not reported | | | | | | | | | | | | |
| **Adverse events** | | | | | | | | | | | | |
| Not reported | | | | | | | | | | | | |

**CI:** confidence interval; **LSD:** least significant difference; **RCT**: randomized controlled trial

Explanations

a. High overall risk of bias in RoB 2

b. Small sample size

##### **Question:** Left lateral position (LLP) + antiacids VS Elevated head (HE) + antiacids

| **Certainty assessment** | | | | | | | **№ of patients** | | **Effect** | | **Certainty** | **Importance** |
| --- | --- | --- | --- | --- | --- | --- | --- | --- | --- | --- | --- | --- |
| **Studies** | **Study design** | **Risk of bias** | **Inconsistency** | **Indirectness** | **Imprecision** | **Other considerations** | **Left lateral position (LLP) + antiacids** | **Elevated head (HE) + antiacids** | **Relative (95% CI)** | **Absolute (95% CI)** |  |  |
| **Vomiting/regurgitation (frequency)** | | | | | | | | | | | | |
| Not reported | | | | | | | | | | | | |
| **Infant Gastro-Esophageal Reflux Questionnaire Revised (I-GERQ-R)** | | | | | | | | | | | | |
| Not reported | | | | | | | | | | | | |
| **Total number of reflux events** | | | | | | | | | | | | |
| Loots 2014 | RCT | not serious | not serious | not serious | serious ^a^ | none | n = 13  Total number of episodes (SD): 47 (6) at baseline; 29 (3) after 2 weeks of treatment | n = 12  Total number of episodes (SD): 55 (9) at baseline; 44 (7) after 2 weeks of treatment | p<0.05 from baseline to after treatment in LLP + antiacids group | not estimable | ⨁⨁⨁◯ Moderate | CRITICAL |
| **Estimated volume regurgitated** | | | | | | | | | | | | |
| Not reported | | | | | | | | | | | | |
| **Respiratory symptoms, nocturnal cough, asthma (frequency)** | | | | | | | | | | | | |
| Loots 2014 | RCT | not serious | not serious | not serious | serious ^a^ | none | n = 13  Cough, mean (SD): 30 (12) at baseline; 31 (14) after 2 weeks of treatment | n = 12  Cough, mean (SD): 32 (9) at baseline; 42 (12) after 2 weeks of treatment | p>0.05 | not estimable | ⨁⨁⨁◯ Moderate | CRITICAL |
| **Weight gain** | | | | | | | | | | | | |
| Not reported | | | | | | | | | | | | |
| **Adverse events** | | | | | | | | | | | | |
| Loots 2014 | RCT | not serious | not serious | not serious | serious ^a^ | none | n = 13  No adverse events reported | n = 12  No adverse events reported | not estimable | not estimable | ⨁⨁⨁◯ Moderate | CRITICAL |

**CI:** confidence interval; **SD:** standard deviation; **RCT**: randomized controlled trial

*Explanations*

a. Small sample size

##### **Question:** Anti-regurgitation formula (Novalac AR Digest) VS Thickened formula with bean gum and starch

| **Certainty assessment** | | | | | | | **№ of patients** | | **Effect** | | **Certainty** | **Importance** |
| --- | --- | --- | --- | --- | --- | --- | --- | --- | --- | --- | --- | --- |
| **Studies** | **Study design** | **Risk of bias** | **Inconsistency** | **Indirectness** | **Imprecision** | **Other considerations** | **Anti-regurgitation formula (Novalac AR Digest)** | **Thickened formula with bean gum and starch** | **Relative (95% CI)** | **Absolute (95% CI)** |  |  |
| **Vomiting/regurgitation (frequency)** | | | | | | | | | | | | |
| Not reported | | | | | | | | | | | | |
| **Infant Gastro-Esophageal Reflux Questionnaire Revised (I-GERQ-R)** | | | | | | | | | | | | |
| Not reported | | | | | | | | | | | | |
| **Total number of reflux events** | | | | | | | | | | | | |
| Vandenplas 2008 | RCT | Very serious ^a^ | not serious | not serious | Serious ^b^ | none | n = 6  Number of regurgitations during treatment, mean (SD): 1.8 (1.2) | n = 6  Number of regurgitations during treatment, mean (SD): 5.1 (1.2) | p = 0.002 | not reported | ⨁◯◯◯ Very low | CRITICAL |
| **Estimated volume regurgitated** | | | | | | | | | | | | |
| Not reported | | | | | | | | | | | | |
| **Respiratory symptoms, nocturnal cough, asthma (frequency)** | | | | | | | | | | | | |
| Not reported | | | | | | | | | | | | |
| **Weight gain** | | | | | | | | | | | | |
| Not reported | | | | | | | | | | | | |
| **Adverse events** | | | | | | | | | | | | |
| Not reported | | | | | | | | | | | | |

**CI:** confidence interval; **SD:** standard deviation; **RCT**: randomized controlled trial

#### Explanations

1. High overall risk of bias in RoB 2
2. Small sample size

##### **Question:** Thickened extensive casein hydrolysate (T-eCH) VS Non thickened extensive casein hydrolysate (NT-eCH)

| **Certainty assessment** | | | | | | | **№ of patients** | | **Effect** | | **Certainty** | **Importance** |
| --- | --- | --- | --- | --- | --- | --- | --- | --- | --- | --- | --- | --- |
| **Studies** | **Study design** | **Risk of bias** | **Inconsistency** | **Indirectness** | **Imprecision** | **Other considerations** | **Thickened extensive casein hydrolysate**  **(T-eCH)** | **Non thickened extensive casein hydrolysate**  **(NT-eCH)** | **Relative (95% CI)** | **Absolute (95% CI)** |  |  |
| **Vomiting/regurgitation (frequency)** | | | | | | | | | | | | |
| Not reported | | | | | | | | | | | | |
| **Infant Gastro-Esophageal Reflux Questionnaire Revised (I-GERQ-R)** | | | | | | | | | | | | |
| Not reported | | | | | | | | | | | | |
| **Total number of reflux events** | | | | | | | | | | | | |
| Vandenplas 2014 | RCT | not serious | not serious | Serious ^a^ | Serious ^b^ | none | n = 35  Number of daily regurgitations, mean (SD), at baseline: 6.6 (2.1); after 30 days of treatment: 2.4 (2.3) | n = 37  Number of daily regurgitations, mean (SD), at baseline: 6.2 (4.0); after 30 days of treatment: 3.3 (3.4) | p = 0.24 | not reported | ⨁⨁◯◯ Low | CRITICAL |
| **Estimated volume regurgitated** | | | | | | | | | | | | |
| Not reported | | | | | | | | | | | | |
| **Respiratory symptoms, nocturnal cough, asthma (frequency)** | | | | | | | | | | | | |
| Not reported | | | | | | | | | | | | |
| **Weight gain** | | | | | | | | | | | | |
| Vandenplas 2014 | RCT | not serious | not serious | Serious ^a^ | Serious ^b^ | none | n = 35  Weight gain, mean (SD): 1.0 (0.4) | n = 37  Weight gain, mean (SD): 0.9 (0.5) | p = 0.96 | not reported | ⨁⨁◯◯ Low | CRITICAL |
| **Adverse events** | | | | | | | | | | | | |
| Not reported | | | | | | | | | | | | |

**CI:** confidence interval; **SD:** standard deviation; **RCT**: randomized controlled trial

#### Explanations

1. Population not selected for GERD but suspected cow’s milk protein allergy (CMPA)
2. Small sample size

#### Dietary modifications + Probiotics

##### **Question:** Partially hydrolysed 100% whey formula (NAN A.R.) thickened with starch + *Lactobacillus reuteri* vs. Regular starter formula (NAN 1) for GERD

| **Certainty assessment** | | | | | | | **№ of patients** | | **Effect** | | **Certainty** | **Importance** |
| --- | --- | --- | --- | --- | --- | --- | --- | --- | --- | --- | --- | --- |
| **Studies** | **Study design** | **Risk of bias** | **Inconsistency** | **Indirectness** | **Imprecision** | **Other considerations** | **Test Formula** | **Control Formula** | **Relative (95% CI)** | **Absolute (95% CI)** |  |  |
| **Vomiting/regurgitation (frequency)** | | | | | | | | | | | | |
| Indrio 2017 | RCT | serious ^a^ | not serious | not serious | serious ^b^ | none | n = 37  Mean daily number of regurgitations (SD): baseline: 7.4 (0.8); 4 weeks: 2.6 (1.0) | n = 35  Mean daily number of regurgitations (SD): baseline: 7.5 (1.0); 4 weeks: 5.3 (1.0) | p < 0.001 | not estimable | ⨁⨁◯◯ Low | CRITICAL |
| **Infant Gastro-Esophageal Reflux Questionnaire Revised (I-GERQ-R)** | | | | | | | | | | | | |
| Not reported | | | | | | | | | | | | |
| **Total number of reflux events** | | | | | | | | | | | | |
| Not reported | | | | | | | | | | | | |
| **Estimated volume regurgitated** | | | | | | | | | | | | |
| Not reported | | | | | | | | | | | | |
| **Respiratory symptoms, nocturnal cough, asthma (frequency)** | | | | | | | | | | | | |
| Not reported | | | | | | | | | | | | |
| **Weight gain** | | | | | | | | | | | | |
| Indrio 2017 | RCT | serious ^a^ | not serious | not serious | serious ^b^ | none | n = 37  Mean body weight (SD): baseline: 5.59 Kg (0.63); 4 weeks: 6.28 Kg (0.39) | n = 35  Mean body weight (SD): baseline: 5.67 Kg (0.74); 4 weeks: 6.32 Kg (0.24) | not significant | not estimable | ⨁⨁◯◯ Low | CRITICAL |
| **Adverse events** | | | | | | | | | | | | |
| Indrio 2017 | RCT | serious ^a^ | not serious | not serious | serious ^b^ | none | n = 37  No adverse events reported | n = 35  No adverse events reported | not estimable | not estimable | ⨁⨁◯◯ Low | CRITICAL |

**CI:** confidence interval; **SD:** standard deviation; **RCT**: randomized controlled trial

Explanations

a. Some concerns in domain 1 of RoB 2

b. Small sample size

#### Massage therapy

##### **Question:** Massage therapy vs. Sham non-massage therapy for GERD

| **Certainty assessment** | | | | | | | **№ of patients** | | **Effect** | | **Certainty** | **Importance** |
| --- | --- | --- | --- | --- | --- | --- | --- | --- | --- | --- | --- | --- |
| **Studies** | **Study design** | **Risk of bias** | **Inconsistency** | **Indirectness** | **Imprecision** | **Other considerations** | **Massage** | **Non-massage** | **Relative (95% CI)** | **Absolute (95% CI)** |  |  |
| **Vomiting/regurgitation (frequency)** | | | | | | | | | | | | |
| Not reported | | | | | | | | | | | | |
| **Infant Gastro-Esophageal Reflux Questionnaire Revised (I-GERQ-R)** | | | | | | | | | | | | |
| Neu 2014 | RCT | not serious | not serious | not serious | serious ^a^ | none | n = 18  Mean scores (SD): baseline: 22.0 (4); 4 weeks: 15.0 (4); 6 weeks: 14.4 (5) | n = 18  Mean scores (SD): baseline: 23.5 (4); 4 weeks: 15.1 (5); 6 weeks: 13.7 (6) | not significant | not estimable | ⨁⨁⨁◯ Moderate | CRITICAL |
| **Total number of reflux events** | | | | | | | | | | | | |
| Not reported | | | | | | | | | | | | |
| **Estimated volume regurgitated** | | | | | | | | | | | | |
| Not reported | | | | | | | | | | | | |
| **Respiratory symptoms, nocturnal cough, asthma (frequency)** | | | | | | | | | | | | |
| Not reported | | | | | | | | | | | | |
| **Weight gain** | | | | | | | | | | | | |
| Neu 2014 | RCT | very serious ^b^ | not serious | not serious | serious ^a^ | none | n = 18  Mean weight for both groups (SD): baseline: 4.8 Kg (0.6); 4 weeks: 5.7 Kg (0.7); 6 weeks: 6.0 Kg (0.7) | n = 18 | not significant | not estimable | ⨁◯◯◯ Very low | CRITICAL |
| **Adverse events** | | | | | | | | | | | | |
| Not reported | | | | | | | | | | | | |

**CI:** confidence interval; **SD:** standard deviation; **RCT**: randomized controlled trial

Explanations

a. Small sample size

b. High overall risk of bias in RoB 2

##### **Question:** Abdominal massage with mastic gum oil VS abdominal massage without mastic gum oil

| **Certainty assessment** | | | | | | | **№ of patients** | | **Effect** | | **Certainty** | **Importance** |
| --- | --- | --- | --- | --- | --- | --- | --- | --- | --- | --- | --- | --- |
| **Studies** | **Study design** | **Risk of bias** | **Inconsistency** | **Indirectness** | **Imprecision** | **Other considerations** | **Massage with mastic gum oil** | **Massage without mastic gum oil** | **Relative (95% CI)** | **Absolute (95% CI)** |  |  |
| **Vomiting/regurgitation (frequency)** | | | | | | | | | | | | |
| Kenari 2020 | RCT | very  serious ^a^ | not serious | not serious | serious ^b^ | serious ^c^ | n = 45  Mean scores (SD): baseline: 10.82 (11.24); 2 weeks: 4.27 (7.22); 4 weeks follow-up: 6.13 (7.63) | n = 45  Mean scores (SD): baseline: 9.31 (11.80); 2 weeks: 6.52 (8.89); 4 weeks follow-up: 6.91 (10.56) | not significant | not estimable | ⨁◯◯◯ Very low | CRITICAL |
|  | | | | | | | | | | | | |
| **Infant Gastro-Esophageal Reflux Questionnaire Revised (I-GERQ-R)** | | | | | | | | | | | | |
| Not reported | | | | | | | | | | | | |
| **Total number of reflux events** | | | | | | | | | | | | |
| Not reported | | | | | | | | | | | | |
| **Estimated volume regurgitated** | | | | | | | | | | | | |
| Not reported | | | | | | | | | | | | |
| **Respiratory symptoms, nocturnal cough, asthma (frequency)** | | | | | | | | | | | | |
| Not reported | | | | | | | | | | | | |
| **Weight gain** | | | | | | | | | | | | |
| Not reported | | | | | | | | | | | | |
| **Adverse events** | | | | | | | | | | | | |
| Not reported | | | | | | | | | | | | |

**CI:** confidence interval; **SD:** standard deviation; **RCT**: randomized controlled trial

#### Explanations

1. High overall risk of bias in RoB 2
2. Small sample size
3. All children were also treated with omeprazole that could have affected results interpretation

### PICO 7 - What is the indication and the effectiveness of different surgical/endoscopic treatment options for GERD?

#### Laparoscopic Nissen fundoplication vs Open Nissen Funduplication

##### **Question:** Laparoscopic Nissan fundoplication compared to Open Nissan fundoplication for GERD

| **Certainty assessment** | | | | | | | **№ of patients** | | **Effect** | | **Certainty** | **Importance** |
| --- | --- | --- | --- | --- | --- | --- | --- | --- | --- | --- | --- | --- |
| **Studies** | **Study design** | **Risk of bias** | **Inconsistency** | **Indirectness** | **Imprecision** | **Other considerations** | **LN** | **ON** | **Relative (95% CI)** | **Absolute (95% CI)** |  |  |
| **Mortality short-term** | | | | | | | | | | | | |
| Knatten 2012  Papandria 2014 | randomised trials | not serious | not serious | not serious | not serious | none | 0/67 (0.0%) | 0/65 (0.0%) | not estimable | not estimable | ⨁⨁⨁⨁ High | CRITICAL |
| **Mortality after 4 years** | | | | | | | | | | | | |
| Fyhn 2015  Papandria 2014 | randomised trials | not serious | not serious | not serious | serious^a^ | none | 7/65 (10.8%) | 7/65 (10.8%) | **RR 1.00** (0.37 to 2.68) | **0 fewer per 1.000** (from 68 fewer to 181 more) | ⨁⨁⨁◯ Moderate^a^ | CRITICAL |
| **GERD recurrence after 4 years** | | | | | | | | | | | | |
| Fyhn 2015  Pacili 2014  Papandria 2014 | randomised trials | serious^b^ | not serious | not serious | serious^a^ | none | 20/90 (22.2%) | 6/86 (7.0%) | **RR 2.70** (0.93 to 7.88) | **119 more per 1.000** (from 5 fewer to 480 more) | ⨁⨁◯◯ Low^a,b^ | CRITICAL |
| **Dysphagia after 2-4 years** | | | | | | | | | | | | |
| Fyhn 2015  McHoney 2011 | randomised trials | serious^b^ | not serious | not serious | very serious^c^ | none | 2/67 (3.0%) | 0/65 (0.0%) | **RR 2.87** (0.31 to 26.84) | **0 fewer per 1.000** (from 0 fewer to 0 fewer) | ⨁◯◯◯ Very low^b,c^ | CRITICAL |
| **Anti-secretory drug after 4 years** | | | | | | | | | | | | |
| Papandria 2014 | randomised trials | not serious | not applicable | not serious | serious^a^ | none | 13/23 (56.5%) | 15/21 (71.4%) | **RR 0.79** (0.51 to 1.24) | **150 fewer per 1.000** (from 350 fewer to 171 more) | ⨁⨁⨁◯ Moderate^a^ | CRITICAL |
| **Retching after 4 years** | | | | | | | | | | | | |
| Fyhn 2015  Pacili 2014 | randomised trials | not serious | serious^d^ | not serious | very serious^c^ | none | 5/67 (7.5%) | 13/65 (20.0%) | **RR 0.36** (0.05 to 2.33) | **128 fewer per 1.000** (from 190 fewer to 266 more) | ⨁◯◯◯ Very low^c,d^ | CRITICAL |
| **Mortality after 12 years** | | | | | | | | | | | | |
| Fyhn 2023 | randomised trials | not serious | not applicable | not serious | serious^a^ | none | 7/44 (15.9%) | 6/44 (13.6%) | **RR 1.17** (0.43 to 3.19) | **23 more per 1.000** (from 78 fewer to 299 more) | ⨁⨁⨁◯ Moderate^a^ | CRITICAL |
| **GERD recurrence after 12 years** | | | | | | | | | | | | |
| Fyhn 2023 | randomised trials | not serious | not applicable | not serious | not serious | none | 24/44 (54.5%) | 13/44 (29.5%) | **RR 1.85** (1.09 to 3.14) | **251 more per 1.000** (from 27 more to 632 more) | ⨁⨁⨁⨁ High | CRITICAL |
| **Dysphagia after 12 years** | | | | | | | | | | | | |
| Fyhn 2023 | randomised trials | not serious | not applicable | not serious | very serious^a^ | none | 1/44 (2.3%) | 3/44 (6.8%) | **RR 0.33** (0.04 to 3.08) | **46 fewer per 1.000** (from 65 fewer to 142 more) | ⨁⨁◯◯ Low^a^ | CRITICAL |
| **Anti-secretory drug use after 12 years** | | | | | | | | | | | | |
| Fyhn 2023 | randomised trials | not serious | not applicable | not serious | serious^a^ | none | 9/44 (20.5%) | 6/44 (13.6%) | **RR 1.50** (0.58 to 3.86) | **68 more per 1.000** (from 57 fewer to 390 more) | ⨁⨁⨁◯ Moderate^a^ | CRITICAL |
| **Retching after 12 years** | | | | | | | | | | | | |
| Fyhn 2023 | randomised trials | not serious | not applicable | not serious | serious^a^ | none | 5/44 (11.4%) | 5/44 (11.4%) | **RR 1.00** (0.31 to 3.21) | **0 fewer per 1.000** (from 78 fewer to 251 more) | ⨁⨁⨁◯ Moderate^a^ | CRITICAL |

**CI:** confidence interval; **RR:** risk ratio;

Explanations

a. Confidence interval crossing the line of no effect

b. Some concerns regarding deviation from intended interventions domain

c. Wide confidence interval crossing the line of no effect

d. I^2 = 62%

#### Laparoscopic Nissen fundoplication vs Thal Nissen Funduplication

##### **Question:** Laparoscopic Nissan fundoplication compared to Thal fundoplication for GERD

| **Certainty assessment** | | | | | | | **№ of patients** | | **Effect** | | **Certainty** | **Importance** |
| --- | --- | --- | --- | --- | --- | --- | --- | --- | --- | --- | --- | --- |
| **№ of studies** | **Study design** | **Risk of bias** | **Inconsistency** | **Indirectness** | **Imprecision** | **Other considerations** | **LN** | **Thal** | **Relative (95% CI)** | **Absolute (95% CI)** |  |  |
| **Mortality short term** | | | | | | | | | | | | |
| Kubiak 2010 | randomised trials | serious^a^ | not applicable | not serious | very serious^b^ | none | 1/89 (1.1%) | 1/86 (1.2%) | **RR 0.97** (0.06 to 15.21) | **0 fewer per 1.000** (from 11 fewer to 165 more) | ⨁◯◯◯ Very low^a,b^ | CRITICAL |
| **Dysphagia short term** | | | | | | | | | | | | |
| Kubiak 2010 | randomised trials | serious^a^ | not applicable | not serious | serious^c^ | none | 12/89 (13.5%) | 10/86 (11.6%) | **RR 1.16** (0.53 to 2.54) | **19 more per 1.000** (from 55 fewer to 179 more) | ⨁⨁◯◯ Low^a,c^ | CRITICAL |
| **Mortality after 9 years** | | | | | | | | | | | | |
| Kubiak 2011 | randomised trials | serious^a^ | not applicable | not serious | serious^c^ | none | 21/89 (23.6%) | 10/86 (11.6%) | **RR 2.03** (1.02 to 4.05) | **120 more per 1.000** (from 2 more to 355 more) | ⨁⨁◯◯ Low^a,c^ | CRITICAL |
| **Dyshpagia - long term*** | | | | | | | | | | | | |
| Kubiak 2011 | randomised trials | very serious^a,d^ | not applicable | not serious | serious^c^ | none | 20/89 (22.5%) | 18/86 (20.9%) | **RR 1.07** (0.61 to 1.89) | **15 more per 1.000** (from 82 fewer to 186 more) | ⨁◯◯◯ Very low^c,d^ | CRITICAL |
| **Anti-secretory drug use – long term *** | | | | | | | | | | | | |
| Kubiak 2011 | randomised trials | very serious^a,d^ | not applicable | not serious | serious^c^ | none | 11/89 (12.4%) | 8/86 (9.3%) | **RR 1.33** (0.56 to 3.14) | **31 more per 1.000** (from 41 fewer to 199 more) | ⨁◯◯◯ Very low^c,d^ | CRITICAL |
| **Mortality after 20 years** | | | | | | | | | | | | |
| Skerritt 2022 | randomised trials | serious^a^ | not applicable | not serious | not serious^c^ | none | 37/89 (41.6%) | 22/86 (25.6%) | **RR 1.63** (1.05 to 2.51) | **161 more per 1.000** (from 13 more to 386 more) | ⨁⨁⨁◯ Moderate^a,c^ | CRITICAL |
| **Dysphagia after 11 years** | | | | | | | | | | | | |
| Skerritt 2022 | randomised trials | serious^d^ | not applicable | not serious | very serious^b^ | none | 3/89 (3.4%) | 2/86 (2.3%) | **RR 1.45** (0.25 to 8.46) | **10 more per 1.000** (from 17 fewer to 173 more) | ⨁◯◯◯ Very low^b,d^ | CRITICAL |
| **Anti-secretory drug use after 11 years** | | | | | | | | | | | | |
| Skerritt 2022 | randomised trials | serious^d^ | not applicable | not serious | serious^c^ | none | 7/89 (7.9%) | 12/86 (14.0%) | **RR 0.56** (0.23 to 1.36) | **61 fewer per 1.000** (from 107 fewer to 50 more) | ⨁⨁◯◯ Low^c,d^ | CRITICAL |

**CI:** confidence interval; **RR:** risk ratio;

Explanations

a. Some concerns regarding the randomization process domain

b. Wide confidence interval crossing line of no effect

c. Confidence interval crossing line of no effect

d. High risk of bias in the domain: selection of the reported results

*. Unclear timing of outcome assessment

#### Laparoscopic Nissen fundoplication vs Hill – Snow Tecnique

##### **Question:** Laparoscopic Nissan fundoplication compared to Hill-Snow procedure for GERD

| **Certainty assessment** | | | | | | | **№ of patients** | | **Effect** | | **Certainty** | **Importance** |
| --- | --- | --- | --- | --- | --- | --- | --- | --- | --- | --- | --- | --- |
| **№ of studies** | **Study design** | **Risk of bias** | **Inconsistency** | **Indirectness** | **Imprecision** | **Other considerations** | **LN** | **Hill-Snow** | **Relative (95% CI)** | **Absolute (95% CI)** |  |  |
| **Dysphagia - early postoperative** | | | | | | | | | | | | |
| Gad 2022 | randomised trials | not serious | not applicable | not serious | very serious^a^ | none | 7/20 (35.0%) | 2/20 (10.0%) | **RR 3.50** (0.83 to 14.83) | **250 more per 1.000** (from 17 fewer to 1.000 more) | ⨁⨁◯◯ Low^a^ | CRITICAL |
| **Gas bloat syndrome - early postoperative** | | | | | | | | | | | | |
| Gad 2022 | randomised trials | not serious | not applicable | not serious | serious^b^ | none | 15/20 (75.0%) | 3/20 (15.0%) | **RR 5.00** (1.71 to 14.63) | **600 more per 1.000** (from 107 more to 1.000 more) | ⨁⨁⨁◯ Moderate^b^ | CRITICAL |
| **GERD recurrence - late postoperative** | | | | | | | | | | | | |
| Gad 2022 | randomised trials | not serious | not applicable | not serious | very serious^a^ | none | 2/20 (10.0%) | 3/20 (15.0%) | **RR 0.67** (0.12 to 3.57) | **49 fewer per 1.000** (from 132 fewer to 385 more) | ⨁⨁◯◯ Low^a^ | CRITICAL |
| **Dysphagia - late postoperative** | | | | | | | | | | | | |
| Gad 2022 | randomised trials | not serious | not applicable | not serious | very serious^a^ | none | 0/20 (0.0%) | 0/20 (0.0%) | not estimable | not estimable | ⨁⨁◯◯ Low^a^ | CRITICAL |
| **Anti-secretory drug use - late postoperative** | | | | | | | | | | | | |
| Gad 2022 | randomised trials | not serious | not applicable | not serious | serious^c^ | none | 5/20 (25.0%) | 8/20 (40.0%) | **RR 0.63** (0.25 to 1.58) | **148 fewer per 1.000** (from 300 fewer to 232 more) | ⨁⨁⨁◯ Moderate^c^ | CRITICAL |
| **Gas bloat syndrome - late postoperative** | | | | | | | | | | | | |
| Gad 2022 | randomised trials | not serious | not applicable | not serious | serious^b^ | none | 11/20 (55.0%) | 0/20 (0.0%) | **RR 23.00** (1.45 to 365.61) | **0 fewer per 1.000** (from 0 fewer to 0 fewer) | ⨁⨁⨁◯ Moderate^b^ | CRITICAL |
| **Dumping syndrome - late postoperative** | | | | | | | | | | | | |
| Gad 2022 | randomised trials | not serious | not applicable | not serious | very serious^a^ | none | 5/20 (25.0%) | 1/20 (5.0%) | **RR 5.00** (0.64 to 39.06) | **200 more per 1.000** (from 18 fewer to 1.000 more) | ⨁⨁◯◯ Low^a^ | CRITICAL |

**CI:** confidence interval; **RR:** risk ratio.

Explanations

a. Wide confidence interval crossing line of no effect

b. Wide confidence interval

c. Confidence interval crossing line of no effect
