# Supplementary material for: Guidelines on diagnosis and management of gastroesophageal reflux disease in infants, children and adolescents: a joint consensus from Italian pediatric societies (SIP and SIGENP) -Part II: management
Source: Ital J Pediatr. 2026 Apr 10;52:90. doi: 10.1186/s13052-026-02255-0 (PMC13182049; doi:10.1186/s13052-026-02255-0)
Supplement: Supplementary file 5 — Additional File 5 [file 13052_2026_2255_MOESM5_ESM.docx]

# Additional File 5. GRADE Evidence-to-Decision tables

## PICO 5. What is the evidence for the effectiveness of pharmacological treatments for GER and GERD in infants, children, and adolescents?

| Question | |
| --- | --- |
| **Should Proton Pump Inhibitors (PPI) vs. Placebo be used for children with GER/GERD?** | |
| **Population:** | Children with GER/GERD |
| **Intervention:** | PPI |
| **Comparison:** | Placebo |
| **Main outcomes:** | - Vomiting/regurgitation (frequency) - Infant Gastro-Esophageal Reflux Questionnaire Revised (I-GERQ-R) - Heartburn (frequency) - Epigastric pain (frequency) - Respiratory symptoms, nocturnal cough, asthma (frequency) - Severity of esophagitis - Adverse events |
| **Setting:** | Outpatient and inpatient |
| **Perspective:** | Clinical recommendation |
| **Background:** | Pediatricians using PPIs to reduce signs and symptoms of GERD in children need to know whether using PPIs leads to clinical benefits and/or side effects compared with placebo. |
| **Conflict of interests:** | SIP (Italian Society of Pediatrics) conflict of interest declaration and management policies were applied and the following panel members were voting panel members (determining the direction and strength of the recommendation): All.  Panel members recused as a result of risk of conflicts of interest: None. |

# Assessment

| Problem Is the problem a priority? | | |
| --- | --- | --- |
| Judgement | Research evidence | Additional considerations |
| ○ No ○ Probably no ○ Probably yes **X** **Yes** ○ Varies ○ Don't know | The use of PPIs to reduce crying/distress, visible vomiting/regurgitation, or signs and symptoms of GERD in pediatric patients with GERD is a common practice. Knowledge of whether using PPIs leads to clinical benefits and/or side effects compared with placebo could reduce patient inconvenience and improve clinical outcomes. |  |
| Desirable Effects How substantial are the desirable anticipated effects? | | |
| Judgement | Research evidence | Additional considerations |
| ○ Trivial **X** **Small** ○ Moderate ○ Large ○ Varies ○ Don't know | For research evidence on Desirable and Undesirable anticipated effects, as well as the certainty of this evidence, see the Evidence Profile. |  |
| Undesirable Effects How substantial are the undesirable anticipated effects? | | |
| Judgement | Research evidence | Additional considerations |
| ○ Trivial **X** **Small** ○ Moderate ○ Large ○ Varies ○ Don't know | For research evidence on Desirable and Undesirable anticipated effects, as well as the certainty of this evidence, see the Evidence Profile. |  |
| Certainty of evidence What is the overall certainty of the evidence of effects? | | |
| Judgement | Research evidence | Additional considerations |
| ○ Very low **X** **Low** ○ Moderate ○ High ○ No included studies | For research evidence on Desirable and Undesirable anticipated effects, as well as the certainty of this evidence, see the Evidence Profile. |  |
| Values Is there important uncertainty about or variability in how much people value the main outcomes? | | |
| Judgement | Research evidence | Additional considerations |
| ○ Important uncertainty or variability ○ Possibly important uncertainty or variability **X** **Probably no important uncertainty or variability** ○ No important uncertainty or variability | No research evidence identified. |  |
| Balance of effects Does the balance between desirable and undesirable effects favor the intervention or the comparison? | | |
| Judgement | Research evidence | Additional considerations |
| ○ Favors the comparison ○ Probably favors the comparison ○ Does not favor either the intervention or the comparison ○ Probably favors the intervention ○ Favors the intervention **X Varies** ○ Don't know | In infants and younger children (1–12 months), PPIs are not significantly more effective than placebo in reducing symptoms such as regurgitation and irritability. Potential side effects are frequent and may outweigh the benefits. In children older than 12 months and adolescents, PPIs can be effective, particularly in cases of erosive esophagitis; side effects are generally less problematic but still require monitoring. |  |
| Resources required | | |
| Judgement | Research evidence | Additional considerations |
| ○ Large costs **X Moderate costs** ○ Negligible costs and savings ○ Moderate savings ○ Large savings ○ Varies ○ Don't know | No research evidence identified. |  |
| Certainty of evidence of required resources What is the certainty of the evidence of resource requirements (costs)? | | |
| Judgement | Research evidence | Additional considerations |
| ○ Very low ○ Low ○ Moderate ○ High **X No included studies** | No research evidence identified. |  |
| Cost effectiveness Does the cost-effectiveness of the intervention favor the intervention or the comparison? | | |
| Judgement | Research evidence | Additional considerations |
| ○ Favors the comparison ○ Probably favors the comparison ○ Does not favor either the intervention or the comparison ○ Probably favors the intervention ○ Favors the intervention ○ Varies **X No included studies** | No research evidence identified. |  |
| Equity What would be the impact on health equity? | | |
| Judgement | Research evidence | Additional considerations |
| ○ Reduced ○ Probably reduced **X Probably no impact** ○ Probably increased ○ Increased ○ Varies ○ Don't know | No research evidence identified. |  |
| Acceptability Is the intervention acceptable to key stakeholders? | | |
| Judgement | Research evidence | Additional considerations |
| ○ No ○ Probably no **X Probably yes** ○ Yes ○ Varies ○ Don't know | No research evidence identified. |  |
| Feasibility Is the intervention feasible to implement? | | |
| Judgement | Research evidence | Additional considerations |
| ○ No ○ Probably no ○ Probably yes **X Yes** ○ Varies ○ Don't know | No research evidence identified. |  |

# Summary of judgements

|  | **Judgement** | | | | | | |
| --- | --- | --- | --- | --- | --- | --- | --- |
| **Problem** | No | Probably no | Probably yes | **Yes** |  | Varies | Don't know |
| **Desirable Effects** | Trivial | **Small** | Moderate | Large |  | Varies | Don't know |
| **Undesirable Effects** | Trivial | **Small** | Moderate | Large |  | Varies | Don't know |
| **Certainty of evidence** | Very low | **Low** | Moderate | High |  |  | No included studies |
| **Values** | Important uncertainty or variability | Possibly important uncertainty or variability | **Probably no important uncertainty or variability** | No important uncertainty or variability |  |  |  |
| **Balance of effects** | Favors the comparison | Probably favors the comparison | Does not favor either the intervention or the comparison | Probably favors the intervention | Favors the intervention | **Varies** | Don't know |
| **Resources required** | Large costs | **Moderate costs** | Negligible costs and savings | Moderate savings | Large savings | Varies | Don't know |
| **Certainty of evidence of required resources** | Very low | Low | Moderate | High |  |  | **No included studies** |
| **Cost effectiveness** | Favors the comparison | Probably favors the comparison | Does not favor either the intervention or the comparison | Probably favors the intervention | Favors the intervention | Varies | **No included studies** |
| **Equity** | Reduced | Probably reduced | **Probably no impact** | Probably increased | Increased | Varies | Don't know |
| **Acceptability** | No | Probably no | **Probably yes** | Yes |  | Varies | Don't know |
| **Feasibility** | No | Probably no | Probably yes | **Yes** |  | Varies | Don't know |

# Type of recommendation

# infants and younger children (1–12 months)

| Strong recommendation against the intervention | Conditional recommendation against the intervention | Conditional recommendation for either the intervention or the comparison | Conditional recommendation for the intervention | Strong recommendation for the intervention |
| --- | --- | --- | --- | --- |
| ○ | X | ○ | ○ | ○ |

# children older than 12 months and adolescents

| Strong recommendation against the intervention | Conditional recommendation against the intervention | Conditional recommendation for either the intervention or the comparison | Conditional recommendation for the intervention | Strong recommendation for the intervention |
| --- | --- | --- | --- | --- |
| ○ | ○ | ○ | X | ○ |

# Conclusions

| Recommendation |
| --- |
| We recommend not to use proton pump inhibitors (PPIs) or H2RA to treat overt crying, distress, or visible regurgitation in otherwise healthy infants based on the I-GERQ-R questionnaire, since these symptoms are common, transient, and do not necessarily indicate pathological GERD.  We recommend a 4-8 week trial of a PPI or H2RA for children and adolescents with typical symptoms of GERD (persistent heartburn, retrosternal or epigastric pain).  We recommend the use of PPIs as first-line treatment of reflux-related erosive esophagitis in infants and children, based on endoscopic confirmation of the disease.  We recommend not to use H2RAs or PPIs in patients with extraesophageal symptoms (e.g., cough, wheezing, asthma), except in the presence of typical GERD symptoms and/or diagnostic testing suggestive of GERD. |
| Justification |
| In infants and younger children (1–12 months), PPIs are not significantly more effective than placebo in reducing symptoms such as regurgitation and irritability. Potential side effects are frequent and may outweigh the benefits. In children older than 12 months and adolescents, PPIs can be effective, particularly in cases of erosive esophagitis; side effects are generally less problematic but still require monitoring. |

| Subgroup considerations |
| --- |
|  |
| Implementation considerations |
|  |

| Monitoring and evaluation |
| --- |
|  |
| Research priorities |
|  |
| REFERENCES SUMMARY See the main text of PICO 5 and Additional File 2. |

| Question | |
| --- | --- |
| **Should Proton Pump Inhibitors (PPI) vs H2-Antagonists be used for children with GER/GERD?** | |
| **Population:** | Children with GER/GERD |
| **Intervention:** | PPI |
| **Comparison:** | H2-Antagonists |
| **Main outcomes:** | - Vomiting/regurgitation (frequency) - Infant Gastro-Esophageal Reflux Questionnaire Revised (I-GERQ-R) - Heartburn (frequency) - Epigastric pain (frequency) - Respiratory symptoms, nocturnal cough, asthma (frequency) - Severity of esophagitis - Adverse events |
| **Setting:** | Outpatient and inpatient |
| **Perspective:** | Clinical recommendation |
| **Background:** | Pediatricians using PPIs to reduce signs and symptoms of GERD in children need to know whether using PPIs leads to clinical benefits and/or side effects compared with H2-Antagonists. |
| **Conflict of interests:** | SIP (Italian Society of Pediatrics) conflict of interest declaration and management policies were applied and the following panel members were voting panel members (determining the direction and strength of the recommendation): All.  Panel members recused as a result of risk of conflicts of interest: None. |

# Assessment

| Problem Is the problem a priority? | | |
| --- | --- | --- |
| Judgement | Research evidence | Additional considerations |
| ○ No ○ Probably no ○ Probably yes **X** **Yes** ○ Varies ○ Don't know | The use of PPIs and H2-Antagonists to reduce crying/distress, visible vomiting/regurgitation, or signs and symptoms of GERD in pediatric patients with GERD is a common practice. Knowledge of whether using PPIs leads to clinical benefits and/or side effects compared with H2-Antagonists could reduce patient inconvenience and improve clinical outcomes. |  |
| Desirable Effects How substantial are the desirable anticipated effects? | | |
| Judgement | Research evidence | Additional considerations |
| ○ Trivial **X** **Small** ○ Moderate ○ Large ○ Varies ○ Don't know | For research evidence on Desirable and Undesirable anticipated effects, as well as the certainty of this evidence, see the Evidence Profile. |  |
| Undesirable Effects How substantial are the undesirable anticipated effects? | | |
| Judgement | Research evidence | Additional considerations |
| ○ Trivial **X** **Small** ○ Moderate ○ Large ○ Varies ○ Don't know | For research evidence on Desirable and Undesirable anticipated effects, as well as the certainty of this evidence, see the Evidence Profile. |  |
| Certainty of evidence What is the overall certainty of the evidence of effects? | | |
| Judgement | Research evidence | Additional considerations |
| ○ Very low **X** **Low** ○ Moderate ○ High ○ No included studies | For research evidence on Desirable and Undesirable anticipated effects, as well as the certainty of this evidence, see the Evidence Profile. |  |
| Values Is there important uncertainty about or variability in how much people value the main outcomes? | | |
| Judgement | Research evidence | Additional considerations |
| ○ Important uncertainty or variability ○ Possibly important uncertainty or variability **X** **Probably no important uncertainty or variability** ○ No important uncertainty or variability | No research evidence identified. |  |
| Balance of effects Does the balance between desirable and undesirable effects favor the intervention or the comparison? | | |
| Judgement | Research evidence | Additional considerations |
| ○ Favors the comparison ○ Probably favors the comparison **○** Does not favor either the intervention or the comparison **○** Probably favors the intervention ○ Favors the intervention **X** **Varies** ○ Don't know | In infants and young children, there is no solid evidence favoring one class of acid-suppressive agents over the other. Accordingly, treatment decisions should be guided by ease of administration, cost, and availability, with H2RAs serving as a second-line option for acid reflux esophagitis when PPIs are unavailable. |  |
| Resources required | | |
| Judgement | Research evidence | Additional considerations |
| ○ Large costs **X** **Moderate costs** ○ Negligible costs and savings ○ Moderate savings ○ Large savings ○ Varies ○ Don't know | No research evidence identified. |  |
| Certainty of evidence of required resources What is the certainty of the evidence of resource requirements (costs)? | | |
| Judgement | Research evidence | Additional considerations |
| ○ Very low ○ Low ○ Moderate ○ High **X** **No included studies** | No research evidence identified. |  |
| Cost effectiveness Does the cost-effectiveness of the intervention favor the intervention or the comparison? | | |
| Judgement | Research evidence | Additional considerations |
| ○ Favors the comparison ○ Probably favors the comparison ○ Does not favor either the intervention or the comparison ○ Probably favors the intervention ○ Favors the intervention ○ Varies **X** **No included studies** | No research evidence identified. |  |
| Equity What would be the impact on health equity? | | |
| Judgement | Research evidence | Additional considerations |
| ○ Reduced ○ Probably reduced **X** **Probably no impact** ○ Probably increased ○ Increased ○ Varies ○ Don't know | No research evidence identified. |  |
| Acceptability Is the intervention acceptable to key stakeholders? | | |
| Judgement | Research evidence | Additional considerations |
| ○ No ○ Probably no **X** **Probably yes** ○ Yes ○ Varies ○ Don't know | No research evidence identified. |  |
| Feasibility Is the intervention feasible to implement? | | |
| Judgement | Research evidence | Additional considerations |
| ○ No ○ Probably no ○ Probably yes **X** **Yes** ○ Varies ○ Don't know | No research evidence identified. |  |

# Summary of judgements

|  | **Judgement** | | | | | | |
| --- | --- | --- | --- | --- | --- | --- | --- |
| **Problem** | No | Probably no | Probably yes | **Yes** |  | Varies | Don't know |
| **Desirable Effects** | Trivial | **Small** | Moderate | Large |  | Varies | Don't know |
| **Undesirable Effects** | Trivial | **Small** | Moderate | Large |  | Varies | Don't know |
| **Certainty of evidence** | Very low | **Low** | Moderate | High |  |  | No included studies |
| **Values** | Important uncertainty or variability | Possibly important uncertainty or variability | **Probably no important uncertainty or variability** | No important uncertainty or variability |  |  |  |
| **Balance of effects** | Favors the comparison | Probably favors the comparison | Does not favor either the intervention or the comparison | Probably favors the intervention | Favors the intervention | **Varies** | Don't know |
| **Resources required** | Large costs | **Moderate costs** | Negligible costs and savings | Moderate savings | Large savings | Varies | Don't know |
| **Certainty of evidence of required resources** | Very low | Low | Moderate | High |  |  | **No included studies** |
| **Cost effectiveness** | Favors the comparison | Probably favors the comparison | Does not favor either the intervention or the comparison | Probably favors the intervention | Favors the intervention | Varies | **No included studies** |
| **Equity** | Reduced | Probably reduced | **Probably no impact** | Probably increased | Increased | Varies | Don't know |
| **Acceptability** | No | Probably no | **Probably yes** | Yes |  | Varies | Don't know |
| **Feasibility** | No | Probably no | Probably yes | **Yes** |  | Varies | Don't know |

# Type of recommendation

| Strong recommendation against the intervention | Conditional recommendation against the intervention | Conditional recommendation for either the intervention or the comparison | Conditional recommendation for the intervention | Strong recommendation for the intervention |
| --- | --- | --- | --- | --- |
| ○ | ○ | ○ | **X** | ○ |

# Conclusions

| Recommendation |
| --- |
| We recommend not to use proton pump inhibitors (PPIs) or H2RA to treat overt crying/distress or visible regurgitation or signs and symptoms of GERD based on the I-GERQ-R questionnaire in otherwise healthy infants.  We recommend a 4-8 week trial of a PPI or H2RA for children and adolescents with typical symptoms (persistent heartburn, retrosternal or epigastric pain).  We recommend the use of PPIs as first-line treatment of reflux-related endoscopy-proven erosive esophagitis in infants and children.  We recommend the use of H2RAs in the treatment of reflux-related erosive esophagitis in infants and children if PPIs are not available or contra-indicated.  We recommend not to use H2RAs or PPIs in patients with extraesophageal symptoms (ie, cough, wheezing, asthma), except in the presence of typical GERD symptoms and/or diagnostic testing suggestive of GERD. |
| Justification |
| In infants and young children, there is no solid evidence favoring one class of acid-suppressive agents over the other, nor any proof that specific drugs within these classes confer unique advantages. Accordingly, treatment decisions should be guided by ease of administration, cost, and availability, with H2RAs serving as a second-line option for acid reflux esophagitis when PPIs are unavailable. |

| Subgroup considerations |
| --- |
|  |
| Implementation considerations |
|  |

| Monitoring and evaluation |
| --- |
|  |
| Research priorities |
|  |
| REFERENCES SUMMARY See the main text of PICO 5 and Additional File 2. |

| Question | |
| --- | --- |
| **Should H2-antagonists vs Placebo be used for children with GER/GERD** | |
| **Population:** | Children with GER/GERD |
| **Intervention:** | H2RAs |
| **Comparison:** | Placebo |
| **Main outcomes:** | - Vomiting/regurgitation (frequency) - Infant Gastro-Esophageal Reflux Questionnaire Revised (I-GERQ-R) - Heartburn (frequency) - Epigastric pain (frequency) - Respiratory symptoms, nocturnal cough, asthma (frequency) - Severity of esophagitis - Adverse events |
| **Setting:** | Outpatient and inpatient |
| **Perspective:** | Clinical recommendation |
| **Background:** | Identify a second-line therapy when PPI treatment is not available or not recommended. |
| **Conflict of interests:** | SIP (Italian Society of Pediatrics) conflict of interest declaration and management policies were applied and the following panel members were voting panel members (determining the direction and strength of the recommendation): All.  Panel members recused as a result of risk of conflicts of interest: None. |

# Assessment

| Problem Is the problem a priority? | | |
| --- | --- | --- |
| Judgement | Research evidence | Additional considerations |
| ○ No ○ Probably no ○ Probably yes **X** **Yes** ○ Varies ○ Don't know | The use of H2RAs as an alternative to PPIs when these are unavailable or not recommended in the treatment of erosive esophagitis due to GER/GERD. Knowledge of whether using H2RAs leads to clinical benefits and/or side effects compared with placebo could reduce patient inconvenience and improve clinical outcomes. |  |
| Desirable Effects How substantial are the desirable anticipated effects? | | |
| Judgement | Research evidence | Additional considerations |
| ○ Trivial **X** **Small** ○ Moderate ○ Large ○ Varies ○ Don't know | For research evidence on Desirable and Undesirable anticipated effects, as well as the certainty of this evidence, see the Evidence Profile. |  |
| Undesirable Effects How substantial are the undesirable anticipated effects? | | |
| Judgement | Research evidence | Additional considerations |
| ○ Trivial **X** **Small** ○ Moderate ○ Large ○ Varies ○ Don't know | For research evidence on Desirable and Undesirable anticipated effects, as well as the certainty of this evidence, see the Evidence Profile. |  |
| Certainty of evidence What is the overall certainty of the evidence of effects? | | |
| Judgement | Research evidence | Additional considerations |
| **x Very low** ○ Low ○ Moderate ○ High ○ No included studies | For research evidence on Desirable and Undesirable anticipated effects, as well as the certainty of this evidence, see the Evidence Profile. |  |
| Values Is there important uncertainty about or variability in how much people value the main outcomes? | | |
| Judgement | Research evidence | Additional considerations |
| ○ Important uncertainty or variability ○ Possibly important uncertainty or variability **X Probably no important uncertainty or variability** ○ No important uncertainty or variability | No research evidence identified. |  |
| Balance of effects Does the balance between desirable and undesirable effects favor the intervention or the comparison? | | |
| Judgement | Research evidence | Additional considerations |
| ○ Favors the comparison ○ Probably favors the comparison ○ Does not favor either the intervention or the comparison **X Probably favors the intervention** ○ Favors the intervention ○ Varies ○ Don't know | The use of H2RAs has shown an improvement in symptoms and endoscopic outcomes in patients with erosive esophagitis, although without a significant impact on crying, discomfort, or heartburn compared to placebo. |  |
| Resources required | | |
| Judgement | Research evidence | Additional considerations |
| ○ Large costs **X Moderate costs** ○ Negligible costs and savings ○ Moderate savings ○ Large savings ○ Varies ○ Don't know | No research evidence identified. |  |
| Certainty of evidence of required resources What is the certainty of the evidence of resource requirements (costs)? | | |
| Judgement | Research evidence | Additional considerations |
| ○ Very low ○ Low ○ Moderate ○ High **X No included studies** | No research evidence identified. |  |
| Cost effectiveness Does the cost-effectiveness of the intervention favor the intervention or the comparison? | | |
| Judgement | Research evidence | Additional considerations |
| ○ Favors the comparison ○ Probably favors the comparison ○ Does not favor either the intervention or the comparison ○ Probably favors the intervention ○ Favors the intervention ○ Varies **X No included studies** | No research evidence identified. |  |
| Equity What would be the impact on health equity? | | |
| Judgement | Research evidence | Additional considerations |
| ○ Reduced ○ Probably reduced **X Probably no impact** ○ Probably increased ○ Increased ○ Varies ○ Don't know | No research evidence identified. |  |
| Acceptability Is the intervention acceptable to key stakeholders? | | |
| Judgement | Research evidence | Additional considerations |
| ○ No ○ Probably no **X Probably yes** ○ Yes ○ Varies ○ Don't know | No research evidence identified. |  |
| Feasibility Is the intervention feasible to implement? | | |
| Judgement | Research evidence | Additional considerations |
| ○ No ○ Probably no ○ Probably yes **X Yes** ○ Varies ○ Don't know | No research evidence identified. |  |

# Summary of judgements

|  | **Judgement** | | | | | | |
| --- | --- | --- | --- | --- | --- | --- | --- |
| **Problem** | No | Probably no | Probably yes | **Yes** |  | Varies | Don't know |
| **Desirable Effects** | Trivial | **Small** | Moderate | Large |  | Varies | Don't know |
| **Undesirable Effects** | Trivial | **Small** | Moderate | Large |  | Varies | Don't know |
| **Certainty of evidence** | **Very low** | **Low** | Moderate | High |  |  | No included studies |
| **Values** | Important uncertainty or variability | Possibly important uncertainty or variability | **Probably no important uncertainty or variability** | No important uncertainty or variability |  |  |  |
| **Balance of effects** | Favors the comparison | Probably favors the comparison | **Does not favor either the intervention or the comparison** | **Probably favors the intervention** | Favors the intervention | Varies | Don't know |
| **Resources required** | Large costs | **Moderate costs** | Negligible costs and savings | Moderate savings | Large savings | Varies | Don't know |
| **Certainty of evidence of required resources** | Very low | Low | Moderate | High |  |  | **No included studies** |
| **Cost effectiveness** | Favors the comparison | Probably favors the comparison | Does not favor either the intervention or the comparison | Probably favors the intervention | Favors the intervention | Varies | **No included studies** |
| **Equity** | Reduced | Probably reduced | **Probably no impact** | Probably increased | Increased | Varies | Don't know |
| **Acceptability** | No | Probably no | **Probably yes** | Yes |  | Varies | Don't know |
| **Feasibility** | No | Probably no | Probably yes | **Yes** |  | Varies | Don't know |

# Type of recommendation

# infants and younger children (1–12 months)

| Strong recommendation against the intervention | Conditional recommendation against the intervention | Conditional recommendation for either the intervention or the comparison | Conditional recommendation for the intervention | Strong recommendation for the intervention |
| --- | --- | --- | --- | --- |
| ○ | ○ | ○ | X | ○ |

# children older than 12 months and adolescents

| Strong recommendation against the intervention | Conditional recommendation against the intervention | Conditional recommendation for either the intervention or the comparison | Conditional recommendation for the intervention | Strong recommendation for the intervention |
| --- | --- | --- | --- | --- |
| ○ | ○ | ○ | X | ○ |

# Conclusions

| Recommendation |
| --- |
| We recommend employing H2RAs as an alternative for treating reflux- related erosive esophagitis in infants and children when PPIs are either unavailable or contraindicated.  We recommend a 4-8 week trial of a PPI or H2RA for children and adolescents with typical symptoms of GERD (persistent heartburn, retrosternal or epigastric pain).  We recommend not to use H2RAs or PPIs in patients with extraesophageal symptoms (e.g., cough, wheezing, asthma), except in the presence of typical GERD symptoms and/or diagnostic testing suggestive of GERD. |
| Justification |
| H2RAs can be effective, particularly in cases of erosive esophagitis but they are not significantly more effective than placebo in reducing symptoms such as regurgitation and irritability. Otherwise, side effects are generally less problematic but still require monitoring. |

| Subgroup considerations |
| --- |
|  |
| Implementation considerations |
|  |

| Monitoring and evaluation |
| --- |
|  |
| Research priorities |
|  |
| REFERENCES SUMMARY See the main text of PICO 5 and Additional File 2. |

| Question | |
| --- | --- |
| **Should H2RAs vs. Sucralfate be used for children with GER/GERD?** | |
| **Population:** | Children with GER/GERD |
| **Intervention:** | H2RAs |
| **Comparison:** | Sucralfate |
| **Main outcomes:** | - Vomiting/regurgitation (frequency) - Infant Gastro-Esophageal Reflux Questionnaire Revised (I-GERQ-R) - Heartburn (frequency) - Epigastric pain (frequency) - Respiratory symptoms, nocturnal cough, asthma (frequency) - Severity of esophagitis - Adverse events |
| **Setting:** | Outpatient and inpatient |
| **Perspective:** | Clinical recommendation |
| **Background:** | Pediatricians using PPIs to reduce signs and symptoms of GERD in children need to know whether using PPIs leads to clinical benefits and/or side effects compared with placebo. |
| **Conflict of interests:** | SIP (Italian Society of Pediatrics) conflict of interest declaration and management policies were applied and the following panel members were voting panel members (determining the direction and strength of the recommendation): All.  Panel members recused as a result of risk of conflicts of interest: None. |

# Assessment

| Problem Is the problem a priority? | | |
| --- | --- | --- |
| Judgement | Research evidence | Additional considerations |
| ○ No x **Probably no** ○ Probably yes ○ Yes ○ Varies ○ Don't know | The use of H2RAs has shown improvement in symptoms and endoscopic outcomes in patients with erosive esophagitis, and serve as a second‑line treatment for acid reflux esophagitis when PPIs are unavailable or not recommended. H2RAs were compared with sucralfate, no significant differences in endoscopic healing were found. |  |
| Desirable Effects How substantial are the desirable anticipated effects? | | |
| Judgement | Research evidence | Additional considerations |
| ○ Trivial **X** **Small** ○ Moderate ○ Large ○ Varies ○ Don't know | For research evidence on Desirable and Undesirable anticipated effects, as well as the certainty of this evidence, see the Evidence Profile. |  |
| Undesirable Effects How substantial are the undesirable anticipated effects? | | |
| Judgement | Research evidence | Additional considerations |
| ○ Trivial **X** **Small** ○ Moderate ○ Large ○ Varies ○ Don't know | For research evidence on Desirable and Undesirable anticipated effects, as well as the certainty of this evidence, see the Evidence Profile. |  |
| Certainty of evidence What is the overall certainty of the evidence of effects? | | |
| Judgement | Research evidence | Additional considerations |
| x**Very low** ○ Low ○ Moderate ○ High ○ No included studies | For research evidence on Desirable and Undesirable anticipated effects, as well as the certainty of this evidence, see the Evidence Profile. |  |
| Values Is there important uncertainty about or variability in how much people value the main outcomes? | | |
| Judgement | Research evidence | Additional considerations |
| ○ Important uncertainty or variability ○ Possibly important uncertainty or variability **X** **Probably no important** uncertainty or variability ○ No important uncertainty or variability | No research evidence identified. |  |
| Balance of effects Does the balance between desirable and undesirable effects favor the intervention or the comparison? | | |
| Judgement | Research evidence | Additional considerations |
| ○ Favors the comparison ○ Probably favors the comparison **X Does not favor either the intervention or the comparison** ○ Probably favors the intervention ○ Favors the intervention ○ Varies ○ Don't know | It remains unclear whether H2 receptor antagonists (H2RAs) improve endoscopic findings or lead to more adverse effects compared to sucralfate. |  |
| Resources required | | |
| Judgement | Research evidence | Additional considerations |
| ○ Large costs **X Moderate costs** ○ Negligible costs and savings ○ Moderate savings ○ Large savings ○ Varies ○ Don't know | No research evidence identified. |  |
| Certainty of evidence of required resources What is the certainty of the evidence of resource requirements (costs)? | | |
| Judgement | Research evidence | Additional considerations |
| ○ Very low ○ Low ○ Moderate ○ High **X No included studies** | No research evidence identified. |  |
| Cost effectiveness Does the cost-effectiveness of the intervention favor the intervention or the comparison? | | |
| Judgement | Research evidence | Additional considerations |
| ○ Favors the comparison ○ Probably favors the comparison ○ Does not favor either the intervention or the comparison ○ Probably favors the intervention ○ Favors the intervention ○ Varies **X No included studies** | No research evidence identified. |  |
| Equity What would be the impact on health equity? | | |
| Judgement | Research evidence | Additional considerations |
| ○ Reduced ○ Probably reduced **X Probably no impact** ○ Probably increased ○ Increased ○ Varies ○ Don't know | No research evidence identified. |  |
| Acceptability Is the intervention acceptable to key stakeholders? | | |
| Judgement | Research evidence | Additional considerations |
| ○ No ○ Probably no **X Probably yes** ○ Yes ○ Varies ○ Don't know | No research evidence identified. |  |
| Feasibility Is the intervention feasible to implement? | | |
| Judgement | Research evidence | Additional considerations |
| ○ No ○ Probably no ○ Probably yes **X Yes** ○ Varies ○ Don't know | No research evidence identified. |  |

# Summary of judgements

|  | **Judgement** | | | | | | |
| --- | --- | --- | --- | --- | --- | --- | --- |
| **Problem** | No | **Probably no** | Probably yes | **Yes** |  | Varies | Don't know |
| **Desirable Effects** | Trivial | **Small** | Moderate | Large |  | Varies | Don't know |
| **Undesirable Effects** | Trivial | **Small** | Moderate | Large |  | Varies | Don't know |
| **Certainty of evidence** | **Very low** | **Low** | Moderate | High |  |  | No included studies |
| **Values** | Important uncertainty or variability | Possibly important uncertainty or variability | **Probably no important uncertainty or variability** | No important uncertainty or variability |  |  |  |
| **Balance of effects** | Favors the comparison | Probably favors the comparison | **Does not favor either the intervention or the comparison** | Probably favors the intervention | Favors the intervention | Varies | Don't know |
| **Resources required** | Large costs | **Moderate costs** | Negligible costs and savings | Moderate savings | Large savings | Varies | Don't know |
| **Certainty of evidence of required resources** | Very low | Low | Moderate | High |  |  | **No included studies** |
| **Cost effectiveness** | Favors the comparison | Probably favors the comparison | Does not favor either the intervention or the comparison | Probably favors the intervention | Favors the intervention | Varies | **No included studies** |
| **Equity** | Reduced | Probably reduced | **Probably no impact** | Probably increased | Increased | Varies | Don't know |
| **Acceptability** | No | Probably no | **Probably yes** | Yes |  | Varies | Don't know |
| **Feasibility** | No | Probably no | Probably yes | **Yes** |  | Varies | Don't know |

# Type of recommendation

# infants and younger children (1–12 months)

| Strong recommendation against the intervention | Conditional recommendation against the intervention | Conditional recommendation for either the intervention or the comparison | Conditional recommendation for the intervention | Strong recommendation for the intervention |
| --- | --- | --- | --- | --- |
| ○ | ○ | X | ○ | ○ |

# children older than 12 months and adolescents

| Strong recommendation against the intervention | Conditional recommendation against the intervention | Conditional recommendation for either the intervention or the comparison | Conditional recommendation for the intervention | Strong recommendation for the intervention |
| --- | --- | --- | --- | --- |
| ○ | ○ | X | ○ | ○ |

# Conclusions

| Recommendation |
| --- |
|  |
| Justification |
|  |

| Subgroup considerations |
| --- |
|  |
| Implementation considerations |
|  |

| Monitoring and evaluation |
| --- |
|  |
| Research priorities |
|  |
| REFERENCES SUMMARY See the main text of PICO 5 and Additional File 2. |

| Question | |
| --- | --- |
| **Should H2RAs vs. Sucralfate be used for children with GER/GERD?** | |
| **Population:** | Children with GER/GERD |
| **Intervention:** | Prokinetics agents |
| **Comparison:** | Placebo |
| **Main outcomes:** | - Vomiting/regurgitation (frequency) - Infant Gastro-Esophageal Reflux Questionnaire Revised (I-GERQ-R) - Heartburn (frequency) - Epigastric pain (frequency) - Respiratory symptoms, nocturnal cough, asthma (frequency) - Severity of esophagitis - Adverse events |
| **Setting:** | Outpatient and inpatient |
| **Perspective:** | Clinical recommendation |
| **Background:** | Prokinetics are not indicated as a primary treatment for gastroesophageal reflux but may be considered in specific circumstances (such as confirmed gastric motility dysfunction) or in patients with GERD that is refractory to conventional therapies. |
| **Conflict of interests:** | SIP (Italian Society of Pediatrics) conflict of interest declaration and management policies were applied and the following panel members were voting panel members (determining the direction and strength of the recommendation): All.  Panel members recused as a result of risk of conflicts of interest: None. |

# Assessment

| Problem Is the problem a priority? | | |
| --- | --- | --- |
| Judgement | Research evidence | Additional considerations |
| ○ No **x Probably no** ○ Probably yes ○ Yes ○ Varies ○ Don't know | The use of prokinetics remains controversial. Currently, they are not recommended as a first-line treatment for gastroesophageal reflux in pediatric patients. |  |
| Desirable Effects How substantial are the desirable anticipated effects? | | |
| Judgement | Research evidence | Additional considerations |
| ○ Trivial **X** **Small** ○ Moderate ○ Large ○ Varies ○ Don't know | For research evidence on Desirable and Undesirable anticipated effects, as well as the certainty of this evidence, see the Evidence Profile. |  |
| Undesirable Effects How substantial are the undesirable anticipated effects? | | |
| Judgement | Research evidence | Additional considerations |
| ○ Trivial **X** **Small** ○ Moderate ○ Large ○ Varies ○ Don't know | For research evidence on Desirable and Undesirable anticipated effects, as well as the certainty of this evidence, see the Evidence Profile. |  |
| Certainty of evidence What is the overall certainty of the evidence of effects? | | |
| Judgement | Research evidence | Additional considerations |
| **xVery low** ○ Low ○ Moderate ○ High ○ No included studies | For research evidence on Desirable and Undesirable anticipated effects, as well as the certainty of this evidence, see the Evidence Profile. |  |
| Values Is there important uncertainty about or variability in how much people value the main outcomes? | | |
| Judgement | Research evidence | Additional considerations |
| ○ Important uncertainty or variability ○ Possibly important uncertainty or variability **X Probably no important uncertainty or variability** ○ No important uncertainty or variability | No research evidence identified. |  |
| Balance of effects Does the balance between desirable and undesirable effects favor the intervention or the comparison? | | |
| Judgement | Research evidence | Additional considerations |
| ○ Favors the comparison ○ Probably favors the comparison **X Does not favor either the intervention or the comparison** ○ Probably favors the intervention ○ Favors the intervention ○ Varies ○ Don't know | There is no clear evidence that prokinetic agents are more effective in reducing GER/GERD symptoms in infants and children, nor that they lead to more side effects. |  |
| Resources required | | |
| Judgement | Research evidence | Additional considerations |
| ○ Large costs **X Moderate costs** ○ Negligible costs and savings ○ Moderate savings ○ Large savings ○ Varies ○ Don't know | No research evidence identified. |  |
| Certainty of evidence of required resources What is the certainty of the evidence of resource requirements (costs)? | | |
| Judgement | Research evidence | Additional considerations |
| ○ Very low ○ Low ○ Moderate ○ High **X No included studies** | No research evidence identified. |  |
| Cost effectiveness Does the cost-effectiveness of the intervention favor the intervention or the comparison? | | |
| Judgement | Research evidence | Additional considerations |
| ○ Favors the comparison ○ Probably favors the comparison ○ Does not favor either the intervention or the comparison ○ Probably favors the intervention ○ Favors the intervention ○ Varies **X No included studies** | No research evidence identified. |  |
| Equity What would be the impact on health equity? | | |
| Judgement | Research evidence | Additional considerations |
| ○ Reduced ○ Probably reduced **X Probably no impact** ○ Probably increased ○ Increased ○ Varies ○ Don't know | No research evidence identified. |  |
| Acceptability Is the intervention acceptable to key stakeholders? | | |
| Judgement | Research evidence | Additional considerations |
| ○ No **X Probably no** ○ Probably yes ○ Yes ○ Varies ○ Don't know | No research evidence identified. |  |
| Feasibility Is the intervention feasible to implement? | | |
| Judgement | Research evidence | Additional considerations |
| ○ No **x Probably no** ○ Probably yes ○ Yes ○ Varies ○ Don't know | No research evidence identified. |  |

# Summary of judgements

|  | **Judgement** | | | | | | |
| --- | --- | --- | --- | --- | --- | --- | --- |
| **Problem** | No | **Probably no** | Probably yes | **Yes** |  | Varies | Don't know |
| **Desirable Effects** | Trivial | **Small** | Moderate | Large |  | Varies | Don't know |
| **Undesirable Effects** | Trivial | **Small** | Moderate | Large |  | Varies | Don't know |
| **Certainty of evidence** | **Very low** | **Low** | Moderate | High |  |  | No included studies |
| **Values** | Important uncertainty or variability | Possibly important uncertainty or variability | **Probably no important uncertainty or variability** | No important uncertainty or variability |  |  |  |
| **Balance of effects** | Favors the comparison | Probably favors the comparison | **Does not favor either the intervention or the comparison** | Probably favors the intervention | Favors the intervention | Varies | Don't know |
| **Resources required** | Large costs | **Moderate costs** | Negligible costs and savings | Moderate savings | Large savings | Varies | Don't know |
| **Certainty of evidence of required resources** | Very low | Low | Moderate | High |  |  | **No included studies** |
| **Cost effectiveness** | Favors the comparison | Probably favors the comparison | Does not favor either the intervention or the comparison | Probably favors the intervention | Favors the intervention | Varies | **No included studies** |
| **Equity** | Reduced | Probably reduced | **Probably no impact** | Probably increased | Increased | Varies | Don't know |
| **Acceptability** | No | **Probably no** | Probably yes | Yes |  | Varies | Don't know |
| **Feasibility** | No | **Probably no** | Probably yes | Yes |  | Varies | Don't know |

# Type of recommendation

# infants and younger children (1–12 months)

| Strong recommendation against the intervention | Conditional recommendation against the intervention | Conditional recommendation for either the intervention or the comparison | Conditional recommendation for the intervention | Strong recommendation for the intervention |
| --- | --- | --- | --- | --- |
| X | ○ | ○ | ○ | ○ |

# children older than 12 months and adolescents

| Strong recommendation against the intervention | Conditional recommendation against the intervention | Conditional recommendation for either the intervention or the comparison | Conditional recommendation for the intervention | Strong recommendation for the intervention |
| --- | --- | --- | --- | --- |
| X | ○ | ○ | ○ | ○ |

# Conclusions

| Recommendation |
| --- |
| We recommend against the use of metoclopramide, domperidone, erythromycin, or bethanechol in the treatment of GERD in infants and children because of their limited evidence of efficacy. |
| Justification |
| There is no clear evidence that prokinetic agents are more effective in reducing GER/GERD symptoms in infants and children, nor that they lead to more side effects. None of these studies reported adverse events, although the overall evidence quality is very low. |

| Subgroup considerations |
| --- |
|  |
| Implementation considerations |
|  |

| Monitoring and evaluation |
| --- |
|  |
| Research priorities |
|  |
| REFERENCES SUMMARY See the main text of PICO 5 and Additional File 2. |

| Question | |
| --- | --- |
| **Should Bethanechol vs Placebo feed be used for children with GER/GERD?** | |
| **Population:** | Children with GER/GERD |
| **Intervention:** | Bethanechol |
| **Comparison:** | Placebo |
| **Main outcomes:** | - Vomiting/regurgitation (frequency) - Infant Gastro-Esophageal Reflux Questionnaire Revised (I-GERQ-R) - Heartburn (frequency) - Epigastric pain (frequency) - Respiratory symptoms, nocturnal cough, asthma (frequency) - Severity of esophagitis - Adverse events |
| **Setting:** | **I**Inpatient |
| **Perspective:** | Clinical recommendation |
| **Background:** | Bethanechol is not FDA-approved for pediatric use, has uncertain efficacy, and carries a high risk of adverse events |
| **Conflict of interests:** | SIP (Italian Society of Pediatrics) conflict of interest declaration and management policies were applied and the following panel members were voting panel members (determining the direction and strength of the recommendation): All.  Panel members recused as a result of risk of conflicts of interest: None. |

# Assessment

| Problem Is the problem a priority? | | |
| --- | --- | --- |
| Judgement | Research evidence | Additional considerations |
| **X** **No** ○ Probably no ○ Probably yes ○ Yes ○ Varies ○ Don't know | The use of bethanechol to reduce signs and symptoms of GERD in pediatric patients with GERD is uncommon and not advised by international guidelines and not approved by FDA. |  |
| Desirable Effects How substantial are the desirable anticipated effects? | | |
| Judgement | Research evidence | Additional considerations |
| **X** **Trivial** ○ Small ○ Moderate ○ Large ○ Varies ○ Don't know | For research evidence on Desirable and Undesirable anticipated effects, as well as the certainty of this evidence, see the Evidence Profile. |  |
| Undesirable Effects How substantial are the undesirable anticipated effects? | | |
| Judgement | Research evidence | Additional considerations |
| ○ Trivial ○ Small ○ Moderate **X** **Large** ○ Varies ○ Don't know | For research evidence on Desirable and Undesirable anticipated effects, as well as the certainty of this evidence, see the Evidence Profile. |  |
| Certainty of evidence What is the overall certainty of the evidence of effects? | | |
| Judgement | Research evidence | Additional considerations |
| ○ Very low **X** **Low** ○ Moderate ○ High ○ No included studies | For research evidence on Certainty of evidence anticipated effects, as well as the certainty of this evidence, see the Evidence Profile. |  |
| Values Is there important uncertainty about or variability in how much people value the main outcomes? | | |
| Judgement | Research evidence | Additional considerations |
| ○ Important uncertainty or variability ○ Possibly important uncertainty or variability ○ Probably no important uncertainty or variability **X** **No important uncertainty or variability** | No research evidence identified. |  |
| Balance of effects Does the balance between desirable and undesirable effects favor the intervention or the comparison? | | |
| Judgement | Research evidence | Additional considerations |
| **X** **Favors the comparison** ○ Probably favors the comparison ○ Does not favor either the intervention or the comparison ○ Probably favors the intervention ○ Favors the intervention ○ Varies ○ Don't know | There is insufficient evidence to recommend bethanechol for GERD treatment in infants and children. |  |
| Resources required | | |
| Judgement | Research evidence | Additional considerations |
| ○ Large costs ○ Moderate costs **X** **Negligible costs and savings** ○ Moderate savings ○ Large savings ○ Varies ○ Don't know | No research evidence identified. |  |
| Certainty of evidence of required resources What is the certainty of the evidence of resource requirements (costs)? | | |
| Judgement | Research evidence | Additional considerations |
| ○ Very low **X** **Low** ○ Moderate ○ High ○ No included studies | No research evidence identified. |  |
| Cost effectiveness Does the cost-effectiveness of the intervention favor the intervention or the comparison? | | |
| Judgement | Research evidence | Additional considerations |
| **X** **Favors the comparison** ○ Probably favors the comparison ○ Does not favor either the intervention or the comparison ○ Probably favors the intervention ○ Favors the intervention ○ Varies ○ No included studies | No research evidence identified. |  |
| Equity What would be the impact on health equity? | | |
| Judgement | Research evidence | Additional considerations |
| ○ Reduced ○ Probably reduced ○ Probably no impact ○ Probably increased ○ Increased ○ Varies **X** **Don't know** | No research evidence identified. |  |
| Acceptability Is the intervention acceptable to key stakeholders? | | |
| Judgement | Research evidence | Additional considerations |
| **X** **No** ○ Probably no ○ Probably yes ○ Yes ○ Varies ○ Don't know | No research evidence identified. |  |
| Feasibility Is the intervention feasible to implement? | | |
| Judgement | Research evidence | Additional considerations |
| **X** **No** ○ Probably no ○ Probably yes ○ Yes ○ Varies ○ Don't know | No research evidence identified. |  |

# Summary of judgements

|  | **Judgement** | | | | | | |
| --- | --- | --- | --- | --- | --- | --- | --- |
| **Problem** | **No** | Probably no | Probably yes | Yes |  | Varies | Don't know |
| **Desirable Effects** | **Trivial** | Small | Moderate | Large |  | Varies | Don't know |
| **Undesirable Effects** | Trivial | Small | Moderate | **Large** |  | Varies | Don't know |
| **Certainty of evidence** | Very low | **Low** | Moderate | High |  |  | No included studies |
| **Values** | Important uncertainty or variability | Possibly important uncertainty or variability | Probably no important uncertainty or variability | **No important uncertainty or variability** |  |  |  |
| **Balance of effects** | **Favors the comparison** | Probably favors the comparison | Does not favor either the intervention or the comparison | Probably favors the intervention | Favors the intervention | Varies | Don't know |
| **Resources required** | Large costs | Moderate costs | **Negligible costs and savings** | Moderate savings | Large savings | Varies | Don't know |
| **Certainty of evidence of required resources** | Very low | **Low** | Moderate | High |  |  | No included studies |
| **Cost effectiveness** | **Favors the comparison** | Probably favors the comparison | Does not favor either the intervention or the comparison | Probably favors the intervention | Favors the intervention | Varies | No included studies |
| **Equity** | Reduced | Probably reduced | Probably no impact | Probably increased | Increased | Varies | **Don't know** |
| **Acceptability** | **No** | Probably no | Probably yes | Yes |  | Varies | Don't know |
| **Feasibility** | **No** | Probably no | Probably yes | Yes |  | Varies | Don't know |

# Type of recommendation

| Strong recommendation against the intervention | Conditional recommendation against the intervention | Conditional recommendation for either the intervention or the comparison | Conditional recommendation for the intervention | Strong recommendation for the intervention |
| --- | --- | --- | --- | --- |
| **X** | ○ | ○ | ○ | ○ |

# Conclusions

| Recommendation |
| --- |
| We recommend against the use of bethanechol in the treatment of GERD in infants and children because of their limited evidence of efficacy. |
| Justification |
| Bethanechol, a direct cholinergic agonist, is not FDA-approved for pediatric use, has uncertain efficacy, and carries a high risk of adverse events. Still, solid evidence supporting its use in pediatric GERD is lacking. There is insufficient evidence to recommend bethanechol for GERD treatment in infants and children. |

| Subgroup considerations |
| --- |
|  |
| Implementation considerations |
|  |

| Monitoring and evaluation |
| --- |
|  |
| Research priorities |
|  |
| REFERENCES SUMMARY See the main text of PICO 5 and Additional File 2. |

## PICO 6. What is the effectiveness of different non-pharmacological treatment options for GER and GERD in infants, children, and adolescents?

| Question | |
| --- | --- |
| **Should Thickened Formulas VS Standard Formula be used for children with GER/GERD?** | |
| **Population:** | Children (infants) with GER/GERD |
| **Intervention:** | Protein Hydrolysed or Thickened Formula |
| **Comparison:** | Standard Formula |
| **Main outcomes:** | - Vomiting/regurgitation (frequency) - Infant Gastro-Esophageal Reflux Questionnaire Revised (I-GERQ-R) - Total number of reflux events - Estimated volume regurgitated - Respiratory symptoms, nocturnal cough, asthma (frequency) - Weight Gain - Adverse events |
| **Setting:** | **OUTPATIENTS** |
| **Perspective:** | Clinical recommendation |
| **Background:** | More than 25% of infants have daily episodes of regurgitation that, when frequent, copious, or with additional symptoms, can lead to parental concerns, impaired growth, medical referrals, changes in formula, and reduced quality of life. Different protein hydrolysed and thickened infant formulas are available on the market for formula-fed infants with persistent regurgitation. |
| **Conflict of interests:** | SIP (Italian Society of Pediatrics) conflict of interest declaration and management policies were applied and the following panel members were voting panel members (determining the direction and strength of the recommendation): All.  Panel members recused as a result of risk of conflicts of interest: None. |

# Assessment

| Problem Is the problem a priority? | | |
| --- | --- | --- |
| Judgement | Research evidence | Additional considerations |
| ○ No ○ Probably no **x Probably yes** ○ Yes ○ Varies ○ Don't know | Reducing GER symptoms may significantly lower the risk of drug abuse in infants with functional regurgitation and uncomplicated GER. Protein hydrolysed and thickened formulas could provide a safe and beneficial option for formula-fed term infants suffering from persistent and troublesome regurgitation who do not respond to parental reassurance, education, or conservative treatment, as these formulas help decrease episodes of regurgitation and vomiting. | Breast feeding should always be recommended and supported in all infants with GER/GERD symptoms. |
| Desirable Effects How substantial are the desirable anticipated effects? | | |
| Judgement | Research evidence | Additional considerations |
| ○ Trivial **x Small** ○ Moderate ○ Large ○ Varies ○ Don't know | For research evidence on Desirable and Undesirable anticipated effects, as well as the certainty of this evidence, see the Evidence Profile. |  |
| Undesirable Effects How substantial are the undesirable anticipated effects? | | |
| Judgement | Research evidence | Additional considerations |
| ○ Trivial **x Small** ○ Moderate ○ Large ○ Varies ○ Don't know | For research evidence on Desirable and Undesirable anticipated effects, as well as the certainty of this evidence, see the Evidence Profile. |  |
| Certainty of evidence What is the overall certainty of the evidence of effects? | | |
| Judgement | Research evidence | Additional considerations |
| ○ Very low **x Low** ○ Moderate ○ High ○ No included studies | For research evidence on Desirable and Undesirable anticipated effects, as well as the certainty of this evidence, see the Evidence Profile. |  |
| Values Is there important uncertainty about or variability in how much people value the main outcomes? | | |
| Judgement | Research evidence | Additional considerations |
| ○ Important uncertainty or variability ○ Possibly important uncertainty or variability **x Probably no important uncertainty or variability** ○ No important uncertainty or variability | No research evidence identified. |  |
| Balance of effects Does the balance between desirable and undesirable effects favor the intervention or the comparison? | | |
| Judgement | Research evidence | Additional considerations |
| ○ Favors the comparison ○ Probably favors the comparison ○ Does not favor either the intervention or the comparison ○ Probably favors the intervention **x Favors the intervention** ○ Varies ○ Don't know | Evidence supports that thickening feeds can benefit infants with persistent, copious and frequent episodes of regurgitations that do not respond to conservative treatment. Formulas with extensively hydrolysed proteins may reduce persistent symptoms of GER particularly when related to cow’s milk allergy | There is insufficient comparative data to determine the most clinical effective thickening agent or the superiority of one thickened or protein hydrolysed formulas over others. Commercial thickened formulas can be preferred to home-made thickened formulas due to nutritional balanced and controlled composition and safety profile. |
| Resources required | | |
| Judgement | Research evidence | Additional considerations |
| ○ Large costs **x Moderate costs** ○ Negligible costs and savings ○ Moderate savings ○ Large savings ○ Varies ○ Don't know | No research evidence identified. | Cost of thickened or hydrolysed formulas may vary across countries and among different formulas |
| Certainty of evidence of required resources What is the certainty of the evidence of resource requirements (costs)? | | |
| Judgement | Research evidence | Additional considerations |
| ○ Very low ○ Low ○ Moderate ○ High **x No included studies** | No research evidence identified | Cost of thickened or hydrolysed formulas may vary across countries and among different formulas |
| Cost effectiveness Does the cost-effectiveness of the intervention favor the intervention or the comparison? | | |
| Judgement | Research evidence | Additional considerations |
| ○ Favors the comparison ○ Probably favors the comparison ○ Does not favor either the intervention or the comparison ○ Probably favors the intervention ○ Favors the intervention ○ Varies **x No included studies** | No research evidence identified. | Cost of thickened or hydrolysed formulas may vary across countries and among different formulas. Breast feeding should always be recommended and supported in all infants with GER/GERD symptoms |
| Equity What would be the impact on health equity? | | |
| Judgement | Research evidence | Additional considerations |
| ○ Reduced ○ Probably reduced **X Probably no impact** ○ Probably increased ○ Increased ○ Varies ○ Don't know | No research evidence identified | The panel agreed that there is no reason that Thickened or hydrolysed Formulas would not be provided to any subgroup of formula-fed populations when indicated. |
| Acceptability Is the intervention acceptable to key stakeholders? | | |
| Judgement | Research evidence | Additional considerations |
| ○ No ○ Probably no **x  Probably yes** ○ Yes ○ Varies ○ Don't know | No research evidence identified. | Taste and acceptability may vary among different formulas depending on the composition of the formulas and infant preference. |
| Feasibility Is the intervention feasible to implement? | | |
| Judgement | Research evidence | Additional considerations |
| ○ No ○ Probably no **x Probably yes** ○ Yes ○ Varies ○ Don't know | No research evidence identified. | The panel agreed that some hydrolysed or thickened formulas are not available in some countries and that price of formulas and accessibility may vary across countries. |

# Summary of judgements

|  | **Judgement** | | | | | | |
| --- | --- | --- | --- | --- | --- | --- | --- |
| **Problem** | No | Probably no | **Probably yes** | Yes |  | Varies | Don't know |
| **Desirable Effects** | Trivial | **Small** | Moderate | Large |  | Varies | Don't know |
| **Undesirable Effects** | Trivial | **Small** | Moderate | Large |  | Varies | Don't know |
| **Certainty of evidence** | Very low | **Low** | Moderate | High |  |  | No included studies |
| **Values** | Important uncertainty or variability | Possibly important uncertainty or variability | **Probably no important uncertainty or variability** | No important uncertainty or variability |  |  |  |
| **Balance of effects** | Favors the comparison | Probably favors the comparison | Does not favor either the intervention or the comparison | Probably favors the intervention | **Favors the intervention** | Varies | Don't know |
| **Resources required** | Large costs | **Moderate costs** | Negligible costs and savings | Moderate savings | Large savings | Varies | Don't know |
| **Certainty of evidence of required resources** | Very low | Low | Moderate | High |  |  | **No included studies** |
| **Cost effectiveness** | Favors the comparison | Probably favors the comparison | Does not favor either the intervention or the comparison | Probably favors the intervention | Favors the intervention | Varies | **No included studies** |
| **Equity** | Reduced | Probably reduced | **Probably no impact** | Probably increased | Increased | Varies | Don't know |
| **Acceptability** | No | Probably no | **Probably yes** | Yes |  | Varies | Don't know |
| **Feasibility** | No | Probably no | **Probably yes** | Yes |  | Varies | Don't know |

# Type of recommendation

| Strong recommendation against the intervention | Conditional recommendation against the intervention | Conditional recommendation for either the intervention or the comparison | Conditional recommendation for the intervention | Strong recommendation for the intervention |
| --- | --- | --- | --- | --- |
| ○ | ○ | ○ | ○ | ○ |

# Conclusions

| Recommendation |
| --- |
| The panel recommends supporting breast feeding in all infants with GER/GERD symptoms and considering a thickened formula for formula-fed infants who have ongoing, frequent, and troubling regurgitation that does not improve with parental reassurance and education. An extensive protein hydrolysed formula may be considered in infants with persistent GER symptoms not improving with conservative management and associated with other symptoms suggestive of cow’s milk allergy. The panel also recommends regularly monitoring symptom improvement and avoiding protracted unnecessary intervention. |
| Justification |
|  |

| Subgroup considerations |
| --- |
| Breast feeding should always be recommended and supported in all infants with GER/GERD symptoms. The panel advises against using a thickened formula for preterm infants because of insufficient data and possible risks to this vulnerable group.  The panel advises against using a home-thickened formula with any thickening agents, due to insufficient data and potential risks of adverse symptoms and unbalanced nutrition. In infants improving on extensively protein hydrolysed formulas the panel recommends to schedule a cow’s milk challenge to rule out or properly diagnose cow’s milk allergy |
| Implementation considerations |
|  |

| Monitoring and evaluation |
| --- |
| The panel recommends regular monitoring and evaluation of clinical benefit and possible adverse effects in all infants fed with thickened or hydrolysed formulas to avoid unnecessary or protracted use. |
| Research priorities |
| None |
| REFERENCES SUMMARY See the main text of PICO 6 and Additional File 2. |

| Question | |
| --- | --- |
| **Should Probiotics VS No treatment or Placebo be used for children with GER/GERD?** | |
| **Population:** | Children with GER/GERD |
| **Intervention:** | Probiotics |
| **Comparison:** | No treatment or Placebo |
| **Main outcomes:** | - Vomiting/regurgitation (frequency) - Infant Gastro-Esophageal Reflux Questionnaire Revised (I-GERQ-R) - Total number of reflux events - Estimated volume regurgitated - Respiratory symptoms, nocturnal cough, asthma (frequency) - Weight Gain - Adverse events |
| **Setting:** | outpatients |
| **Perspective:** | clinical recommendation |
| **Background:** | The influence of microbiota on the pathophysiology of GER and regurgitation still needs to be fully clarified. Probiotics can enhance gut homeostasis by influencing intestinal barrier function, immune response, gut motility, and gut-brain interaction.  Evidence for the potential effect of probiotics on GER/GERD is currently scanty. |
| **Conflict of interests:** | SIP (Italian Society of Pediatrics) conflict of interest declaration and management policies were applied and the following panel members were voting panel members (determining the direction and strength of the recommendation): All.  Panel members recused as a result of risk of conflicts of interest: None. |

# Assessment

| Problem Is the problem a priority? | | |
| --- | --- | --- |
| Judgement | Research evidence | Additional considerations |
| ○ No ○ Probably no **x Probably yes** ○ Yes ○ Varies ○ Don't know | Probiotics may influence gastric emptying and possible GER symptoms in infants.  Although recent studies have emerged, the understanding of the role of probiotics and microbiota in GER and regurgitation remains limited. | Research on probiotic interventions for regurgitation in infants is limited, and evidence is still scarce. While no clear side effects from probiotic treatment have been identified, significant health benefits are not expected for infants and children with GERD. Conversely, the potential role of probiotics in preventing PPI side effects may require further investigation. |
| Desirable Effects How substantial are the desirable anticipated effects? | | |
| Judgement | Research evidence | Additional considerations |
| ○ Trivial **x Small** ○ Moderate ○ Large ○ Varies ○ Don't know | For research evidence on Desirable and Undesirable anticipated effects, as well as the certainty of this evidence, see the Evidence Profile. |  |
| Undesirable Effects How substantial are the undesirable anticipated effects? | | |
| Judgement | Research evidence | Additional considerations |
| **x Trivial** ○ Small ○ Moderate ○ Large ○ Varies ○ Don't know | For research evidence on Desirable and Undesirable anticipated effects, as well as the certainty of this evidence, see the Evidence Profile. |  |
| Certainty of evidence What is the overall certainty of the evidence of effects? | | |
| Judgement | Research evidence | Additional considerations |
| ○ Very low **x Low** ○ Moderate ○ High ○ No included studies | For research evidence on Desirable and Undesirable anticipated effects, as well as the certainty of this evidence, see the Evidence Profile. |  |
| Values Is there important uncertainty about or variability in how much people value the main outcomes? | | |
| Judgement | Research evidence | Additional considerations |
| ○ Important uncertainty or variability ○ Possibly important uncertainty or variability **x Probably no important uncertainty or variability** ○ No important uncertainty or variability | No research evidence identified |  |
| Balance of effects Does the balance between desirable and undesirable effects favor the intervention or the comparison? | | |
| Judgement | Research evidence | Additional considerations |
| ○ Favors the comparison ○ Probably favors the comparison ○ Does not favor either the intervention or the comparison **x Probably favors the intervention** ○ Favors the intervention ○ Varies ○ Don't know | Probiotics, particularly the strains *Lactobacillus reuteri* DSM17938 and *Bifidobacterium animalis* subsp*. lactis* BB-12, have been shown to reduce the occurrence of regurgitation episodes and crying time or a GER infant score (I-GERQ-R) in selected infants. However, there is no current evidence of the efficacy of a specific strain of probiotic in treating infant regurgitation and GERD. | Available studies included a small number of infants or focused on prevention of regurgitation or have an open design or lack a control group. |
| Resources required | | |
| Judgement | Research evidence | Additional considerations |
| ○ Large costs ○ Moderate costs **x Negligible costs and savings** ○ Moderate savings ○ Large savings ○ Varies ○ Don't know | No research evidence identified |  |
| Certainty of evidence of required resources What is the certainty of the evidence of resource requirements (costs)? | | |
| Judgement | Research evidence | Additional considerations |
| ○ Very low ○ Low ○ Moderate ○ High **x No included studies** | No research evidence identified. |  |
| Cost effectiveness Does the cost-effectiveness of the intervention favor the intervention or the comparison? | | |
| Judgement | Research evidence | Additional considerations |
| ○ Favors the comparison ○ Probably favors the comparison ○ Does not favor either the intervention or the comparison ○ Probably favors the intervention ○ Favors the intervention ○ Varies **x No included studies** | No research evidence identified. |  |
| Equity What would be the impact on health equity? | | |
| Judgement | Research evidence | Additional considerations |
| ○ Reduced ○ Probably reduced **x Probably no impact** ○ Probably increased ○ Increased ○ Varies ○ Don't know | No research evidence identified. |  |
| Acceptability Is the intervention acceptable to key stakeholders? | | |
| Judgement | Research evidence | Additional considerations |
| ○ No ○ Probably no **x Probably yes** ○ Yes ○ Varies ○ Don't know | No research evidence identified. | Probiotic treatment is generally well accepted by patients and families, with limited costs. Only strains with demonstrated efficacy and safety should be considered |
| Feasibility Is the intervention feasible to implement? | | |
| Judgement | Research evidence | Additional considerations |
| ○ No ○ Probably no ○ Probably yes **x Yes** ○ Varies ○ Don't know | No research evidence identified. |  |

# Summary of judgements

|  | **Judgement** | | | | | | |
| --- | --- | --- | --- | --- | --- | --- | --- |
| **Problem** | No | Probably no | **Probably yes** | Yes |  | Varies | Don't know |
| **Desirable Effects** | Trivial | **Small** | Moderate | Large |  | Varies | Don't know |
| **Undesirable Effects** | **Trivial** | Small | Moderate | Large |  | Varies | Don't know |
| **Certainty of evidence** | Very low | **Low** | Moderate | High |  |  | No included studies |
| **Values** | Important uncertainty or variability | Possibly important uncertainty or variability | **Probably no important uncertainty or variability** | No important uncertainty or variability |  |  |  |
| **Balance of effects** | Favors the comparison | Probably favors the comparison | Does not favor either the intervention or the comparison | **Probably favors the intervention** | Favors the intervention | Varies | Don't know |
| **Resources required** | Large costs | Moderate costs | **Negligible costs and savings** | Moderate savings | Large savings | Varies | Don't know |
| **Certainty of evidence of required resources** | Very low | Low | Moderate | High |  |  | **No included studies** |
| **Cost effectiveness** | Favors the comparison | Probably favors the comparison | Does not favor either the intervention or the comparison | Probably favors the intervention | Favors the intervention | Varies | **No included studies** |
| **Equity** | Reduced | Probably reduced | **Probably no impact** | Probably increased | Increased | Varies | Don't know |
| **Acceptability** | No | Probably no | **Probably yes** | Yes |  | Varies | Don't know |
| **Feasibility** | No | Probably no | Probably yes | **Yes** |  | Varies | Don't know |

# Type of recommendation

| Strong recommendation against the intervention | Conditional recommendation against the intervention | Conditional recommendation for either the intervention or the comparison | Conditional recommendation for the intervention | Strong recommendation for the intervention |
| --- | --- | --- | --- | --- |
| ○ | ○ | **x** | ○ | ○ |

# Conclusions

| Recommendation |
| --- |
| Due to limited evidence and the heterogeneity in intervention trials, no specific recommendation can be made for or against the use of probiotics to prevent or treat infant regurgitation at this time. |
| Justification |
| Currently, there is no evidence supporting the effectiveness of a specific strain of probiotic in treating infant regurgitation. The potential preventive effect of *L. reuteri* DSM17948 on the onset of troublesome regurgitation necessitates further well-designed double-blind RCTs with a large population and an analysis of possible confounding variables, including the volume of formula intake, neonatal complications, intercurrent infections, and concomitant treatments. |

| Subgroup considerations |
| --- |
|  |
| Implementation considerations |
|  |

| Monitoring and evaluation |
| --- |
|  |
| Research priorities |
| Further research is needed through randomized double-blind studies involving larger populations to provide insights into the effects of probiotics on infant regurgitation, as well as for GER and GERD in children, and for patients undergoing PPI therapy.  Moreover, analysis of possible confounding variables (i.e., volume intake of formula, neonatal complications, intercurrent infections, and concomitant treatments) should also be considered in studies assessing the efficacy of probiotics. |
| REFERENCES SUMMARY See the main text of PICO 6 and Additional File 2. |

| Question | |
| --- | --- |
| **Should Positioning therapy VS Other Positioning therapy be used for children with GER/GERD?** | |
| **Population:** | Children with GER/GERD |
| **Intervention:** | Positioning therapy |
| **Comparison:** | Other Positioning therapy |
| **Main outcomes:** | - Vomiting/regurgitation (frequency) - Infant Gastro-Esophageal Reflux Questionnaire Revised (I-GERQ-R) - Total number of reflux events - Estimated volume regurgitated - Respiratory symptoms, nocturnal cough, asthma (frequency) - Weight Gain - Adverse events |
| **Setting:** | outpatients |
| **Perspective:** | clinical recommendation |
| **Background:** | Traditionally, postural treatment has been considered in patients with GERD symptoms. Various positioning strategies for infants have been explored, including the use of infant seats and elevated cribs. Importantly, some positions may also increase the risk of sudden infant death syndrome. It is essential for pediatricians to be aware of the potential risks and benefits associated with positional treatment in infants to prevent safety concerns. |
| **Conflict of interests:** | SIP (Italian Society of Pediatrics) conflict of interest declaration and management policies were applied and the following panel members were voting panel members (determining the direction and strength of the recommendation): All.  Panel members recused as a result of risk of conflicts of interest: None. |

# Assessment

| Problem Is the problem a priority? | | |
| --- | --- | --- |
| Judgement | Research evidence | Additional considerations |
| ○ No ○ Probably no **x Probably yes** ○ Yes ○ Varies ○ Don't know | Prioritizing postural treatment for GERD symptoms is useful to reduce inappropriate drug use in infants. Nonetheless, some positions could increase the risk of sudden infant death syndrome. It is crucial to understand the potential risks and benefits of using positional treatment in infants. |  |
| Desirable Effects How substantial are the desirable anticipated effects? | | |
| Judgement | Research evidence | Additional considerations |
| ○ Trivial **x Small** ○ Moderate ○ Large ○ Varies ○ Don't know | For research evidence on Desirable and Undesirable anticipated effects, as well as the certainty of this evidence, see the Evidence Profile. |  |
| Undesirable Effects How substantial are the undesirable anticipated effects? | | |
| Judgement | Research evidence | Additional considerations |
| ○ Trivial ○ Small **x Moderate** ○ Large ○ Varies ○ Don't know | For research evidence on Desirable and Undesirable anticipated effects, as well as the certainty of this evidence, see the Evidence Profile. | Concerns about safety emerge in relation to the prone and lateral sleeping positions of infants, as these positions have been associated with increased risk of sudden infant death syndrome. |
| Certainty of evidence What is the overall certainty of the evidence of effects? | | |
| Judgement | Research evidence | Additional considerations |
| ○ Very low **x Low** ○ Moderate ○ High ○ No included studies | For research evidence on Desirable and Undesirable anticipated effects, as well as the certainty of this evidence, see the Evidence Profile. |  |
| Values Is there important uncertainty about or variability in how much people value the main outcomes? | | |
| Judgement | Research evidence | Additional considerations |
| ○ Important uncertainty or variability ○ Possibly important uncertainty or variability **x Probably no important uncertainty or variability** ○ No important uncertainty or variability | No research evidence identified. |  |
| Balance of effects Does the balance between desirable and undesirable effects favor the intervention or the comparison? | | |
| Judgement | Research evidence | Additional considerations |
| ○ Favors the comparison ○ Probably favors the comparison ○ Does not favor either the intervention or the comparison **x Probably favors the intervention** ○ Favors the intervention ○ Varies ○ Don't know | Evidence supporting head elevation in infants and children for preventing GERD symptoms is lacking. Conversely, both prone and left lateral positions seem to effectively decrease reflux episodes, although the advantages of positional treatment for GER-related signs or symptoms are still unclear and should be carefully balanced against the potential risk of sudden infant death syndrome in the first months of life. | Concerns about safety arise regarding infants' prone and lateral sleeping positions, as these positions are associated with increased risk of sudden infant death syndrome and are therefore contraindicated. |
| Resources required | | |
| Judgement | Research evidence | Additional considerations |
| ○ Large costs ○ Moderate costs **x Negligible costs and savings** ○ Moderate savings ○ Large savings ○ Varies ○ Don't know | No research evidence identified. |  |
| Certainty of evidence of required resources What is the certainty of the evidence of resource requirements (costs)? | | |
| Judgement | Research evidence | Additional considerations |
| ○ Very low ○ Low ○ Moderate ○ High **x No included studies** | No research evidence identified. |  |
| Cost effectiveness Does the cost-effectiveness of the intervention favor the intervention or the comparison? | | |
| Judgement | Research evidence | Additional considerations |
| ○ Favors the comparison ○ Probably favors the comparison ○ Does not favor either the intervention or the comparison ○ Probably favors the intervention ○ Favors the intervention ○ Varies **x No included studies** | No research evidence identified. |  |
| Equity What would be the impact on health equity? | | |
| Judgement | Research evidence | Additional considerations |
| ○ Reduced ○ Probably reduced **x Probably no impact** ○ Probably increased ○ Increased ○ Varies ○ Don't know | No research evidence identified. |  |
| Acceptability Is the intervention acceptable to key stakeholders? | | |
| Judgement | Research evidence | Additional considerations |
| ○ No ○ Probably no ○ Probably yes **x Yes** ○ Varies ○ Don't know | No research evidence identified. |  |
| Feasibility Is the intervention feasible to implement? | | |
| Judgement | Research evidence | Additional considerations |
| ○ No ○ Probably no ○ Probably yes **x Yes** ○ Varies ○ Don't know | No research evidence identified. |  |

# Summary of judgements

|  | **Judgement** | | | | | | |
| --- | --- | --- | --- | --- | --- | --- | --- |
| **Problem** | No | Probably no | **Probably yes** | Yes |  | Varies | Don't know |
| **Desirable Effects** | Trivial | **Small** | Moderate | Large |  | Varies | Don't know |
| **Undesirable Effects** | Trivial | Small | **Moderate** | Large |  | Varies | Don't know |
| **Certainty of evidence** | Very low | **Low** | Moderate | High |  |  | No included studies |
| **Values** | Important uncertainty or variability | Possibly important uncertainty or variability | **Probably no important uncertainty or variability** | No important uncertainty or variability |  |  |  |
| **Balance of effects** | Favors the comparison | Probably favors the comparison | Does not favor either the intervention or the comparison | **Probably favors the intervention** | Favors the intervention | Varies | Don't know |
| **Resources required** | Large costs | Moderate costs | **Negligible costs and savings** | Moderate savings | Large savings | Varies | Don't know |
| **Certainty of evidence of required resources** | Very low | Low | Moderate | High |  |  | **No included studies** |
| **Cost effectiveness** | Favors the comparison | Probably favors the comparison | Does not favor either the intervention or the comparison | Probably favors the intervention | Favors the intervention | Varies | **No included studies** |
| **Equity** | Reduced | Probably reduced | **Probably no impact** | Probably increased | Increased | Varies | Don't know |
| **Acceptability** | No | Probably no | Probably yes | **Yes** |  | Varies | Don't know |
| **Feasibility** | No | Probably no | Probably yes | **Yes** |  | Varies | Don't know |

# Type of recommendation

| Strong recommendation against the intervention | Conditional recommendation against the intervention | Conditional recommendation for either the intervention or the comparison | Conditional recommendation for the intervention | Strong recommendation for the intervention |
| --- | --- | --- | --- | --- |
| **x** | ○ | ○ | ○ | ○ |

# Conclusions

| Recommendation |
| --- |
| The panel recommends against the routine use of positional therapy to alleviate GERD symptoms in sleeping infants due to insufficient evidence regarding its efficacy (head elevation) and safety (lateral and prone positions), especially considering the heightened risks associated with sudden infant death syndrome. |
| Justification |
|  |

| Subgroup considerations |
| --- |
| Positional treatment may be considered in older infants and children with GERD. |
| Implementation considerations |
|  |

| Monitoring and evaluation |
| --- |
|  |
| Research priorities |
|  |
| REFERENCES SUMMARY See the main text of PICO 6 and Additional File 2. |

| Question | |
| --- | --- |
| **Should Alginates vs Placebo be used for children with GER/GERD?** | |
| **Population:** | Children with GER/GERD |
| **Intervention:** | Alginate |
| **Comparison:** | Placebo |
| **Main outcomes:** | - Vomiting/regurgitation (frequency) - Infant Gastro-Esophageal Reflux Questionnaire Revised (I-GERQ-R) - Heartburn (frequency) - Epigastric pain (frequency) - Respiratory symptoms, nocturnal cough, asthma (frequency) - Severity of esophagitis - Adverse events |
| **Setting:** | Outpatients and inpatients |
| **Perspective:** | clinical recommendation |
| **Background:** | Pediatricians should know if alginates may be useful for treating GER/GERD symptoms and understand the risk/benefit ratio of alginate treatment compared with placebo. |
| **Conflict of interests:** | SIP (Italian Society of Pediatrics) conflict of interest declaration and management policies were applied and the following panel members were voting panel members (determining the direction and strength of the recommendation): All.  Panel members recused as a result of risk of conflicts of interest: None. |

# Assessment

| Problem Is the problem a priority? | | |
| --- | --- | --- |
| Judgement | Research evidence | Additional considerations |
| ○ No ○ Probably no ○ Probably yes **x Yes** ○ Varies ○ Don't know | Using alginates to alleviate visible vomiting, regurgitation, or other signs and symptoms of GER/GERD in children is a widespread practice. Evaluating whether alginates offer clinical advantages or side effects compared to a placebo could enhance patient comfort and improve clinical outcomes. |  |
| Desirable Effects How substantial are the desirable anticipated effects? | | |
| Judgement | Research evidence | Additional considerations |
| ○ Trivial **x Small** ○ Moderate ○ Large ○ Varies ○ Don't know | For research evidence on Desirable and Undesirable anticipated effects, as well as the certainty of this evidence, see the Evidence Profile. |  |
| Undesirable Effects How substantial are the undesirable anticipated effects? | | |
| Judgement | Research evidence | Additional considerations |
| ○ Trivial **x Small** ○ Moderate ○ Large ○ Varies ○ Don't know | For research evidence on Desirable and Undesirable anticipated effects, as well as the certainty of this evidence, see the Evidence Profile. |  |
| Certainty of evidence What is the overall certainty of the evidence of effects? | | |
| Judgement | Research evidence | Additional considerations |
| ○ Very low **x Low** ○ Moderate ○ High ○ No included studies | For research evidence on Desirable and Undesirable anticipated effects, as well as the certainty of this evidence, see the Evidence Profile. |  |
| Values Is there important uncertainty about or variability in how much people value the main outcomes? | | |
| Judgement | Research evidence | Additional considerations |
| ○ Important uncertainty or variability ○ Possibly important uncertainty or variability **x Probably no important uncertainty or variability** ○ No important uncertainty or variability | No research evidence identified. |  |
| Balance of effects Does the balance between desirable and undesirable effects favor the intervention or the comparison? | | |
| Judgement | Research evidence | Additional considerations |
| ○ Favors the comparison ○ Probably favors the comparison ○ Does not favor either the intervention or the comparison **x Probably favors the intervention** ○ Favors the intervention ○ Varies ○ Don't know | Limited evidence indicates that alginate formulations may relieve GERD symptoms and specific MII-pH parameters in infants and children. Nevertheless, the current body of research has limitations, including small sample sizes and differing formulations. More extensive studies are needed to enhance our understanding of their efficacy and safety profiles. |  |
| Resources required | | |
| Judgement | Research evidence | Additional considerations |
| ○ Large costs ○ Moderate costs **x Negligible costs and savings** ○ Moderate savings ○ Large savings ○ Varies ○ Don't know | No research evidence identified. |  |
| Certainty of evidence of required resources What is the certainty of the evidence of resource requirements (costs)? | | |
| Judgement | Research evidence | Additional considerations |
| ○ Very low ○ Low ○ Moderate ○ High **x No included studies** | No research evidence identified. |  |
| Cost effectiveness Does the cost-effectiveness of the intervention favor the intervention or the comparison? | | |
| Judgement | Research evidence | Additional considerations |
| ○ Favors the comparison ○ Probably favors the comparison ○ Does not favor either the intervention or the comparison ○ Probably favors the intervention ○ Favors the intervention ○ Varies **x No included studies** | No research evidence identified. |  |
| Equity What would be the impact on health equity? | | |
| Judgement | Research evidence | Additional considerations |
| ○ Reduced ○ Probably reduced **x Probably no impact** ○ Probably increased ○ Increased ○ Varies ○ Don't know | No research evidence identified. |  |
| Acceptability Is the intervention acceptable to key stakeholders? | | |
| Judgement | Research evidence | Additional considerations |
| ○ No ○ Probably no **x Probably yes** ○ Yes ○ Varies ○ Don't know | No research evidence identified. |  |
| Feasibility Is the intervention feasible to implement? | | |
| Judgement | Research evidence | Additional considerations |
| ○ No ○ Probably no ○ Probably yes **X Yes** ○ Varies ○ Don't know | No research evidence identified. |  |

# Summary of judgements

|  | **Judgement** | | | | | | |
| --- | --- | --- | --- | --- | --- | --- | --- |
| **Problem** | No | Probably no | Probably yes | **Yes** |  | Varies | Don't know |
| **Desirable Effects** | Trivial | **Small** | Moderate | Large |  | Varies | Don't know |
| **Undesirable Effects** | Trivial | **Small** | Moderate | Large |  | Varies | Don't know |
| **Certainty of evidence** | Very low | **Low** | Moderate | High |  |  | No included studies |
| **Values** | Important uncertainty or variability | Possibly important uncertainty or variability | **Probably no important uncertainty or variability** | No important uncertainty or variability |  |  |  |
| **Balance of effects** | Favors the comparison | Probably favors the comparison | Does not favor either the intervention or the comparison | **Probably favors the intervention** | Favors the intervention | Varies | Don't know |
| **Resources required** | Large costs | Moderate costs | **Negligible costs and savings** | Moderate savings | Large savings | Varies | Don't know |
| **Certainty of evidence of required resources** | Very low | Low | Moderate | High |  |  | **No included studies** |
| **Cost effectiveness** | Favors the comparison | Probably favors the comparison | Does not favor either the intervention or the comparison | Probably favors the intervention | Favors the intervention | Varies | **No included studies** |
| **Equity** | Reduced | Probably reduced | **Probably no impact** | Probably increased | Increased | Varies | Don't know |
| **Acceptability** | No | Probably no | **Probably yes** | Yes |  | Varies | Don't know |
| **Feasibility** | No | Probably no | Probably yes | **Yes** |  | Varies | Don't know |

# Type of recommendation

| Strong recommendation against the intervention | Conditional recommendation against the intervention | Conditional recommendation for either the intervention or the comparison | Conditional recommendation for the intervention | Strong recommendation for the intervention |
| --- | --- | --- | --- | --- |
| ○ | ○ | ○ | **x** | ○ |

# Conclusions

| Recommendation |
| --- |
| The panel suggests considering alginate formulations for infants and children suffering from troublesome, persistent GERD symptoms that do not respond well to conservative treatments. Efficacy should be evaluated after 1 to 2 weeks with careful monitoring, and efforts should be made to discontinue use to avoid unnecessary prolonged treatment. Extra caution is recommended for individuals at risk of intestinal obstruction, renal problems, or those using thickened formulas. |
| Justification |
| Research suggests that alginate formulations could alleviate GERD symptoms and particular MII-pH readings in infants and children. However, current studies have limitations, such as small sample sizes and varied formulations. Larger studies are needed to enhance our comprehension of their effectiveness and safety. |

| Subgroup considerations |
| --- |
| Alginate formulations are not recommended in infants fed with concomitant thickening agent or thickened formula; alginate formulation with sodium content should be carefully used and monitored in preterm infants or in patients with renal impairment. Treatment of alginate for erosive esophagitis and chronic use for GERD symptoms should be discouraged according to lack of evidence of efficacy and of safety data. |
| Implementation considerations |
| None |

| Monitoring and evaluation |
| --- |
| Clinical efficacy of alginate formulation or thickening agent or thickened formulas should be regularly monitored in all treated patients to avoid unnecessary or protracted use and possible adverse effects. |
| Research priorities |
| Large multicenter randomized controlled studies with adequate sample size, well-designed outcome measures and follow-up, are needed to clarify efficacy of alginate in infants and children with esophageal and extraesophageal symptoms of GERD. |
| REFERENCES SUMMARY See the main text of PICO 6 and Additional File 2. |

| Question | |
| --- | --- |
| **Should Alginates vs Thickened feed be used for children with GER/GERD?** | |
| **Population:** | Children (infants) with GER/GERD |
| **Intervention:** | Alginate |
| **Comparison:** | Thickened feed |
| **Main outcomes:** | - Vomiting/regurgitation (frequency) - Infant Gastro-Esophageal Reflux Questionnaire Revised (I-GERQ-R) - Heartburn (frequency) - Epigastric pain (frequency) - Respiratory symptoms, nocturnal cough, asthma (frequency) - Severity of esophagitis - Adverse events |
| **Setting:** | outpatients |
| **Perspective:** | clinical recommendation |
| **Background:** | Pediatricians should recognize the role of alginates in alleviating symptoms of GER/GERD in infants and assess the cost-benefit comparison of alginates versus thickened feedings. |
| **Conflict of interests:** | SIP (Italian Society of Pediatrics) conflict of interest declaration and management policies were applied and the following panel members were voting panel members (determining the direction and strength of the recommendation): All.  Panel members recused as a result of risk of conflicts of interest: None. |

# Assessment

| Problem Is the problem a priority? | | |
| --- | --- | --- |
| Judgement | Research evidence | Additional considerations |
| ○ No **x Probably no** ○ Probably yes ○ Yes ○ Varies ○ Don't know | Alginates and thickened feeding are frequently used non-pharmacologic approaches to manage GER and GERD in infants, aiming to relieve symptoms. Evaluating the clinical advantages or side effects of alginates in comparison to thickened formulas can enhance patient comfort and may help lower expenses for families. | Breast feeding should always be recommended and supported in all infants with GER/GERD symptoms |
| Desirable Effects How substantial are the desirable anticipated effects? | | |
| Judgement | Research evidence | Additional considerations |
| **x Trivial** ○ Small ○ Moderate ○ Large ○ Varies ○ Don't know | For research evidence on Desirable and Undesirable anticipated effects, as well as the certainty of this evidence, see the Evidence Profile. |  |
| Undesirable Effects How substantial are the undesirable anticipated effects? | | |
| Judgement | Research evidence | Additional considerations |
| **x Trivial** ○ Small ○ Moderate ○ Large ○ Varies ○ Don't know | For research evidence on Desirable and Undesirable anticipated effects, as well as the certainty of this evidence, see the Evidence Profile. |  |
| Certainty of evidence What is the overall certainty of the evidence of effects? | | |
| Judgement | Research evidence | Additional considerations |
| **x Very low** ○ Low ○ Moderate ○ High ○ No included studies | For research evidence on Desirable and Undesirable anticipated effects, as well as the certainty of this evidence, see the Evidence Profile. |  |
| Values Is there important uncertainty about or variability in how much people value the main outcomes? | | |
| Judgement | Research evidence | Additional considerations |
| ○ Important uncertainty or variability ○ Possibly important uncertainty or variability **x Probably no important uncertainty or variability** ○ No important uncertainty or variability | No research evidence identified. |  |
| Balance of effects Does the balance between desirable and undesirable effects favor the intervention or the comparison? | | |
| Judgement | Research evidence | Additional considerations |
| ○ Favors the comparison ○ Probably favors the comparison **x Does not favor either the intervention or the comparison** ○ Probably favors the intervention ○ Favors the intervention ○ Varies ○ Don't know | Both alginates and thickened formulas can alleviate GER symptoms in infants with limited side effects. However, the existing literature has limitations, including small sample sizes. Additional studies are necessary to enhance our understanding of their effectiveness and safety. | Breast feeding should always be recommended and supported in all infants with GER/GERD symptoms |
| Resources required | | |
| Judgement | Research evidence | Additional considerations |
| ○ Large costs ○ Moderate costs **x Negligible costs and savings** ○ Moderate savings ○ Large savings ○ Varies ○ Don't know | Limited evidence exists regarding cost savings when using alginates instead of thickened formula for infants with GERD. |  |
| Certainty of evidence of required resources What is the certainty of the evidence of resource requirements (costs)? | | |
| Judgement | Research evidence | Additional considerations |
| **x Very low** ○ Low ○ Moderate ○ High ○ No included studies | Very low quality of evidence exists regarding cost savings when using alginates instead of thickened formula for infants with GERD. |  |
| Cost effectiveness Does the cost-effectiveness of the intervention favor the intervention or the comparison? | | |
| Judgement | Research evidence | Additional considerations |
| ○ Favors the comparison ○ Probably favors the comparison ○ Does not favor either the intervention or the comparison **x Probably favors the intervention** ○ Favors the intervention ○ Varies ○ No included studies | Alginates seem to be more cost-effective than commercially thickened formulas; however, there is limited evidence regarding this issue and the cost of thickened formulas vary across countries and among different commercial formulas. |  |
| Equity What would be the impact on health equity? | | |
| Judgement | Research evidence | Additional considerations |
| ○ Reduced ○ Probably reduced **x Probably no impact** ○ Probably increased ○ Increased ○ Varies ○ Don't know | No research evidence identified. |  |
| Acceptability Is the intervention acceptable to key stakeholders? | | |
| Judgement | Research evidence | Additional considerations |
| ○ No ○ Probably no **x Probably yes** ○ Yes ○ Varies ○ Don't know | No research evidence identified. |  |
| Feasibility Is the intervention feasible to implement? | | |
| Judgement | Research evidence | Additional considerations |
| ○ No ○ Probably no ○ Probably yes **x Yes** ○ Varies ○ Don't know | No research evidence identified. |  |

# Summary of judgements

|  | **Judgement** | | | | | | |
| --- | --- | --- | --- | --- | --- | --- | --- |
| **Problem** | No | **Probably no** | Probably yes | Yes |  | Varies | Don't know |
| **Desirable Effects** | **Trivial** | Small | Moderate | Large |  | Varies | Don't know |
| **Undesirable Effects** | **Trivial** | Small | Moderate | Large |  | Varies | Don't know |
| **Certainty of evidence** | **Very low** | Low | Moderate | High |  |  | No included studies |
| **Values** | Important uncertainty or variability | Possibly important uncertainty or variability | **Probably no important uncertainty or variability** | No important uncertainty or variability |  |  |  |
| **Balance of effects** | Favors the comparison | Probably favors the comparison | **Does not favor either the intervention or the comparison** | Probably favors the intervention | Favors the intervention | Varies | Don't know |
| **Resources required** | Large costs | Moderate costs | **Negligible costs and savings** | Moderate savings | Large savings | Varies | Don't know |
| **Certainty of evidence of required resources** | **Very low** | Low | Moderate | High |  |  | No included studies |
| **Cost effectiveness** | Favors the comparison | Probably favors the comparison | Does not favor either the intervention or the comparison | **Probably favors the intervention** | Favors the intervention | Varies | No included studies |
| **Equity** | Reduced | Probably reduced | **Probably no impact** | Probably increased | Increased | Varies | Don't know |
| **Acceptability** | No | Probably no | **Probably yes** | Yes |  | Varies | Don't know |
| **Feasibility** | No | Probably no | Probably yes | **Yes** |  | Varies | Don't know |

# Type of recommendation

| Strong recommendation against the intervention | Conditional recommendation against the intervention | Conditional recommendation for either the intervention or the comparison | Conditional recommendation for the intervention | Strong recommendation for the intervention |
| --- | --- | --- | --- | --- |
| ○ | ○ | x | ○ | ○ |

# Conclusions

| Recommendation |
| --- |
| The panel suggests considering both alginate formulations and thickened formulas for infants and children with troublesome, persistent GERD symptoms that do not respond to conservative treatments. While both interventions appear viable, they should be considered separately to avoid increasing side effects. Very limited evidence indicates that alginates may be more cost-effective, but it should be considered the possible difference of cost across countries and among different thickened formulas. |
| Justification |
|  |

| Subgroup considerations |
| --- |
| Alginate formulations are not recommended in infants fed with concomitant thickening agent or thickened formula; alginate formulation with sodium content should be carefully used and monitored in preterm infants or in patients with renal impairment. Treatment of alginate for erosive esophagitis and chronic use for GERD symptoms should be discouraged according to lack of evidence of efficacy and of safety data. |
| Implementation considerations |
| None |

| Monitoring and evaluation |
| --- |
| Clinical efficacy of alginate formulation or thickening agent or thickened formulas should be regularly monitored in all treated patients to avoid unnecessary or protracted use and possible adverse effects. |
| Research priorities |
| Large multicenter randomized controlled studies with adequate sample size, well-designed outcome measures and follow-up, are needed to clarify efficacy of alginate in infants and children with esophageal and extraesophageal symptoms of GERD. |
| REFERENCES SUMMARY See the main text of PICO 6 and Additional File 2. |

| Question | |
| --- | --- |
| **Should Massage therapy VS Sham non-massage therapy be used for children with GER/GERD?** | |
| **Population:** | Children with GER/GERD |
| **Intervention:** | Massage therapy |
| **Comparison:** | Sham non-massage therapy |
| **Main outcomes:** | - Vomiting/regurgitation (frequency) - Infant Gastro-Esophageal Reflux Questionnaire Revised (I-GERQ-R) - Total number of reflux events - Estimated volume regurgitated - Respiratory symptoms, nocturnal cough, asthma (frequency) - Weight Gain - Adverse events |
| **Setting:** | OUTPATIENTS |
| **Perspective:** | CLINICAL RECOMMENDATION |
| **Background:** | Massage therapy may enhance relaxation and sleep while reducing crying and irritability in infants; however, it is not recommended as a primary non-pharmacological treatment for GERD. Pediatricians should understand the risk-benefit ratio of massage therapy when managing GER/GERD symptoms in infants. |
| **Conflict of interests:** | SIP (Italian Society of Pediatrics) conflict of interest declaration and management policies were applied and the following panel members were voting panel members (determining the direction and strength of the recommendation): All.  Panel members recused as a result of risk of conflicts of interest: None. |

# Assessment

| Problem Is the problem a priority? | | |
| --- | --- | --- |
| Judgement | Research evidence | Additional considerations |
| **x No** ○ Probably no ○ Probably yes ○ Yes ○ Varies ○ Don't know | Given the frequent inquiries from parents about complementary treatments for functional disorders in infants and children, massage therapy has been considered for GER/GERD treatment. Its potential to promote relaxation and sleep might alleviate some referral symptoms of infants and children. However, no significant benefits to infant health can be deemed evident in this context also related to very limited studies and risk of bias. Furthermore, the lack of standardized intervention, of cost-effect evaluation and of safety data should be considered. |  |
| Desirable Effects How substantial are the desirable anticipated effects? | | |
| Judgement | Research evidence | Additional considerations |
| **x Trivial** ○ Small ○ Moderate ○ Large ○ Varies ○ Don't know | For research evidence on Desirable and Undesirable anticipated effects, as well as the certainty of this evidence, see the Evidence Profile. |  |
| Undesirable Effects How substantial are the undesirable anticipated effects? | | |
| Judgement | Research evidence | Additional considerations |
| **x Trivial** ○ Small ○ Moderate ○ Large ○ Varies ○ Don't know | For research evidence on Desirable and Undesirable anticipated effects, as well as the certainty of this evidence, see the Evidence Profile. |  |
| Certainty of evidence What is the overall certainty of the evidence of effects? | | |
| Judgement | Research evidence | Additional considerations |
| **x Very low** ○ Low ○ Moderate ○ High ○ No included studies | For research evidence on Desirable and Undesirable anticipated effects, as well as the certainty of this evidence, see the Evidence Profile. |  |
| Values Is there important uncertainty about or variability in how much people value the main outcomes? | | |
| Judgement | Research evidence | Additional considerations |
| ○ Important uncertainty or variability **x Possibly important uncertainty or variability** ○ Probably no important uncertainty or variability ○ No important uncertainty or variability | No research evidence identified. |  |
| Balance of effects Does the balance between desirable and undesirable effects favor the intervention or the comparison? | | |
| Judgement | Research evidence | Additional considerations |
| ○ Favors the comparison ○ Probably favors the comparison **x Does not favor either the intervention or the comparison** ○ Probably favors the intervention ○ Favors the intervention ○ Varies ○ Don't know | No clear evidence supports the intervention's benefits, and uncertainty remains about its ability to reduce GER and GERD symptoms. |  |
| Resources required | | |
| Judgement | Research evidence | Additional considerations |
| ○ Large costs **x Moderate costs** ○ Negligible costs and savings ○ Moderate savings ○ Large savings ○ Varies ○ Don't know | No research evidence identified. |  |
| Certainty of evidence of required resources What is the certainty of the evidence of resource requirements (costs)? | | |
| Judgement | Research evidence | Additional considerations |
| ○ Very low ○ Low ○ Moderate ○ High **x No included studies** | No research evidence identified. |  |
| Cost effectiveness Does the cost-effectiveness of the intervention favor the intervention or the comparison? | | |
| Judgement | Research evidence | Additional considerations |
| ○ Favors the comparison ○ Probably favors the comparison ○ Does not favor either the intervention or the comparison ○ Probably favors the intervention ○ Favors the intervention ○ Varies **x No included studies** | No research evidence identified. |  |
| Equity What would be the impact on health equity? | | |
| Judgement | Research evidence | Additional considerations |
| ○ Reduced ○ Probably reduced **x Probably no impact** ○ Probably increased ○ Increased ○ Varies ○ Don't know | No research evidence identified. |  |
| Acceptability Is the intervention acceptable to key stakeholders? | | |
| Judgement | Research evidence | Additional considerations |
| ○ No ○ Probably no ○ Probably yes ○ Yes **x Varies** ○ Don't know | No research evidence identified. |  |
| Feasibility Is the intervention feasible to implement? | | |
| Judgement | Research evidence | Additional considerations |
| ○ No ○ Probably no ○ Probably yes ○ Yes **x Varies** ○ Don't know | No research evidence identified. | Some families may face barriers to accessing infant massage therapy due to potential expenses and differing availability based on social and geographical factors. |

# Summary of judgements

|  | **Judgement** | | | | | | |
| --- | --- | --- | --- | --- | --- | --- | --- |
| **Problem** | **No** | Probably no | Probably yes | Yes |  | Varies | Don't know |
| **Desirable Effects** | **Trivial** | Small | Moderate | Large |  | Varies | Don't know |
| **Undesirable Effects** | **Trivial** | Small | Moderate | Large |  | Varies | Don't know |
| **Certainty of evidence** | **Very low** | Low | Moderate | High |  |  | No included studies |
| **Values** | Important uncertainty or variability | **Possibly important uncertainty or variability** | Probably no important uncertainty or variability | No important uncertainty or variability |  |  |  |
| **Balance of effects** | Favors the comparison | Probably favors the comparison | **Does not favor either the intervention or the comparison** | Probably favors the intervention | Favors the intervention | Varies | Don't know |
| **Resources required** | Large costs | **Moderate costs** | Negligible costs and savings | Moderate savings | Large savings | Varies | Don't know |
| **Certainty of evidence of required resources** | Very low | Low | Moderate | High |  |  | **No included studies** |
| **Cost effectiveness** | Favors the comparison | Probably favors the comparison | Does not favor either the intervention or the comparison | Probably favors the intervention | Favors the intervention | Varies | **No included studies** |
| **Equity** | Reduced | Probably reduced | **Probably no impact** | Probably increased | Increased | Varies | Don't know |
| **Acceptability** | No | Probably no | Probably yes | Yes |  | **Varies** | Don't know |
| **Feasibility** | No | Probably no | Probably yes | Yes |  | **Varies** | Don't know |

# Type of recommendation

| Strong recommendation against the intervention | Conditional recommendation against the intervention | Conditional recommendation for either the intervention or the comparison | Conditional recommendation for the intervention | Strong recommendation for the intervention |
| --- | --- | --- | --- | --- |
| **x** | ○ | ○ | ○ | ○ |

# Conclusions

| Recommendation |
| --- |
| The panel advises against the routine use of massage therapy for GER/GERD symptoms in infants and children, due to current lack of supporting evidence for both efficacy and safety. |
| Justification |
|  |

| Subgroup considerations |
| --- |
| None |
| Implementation considerations |
| None |

| Monitoring and evaluation |
| --- |
| No current recommendations due to lack of standard of care and massage evaluation scores. |
| Research priorities |
| None |
| REFERENCES SUMMARY See the main text of PICO 6 and Additional File 2. |

## PICO 7. What are the indications and effectiveness of different surgical and endoscopic treatment options for GERD in infants, children, and adolescents?

| Question | |
| --- | --- |
| **Should Laparoscopic Nissen fundoplication compared to Open Nissen fundoplication be used for children with GER/GERD?** | |
| **Population:** | Children with GER/GERD |
| **Intervention:** | Laparoscopic Nissen fundoplication |
| **Comparison:** | Open Nissan fundoplication |
| **Main outcomes:** | - Mortality - Dysphagia - Gerd Recurrence - Anti-Secretory drug use - Retching - Dumping Syndrome - Gas bloat syndrome |
| **Setting:** | **inpatient hospital setting** |
| **Perspective:** | **CLINICAL RECOMENDATION** |
| **Background:** | GERD in children may require fundoplication surgery, with two main approaches: open fundoplication (OF) and laparoscopic fundoplication (LF). OF is the traditional method, while LF is a minimally invasive technique that has been shown to offer benefits in adults |
| **Conflict of interests:** | SIP (Italian Society of Pediatrics) conflict of interest declaration and management policies were applied and the following panel members were voting panel members (determining the direction and strength of the recommendation): All.  Panel members recused as a result of risk of conflicts of interest: None. |

# Assessment

| Problem Is the problem a priority? | | |
| --- | --- | --- |
| Judgement | Research evidence | Additional considerations |
| ○ No ○ Probably no ○ Probably yes **X Yes** ○ Varies ○ Don't know | Yes, the problem is a priority. GERD is a common issue in children, especially those with neurological impairments. The surgical treatments, laparoscopic and open fundoplication, have different outcomes that significantly impact recurrence rates, patient satisfaction, quality of life, and healthcare costs. |  |
| Desirable Effects How substantial are the desirable anticipated effects? | | |
| Judgement | Research evidence | Additional considerations |
| ○ Trivial ○ Small **X Moderate** ○ Large ○ Varies ○ Don't know | The desirable effects are substantial. Both laparoscopic and open fundoplication significantly reduce GERD symptoms, with substantial improvements in quality of life, nutritional status, and a reduction in the need for anti-secretory drugs. The studies show that both techniques lead to a decrease in symptoms like regurgitation and vomiting, with similar results in patient satisfaction. |  |
| Undesirable Effects How substantial are the undesirable anticipated effects? | | |
| Judgement | Research evidence | Additional considerations |
| ○ Trivial ○ Small **X Moderate** ○ Large ○ Varies ○ Don't know | Both laparoscopic and open fundoplication are generally safe, but laparoscopic surgery is associated with fewer instances of retching (6% vs. 56% for open surgery). However, recurrence rates are higher after laparoscopic surgery. Overall, both approaches have similar complication rates. |  |
| Certainty of evidence What is the overall certainty of the evidence of effects? | | |
| Judgement | Research evidence | Additional considerations |
| ○ Very low ○ Low **X Moderate** ○ High ○ No included studies | The overall certainty of the evidence is moderate. While the studies consistently show that both laparoscopic and open fundoplication improve outcomes like GERD symptoms, quality of life, and medication use, there is variability in recurrence rates, complications (e.g., retching), and surgical times. Some studies have small sample sizes or other potential biases, which reduces the confidence in the findings. Therefore, while the evidence is reliable, it is not of the highest certainty. |  |
| Values Is there important uncertainty about or variability in how much people value the main outcomes? | | |
| Judgement | Research evidence | Additional considerations |
| ○ Important uncertainty or variability ○ Possibly important uncertainty or variability **X Probably no important uncertainty or variability** ○ No important uncertainty or variability | There is limited uncertainty about how people value the main outcomes, as most prioritize improvements in GERD symptoms, quality of life, and reduced medication use, though some may vary in their preference for factors like recovery time or recurrence |  |
| Balance of effects Does the balance between desirable and undesirable effects favor the intervention or the comparison? | | |
| Judgement | Research evidence | Additional considerations |
| ○ Favors the comparison ○ Probably favors the comparison **X Does not favor either the intervention or the comparison** ○ Probably favors the intervention ○ Favors the intervention ○ Varies ○ Don't know | Since both laparoscopic and open fundoplication have:   - Similar benefits in terms of quality of life and symptom relief - Trade-offs (e.g., higher recurrence in lap, more retching in open)   Because the overall balance of effects is relatively equal, with each option having different advantages and disadvantages. |  |
| Resources required | | |
| Judgement | Research evidence | Additional considerations |
| ○ Large costs **X Moderate costs** ○ Negligible costs and savings ○ Moderate savings ○ Large savings ○ Varies ○ Don't know | While laparoscopic surgery has higher upfront costs and longer surgical time, the overall resource use (hospital stay, complications, readmission rates) is comparable between laparoscopic and open fundoplication. |  |
| Certainty of evidence of required resources What is the certainty of the evidence of resource requirements (costs)? | | |
| Judgement | Research evidence | Additional considerations |
| ○ Very low ○ Low **X Moderate** ○ High ○ No included studies | The certainty of evidence for resource requirements (costs) is moderate due to consistent reporting but with some variability in the data across studies. |  |
| Cost effectiveness Does the cost-effectiveness of the intervention favor the intervention or the comparison? | | |
| Judgement | Research evidence | Additional considerations |
| ○ Favors the comparison **X Probably favors the comparison** ○ Does not favor either the intervention or the comparison ○ Probably favors the intervention ○ Favors the intervention ○ Varies ○ No included studies | Cost-effectiveness: Probably favors the comparison (open fundoplication).  The cost-effectiveness likely favors the comparison (open fundoplication) due to lower initial surgical costs, and similar resource use (hospital stay, complications, and readmissions) between the two approaches.  Although laparoscopic surgery has some advantages in terms of fewer complications, its higher initial costs and higher recurrence rates make open fundoplication potentially more cost-effective overall. |  |
| Equity What would be the impact on health equity? | | |
| Judgement | Research evidence | Additional considerations |
| ○ Reduced ○ Probably reduced **X Probably no impact** ○ Probably increased ○ Increased ○ Varies ○ Don't know | Both approaches are accessible in various settings and provide similar outcomes, with no major disparities in how they impact different population groups |  |
| Acceptability Is the intervention acceptable to key stakeholders? | | |
| Judgement | Research evidence | Additional considerations |
| ○ No ○ Probably no ○ Probably yes **X Yes** ○ Varies ○ Don't know | Does not favor either the intervention or the comparison, as both procedures are generally well-accepted by patients, parents, and healthcare providers, depending on the setting and resources available. |  |
| Feasibility Is the intervention feasible to implement? | | |
| Judgement | Research evidence | Additional considerations |
| ○ No ○ Probably no **x Probably yes** ○ Yes ○ Varies ○ Don't know | Laparoscopic fundoplication is probably feasible in many settings, particularly in those with adequate resources and surgical expertise. |  |

# Summary of judgements

|  | **Judgement** | | | | | | |
| --- | --- | --- | --- | --- | --- | --- | --- |
| **Problem** | No | Probably no | Probably yes | **Yes** |  | Varies | Don't know |
| **Desirable Effects** | Trivial | Small | **Moderate** | Large |  | Varies | Don't know |
| **Undesirable Effects** | Trivial | Small | **Moderate** | Large |  | Varies | Don't know |
| **Certainty of evidence** | Very low | Low | **Moderate** | High |  |  | No included studies |
| **Values** | Important uncertainty or variability | Possibly important uncertainty or variability | **Probably no important uncertainty or variability** | No important uncertainty or variability |  |  |  |
| **Balance of effects** | Favors the comparison | Probably favors the comparison | **Does not favor either the intervention or the comparison** | Probably favors the intervention | Favors the intervention | Varies | Don't know |
| **Resources required** | Large costs | **Moderate costs** | Negligible costs and savings | Moderate savings | Large savings | Varies | Don't know |
| **Certainty of evidence of required resources** | Very low | Low | **Moderate** | High |  |  | No included studies |
| **Cost effectiveness** | Favors the comparison | **Probably favors the comparison** | Does not favor either the intervention or the comparison | Probably favors the intervention | Favors the intervention | Varies | No included studies |
| **Equity** | Reduced | Probably reduced | **Probably no impact** | Probably increased | Increased | Varies | Don't know |
| **Acceptability** | No | Probably no | Probably yes | **Yes** |  | Varies | Don't know |
| **Feasibility** | No | Probably no | **Probably yes** | Yes |  | Varies | Don't know |

# Type of recommendation

| Strong recommendation against the intervention | Conditional recommendation against the intervention | Conditional recommendation for either the intervention or the comparison | Conditional recommendation for the intervention | Strong recommendation for the intervention |
| --- | --- | --- | --- | --- |
| ○ | ○ | ○ | ○ | ○ |

# Conclusions

| Recommendation |
| --- |
| Both laparoscopic and open fundoplication are effective treatments for pediatric GERD. However, the choice between the two should be based on factors such as recurrence rates, surgical time, complication rates, patient preferences, and the available resources. |
| Justification |
| The recommendation that both laparoscopic and open fundoplication are effective treatments for pediatric GERD and that the choice should depend on recurrence rates, surgical time, complications, and patient preferences is well-supported by the available evidence. It balances clinical outcomes with practical considerations related to costs, resources, and patient needs, ensuring a comprehensive and patient-centered approach to treatment decision-making. |

| Subgroup considerations |
| --- |
| The choice between laparoscopic and open fundoplication for pediatric GERD should consider subgroups such as neurological impairment, age, comorbidities, obesity, healthcare setting, and patient preferences. |
| Implementation considerations |
| Laparoscopic fundoplication requires specialized training, access to advanced equipment, higher costs, and thorough postoperative follow-up to monitor for complications and GERD recurrence. |

| Monitoring and evaluation |
| --- |
| Monitor postoperative complications, GERD recurrence, and long-term outcomes, with ongoing feedback from patients and surgical teams to guide continuous improvement |
| Research priorities |
| In children, based on available RCTs, laparoscopic fundoplication does not consistently show superior outcomes compared to open surgery. However, data from retrospective series in children, as well as data from adults, generally favor the laparoscopic approach due to advantages like shorter recovery times, fewer complications, and potential long-term cost savings. These findings highlight the need to understand the differences in outcomes between children and adults. |
| REFERENCES SUMMARY See the main text of PICO 7 and Additional File 2. |

| Question | |
| --- | --- |
| **Should Laparoscopic Nissen fundoplication compared to Thal fundoplication be used for children with GER/GERD?** | |
| **Population:** | Children with GER/GERD |
| **Intervention:** | Laparoscopic Nissan fundoplication |
| **Comparison:** | Thal fundoplication |
| **Main outcomes:** | - Mortality - Dysphagia - Gerd Recurrence - Anti-Secretory drug use - Retching - Dumping Syndrome - Gas bloat syndrome |
| **Setting:** | **inpatient hospital setting** |
| **Perspective:** | **CLINICAL RECOMENDATION** |
| **Background:** | Surgery for GERD is indicated for children with refractory GERD. Current research does not provide clear guidance on whether complete or partial fundoplication is the superior technique for treating GERD children. |
| **Conflict of interests:** | SIP (Italian Society of Pediatrics) conflict of interest declaration and management policies were applied and the following panel members were voting panel members (determining the direction and strength of the recommendation): All.  Panel members recused as a result of risk of conflicts of interest: None. |

# Assessment

| Problem Is the problem a priority? | | |
| --- | --- | --- |
| Judgement | Research evidence | Additional considerations |
| ○ No ○ Probably no **X Probably yes** ○ Yes ○ Varies ○ Don't know | Postoperative complications such as severe dysphagia and recurrent symptoms directly impact patients' quality of life, making it necessary to prioritize research and decisions around surgical approaches to minimize these issues. |  |
| Desirable Effects How substantial are the desirable anticipated effects? | | |
| Judgement | Research evidence | Additional considerations |
| ○ Trivial ○ Small **X Moderate** ○ Large ○ Varies ○ Don't know | Nissen: Significantly lower long-term recurrence and failure rates (Study 3: 5.9% vs 15.9%, P = 0.038), especially in neurologically impaired children.  Thal: Still effective, though with higher rates of redo fundoplication and absolute failure.  Desirable effects are greater in the Nissen group, particularly for long-term reflux control in high-risk populations. |  |
| Undesirable Effects How substantial are the undesirable anticipated effects? | | |
| Judgement | Research evidence | Additional considerations |
| ○ Trivial ○ Small **X Moderate** ○ Large ○ Varies ○ Don't know | Dysphagia occurs similarly in both groups but is more severe in the Nissen group, requiring more interventions.  Intraoperative complications were rare but present in both groups.  Mortality is not directly linked to the type of fundoplication in most cases but is high overall due to comorbidities (35% mortality at follow-up).  Overall, undesirable effects are more pronounced in Nissen, though manageable. |  |
| Certainty of evidence What is the overall certainty of the evidence of effects? | | |
| Judgement | Research evidence | Additional considerations |
| ○ Very low ○ Low **X Moderate** ○ High ○ No included studies | Data comes from only three studies, all conducted by a single research group, limiting generalizability.  Study sizes are moderate, and results are generally consistent, but more independent studies are needed for higher certainty. |  |
| Values Is there important uncertainty about or variability in how much people value the main outcomes? | | |
| Judgement | Research evidence | Additional considerations |
| ○ Important uncertainty or variability **X Possibly important uncertainty or variability** ○ Probably no important uncertainty or variability ○ No important uncertainty or variability | There is important uncertainty and variability about how much people value the main outcomes of Nissen fundoplication and Thal fundoplication. While the studies provide valuable insights, the variability in values and the uncertainty about patient priorities must be taken into account when interpreting the results and making clinical decisions.  Families may vary in how they value long-term reflux control versus avoiding dysphagia and complications.  Particularly in high-risk populations, values can differ based on the child’s baseline health and prognosis. |  |
| Balance of effects Does the balance between desirable and undesirable effects favor the intervention or the comparison? | | |
| Judgement | Research evidence | Additional considerations |
| ○ Favors the comparison ○ Probably favors the comparison ○ Does not favor either the intervention or the comparison ○ Probably favors the intervention ○ Favors the intervention **X Varies** ○ Don't know | Favors Nissen in high-risk populations due to better long-term reflux control and lower failure rates.  However, in children where severe dysphagia risk or surgical complexity is a concern, Thal may be preferred.  Evidence is limited, and decisions should be individualized. |  |
| Resources required | | |
| Judgement | Research evidence | Additional considerations |
| ○ Large costs ○ Moderate costs ○ Negligible costs and savings ○ Moderate savings ○ Large savings ○ Varies **x Don't know** | The studies reviewed do not provide comprehensive details on the exact medical, surgical, or facility resources needed. |  |
| Certainty of evidence of required resources What is the certainty of the evidence of resource requirements (costs)? | | |
| Judgement | Research evidence | Additional considerations |
| **X Very low** ○ Low ○ Moderate ○ High ○ No included studies | None of the studies provide direct data on costs, length of stay, or resource utilization, limiting any conclusions on economic impact. |  |
| Cost effectiveness Does the cost-effectiveness of the intervention favor the intervention or the comparison? | | |
| Judgement | Research evidence | Additional considerations |
| ○ Favors the comparison ○ Probably favors the comparison ○ Does not favor either the intervention or the comparison ○ Probably favors the intervention ○ Favors the intervention ○ Varies **X No included studies** | Unclear.  Nissen may offer better long-term outcomes, possibly reducing future GERD-related costs, but at the expense of higher complication-related costs.  Thal may have lower short-term costs but higher recurrence, possibly increasing long-term treatment needs. No formal cost-effectiveness analysis is available. |  |
| Equity What would be the impact on health equity? | | |
| Judgement | Research evidence | Additional considerations |
| ○ Reduced ○ Probably reduced ○ Probably no impact ○ Probably increased ○ Increased ○ Varies **x Don't know** | Without specific data on health equity, the impact on equity depends largely on access to care, cost, and the availability of long-term support services. |  |
| Acceptability Is the intervention acceptable to key stakeholders? | | |
| Judgement | Research evidence | Additional considerations |
| ○ No ○ Probably no ○ Probably yes ○ Yes **x Varies** ○ Don't know | Both Nissen and Thal fundoplication have their merits and drawbacks in terms of acceptability. For families, the long-term benefits of Nissen may be more acceptable despite the increased risk of dysphagia and complications, especially in high-risk populations. Conversely, Thal may be more acceptable in the short term due to its simpler recovery and lower immediate complication rates, but the higher recurrence rate of GERD may reduce its acceptability for families seeking a more lasting solution. |  |
| Feasibility Is the intervention feasible to implement? | | |
| Judgement | Research evidence | Additional considerations |
| ○ No ○ Probably no ○ Probably yes ○ Yes ○ Varies **X Don't know** | The studies reviewed do not provide evidence on differences in surgical expertise or complexity between the procedures. |  |

# Summary of judgements

|  | **Judgement** | | | | | | |
| --- | --- | --- | --- | --- | --- | --- | --- |
| **Problem** | No | Probably no | **Probably yes** | Yes |  | Varies | Don't know |
| **Desirable Effects** | Trivial | Small | **Moderate** | Large |  | Varies | Don't know |
| **Undesirable Effects** | Trivial | Small | **Moderate** | Large |  | Varies | Don't know |
| **Certainty of evidence** | Very low | Low | **Moderate** | High |  |  | No included studies |
| **Values** | Important uncertainty or variability | **Possibly important uncertainty or variability** | Probably no important uncertainty or variability | No important uncertainty or variability |  |  |  |
| **Balance of effects** | Favors the comparison | Probably favors the comparison | Does not favor either the intervention or the comparison | Probably favors the intervention | Favors the intervention | **Varies** | Don't know |
| **Resources required** | Large costs | Moderate costs | Negligible costs and savings | Moderate savings | Large savings | Varies | **Don't know** |
| **Certainty of evidence of required resources** | **Very low** | Low | Moderate | High |  |  | No included studies |
| **Cost effectiveness** | Favors the comparison | Probably favors the comparison | Does not favor either the intervention or the comparison | Probably favors the intervention | Favors the intervention | Varies | **No included studies** |
| **Equity** | Reduced | Probably reduced | Probably no impact | Probably increased | Increased | Varies | **Don't know** |
| **Acceptability** | No | Probably no | Probably yes | Yes |  | **Varies** | Don't know |
| **Feasibility** | No | Probably no | Probably yes | Yes |  | Varies | **Don't know** |

# Type of recommendation

| Strong recommendation against the intervention | Conditional recommendation against the intervention | Conditional recommendation for either the intervention or the comparison | Conditional recommendation for the intervention | Strong recommendation for the intervention |
| --- | --- | --- | --- | --- |
| ○ | ○ | ○ | ○ | ○ |

# Conclusions

| Recommendation |
| --- |
| Either Nissen or Thal fundoplication may be appropriate for high-risk pediatric patients, including those with neurological impairment.  Current evidence suggests that Nissen fundoplication may offer better long-term reflux control and lower recurrence, but it is associated with a higher risk of severe postoperative dysphagia.  Thal fundoplication may be associated with fewer immediate postoperative complications, but possibly a higher rate of long-term GERD recurrence.  Given the limited number of studies, all from a single group, and the lack of data on cost, feasibility, and equity, the choice between procedures should be made on a case-by-case basis, considering the patient's clinical status, caregiver preferences, and available resources. |
|  |
| Justification |
| Evidence from three RCTs suggests that Nissen fundoplication is associated with lower long-term recurrence and failure rates, particularly in children with neurological impairment. However, it also carries a higher risk of severe postoperative dysphagia, often requiring intervention.  Thal fundoplication shows a trend toward fewer severe complications but has a higher rate of GERD recurrence and redo fundoplication. The overall mortality in these studies was related more to underlying conditions than to the surgical technique.  Since the studies come from a single research group, and there is no data on cost-effectiveness, resource use, or patient-centered outcomes, the certainty of the evidence is low. Therefore, a balanced and individualized approach is warranted. |

| Subgroup considerations |
| --- |
| Neurologically Impaired Children: This subgroup shows greater benefit from Nissen fundoplication, with significantly lower recurrence and failure rates compared to Thal. However, they are also more vulnerable to postoperative dysphagia, which was more frequent and severe in the Nissen group. Careful postoperative monitoring and feeding support are critical.  Children Without Neurological Impairment: In children with normal neurological status, the differences between Nissen and Thal are less pronounced. Recurrence and complication rates were similar, making either procedure a reasonable option depending on surgical expertise and individual patient factors. |
| Implementation considerations |
| Successful implementation depends on matching the surgical approach to patient needs and institutional capacity, ensuring multidisciplinary support, and maintaining robust follow-up systems |

| Monitoring and evaluation |
| --- |
| Monitor postoperative dysphagia, GERD recurrence, complications, and long-term outcomes, with ongoing feedback from patients and surgical teams to guide continuous improvement |
| Research priorities |
| Research should focus on long-term outcomes, cost-effectiveness, equity impact in resource-limited settings, and conducting multicenter, large-scale studies for more robust data. |
| REFERENCES SUMMARY See the main text of PICO 7 and Additional File 2. |

| Question | |
| --- | --- |
| **Should Laparoscopic Nissen fundoplication compared to Hill-Snow procedure be used for children with GER/GERD?** | |
| **Population:** | Children with GER/GERD |
| **Intervention:** | Laparoscopic Nissen fundoplication |
| **Comparison:** | Hill-Snow procedure |
| **Main outcomes:** | - Mortality - Dysphagia - Gerd Recurrence - Anti-Secretory drug use - Retching - Dumping Syndrome - Gas bloat syndrome |
| **Setting:** | **INPATIENTS** |
| **Perspective:** | **CLINICAL RECOMENDATION** |
| **Background:** | Surgery for GERD is indicated for children with refractory GERD. Current research does not provide clear guidance on whether complete or partial fundoplication is the superior technique for treating GERD children. |
| **Conflict of interests:** | SIP (Italian Society of Pediatrics) conflict of interest declaration and management policies were applied and the following panel members were voting panel members (determining the direction and strength of the recommendation): All.  Panel members recused as a result of risk of conflicts of interest: None. |

# Assessment

| Problem Is the problem a priority? | | |
| --- | --- | --- |
| Judgement | Research evidence | Additional considerations |
| ○ No ○ Probably no **x Probably yes** ○ Yes ○ Varies ○ Don't know | Management of refractory GERD in children is a high-priority issue due to its significant impact on health, quality of life, and healthcare use. Identifying safer, effective alternatives to Nissen fundoplication—such as the Hill-Snow procedure—may help improve outcomes for these patients. |  |
| Desirable Effects How substantial are the desirable anticipated effects? | | |
| Judgement | Research evidence | Additional considerations |
| **x Trivial** ○ Small ○ Moderate ○ Large ○ Varies ○ Don't know | The desirable effects of the Hill-Snow procedure appear to be substantial, particularly in terms of reducing complications while maintaining symptom control. However, due to the very low certainty of the evidence, these findings should be interpreted with caution. If future studies confirm these benefits, the Hill-Snow procedure could become a preferred alternative to complete fundoplication in selected pediatric patients. |  |
| Undesirable Effects How substantial are the undesirable anticipated effects? | | |
| Judgement | Research evidence | Additional considerations |
| **x Trivial** ○ Small ○ Moderate ○ Large ○ Varies ○ Don't know | Based on current evidence, the undesirable effects of the Hill-Snow procedure are likely small to moderate in magnitude and may be less frequent than those associated with complete Nissen fundoplication (e.g., less bloating and dysphagia). However, due to the very low certainty of evidence and lack of long-term data, there is meaningful uncertainty about the frequency and severity of these effects. As such, these potential downsides must be weighed carefully when considering the procedure, and patients should be informed about the limited data supporting its use. |  |
| Certainty of evidence What is the overall certainty of the evidence of effects? | | |
| Judgement | Research evidence | Additional considerations |
| **x Very low** ○ Low ○ Moderate ○ High ○ No included studies | The overall certainty of the evidence for the Hill-Snow procedure is very low, meaning that there is very limited confidence in the estimated effects, and the true effect may be substantially different from what the single available study suggests. High-quality, well-designed randomized controlled trials are needed to increase the certainty of evidence and guide clinical decision-making. |  |
| Values Is there important uncertainty about or variability in how much people value the main outcomes? | | |
| Judgement | Research evidence | Additional considerations |
| ○ Important uncertainty or variability ○ Possibly important uncertainty or variability **x Probably no important uncertainty or variability** ○ No important uncertainty or variability | The Hill-Snow procedure may be favored by some groups who prioritize reducing complications and shortening recovery, while others may prefer the more established Nissen fundoplication for its higher certainty of long-term symptom control. |  |
| Balance of effects Does the balance between desirable and undesirable effects favor the intervention or the comparison? | | |
| Judgement | Research evidence | Additional considerations |
| ○ Favors the comparison ○ Probably favors the comparison ○ Does not favor either the intervention or the comparison **x Probably favors the intervention** ○ Favors the intervention ○ Varies ○ Don't know | At present, the balance of effects between the desirable and undesirable outcomes of the Hill-Snow procedure seems to favor the intervention in terms of reducing postoperative complications, particularly bloating and dysphagia, with similar efficacy in controlling reflux symptoms. However, the limited evidence base means that these conclusions are based on preliminary findings and may change as more data become available. Given the potential for fewer complications and quicker recovery times, the Hill-Snow procedure presents a promising option, but further high-quality studies are necessary to fully establish its long-term benefits and risks. |  |
| Resources required | | |
| Judgement | Research evidence | Additional considerations |
| ○ Large costs ○ Moderate costs ○ Negligible costs and savings ○ Moderate savings ○ Large savings ○ Varies **x Don't know** | No data available. |  |
| Certainty of evidence of required resources What is the certainty of the evidence of resource requirements (costs)? | | |
| Judgement | Research evidence | Additional considerations |
| ○ Very low ○ Low ○ Moderate ○ High **x No included studies** | At present, the certainty of evidence regarding the resource requirements (costs) of the Hill-Snow procedure is low due to the limited availability of relevant data. While it is plausible that the procedure may result in cost savings related to fewer complications and shorter recovery times. |  |
| Cost effectiveness Does the cost-effectiveness of the intervention favor the intervention or the comparison? | | |
| Judgement | Research evidence | Additional considerations |
| ○ Favors the comparison ○ Probably favors the comparison ○ Does not favor either the intervention or the comparison **x Probably favors the intervention** ○ Favors the intervention ○ Varies ○ No included studies | Although a definitive economic assessment remains absent, the Hill-Snow procedure demonstrates a lower occurrence of complications relative to the Nissen technique, which is likely to result in decreased healthcare expenses. |  |
| Equity What would be the impact on health equity? | | |
| Judgement | Research evidence | Additional considerations |
| ○ Reduced ○ Probably reduced **x Probably no impact** ○ Probably increased ○ Increased ○ Varies ○ Don't know | No data available- |  |
| Acceptability Is the intervention acceptable to key stakeholders? | | |
| Judgement | Research evidence | Additional considerations |
| ○ No ○ Probably no **x Probably yes** ○ Yes ○ Varies ○ Don't know | Clinicians: Hill-Snow procedure may be seen as an appealing option due to the reduced risk of complications. However, the limited evidence base may influence its widespread adoption.  Caregivers/Parents and Patients: They would likely benefit from a procedure with fewer complications, although the long-term efficacy and safety remain uncertain. |  |
| Feasibility Is the intervention feasible to implement? | | |
| Judgement | Research evidence | Additional considerations |
| ○ No ○ Probably no **x Probably yes** ○ Yes ○ Varies ○ Don't know | The Hill-Snow procedure could be feasible to implement, particularly in settings with adequate surgical expertise. However, widespread adoption may be constrained by the limited evidence supporting its efficacy and safety. |  |

# Summary of judgements

|  | **Judgement** | | | | | | |
| --- | --- | --- | --- | --- | --- | --- | --- |
| **Problem** | No | Probably no | **Probably yes** | Yes |  | Varies | Don't know |
| **Desirable Effects** | **Trivial** | Small | Moderate | Large |  | Varies | Don't know |
| **Undesirable Effects** | **Trivial** | Small | Moderate | Large |  | Varies | Don't know |
| **Certainty of evidence** | **Very low** | Low | Moderate | High |  |  | No included studies |
| **Values** | Important uncertainty or variability | Possibly important uncertainty or variability | **Probably no important uncertainty or variability** | No important uncertainty or variability |  |  |  |
| **Balance of effects** | Favors the comparison | Probably favors the comparison | Does not favor either the intervention or the comparison | **Probably favors the intervention** | Favors the intervention | Varies | Don't know |
| **Resources required** | Large costs | Moderate costs | Negligible costs and savings | Moderate savings | Large savings | Varies | **Don't know** |
| **Certainty of evidence of required resources** | Very low | Low | Moderate | High |  |  | **No included studies** |
| **Cost effectiveness** | Favors the comparison | Probably favors the comparison | Does not favor either the intervention or the comparison | **Probably favors the intervention** | Favors the intervention | Varies | No included studies |
| **Equity** | Reduced | Probably reduced | **Probably no impact** | Probably increased | Increased | Varies | Don't know |
| **Acceptability** | No | Probably no | **Probably yes** | Yes |  | Varies | Don't know |
| **Feasibility** | No | Probably no | **Probably yes** | Yes |  | Varies | Don't know |

# Type of recommendation

| Strong recommendation against the intervention | Conditional recommendation against the intervention | Conditional recommendation for either the intervention or the comparison | Conditional recommendation for the intervention | Strong recommendation for the intervention |
| --- | --- | --- | --- | --- |
| ○ | ○ | ○ | **x** | ○ |

# Conclusions

| Recommendation |
| --- |
| The panel suggests considering the Hill-Snow procedure, a partial fundoplication, as an alternative to complete Nissen fundoplication, as it has been associated with a lower incidence of postoperative complications (such as bloating and dysphagia) while maintaining similar efficacy in symptom control. However, as this recommendation is based on a single study, the quality of evidence is very limited, and no strong conclusions can be drawn. |
| Justification |
| This recommendation is derived from a single study, and as such, the overall quality of evidence is considered very limited. The findings should be interpreted with caution, as the study has small sample size and methodological limitations. |

| Subgroup considerations |
| --- |
| Not applicable. |
| Implementation considerations |
| The efficacy and safety of the Hill-Snow procedure as an alternative to complete Nissen fundoplication should be evaluated in larger randomized controlled trials. Improved patient stratification is pivotal to accurately assess outcomes. |

| Monitoring and evaluation |
| --- |
| Systematic tracking of clinical results, combined with thorough long-term evaluations, could help determine the relevance of Hill-Snow procedure in the treatment of pediatric refractory GERD. |
| Research priorities |
| There is limited evidence to ascertain whether a complete or partial fundoplication is the optimal surgical approach for addressing refractory GERD in the pediatric population. Current findings are not sufficient to establish definitive conclusions regarding comparative efficacy and safety in this age group. Therefore, well-designed, adequately powered RCTs are needed to evaluate the most effective surgical interventions with reduced morbidity for children experiencing refractory GERD. |
| REFERENCES SUMMARY See the main text of PICO 7 and Additional File 2. |
